# Spin effect on redox acceleration and regioselectivity in Fe-catalyzed alkyne hydrosilylation

**DOI:** 10.1093/nsr/nwad324

**Published:** 2023-12-20

**Authors:** Peng He, Meng-Yang Hu, Jin-Hong Li, Tian-Zhang Qiao, Yi-Lin Lu, Shou-Fei Zhu

**Affiliations:** Frontiers Science Center for New Organic Matter, State Key Laboratory and Institute of Elemento-Organic Chemistry, Nankai University, Tianjin 300071, China; Frontiers Science Center for New Organic Matter, State Key Laboratory and Institute of Elemento-Organic Chemistry, Nankai University, Tianjin 300071, China; Frontiers Science Center for New Organic Matter, State Key Laboratory and Institute of Elemento-Organic Chemistry, Nankai University, Tianjin 300071, China; Frontiers Science Center for New Organic Matter, State Key Laboratory and Institute of Elemento-Organic Chemistry, Nankai University, Tianjin 300071, China; Frontiers Science Center for New Organic Matter, State Key Laboratory and Institute of Elemento-Organic Chemistry, Nankai University, Tianjin 300071, China; Frontiers Science Center for New Organic Matter, State Key Laboratory and Institute of Elemento-Organic Chemistry, Nankai University, Tianjin 300071, China

**Keywords:** spin effect, spin-delocalization, spin crossover, iron-catalysis, alkyne hydrosilylation

## Abstract

Iron catalysts are ideal transition metal catalysts because of the Earths abundant, cheap, biocompatible features of iron salts. Iron catalysts often have unique open-shell structures that easily undergo spin crossover in chemical transformations, a feature rarely found in noble metal catalysts. Unfortunately, little is known currently about how the open-shell structure and spin crossover affect the reactivity and selectivity of iron catalysts, which makes the development of iron catalysts a low efficient trial-and-error program. In this paper, a combination of experiments and theoretical calculations revealed that the iron-catalyzed hydrosilylation of alkynes is typical spin-crossover catalysis. Deep insight into the electronic structures of a set of well-defined open-shell active formal Fe(0) catalysts revealed that the spin-delocalization between the iron center and the 1,10-phenanthroline ligand effectively regulates the iron center's spin and oxidation state to meet the opposite electrostatic requirements of oxidative addition and reductive elimination, respectively, and the spin crossover is essential for this electron transfer process. The triplet transition state was essential for achieving high regioselectivity through tuning the nonbonding interactions. These findings provide an important reference for understanding the effect of catalyst spin state on reaction. It is inspiring for the development of iron catalysts and other Earth-abundant metal catalysts, especially from the point of view of ligand development.

## Teaser

The electron spin effect in iron-catalysis was disclosed through a combination of experiments and theoretical calculations.

## INTRODUCTION

Spin is an intrinsic property of electrons, and studies on electron spin have been at the forefront of materials science and interdisciplinary fields [1–4]. Spin crossover phenomena are common in open-shell metal complexes and have a wide range of applications in the field of materials science, such as spin-crossover sensors [5] and molecular spintronic materials [6]. In the field of transition metal catalysis, the effect of the catalyst spin state on chemical reactions has also received increasing attention [7–9]. Studies on the effect of catalyst spin state are of great value for the development of first row transition metal (3d metal) catalysts. For a long time, precious metal catalysts, especially those based on 4d and 5d metals, have dominated the scientific research and production applications of transition metal catalysis. However, the scarce and non-renewable resources, high prices, and poor biocompatibility of 4d and 5d metals are increasingly becoming factors in limiting their application. Therefore, 3d metal catalysts, especially iron catalysts, have attracted much attention in recent years because of the abundant resources, low prices, and good biocompatibility of their central metals [10,11]. The 3d metal catalysts and 4d or 5d metal catalysts have significant differences in electronic structures. According to crystal field theory, 4d or 5d metals, such as Pd and Pt, have large crystal field splitting energies and 4d or 5d metal complexes tend to be dominated by double electron transfer in the reaction with closed-shell electronic states. In such a case, the catalysts always maintain a single spin state (generally singlet), and the corresponding catalytic processes can be called ‘spin-constant catalysis’. By contrast, 3d metals, typically iron, have small crystal field splitting energies, so 3d metal complexes are prone to form open-shell structures, which usually have unique properties different from those of closed-shell metal catalysts [12]. Open-shell catalysts with different spin states might also have different catalytic properties [13]. Moreover, open-shell catalysts can undergo spin crossover in catalytic reactions, thus affecting the reaction process and showing a unique ‘two-state/multi-state reactivity’ (TSR/MSR) [14]. Such a catalytic process can be called ‘spin-crossover catalysis’. In fact, the promotion of reactions by spin crossover in 3d metal catalysis has been proposed for a long time and has been widely applied for explaining bioinorganic catalysis [15]. In recent years, the effect of spin state on transition metal catalysis has also received increasing attention [16–20]. Several unique properties of open-shell metal catalysts have been disclosed. For example, high-spin iron carbene, imido and oxo tend to have significant free radical properties and are prone to single electron transfer reactions [21–23]. The high spin catalyst's 3d-orbitals are occupied by unbonded electrons, making it immune to the common Lewis bases [18,24]. The spin crossover effect of some open-shell catalysts has also been studied. For example, in the reactions of Fe/Co-catalyzed C−H bond activation, the transformation of catalyst from high spin state to low spin state provides a vacant metal 3d-orbital for the coordination and activation of the C−H bond and the spin crossover in these processes may be partly attributed to the change of coordination shape of the catalysts [16–18,20,25–27]. Another research article concluded that metal-oxo enzymes/synthetic reagents showed significant exchange-enhanced reactivity in the process of chemical bond activation [8]. There is no doubt that the above research has greatly promoted the understanding of open-shell catalysts; however, little is known about how, exactly, the spin state affects the reactivity and selectivity of catalysts, which has become a bottleneck in the development of 3d metal catalysis.

**Figure 1.**
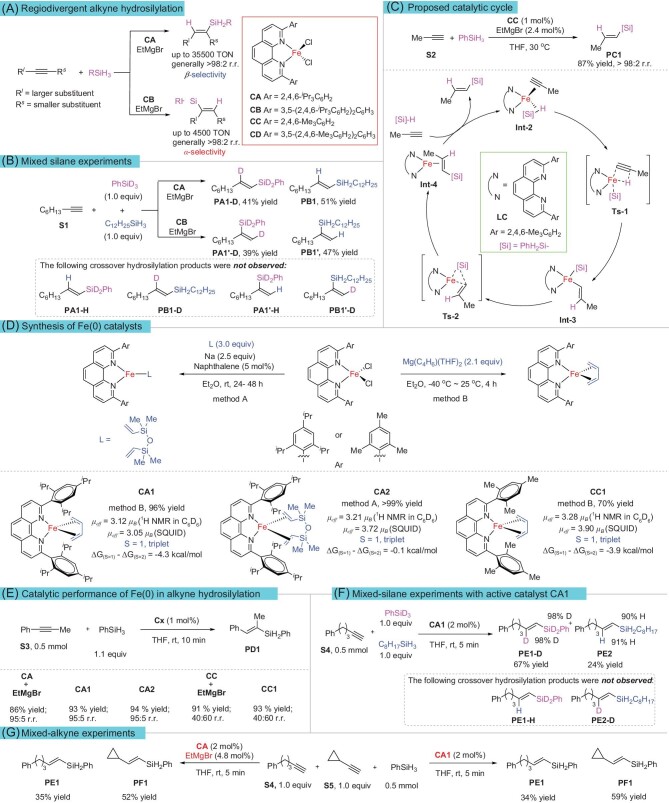
(A) Iron-catalyzed regiodivergent alkyne hydrosilylation from reference [30]. (B) Mixed silane experiments from reference [30]. (C) Proposed catalytic cycle. (D) Synthesis of Fe(0) catalysts. Magnetic moments were detected by Evans method or superconducting quantum interference device (SQUID); DFT calculations were performed at ωB97XD/def2TZVPP-CPCM (THF) || ωB97XD/6–311g*-TZVP (Fe) level. (E) Catalytic performance of Fe(0) in alkyne hydrosilylation. (F) Mixed silane experiments with active catalyst CA1. (G) Mixed alkyne experiments.

Recently, our research group has developed a series of iron complexes of 1,10-phenanthroline ligands, which can efficiently catalyze the addition reactions of various alkenes and alkynes [28–34]. Not long ago, we reported the regioselectivity divergent hydrosilylation of alkynes catalyzed by the 1,10-phenanthroline-iron complex and found that the regioselectivity of the reaction could be completely reversed by simply changing the aryl substituent at the 2,9-position of the ligand (Fig. 1A) [30]. In this study, we carried out in-depth research on the above reaction mechanism through the preparation and characterization of active catalysts combined with theoretical calculations. It was found for the first time that there is a typical two-state reactivity in the iron-catalyzed hydrosilylation of alkynes, in which a triplet iron catalyst promotes the oxidative addition process, while a quintet iron catalyst promotes the reductive elimination process. The prominent spin effect of the iron catalyst is the fundamental reason for the excellent activity of the reaction. The active iron catalysts were synthesized and characterized by single-crystal X-ray diffraction, Mössbauer spectroscopy, X-ray photoelectron spectroscopy (XPS), and magnetic measurements, etc. to get deep insight into the electronic structure of Fe-phenanthroline complexes. It was found that the active iron catalysts exhibited almost the same catalytic performances with the corresponding catalyst precursors (reduced *in situ*) in the hydrosilylation of alkynes. We further established a ‘Central Metal Charge Analysis’ (CMCA) method to study the deep-seated mechanism of oxidative addition and reductive elimination promoted by the spin crossover of the iron catalyst. Combined experiments and calculations revealed that 1,10-phenanthroline, acting as a kind of redox active ligand, could promote electron transfer between the iron center and the ligand through spin-delocalization and then adjust the oxidation state of the iron center to meet the electronic requirements of oxidative addition and reductive elimination. Finally, we also found that iron catalysts with specific spin states help in achieving the precise control of regioselectivity by affecting multiple nonbonding interactions between ligands and substrates. Because spin delocalization not only facilitates the spin crossover of the catalyst but also regulates the activity and selectivity of the catalyst, this spin effect of the open-shell catalyst could be defined as ‘Spin Delocalization Regulated Reactivity’ (SDRR). Although spin-delocalization has been reported in other iron catalysts with a redox-active ligand [35–37], little is known about how it affects catalytic properties. Since oxidative addition and reductive elimination are two important elementary steps in many transition metal catalytic reactions, hopefully, the regulations of spin effect disclosed in this study could be extended to other 2e redox catalytic procedures promoted by other open-shell catalysts.

## RESULTS AND DISCUSSION

In a previous study [30], mixed-silane experiments showed that a hydrogen atom and a silyl group reacted with the C–C triple bond from the same silane (Fig. 1B). This clearly indicated that the reaction was not initiated by Fe–H or Fe–Si species; otherwise, crossing-hydrosilylation products (**PA1-H, PB1-D, PA1′-H**, and **PB1′-D**) would be generated. Accordingly, an Fe(0)–Fe(II) catalytic cycle was then proposed (Fig. 1C). In this process, the catalyst precursor **CC** is reduced to Fe(0) by EtMgBr, which first coordinates with propyne and phenylsilane to form **Int-2. Int-2** then promotes the migration of hydrogen atoms on the phenylsilane to the C≡C triple bond via the transition state **Ts-1** by ligand-to-ligand hydrogen transfer to form **Int-3**. The **Int-3** then undergoes reductive elimination through **Ts-2** to afford **Int-4**. Finally, **Int-4** undergoes substrate exchange with the alkyne and silane to release the product **PC1** and re-generate **Int-2** for another catalytic cycle. It is always desirable to isolate an intermediate in the catalytic cycle, but such attempts usually fail because the intermediates are extremely active and short lived. The suboptimal goal to synthesize an alkyne-coordinated iron complex also failed because both terminal and internal alkynes underwent trimerization to afford benzene derivatives. Finally, we prepared a series of alkene-coordinated formal Fe(0) complexes **CA1, CA2** and **CC1** (Fig. 1D and Figs S1–S4), analogues of **Int-4**. We measured the magnetic moments of the above catalysts both by Evans method and with a superconducting quantum interference device (SQUID), and we found that the ground states of **CA1, CA2**, and **CC1** with diene coordination were triplet (S = 1). Meanwhile, DFT calculations revealed that the energies of triplet **CA1, CA2**, and **CC1** are lower than that of the corresponding quintet states, indicating triplet ground states. Next we systematically evaluated the catalytic performances of these active Fe(0) catalysts in the hydrosilylation reaction of alkynes. All three Fe(0) complexes mentioned above were catalytically active without an additional activator in the alkyne hydrosilylation reaction, giving almost the same result as the reaction promoted by the *in-situ*-generated catalyst from precursor **CA** and EtMgBr (Fig. 1E and Figs S1–S9). No crossing-hydrosilylation products **PE1-H** or **PE2-D** were observed in the mixed-silane experiments (Fig. 1F and Figs S10–S14), consistent with the *in-situ*-activated system (Fig. 1B). To further illustrate the kinetic performance of the active catalyst in the reaction, equal amounts of two different alkynes (**S4** and **S5**) were mixed with one equivalent of phenylsilane for the competitive hydrosilylation reaction. As a result, the hydrosilylation products (**PE1** and **PF1**) of the two alkynes had similar ratios when the reaction was catalyzed by the catalytic precursor **CA**/EtMgBr or by the pre-prepared active catalyst **CA1** (Fig. 1G and Figs S15–S17). These results indicate that the catalyst activated *in situ* exhibited comparable kinetics to those of the active iron complex, which fully indicates that Fe(0) was the active species (at least the main species) that initiated the reaction.

**Figure 2.**
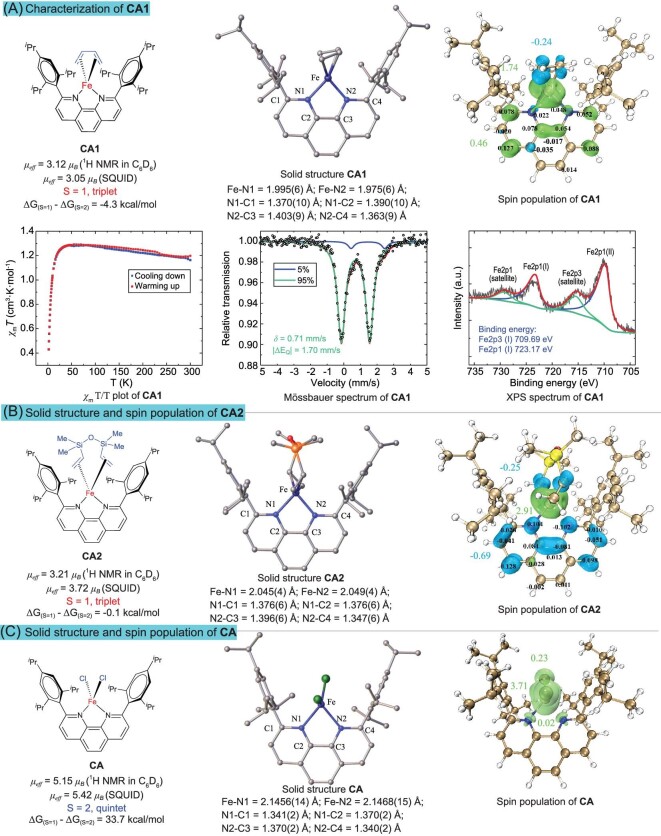
(A) Solid structure, spin populations (green, positive spin density; blue, negative spin density), magnetic property, Mössbauer and XPS spectra of CA1. (B) Solid structure and spin populations of CA2. (C) Solid structure and spin populations of CA.

We were fortunate to obtain single crystals of the Fe(0) complexes **CA1** (Fig. 2A), **CA2** (Fig. 2B), and **CC1** (Fig. S25), and we determined their chemical structures by single-crystal X-ray diffraction. Comparing the crystal structures of the two Fe(0) complexes **CA1** and **CA2** with the Fe(II) precursor **CA** (Fig. 2C), we found that the Fe−N bond lengths of the Fe(0) complexes were significantly shorter than those of the corresponding Fe(II) complexes. Relative to Fe(II) **CA** (2.1462(15)  Å, on average), the average Fe−N bond lengths of **CA1** (1.985(6)  Å, on average) and **CA2** (2.047(4)  Å, on average) were shorter by 7.5% and 4.6%, respectively. The difference in bond lengths may be attributed to two factors. On the one hand, spin multiplicity might affect the molecular electronic structure, thus leading to a difference in bond lengths. Compared with the triplet complexes, the occupied ligand-directed (anti-bonding) d-orbitals in the quintet complex **CA** weakened and elongated the M–L bond [38]. On the other hand, the degrees of spin-delocalization in the complexes caused differences in bond lengths. By examining the spin populations of these complexes, it was observed that large amounts of spin-delocalization occurred in both **CA1** and **CA2**. Namely, a large amount of spin delocalized from the iron center to the 1,10-phenanthroline backbone, while almost no spin-delocalization between the iron center and ligand occurred in **CA**. Spin-delocalization enhanced the metal–ligand interactions and thus shortened the Fe−N bond lengths of **CA1** and **CA2** [39,40]. The C−N bond length of the 1,10-phenanthroline can also reflect the spin delocalization condition. The transfer of an electron from the metal to the ligand makes the ligand appear in a ‘reduced’ state, so the C−N bond length of **CA1** and **CA2** increases significantly compared with **CA** (Fig. 2) [41]. The above laws of Fe−N bond length, C−N bond length and spin delocalization are also applicable to **CC1** and **CC** (Tables S3–S7).

In addition, although both **CA1** and **CA2** were triplet (S = 1) Fe(0) complexes, there was a significant difference in their Fe−N bond lengths, which we believe was due to the difference in their spin populations. The analysis of the spin populations of these two complexes revealed that both had significant spin-delocalization from the iron center to the 1,10-phenanthroline ligand backbone. The difference was that the spin on the 1,10-phenanthroline ligand in **CA1** (Fig. 2A) was in the same direction as the spin on the iron center while the spin on the 1,10-phenanthroline in **CA2** (Fig. 2B) was in the opposite direction as the spin on the iron center, which led to a decrease and increase in the spin on the iron, respectively. For **CA1**, the net spin on the iron was 1.74, and for **CA2**, the net spin on the iron was 2.91. As mentioned above, the higher spin density on the iron center weakened the iron–ligand bonds, which is manifested by an increase in the Fe−N bond length. Frontier orbital analyses (Table S12) suggest that there are covalent interactions between iron and 1,10-phenanthroline ligands in **CA1** and **CA2** due to spin delocalization. The varying strengths of the covalent interactions cause the differences in C−N and Fe−N bond lengths in **CA1** and **CA2**. The above analyses clearly showed that the spin-delocalization function of 1,10-phenanthroline played a crucial role in the regulation of the spin state of the iron atom. Taking **CA1** as an example, further characterization was carried out to understand the electronic structure of Fe-phenanthroline complexes (Fig. 2A. See Figs S18–S25 and Tables S1–S2 for supplementary analytical data). The magnetic susceptibility at higher temperatures suggests a S = 1 complex, possibly with significant spin orbit coupling. The low-temperature drop of the *χ*_m_T/T curve was likely a result of zero-field splitting (D). Large D may be responsible for the EPR silent fact of all the three active complexes (Fig. 2A) [42]. Fitting to the Mössbauer spectrum gives parameters *δ* = 0.71 and $\vert $ΔE_Q_$\vert $ = 1.70 mm/s with 5% impurity. Combined with spin population, this was identified as a S_(Fe)_ = 1/2 Fe (I) ferromagnetically coupled to the ligand radical anion. The Mössbauer spectrum parameters are similar to a reported *β*-diketiminate-Fe complex [43]. Calculated Mössbauer parameters (*δ* = 0.60 and $\vert $ΔE_Q_$\vert $ = 1.97 mm/s, Table S1) using Holland's calibration [44] were in reasonable error with experimental data. Careful measurement of the XPS spectrum afforded 709.69 eV Fe2p3 binding energy, slightly lower than that of FeCl_2_ (710.40 eV). Combined with XRD structure, Mössbauer spectrum, magnetic property measurement and DFT calculations, this was best described as an Fe(I) species. It is clear that spin-delocalization between the iron and 1,10-phenanthroline is responsible for the elevated oxidation state of the formal Fe(0) species.

In summary, the above structural analyses indicated that the 1,10-phenanthroline ligand backbone had a good spin-delocalization function, which efficiently regulated both the spin state and oxidation state of the iron atom. This might be the electronic structural basis for the pronounced spin effect of the iron-catalyzed alkyne hydrosilylation reaction.

### Spin effect on catalytic activity

**Figure 3.**
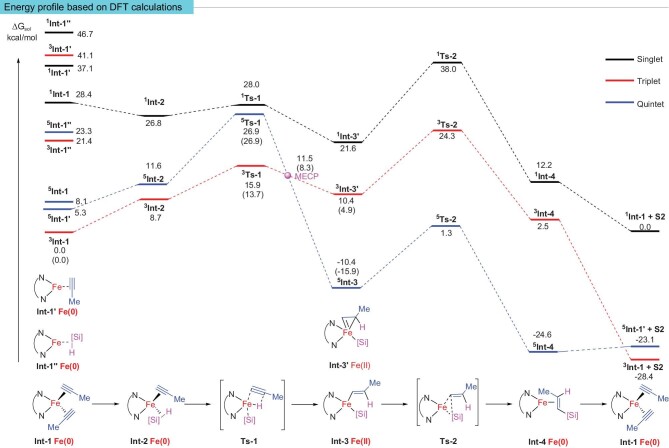
Energy profiles based on DFT calculations. Numbers in parentheses denote electronic energies of the structure. DFT calculations were performed at ωB97XD/def2TZVPP-CPCM (THF) || ωB97XD/6–31G*-TZVP (Fe) level. Energies were reported in kcal/mol.

We performed DFT calculations for a further understanding of the catalytic behaviors of the open-shell iron catalyst in alkyne hydrosilylation (Fig. 3, Figs S26–S29 and Tables S8–S17). Since the reactions affording *α*-selectivity and *β*-selectivity had similar mechanisms, we only discussed the *β*-selective reaction in the main text and included the calculation data of *α*-selective reaction in the Supplementary information.

DFT calculations showed that the singlet potential energy surface is always at the highest position throughout the reaction, so the reaction is more likely to proceed on the triplet and quintet potential energy surfaces. It is likely that the catalyst precursors coordinate mainly with two alkynes after being reduced to Fe(0). In the subsequent oxidative addition step (**Int-2**–**Ts-1**–**Int-3**), the ∆*G*^≠^ values of the triplet and quintet transition state **Ts-1** relative to **^3^Int-1** are 15.9 and 26.9 kcal/mol, respectively. In the oxidative addition process, both the intermediate and transition state in the triplet are more energetically favorable than those in the quintet. In the reductive elimination process, the situation is totally reversed, as **^5^Int-3** and **^5^Ts-2** are much lower in energy than **^3^Int-3'** and **^3^Ts-2**, respectively. As a result, the reductive elimination process takes place on the quintet potential energy surface to afford **^5^Int-4**. Based on the above analysis, this reaction has a typical two-state reactivity. Overall, the spin crossover results in a decrease of the reaction energy barrier by 8.4 kcal/mol (from 24.3 to 15.9 kcal/mol), which greatly accelerates the reaction rate. We located a minimum energy crossing point (MECP) [45,46] between the triplet and quintet, which lies between **^3^Int-3′** (or**^3^Ts-1**) and **^5^Int-3** along the reaction pathway. Since the MECP is not a stationary point on the potential energy surface, normal frequency analysis is not physically meaningful. Thus, we calculated the projected frequency [47] in the direction of the reaction pathway to estimate the Gibbs free energy correction and then estimated the relative Gibbs free energy of the MECP (11.5 kcal/mol). Since MECP is highly similar to **^3^Int-3′** in structure, **^3^Int-3′** is most likely to have been formed through **^3^Ts-1**, which then undergoes a spin crossover to afford **^5^Int-3** via MECP. An alternative possible pathway is that **^5^Int-3** is formed through MECP directly from **^3^Ts-1** without forming **^3^Int-3′**. Since the difference in energy between MECP and **^3^Int-3′** is small (1.1 kcal/mol), this may indicate a fast spin-crossover rate, which is consistent with the fast experimental reaction rate (TOF 35.5 s^−1^) [30].

To further understand the mechanism that a triplet iron catalyst promotes the oxidative addition process while a quintet iron catalyst promotes the reductive elimination process, a simple but effective index, charge-of-central-metal, was set up to measure both elementary steps based on DFT calculations. In a transition metal-catalyzed reaction, the mechanism of the oxidative addition step is usually the filling of the d electrons of the metal into the anti-bond orbitals of the σ or π coordination bonds, thus weakening their bond strength and breaking their σ or π bonds to achieve oxidative addition to the metal atoms. Therefore, the higher the electron density on the central metal, the more likely oxidative addition is to occur. Reductive elimination is the reverse process of oxidative addition. The lower the electron density on the central metal, the more likely reductive elimination is to occur. Based on this, we established the ‘Central Metal Charge Analysis’ (CMCA) method to understand the spin effect on the oxidative addition and reductive elimination processes. The basic principle for the CMCA method is that a lower charge on central metal (lower oxidation state) favors oxidative addition, and a higher charge on central metal (higher oxidation state) favors reductive elimination.

**Figure 4.**
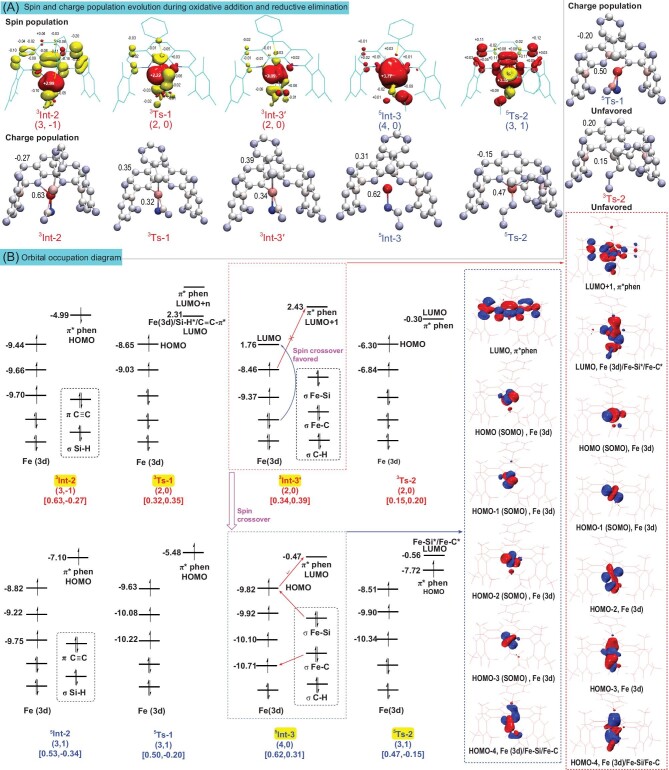
(A) Mulliken spin (red, spin up; yellow, spin down) and charge (red, positive charge; blue, negative charge; darker colour indicates larger charge) population evolution during reaction. The (m, n) labelling denotes the number of the unpaired electrons on the iron (m) and the ligand backbone (n), and the negative sign for n indicates antiferromagnetic coupling between the iron and the ligand. (B) Calculated molecular orbital occupation diagram and part of the frontier orbitals treated by wavefunction biorthogonalization. Orbital energies (eV) were evaluated for qualitative discussion. The [m, n] labelling denotes the charge on the iron (m) and the ligand backbone (n).

We first performed a charge population analysis of some key intermediates and transition states related to oxidative addition and reductive elimination (Fig. 4A). According to CMCA, the iron center of oxidative addition transition state **Ts-1** had a lower charge in the triplet state (**^3^Ts-1**, 0.32 a.u.) than in the corresponding quintet state (**^5^Ts-1**, 0.50 a.u.), and thus, the triplet **^3^Ts-1** with a higher electron density on the iron center favored the oxidative addition process. The variation of the charge on the 1,10-phenanthroline ligand backbone from **Int-2** to **Ts-1** (ligand charge variation in triplet potential energy surface, **Int-2**–**Ts-1**: −0.27 to 0.35 a.u.; quintet potential energy surface, **Int-2**–**Ts-1**: −0.34 to −0.20 a.u.) revealed the origin of the above metal charge difference. The iron center of the triplet iron catalyst apparently took a larger number of electrons from the ligand than its quintet counterpart. Thus, the triplet iron catalyst was more favorable for oxidative addition. The above phenomenon was further confirmed by the spin population analysis from **Int-2** to **Ts-1** (Fig. 4A). The spin population changed from **^3^Int-2** (3, −1) to **^3^Ts-1** (2, 0), indicating a ligand *β*-electron transfer to the metal center, resulting in the charge on Fe changing from 0.63 to 0.32 a.u., promoting oxidative addition. In contrast, there was no significant change in the catalyst charge and spin population from **^5^Int-2** to **^5^Ts-1** (Table S9) under the quintet potential energy surface. In the reductive elimination process, an *α*-electron on Fe was transferred to the ligand during the process of **^5^Int-3** (4, 0) to **^5^Ts-2** (3, 1). However, in the triplet potential energy surface, there was no significant change in the electron spin and charge population from **^3^Int-3′** to **^3^Ts-2**. As a result, the metal charge of the quintet transition state **^5^Ts-2** was 0.47 a.u., which was higher than the metal charge of the triplet transition state **^3^Ts-2** (0.15 a.u.), thus making it easier for reductive elimination to occur.

To further investigate the electron transfer process between the ligand and iron center and reveal the influence of the catalyst spin states on the reaction, we made a molecular orbital occupation diagram by wavefunction biorthogonalization [48] (Fig. 4B). As mentioned above, electron transfer from the ligand to the metal during the oxidative addition process lowered the metal oxidation state to facilitate this process. By analyzing the orbital occupation diagram, unpaired electrons were found on both ligand backbones of **^3^Int-2** and **^5^Int-2**. The difference was that the unpaired electron on the ligand of **^3^Int-2** had the opposite spin direction as the unpaired electrons on the metal, while the unpaired electrons on the ligand of **^5^Int-2** had the same spin direction as the unpaired electrons on the metal. According to the Pauli exclusion principle, there was no suitable iron singly occupied orbital or empty orbital in **^5^Int-2** that could accommodate *α*-electrons on the 1,10-phenanthroline, and the electron transfer from the ligand to the metal was more favorable in **^3^Int-2** than in **^5^Int-2**. Thus, the oxidative addition step proceeded at the triplet potential energy surface. The LUMO of **^3^Ts-1** showed strong iron 3d-orbital interacting with Si-H* antibonding orbital and C≡C* antibonding orbital (activating Si–H and C≡C), promoting the transfer of hydrogen atoms to alkyne, giving **^3^Int-3′**. Similarly, the electron transfer from the metal to the ligand during reductive elimination elevated the metal oxidation state to facilitate this process. Although the phenanthroline ligand in both **^3^Int-3′** and **^5^Int-3** had the potential to accept an electron from the iron center and thus to elevate the oxidation state of the iron center, the low-lying Fe 3d-orbital (LUMO, 1.76 eV) in **^3^Int-3′** made electron transfer difficult (the energy gap between HOMO and π* phen orbital was 10.89 eV). As a result, there was no obvious spin-delocalization in **^3^Ts-2**, and all 3d-orbitals of iron were occupied, so the oxidation state of Fe is very low (0.15 a.u.), (energy barrier for reductive elimination was 13.9 kcal/mol, Fig. 3). On the other hand, this low-lying Fe 3d-orbital provided conditions for the spin crossover from triplet to quintet. In contrast to **^3^Int-3′**, 3d-orbitals of iron in **^5^Int-3** were all occupied, and the band gap between π* phen orbital (−0.47 eV) and HOMO (−9.82 eV) is 9.35 eV, which was lower than that of **^3^Int-3′** (10.89 eV). Therefore, there was significant spin-delocalization in the subsequent **^5^Ts-2**, which made the net charge of iron in **^5^Ts-2** decrease from 0.62 to 0.47 a.u. Moreover, the LUMO of **^5^Ts-2** was mainly composed of Fe-Si* and Fe-C* antibonding orbitals, which was favorable for reduction elimination (11.7 kcal/mol, Fig. 3).

Overall, in this Fe-catalyzed alkyne hydrosilylation reaction, the 1,10-phenanthroline with a large planar conjugated structure serves as a typical redox non-innocent ligand. It acts as an electron reservoir to regulate the spin states and oxidation states of the central metal, thus adapting to the electrostatic demands of both the oxidative addition and reductive elimination processes. The ligand acts as an electron donor to lower the oxidation state of the iron atom, facilitating the oxidative addition process that occurs on the triplet potential energy surface, while acting as an electron acceptor to elevate the oxidation state of the iron atom, facilitating the reductive elimination process that occurs on the quintet potential energy surface. Most importantly, a spin crossover from the triplet state to the quintet state is necessary to help realize such an electron transfer process. It is important to point out that traditional methods such as ligand modification with some electron withdrawing/donating group can hardly accelerate the oxidative addition and reductive elimination processes simultaneously because these two processes have opposite electrostatic demand. The SDRR-enabled simultaneous acceleration of the processes with opposite electrostatic demand in iron catalysis might also be a key to understand the other spin-crossover catalysis.

### Effect of spin state on regioselectivity

**Figure 5.**
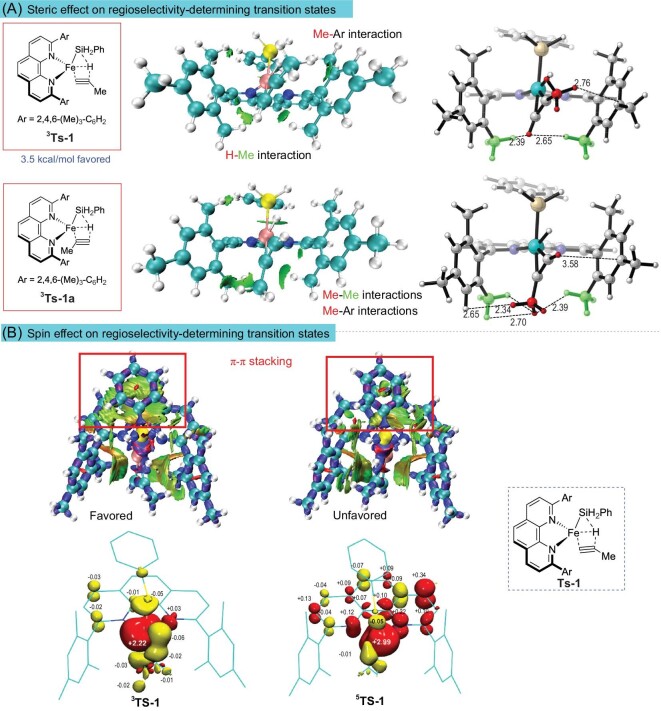
(A) Noncovalent interaction analysis of key transition states that determine regioselectivity by IGMH. IGMH analysis reveals intramolecular interactions among specific fragments of the molecule, with a focus on three fragments: 1,10-phenanthroline, the silyl group, and the alkyne. The green sheets represent intramolecular noncovalent interactions. Larger sheets indicate greater noncovalent interactions. Some representative distances between atoms and fragments are shown in units of Å. (B) Intramolecular interaction analysis by IRI and spin population of ^3^Ts-1 and ^5^Ts-1. The IRI analysis distinguishes various intramolecular interactions through different colors (Fig. S28). (See online supplementary material for a colour version of this figure.)

To further understand the origin of the regioselectivity, independent gradient model analysis based on a Hirshfeld partition (IGMH) [49] (Fig. 5A) and interaction region indicator (IRI) [50] analyses (Fig. 5B) were performed using Multiwfn [48] to analyze the noncovalent intramolecular interactions of **Ts-1**, the key transition state that determines regioselectivity.

As shown in Fig. 5A, the methyl substituents at the aryl of the 1,10-phenanthroline ligand (denoted in green) in **Ts-1** form a congested region at the bottom of the iron center (*trans* to silyl group). In **^3^Ts-1** that leads to *β*-selectivity, the small terminal hydrogen of the alkyne falls into this congested region while the big methyl group of the alkyne positions to the relatively open region of the reaction pocket. In contrast, the methyl group of the alkyne in **^3^Ts-1a** that yields *α*-selectivity falls into the congested region, resulting in significant steric repulsion. Consequently, *β*-selectivity is energetically favored by 3.5 kcal/mol over *α*-selectivity owing to a substantial reduction in the repulsive interaction (Fig. 5A).

To understand how spin states affect regioselectivity, IRI analysis (Fig. 5B and Fig. S28) was applied to investigate the intramolecular interaction of both **^3^Ts-1** and **^5^Ts-1**. It seemed clear that there was no significant difference between **^3^Ts-1** and **^5^Ts-1**, except for the distinct π–π stacking interaction on **^3^Ts-1 (**π–π stacking the 1,10-phenanthroline backbone and the phenyl group of hydrosilane), which was likely to play an important role in stabilizing the transition state. This π–π stacking interaction was inhibited in **^5^Ts-1**. This can be seen directly from the structure of **^3^Ts-1** and **^5^Ts-1**. The 1,10-phenanthroline backbone in **^5^Ts-1** was clearly not parallel to the phenyl group of the hydrosilane. We think this could be attributed to spin-delocalization. By comparing the spin populations (Fig. 5B) of **^3^Ts-1** and **^5^Ts-1**, we found that a large amount of spin on the iron in **^5^Ts-1** delocalized to the 1,10-phenanthroline backbone, leaving a large amount of negative charge on the 1,10-phenanthroline backbone. Consequently, the π–π stacking that stabilized the transition states was suppressed by electrostatic repulsion. However, in **^3^Ts-1**, there was rarely a spin-delocalization from the iron to the ligand, and the π–π stacking was not affected. The IRI analysis of the transition states affording α-selectivity (**M-^3^Ts-1** and **M-^5^Ts-1**) exhibited similar spin effect on intramolecular noncovalent interactions as those for **^3^Ts-1** and **^5^Ts-1** (Fig. S29). The above analysis concludes that, in addition to the effect of steric hindrance, the catalyst's spin state also has an impact on the realization of regioselectivity of the reaction. That is, the spin states of the catalyst can modify the intramolecular noncovalent interactions in the transition state by managing spin-delocalization, affect stability of the regioselectivity-determining transition state and thus somewhat influence regioselectivity.

## CONCLUSION

In conclusion, we have systematically investigated the mechanism of the iron-catalyzed hydrosilylation of alkynes and found that iron catalysts could promote oxidative addition and reductive elimination processes and enhance transition state stability through spin-delocalization. We synthesized and characterized the electronic structure of the well-defined formal Fe(0)-phenanthroline complexes to reveal the unique electronic structure of the catalysts. We developed the ‘Central Metal Charge Analysis’ method as an effective index to help understand the spin effect in elementary steps of the reaction, which revealed that the redox non-innocent 1,10-phenanthroline acted as an electron donor and acceptor that regulated the oxidation state of the iron center by spin-delocalization to meet the opposite electrostatic requirements of oxidative addition and reductive elimination, respectively, thus facilitating the reaction. The spin crossover of the iron catalyst was the key to facilitating the above electron transfer. The precise regulation of the regioselectivity relied on the unique active cavity formed near the iron center by the ligand's steric effect and was enhanced by stabilization of the transition state by specific spin state. These unique spin effects were designed as ‘Spin-delocalization Regulated Reactivity’ (SDRR). The above findings have important implications for understanding the mechanisms of iron-catalyzed reactions, the spin effect of open-shell catalysts, and the development of new iron catalysts and other Earth-abundant metal catalysts.

## MATERIALS AND METHODS

All air- and moisture-sensitive manipulations were carried out using standard Schlenk, high-vacuum and glovebox techniques unless described otherwise. ^1^H and ^13^C NMR spectra were recorded with a Bruker AV 400 spectrometer at 400 (^1^H NMR) and 101 (^13^C NMR) MHz, respectively. Chemical shifts were reported in ppm relative to internal Me_4_Si (^1^H NMR), CDCl_3_ (^13^C NMR) or C_6_D_6_ (^1^H NMR). High-resolution mass spectrometric analyses were determined on an IonSpec FT-ICR mass spectrometer. Single crystals suitable for X-ray diffraction were measured by a Rigaku MSC single-crystal diffractometer (Rigaku 007 Saturn 70) equipped with a molybdenum X-ray tube (λ = 0.71073 Å). The structures were solved using Olex2. Magnetic moment was measured by Evans method or SQUID VSM (Quantum Design). XPS measurements were performed with a Thermo SCIENTIFIC ESCALAB 250Xi instrument, using a mono-chromated Al Kα source (1486.68 eV). XPS data were analyzed using Thermo Avantage software. All spectra were calibrated with hydrocarbon C 1s photoemission set to a 284.8 eV binding energy. Zero-field ^57^Fe Mössbauer spectra were collected on solid powder samples maintained at 77 K. The isomer shift is referred to α-iron at 295 K and spectra were fit using MӧssWinn spectral analysis software. Mössbauer parameter calculations were performed in ORCA.

All calculations (except for Mössbauer parameter calculations) were fully calculated with the density functional theory in Gaussian 09. Calculated structures were visualized with CYLview. The MECP was located by the sobMECP program. Mulliken population, IGMH and IRI analyses were carried out using Multiwfn and visualized by Multiwfn or VMD. Geometry optimization and frequency analysis were performed in the gas phase using ωB97XD functional and a mixed basis set of TZVP for Fe and 6–31G(d) for H, C, N and Si. Single point energy calculations were carried out with the same functional for optimization using a larger basis set of def2-TZVPP and the CPCM solvation model with THF as the solvent.

## Supplementary Material

nwad324_Supplemental_File
