# Supplementary material for: Spin effect on redox acceleration and regioselectivity in Fe-catalyzed alkyne hydrosilylation
Source: Natl Sci Rev. 2023 Dec 20;11(2):nwad324. doi: 10.1093/nsr/nwad324 (PMC10837105; doi:10.1093/nsr/nwad324)
Supplement: nwad324_Supplemental_File [file nwad324_supplemental_file.docx]

Supplementary Information for

**Spin effect on redox acceleration and regioselectivity in Fe-catalyzed alkyne hydrosilylation**

*Peng He^†^, Meng-Yang Hu^†^, Jin-Hong Li^†^, Tian-Zhang Qiao^†^, Yi-Lin Lu^†^, Shou-Fei Zhu*^,†^*

*^†^ Frontiers Science Center for New Organic Matter, State Key Laboratory and Institute of Elemento-Organic Chemistry, Nankai University, Tianjin 300071, China*

**Email: sfzhu@nankai.edu.cn*

**Contents**

1. General considerations…………………………………………………..………...S2

2. Synthesis of Fe(0) complexes…………………………………………..…………S4

3. Kinetic experiments of Fe(0) complexes………………………………………..S9

4. Mössbauer spectra of Fe(0) complexes………………………………………..S19

5. XPS spectrum of **CA1**…………………………………………………..S24

6. Magnetic measurement of **CA1**…………………………..……………….S25

7. Single crystal structures of **CA**, **CA1**, **CA2**, and **CC1**………………………….S26

8. Computational details……………………………………………………………S32

9. References………………………………………………………………………S232

**1. General considerations**

All air- and moisture-sensitive manipulations were carried out using standard Schlenk, high-vacuum and glovebox techniques unless described otherwise. Magnesium, sodium, 1,3-butadiene, 1,1,3,3-tetramethyl-1,3-divinyldisiloxane (dvtms) and ferrous chloride (99.99%) were purchased from Acros, Alfa Aesar or TCI. Diethyl ether, tetrahydrofuran (THF), hexane, pentane, and hexadeuteriobenzene (C_6_D_6_) were distilled from sodium/benzophenone ketyl prior to use. Chloroform-*d* (CDCl_3_) was used as received for some NMR experiments. The dvtms was dried over calcium hydride (CaH_2_) and distilled prior to use. ^1^H and ^13^C NMR spectra were recorded with a Bruker AV 400 spectrometer at 400 (^1^H NMR) and 101 (^13^C NMR) MHz, respectively. Chemical shifts were reported in ppm relative to internal Me_4_Si (^1^H NMR), CDCl_3_ (^13^C NMR) or C_6_D_6_ (^1^H NMR). High resolution mass spectrometric (HRMS) analyses spectrum was determined on an IonSpec FT-ICR mass spectrometer. Single crystals suitable for X-ray diffraction were coated with polyisobutylene oil in an argon-protected glovebox before transferred to a Rigaku MSC single-crystal diffractometer (Rigaku 007 Saturn 70) equipped with a molybdenum X-ray tube (λ = 0.71073 Å). The structures were solved using Olex2 (version 1.5). Magnetic moment was measured by Even’s method (in solution) or SQUID VSM (Quantum Design) (solid state). XPS measurements were performed with a Thermo SCIENTIFIC ESCALAB 250Xi instrument, using a mono-chromated Al Kα source (1486.68 eV). A pass energy (PE) of 100 eV and step of 1 eV was used to acquire wide-range survey spectra. A pass energy of 30 eV was used to acquire narrow spectra of the C 1s, O 1s, Li 1s, F 1s, N 1s, P 2p, and Fe 2p levels. XPS data were analyzed using the Thermo Avantage software (version 5.99) and corresponding figures were plotted using Origin (version 2018). All spectra were calibrated with hydrocarbon C 1s photoemission set to 284.8 eV binding energy. Zero-field ^57^Fe Mössbauer spectra were collected on solid powder samples maintained at 77 K. The 𝛿, |ΔE_Q_| and Γ represents isomer shift (IS), quadrupole splitting (QS) and line width. The isomer shift is referred to α-iron at 295 K and spectra were fit using MӧssWinn spectral analysis software. Mössbauer parameter calculations were performed in ORCA[1-3], following the calibration established by Neese [4] or Holland. [5]

All calculation (except for Mössbauer parameter calculations) were fully calculated with the density functional theory level in Gaussian 09 [6]. Calculated structures were visualized with CYLview. [7] The minimum energy crossing point (MECP) was located by the sobMECP program which was modified by Lu [8] based on code developed by Harvey et al. [9] Mulliken spin population and Mulliken charge distribution plots were obtained from a Mulliken population analysis by software Multiwfn[10] and visualized by VMD[11] program. IGMH[12] and IRI[13] analysis for intramolecular interactions were carried out using Multiwfn. All structure Cartesian coordinates can be found in **Table S16**. Geometry optimization and frequency analysis were performed in gas phase using ωB97XD functional (restricted or unrestricted) [14] and a mixed basis set of TZVP [15, 16] for Fe and 6-31G(d) for H, C, N and Si. Intrinsic reaction coordinate (IRC) [17, 18] calculations were conducted to determine the connectivity of minima and transition states. Single point energy calculations were carried out with the same functional in optimization using a larger basis set of def2-TZVPP [18, 19] and the CPCM solvation model [20, 21] with THF as the solvent (United Atom Topological Model (UAHF) defined the solute cavity).

**2. Synthesis of Fe(0) complexes.**

**Figure S1** Fe(0) complexes synthesized by modified references’ **method A**[22] and **method B**[23]

**CA** (5.27 *μ*_B_ by Evans’ method) and **CC** (5.15 *μ*_B_ by Evans’ method; 5.42 *μ*_B_ by SQUID) were synthesized according to our previous report[25]. Magnetic moments were detected by Evans’ method or SQUID.

**Preparation of CA1 through method B:** In an argon-protected glove box, to a vial (25 mL) containing a stir bar was added **CA** (0.463 g, 0.65 mmol) and diethyl ether (15 mL, not completely dissolved). The mixture was stirred in an oil bath at −40 ℃ for 15 min, followed by the addition of Mg(C_4_H_6_)(THF)_2_ [24] (0.304 g, 1.37 mmol). The vial was sealed and stirred in an oil bath at −40 ℃ for 1 h and at room temperature for another 3 h. The color of the mixture rapidly deepened and finally became brownish black. After 4 h of reaction, two-thirds of the solvent was removed under vacuum, 15 mL of hexane was added and stirred to dissolve. It was filtered through a sand core funnel (5-10 μm pore size, covered by about 0.4 cm thick celite), the filter residue was washed with ether. The filtrate was desolvated under vacuum and dissolved with hexane, then filtered three times using a 0.45 μm pore size filter membrane, and vacuum desolvated to obtain 0.432 g of brown-black solid powder (96% yield). The powder was further purified by crystallization using hexane mixed with ether under −37 ℃. The crystals crystallized by the above method could be used for X-ray single crystal diffraction, and the structure of the product was confirmed to be **CA1**. Anal. Calc.: C, 79.52; H, 8.41; N, 4.03, Found: C, 74.76; H, 7.93; N, 3.85. Encapsulated Et_2_O might be responsible for the difference. The magnetic moment in deuterated benzene was measured as 3.12 *μ_B_* using Even's method and 3.05 *μ_B_* using SQUID, both corresponding to the triplet state; DFT calculations showed that the energy of the triplet **CA1** was 4.3 kcal/mol lower than that of the quintet **CA1**, indicating a triplet ground state, which was consistent with the conclusion derived from experimentally measured magnetic moment. The reasons for the observed larger magnetic moments compared to the theoretical spin moment (2.89 *μ_B_*) might attribute to: 1) it contains the contribution of unquenched orbital magnetic moments; 2) there may be a small distribution of higher spin states such as the quintet state in addition to the triplet state. ^1^H NMR (400 MHz, C_6_D_6_) δ 169.69, 161.68, 146.87, 96.81, 66.02, 18.89, 17.19, 4.68, 3.59, 2.43, 1.35, 0.28, -2.33, -5.42, -7.13. 18 peaks are expected for a rough Cs symmetric molecule in the ^1^H NMR spectrum, 15 peaks are observed.

**Figure S2** ^1^H NMR spectrum (C_6_D_6_) of **CA1**.

**Preparation of CA2 through method A:** In an argon-protected glove box, to a vial (25 mL) containing a stir bar was added **CA** (0.463 g, 0.65 mmol), naphthalene (0.0042 g, 0.0325 mmol), dvtms (450 μL, 1.95 mmol), followed by diethyl ether (15 mL, not completely dissolved) to dissolve, and finally small pieces of sodium metal (0.0374 g, 1.63 mmol; strip off the surface oxides). The color of the mixture slowly deepened and finally became deep purple-black. The vial was sealed and stirred at room temperature for 4 h before the solvent was removed under vacuum, 15 mL of hexane was added and stirred to dissolve. It was filtered through a sand core funnel (5-10 μm pore size, covered by about 0.4 cm thick celite), the filter residue was washed with ether. The filtrate was desolvated under vacuum and dissolved with hexane, then filtered three times using a 0.45 *μ*m pore size filter membrane, and vacuum desolvated to obtain 0.616 g of purple-black solid powder (>99% yield). The powder was further purified by crystallization using hexane mixed with ether under −37 ℃. Take about 10 mg of sample into a 4 mL sample bottle, dissolve it with 3 mL of hexane, mask the bottle cap, and slowly evaporate and crystallize to obtain a single crystal suitable for X-ray single crystal diffraction, and the structure of the product was confirmed to be **CA2**. Anal. Calc.: C, 72.78; H, 8.31; N, 3.40, Found: C, 70.98; H, 8.53; N, 3.17. The magnetic moment in deuterated benzene was measured as 3.21 *μ_B_* using Even's method and 3.72 *μ_B_* using SQUID, both corresponding to the triplet state; DFT calculations showed that the energy of the triplet **CA2** was 0.1 ‒ 6.4 kcal/mol lower than that of the quintet **CA2**, indicating a triplet ground state, which was consistent with the conclusion derived from experimentally measured magnetic moment. The reasons for the observed larger magnetic moments compared to the theoretical spin moment (2.89 *μ_B_*) might attribute to: 1) it contains the contribution of unquenched orbital magnetic moments; 2) there may be a small distribution of higher spin states such as the quintet state in addition to the triplet state. ^1^H NMR (400 MHz, C_6_D_6_) δ 2.87, 1.36, -0.55, -1.43. 12 peaks are expected for a rough C2v symmetric molecule in the ^1^H NMR spectrum, only 4 broad peaks are observed.

**Figure S3** ^1^H NMR spectrum (C_6_D_6_) of **CA2**.

**Preparation of CC1 through method B:** In an argon-protected glove box, to a vial (25 mL) containing a stir bar was added **CC** (0.272 g, 0.5 mmol), diethyl ether (15 mL, not completely dissolved). The mixture was stirred in an oil bath at −40 ℃ for 15 min, followed by the addition of Mg(C_4_H_6_) (THF)_2_ (0.234 g, 1.05 mmol). The vial was sealed and stirred in an oil bath at −40 ℃ for 1 h and at room temperature for another 3 h. The color of the mixture rapidly deepened and finally appeared brownish black. After 4 h of reaction, the mixture was filtered through a sand core funnel (5-10 *μ*m pore size, covered by about 0.4 cm thick celite), the filter residue was washed with ether. The filtrate was desolvated under vacuum and dissolved with hexane, then filtered three times using a 0.45 *μ*m pore size filter membrane, and vacuum desolvated to obtain 0.184 g of brown-black solid powder (70% yield). The powder was further purified by crystallization using hexane mixed with ether under −37 ℃. The crystals crystallized by the above method could be used for X-ray single crystal diffraction, and the structure of the product was confirmed to be **CC1**. Anal. Calc.: C, 77.56; H, 6.51; N, 5.32, Found: C, 71.31; H, 5.92; N, 5.18. Small portion of dissociated free ligand might be responsible for the difference. The magnetic moment in deuterated benzene was measured as 3.28 *μ_B_* using Even's method and 3.90 *μ_B_* using SQUID, both corresponding to the triplet state; DFT calculations showed that the energy of the triplet **CC1** was 3.9 kcal/mol lower than that of the quintet **CC1**, indicating a triplet ground state, which is consistent with the conclusion derived from experimentally measured magnetic moment. The reasons for the observed larger magnetic moments compared to the theoretical spin moment (2.89 *μ_B_*) might attribute to: 1) it contains the contribution of unquenched orbital magnetic moments; 2) there may be a small distribution of higher spin states such as the quintet state in addition to the triplet state. ^1^H NMR (400 MHz, C_6_D_6_) δ 170.05, 164.77, 149.17, 98.26, 68.76, 19.32, 16.61, 5.43 (9), 2.85 (8), 0.60 (14), 0.41 (13), -8.49 (7), -9.96 (12). 14 peaks are expected for a rough Cs symmetric molecule in the ^1^H NMR spectrum, 13 peaks are observed.

**Figure S4** ^1^H NMR spectrum (C_6_D_6_) of **CC1**.

**3.** **Kinetic experiments of Fe(0) complexes**

**Catalytic performances of Fe(0) complexes in alkyne hydrosilylation reactions**

**Figure S5** Iron-catalyzed alkyne hydrosilylation reactions

**General procedure A**: In an argon-filled glovebox, to a vial (10 mL) containing a stir bar was added **CA** (0.0036 g, 0.005 mmol), THF (1 mL), **S3** (0.0581 g, 0.5 mmol) and PhSiH_3_ (0.0595 g, 0.55 mmol) in sequence and stirred for 2 min. Subsequently, EtMgBr (12 *μ*L, 0.012 mmol, 1.0 mol/L in THF) was added and the vial was sealed. After vigorous stirring for 10 min at room temperature, the vial was removed from the glovebox and the reaction mixture was concentrated by rotary evaporation. **PD1** and **PD1'** (colorless oil, 0.0956 g, 86% yield, 95:5 r.r.) [25] were obtained by flash column chromatography with hexane as eluent. ^1^HNMR was used to determine regioselectivity.

**General procedure B**: In an argon-filled glovebox, to a vial (10 mL) containing a stir bar was added THF (1 mL), **S3** ( 0.0581 g, 0.5 mmol) and PhSiH_3_ (0.0595 g, 0.55 mmol) in sequence and stirred for 2 min. Subsequently, **CA1** (0.0035 g, 0.005 mmol) was added and the vial was sealed. After vigorous stirring for 10 min at room temperature, the vial was removed from the glovebox and the reaction mixture was concentrated by rotary evaporation. **PD1** and **PD1'** (colorless oil, 0.1039 g, 93% yield, 95:5 r.r.) were obtained by flash column chromatography with hexane as eluent. ^1^HNMR was used to determine regioselectivity. ^1^H NMR (400 MHz, CDCl_3_) δ 7.64-7.62 (m, 2H), 7.42-7.31 (m, 7H), 7.25-7.22 (m, 1H), 7.01 (q, *J*=2.0 Hz, 1H), 4.66 (s, 2H), 2.03 (d, *J*=1.6 Hz, 3H).

**Figure S6** ^1^H NMR spectrum (CDCl_3_) of alkyne hydrosilylation products obtained with **CA1**.

Following **general procedure B**, when **CA2** (0.0041 g) was applied instead of **CA1**, 0.1050 g **PD1** and **PD1'** (94%, 95:5 r.r.) were obtained.

**Figure S7** ^1^H NMR spectrum (CDCl_3_) of alkyne hydrosilylation products obtained with **CA2**.

Following **general procedure A**, when **CC** (0.0027 g) was applied instead of **CA**, 0.1021 g **PD1** and **PD1'** (91%, 40:60 r.r.) were obtained.

**Figure S8** ^1^H NMR spectrum (CDCl_3_) of alkyne hydrosilylation products obtained with **CC**.

Following **general procedure B**, when **CC1** (0.0026 g) was applied instead of **CA1**, 0.1044 g **PD1** and **PD1'** (93%, 40:60 r.r.) were obtained.

**Figure S9** ^1^H NMR spectrum (CDCl_3_) of alkyne hydrosilylation products obtained with **CC1**.

**Mixed silane experiment**

**Figure S10** Mixed silane experiment using **CA1** as the catalyst.

**Procedure**: in an argon-filled glovebox, to a vial (10 mL) containing a stir bar was added THF (1 mL), **S4** ( 0.0721 g, 0.5 mmol) and PhSiD_3_ (0.0556 g, 0.5 mmol) and C_8_H_17_SiH_3_ (0.0722 g, 0.5 mmol) and stirred for 5 min. Subsequently, **CA1** (0.0069 g, 0.01 mmol) was added and the vial was sealed. After vigorous stirring for 5 min at room temperature, the vial was removed from the glovebox and the reaction mixture was concentrated by rotary evaporation. **PE1-D** (colorless oil, 0.086 g, 67% yield) and **PE2** (colorless oil, 0.035 g, 24% yield) were obtained by column chromatography with hexane as eluent. **PE1-H** or **PE2-D** was not detected. The deuterated ratio of the products was determined by ^1^H NMR spectra. Slight duteration detected on **PE2** could be attributed to rapid H/D exchange between PhSiD_3_ and C_8_H_17_SiH_3_ as C_8_H_17_SiH_3_ reacted slower with **S4** than PhSiD_3_.

Analytical data of **PE1-D**: ^1^H NMR (400 MHz, CDCl_3_) δ 7.60 – 7.52 (m, 2H), 7.43 – 7.32 (m, 3H), 7.31 – 7.23 (m, 2H), 7.17 (m, 3H), 5.73 (s, 1H), 2.63 (t, *J* = 8.0 0Hz, 2H), 2.22 (t, *J* = 7.5 Hz, 2H), 1.76 (p, *J* = 7.6 Hz, 2H). ^13^C NMR (101 MHz, CDCl_3_) δ 153.05 (t, *J* = 24.0 Hz, 1C), 142.23 (s, 1C), 135.33 (s, 2C), 132.19 (s, 1C), 129.59 (s, 1C), 128.43 (s, 2C), 128.29 (s, 2C), 127.98 (s, 2C), 125.74 (s, 1C), 124.68 (s, 1C), 120.29 (s, 1C), 36.83 (s, 1C), 35.31 (s, 1C), 30.05 (s, 1C). HRMS (EI) calcd for[M, C_17_H_17_D_3_Si]^+^: 255.15226, found 255.15193.

Analytical data of **PE2**: ^1^H NMR (400 MHz, CDCl_3_) δ 7.27 (m, 2H), 7.22 – 7.13 (m, 3H), 6.26 (dt, *J* = 18.4, 6.3 Hz, 1H), 5.59 (d, *J* = 18.4 Hz, 1H), 3.89 (q, *J* = 3.5 Hz, 2H), 2.62 (t, *J* = 7.7 Hz, 2H), 2.18 (q, *J* = 7.0 Hz, 2H), 1.74 (p, *J* = 7.5 Hz, 2H), 1.46 – 1.18 (m, 12H), 0.87 (t, *J* = 6.8 Hz, 3H), 0.72 (tt, *J* = 7.3, 3.6 Hz, 2H). ^13^C NMR (101 MHz, CDCl_3_) δ 151.53 (s, 1C), 142.32 (s, 1C), 128.43(s, 2C), 128.26 (s, 2C), 125.70 (s, 1C), 121.56 (s, 1C), 36.29 (s, 1C), 35.29 (s, 1C), 32.82 (s, 1C), 31.90 (s, 1C), 30.16 (s, 1C), 29.28 (s, 1C), 29.21 (s, 1C), 25.17 (s, 1C), 22.67 (s, 1C), 14.12 (s, 1C), 9.80 (s, 1C). HRMS (EI) calcd for[M, C_19_H_32_Si]^+^: 288.22733, found 288.22687.

**Figure S11** ^1^H NMR spectrum (CDCl_3_) of **PE1-D** mixed with little **PE1**.

**Figure S12** ^13^C NMR spectrum (CDCl_3_) of **PE1-D**.

**Figure S13** ^1^H NMR spectrum (CDCl_3_) of **PE2**.

**Figure S14** ^13^C NMR spectrum (CDCl_3_) of **PE2**.

**Mixed alkyne experiments**

**Figure S15** Mixed alkyne experiments using **CA1** (**A**) or **CA**/EtMgBr (**B**).

**Procedure of reaction A:** In an argon-filled glovebox, to a vial (10 mL) containing a stir bar was added THF (1 mL), **S4** (0.0721 g, 0.5 mmol), **S5** ( 0.0331 g, 0.5 mmol) and PhSiH_3_ (0.0541 g, 0.5 mmol) and stirred for 5 min. Subsequently, **CA1** (0.0069 g, 0.01 mmol) was added and the vial was sealed. After vigorous stirring for 5 min at room temperature, the vial was removed from the glovebox and quenched by air. 1,3,5-Trimethoxybenzene (0.0336 g, 0.2 mmol) was added to the mixture as internal indicator. After further stirring for 5 min, the crude mixture was sampled in a NMR tube for ^1^H NMR experiment. Based on NMR analysis, 34% **PE1** and 59% **PF1** were produced.

**Figure S16** Crude ^1^H NMR spectrum in CDCl_3_ of reaction **A** with 1,3,5-trimethoxybenzene as internal indicator. Integral area of H^5^ was assumed to be 1.2.

**Procedure of reaction B:** In an argon-filled glovebox, to a vial (10 mL) containing a stir bar was added **CA** (0.0070 g, 0.01 mmol), THF (1 mL), **S4** ( 0.0721 g, 0.5 mmol), **S5** ( 0.0331 g, 0.5 mmol) and PhSiH_3_ (0.0541 g, 0.5 mmol). Subsequently, EtMgBr (24 μL, 1.0 mol/L in THF, 0.024 mmol) was added after stirring for 5 min. The vial was sealed. After vigorous stirring for 5 min at room temperature, the vial was removed from the glovebox and quenched by air. 1,3,5-Trimethoxybenzene (0.0336 g, 0.2 mmol) was added to the mixture as internal indicator. After further stirring for 5 min, the crude mixture was sampled in a NMR tube for ^1^H NMR experiment. As a result, 35% **PE1** and 52% **PF1** were produced.

**Figure S17** Crude ^1^H NMR spectrum in CDCl_3_ of reaction **B** with 1,3,5-trimethoxybenzene as internal indicator. Integral area of H^5^ was assumed to be 1.2.

**4. Mössbauer spectra of Fe(0)complexes**


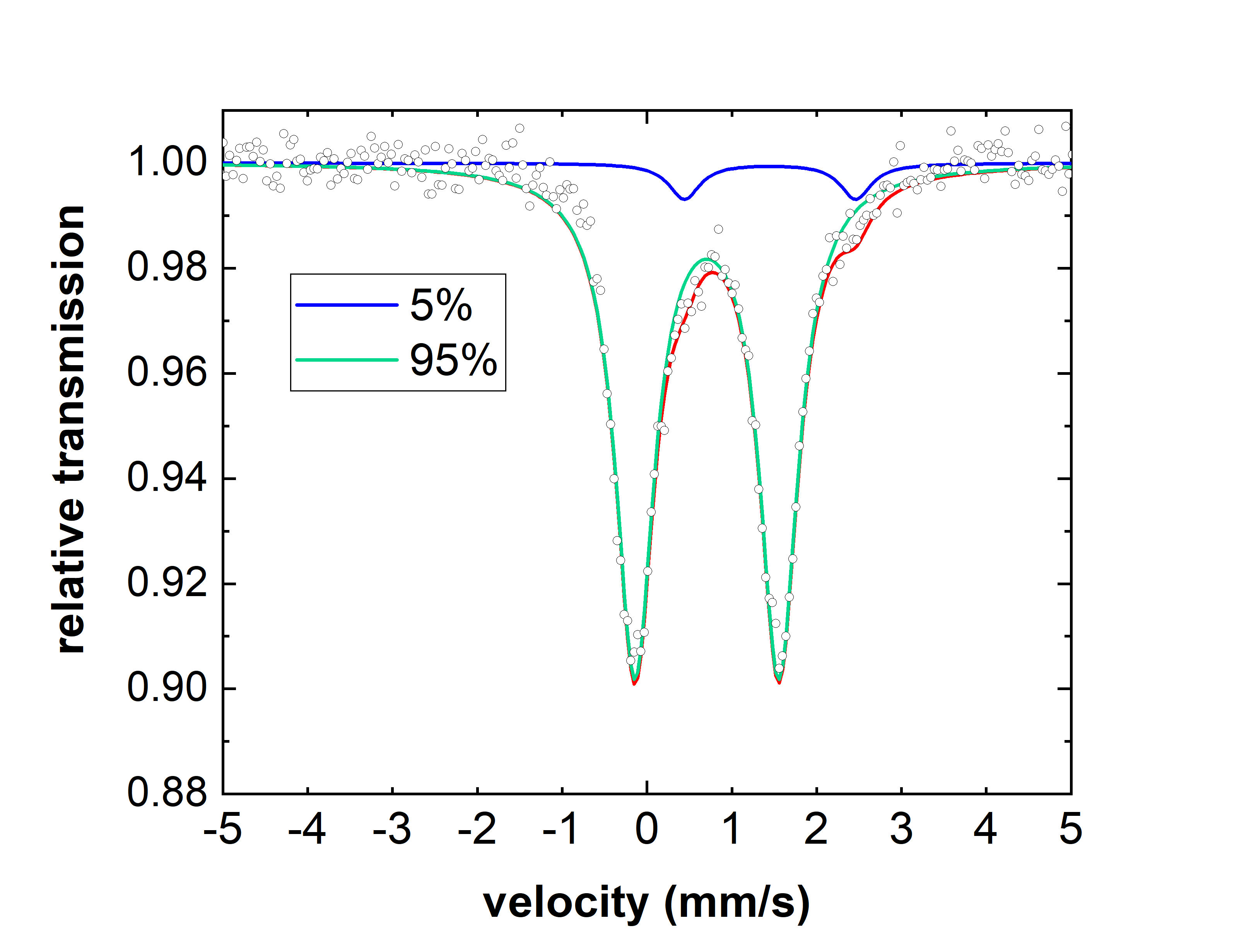


**Figure S18** Mössbauer spectrum of **CA1**. Mössbauer parameters for **CA1** (95%) are 𝛿= 0.71 mm/s, |ΔE_Q_| = 1.70 mm/s and Γ = 0.55 mm/s. Mössbauer parameters for one unknown impurity (5%) are 𝛿= 1.45 mm/s, |ΔE_Q_| = 2.02 mm/s and Γ = 0.45 mm/s.


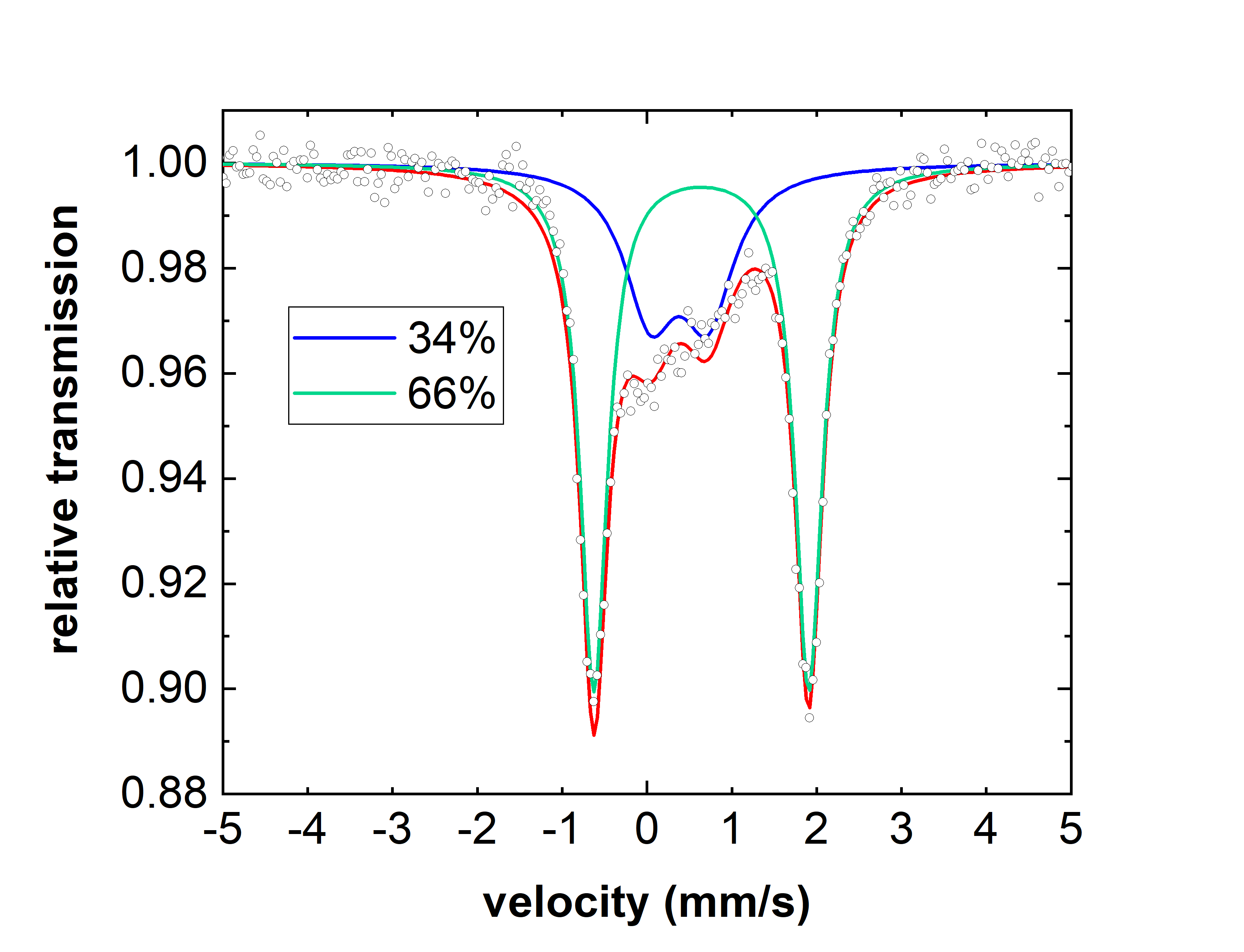


**Figure S19** Mössbauer spectrum of **CA2**. Mössbauer parameters for **CA1** (66%) are 𝛿= 0.64 mm/s, |ΔE_Q_| = 2.53 mm/s and Γ = 0.39 mm/s. Mössbauer parameters for one unknown impurity (34%) are 𝛿= 0.38 mm/s, |ΔE_Q_| = 0.68 mm/s and Γ = 0.74 mm/s. We speculate that this impurity may be an isomer of **CA2** (e.g., a monoene coordinated isomer, similar iron complex were isolated in our lab).

**CA2** is best described as a S = 1 complex, with S_(Fe)_ = 3/2 Fe (I) antiferromagnetically coupled to the ligand radical anion, combining the Mössbauer parameters and DFT calculations.

**Table S1** Experimental and calculated Mössbauer parameters.

|  | **Exp.** | | **Cal.**  **Holland’s calibration** | | | **Cal.**  **Neese’s calibration** | | |
| --- | --- | --- | --- | --- | --- | --- | --- | --- |
|  | 𝛿 (mm/s) | \|ΔE_Q_\| (mm/s) | *ρ_0_* (a.u.^-3^) | 𝛿 (mm/s) | \|ΔE_Q_\| (mm/s) | *ρ_0_* (a.u.^-3^) | 𝛿 (mm/s) | \|ΔE_Q_\| (mm/s) |
| **CA1** | 0.71 | 1.70 | 13781.82 | 0.60 | 1.97 | 13781.62 | 0.48 | 2.01 |
| **CA2** | 0.64 | 2.53 | 13781.12 | 0.82 | 1.97 | 13781.31 | 0.58 | 1.82 |

Mössbauer parameters were calculated based on the following formula:

*δ*= *α*(*ρ_0_*-C) + *β*

*δ* is the isomer shift, *ρ_0_* is the calculated electron density at Fe, *α*, *β* and C are calibration constants.

**For Holland’s calibration (ORCA 303):**

*δ*= -0.32*(*ρ_0_*-13780) + 1.18

An example of input file for geometry optimization is:

! UKS BP86 RI ZORA def2-TZVP def2-TZVP/J noautostart miniprint nopop Grid4 NoFinalGrid

! TightSCF D3BJ COSMO(toluene) TightOpt KDIIS SOSCF

%scf MaxIter 220 end

%pal nprocs 24 end

* xyz 0 3

xyz coordinates

*

An example of input file for Mössbauer parameters calculation is:

! UKS B3LYP ZORA def2-TZVP Grid4 NoFinalGrid

! TightSCF COSMO(toluene) D3BJ KDIIS SOSCF

%Method SpecialGridAtoms 26

SpecialGridIntAcc 7 end

%scf MaxIter 220 end

%pal nprocs 20 end

* xyz 0 3

xyz coordinates

*

%eprnmr

nuclei = all Fe{fgrad,rho} end

**For Neese’s calibration (ORCA 502):**

*δ*= -0.312*(*ρ_0_*-13770) + 4.103

An example of input file for geometry optimization is:

! UKS BP86 ZORA-TZVP ZORA noautostart miniprint nopop

! TightSCF CPCMC(water) opt

%Method SpecialGridAtoms 26

SpecialGridIntAcc 7 end

%scf MaxIter 320 end

%pal nprocs 20 end

* xyz 0 3

xyz coordinates

*

An example of input file for Mössbauer parameters calculation is:

! UKS B3LYP ZORA ZORA-TZVP noautostart miniprint nopop

! TightSCF CPCMC(water) KDIIS SOSCF

%Method SpecialGridAtoms 26

SpecialGridIntAcc 7 end

%scf MaxIter 320 end

%pal nprocs 20 end

* xyz 0 3

xyz coordinates

*

%eprnmr

nuclei = all Fe{fgrad,rho} end

**5. XPS spectrum of CA1**


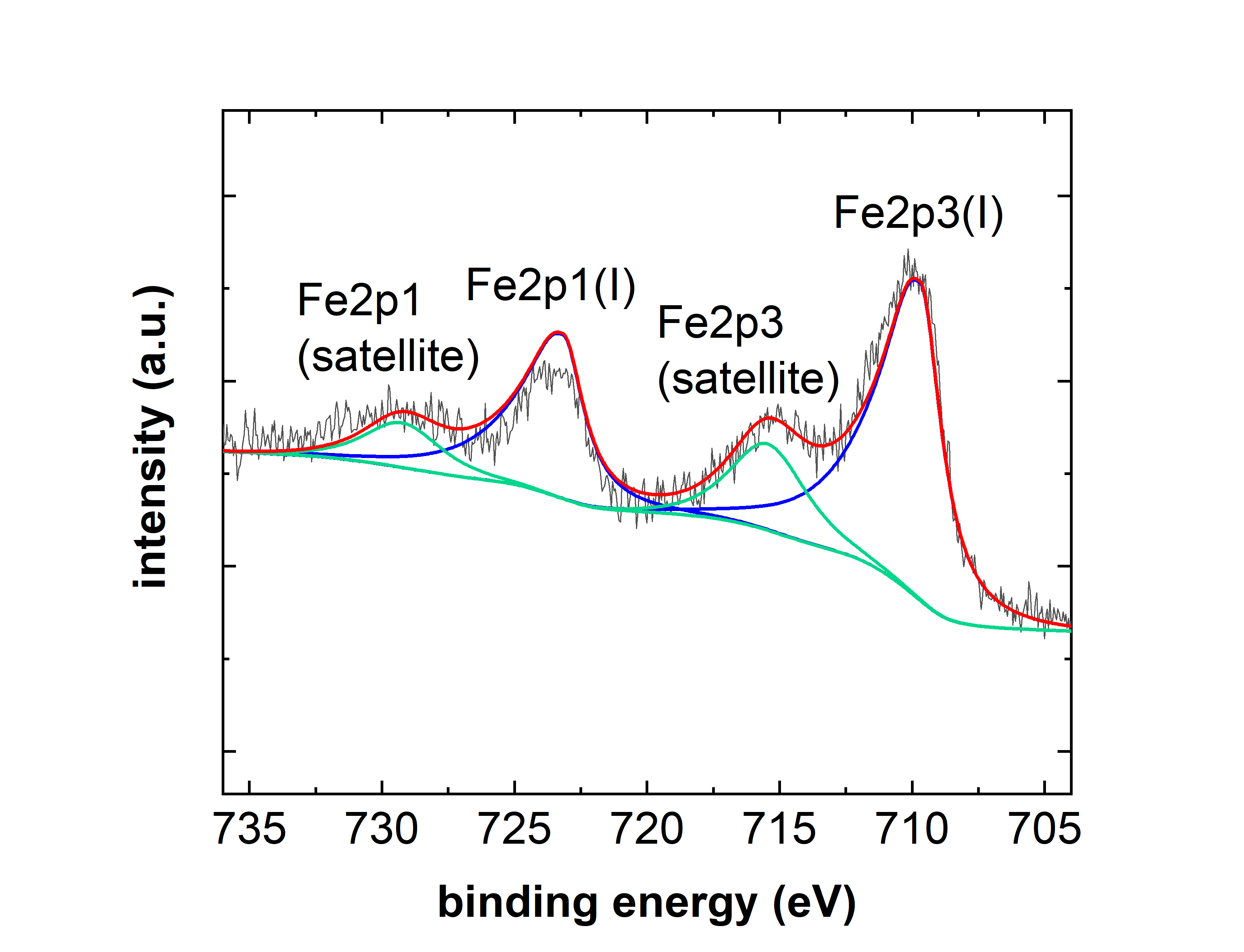


**Figure S20** Fe 2p XPS spectrum of **CA1** with overlapping peak resolution.

**Table S2**. Fitting parameters of peak resolution for Fe 2p XPS spectrum of **CA1**.

| Peaks | Binding energy (eV) | FWHM (eV) |
| --- | --- | --- |
| Fe2p3(I) | 709.69 | 1.91 |
| Fe2p1(I) | 723.17 | 1.91 |
| Fe2p3 (satellite) | 715.44 | 3.5 |
| Fe2p1 (satellite) | 729.24 | 3.5 |

**6. Magnetic property measurement of CA1**


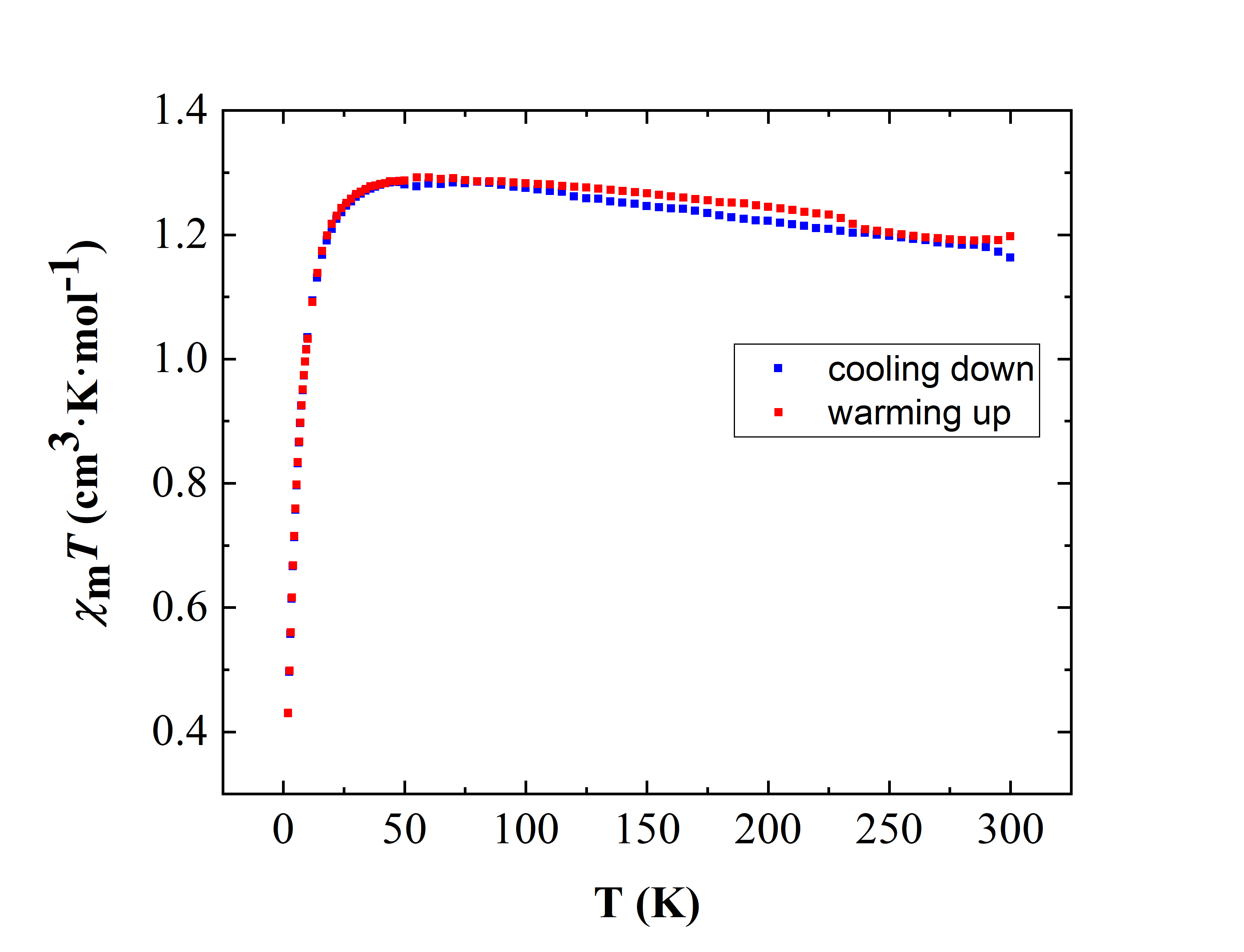


**Figure S21** *χ*_m_T/T curve of **CA1**. Temperature dependent magnetic moment was measured on SQUID at 1000 Oe magnetic field, and temperature varied in the range of 2-300 K.

**7. Single crystal structures of CA, CA1, CA2, and CC1**

**Table S3** Crystal data and structure refinement for **CA.**

| Empirical formula | C_42_H_52_Cl_2_FeN_2_ |
| --- | --- |
| Formula weight | 711.60 |
| Temperature/K | 113.15 |
| Crystal system | monoclinic |
| Space group | P2_1_/n |
| a/Å | 9.0273(3) |
| b/Å | 19.5070(6) |
| c/Å | 22.0924(6) |
| α/° | 90 |
| β/° | 94.421(3) |
| γ/° | 90 |
| Volume/Å^3^ | 3878.8(2) |
| Z | 4 |
| ρ_calc_g/cm^3^ | 1.219 |
| *μ*/mm^‑1^ | 0.557 |
| F(000) | 1512.0 |
| Crystal size/mm^3^ | 0.24 × 0.22 × 0.17 |
| Radiation | MoKα (λ = 0.71073) |
| 2Θ range for data collection/° | 4.176 to 67.118 |
| Index ranges | -13 ≤ h ≤ 12, -29 ≤ k ≤ 28, -33 ≤ l ≤ 34 |
| Reflections collected | 46877 |
| Independent reflections | 14323 [R_int_ = 0.0693, R_sigma_ = 0.0771] |
| Data/restraints/parameters | 14323/109/467 |
| Goodness-of-fit on F^2^ | 1.041 |
| Final R indexes [I>=2σ (I)] | R_1_ = 0.0583, wR_2_ = 0.1151 |
| Final R indexes [all data] | R_1_ = 0.0997, wR_2_ = 0.1381 |
| Largest diff. peak/hole / e Å^-3^ | 0.49/-0.44 |

**
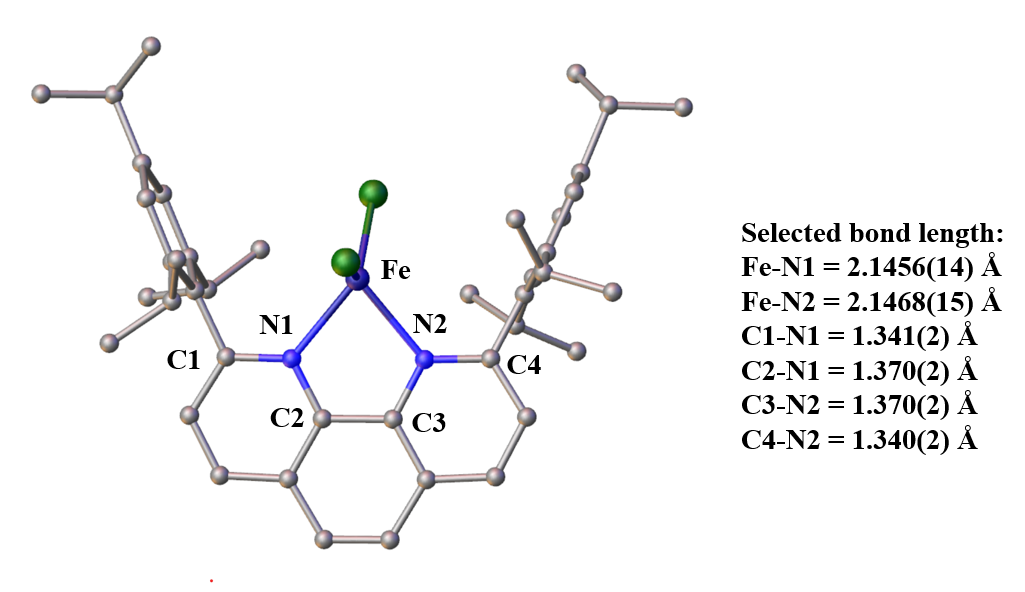
**

**Figure S22** Representation of the solid-state structure and selected bond length of **CA**. Solvents, hydrogens and part of some other structures were omitted for clarity.

**Table S4** Crystal data and structure refinement for **CA1.**

| Empirical formula | C_196_H_262_Fe_4_N_8_O_3_ |
| --- | --- |
| Formula weight | 3001.52 |
| Temperature/K | 138(1) |
| Crystal system | monoclinic |
| Space group | P2_1_/n |
| a/Å | 15.2442(6) |
| b/Å | 16.3201(5) |
| c/Å | 35.4422(14) |
| α/° | 90 |
| β/° | 98.016(4) |
| γ/° | 90 |
| Volume/Å^3^ | 8731.4(6) |
| Z | 2 |
| ρ_calc_g/cm^3^ | 1.142 |
| *μ*/mm^‑1^ | 0.381 |
| F(000) | 3244.0 |
| Crystal size/mm^3^ | ? × ? × ? |
| Radiation | Mo Kα (λ = 0.71073) |
| 2Θ range for data collection/° | 6.5 to 58.584 |
| Index ranges | -19 ≤ h ≤ 18, -20 ≤ k ≤ 20, -45 ≤ l ≤ 47 |
| Reflections collected | 21911 |
| Independent reflections | 21911 [R_int_ = ?, R_sigma_ = 0.2663] |
| Data/restraints/parameters | 21911/977/1097 |
| Goodness-of-fit on F^2^ | 1.027 |
| Final R indexes [I>=2σ (I)] | R_1_ = 0.1134, wR_2_ = 0.2507 |
| Final R indexes [all data] | R_1_ = 0.2074, wR_2_ = 0.2692 |
| Largest diff. peak/hole / e Å^-3^ | 1.11/-0.67 |

**
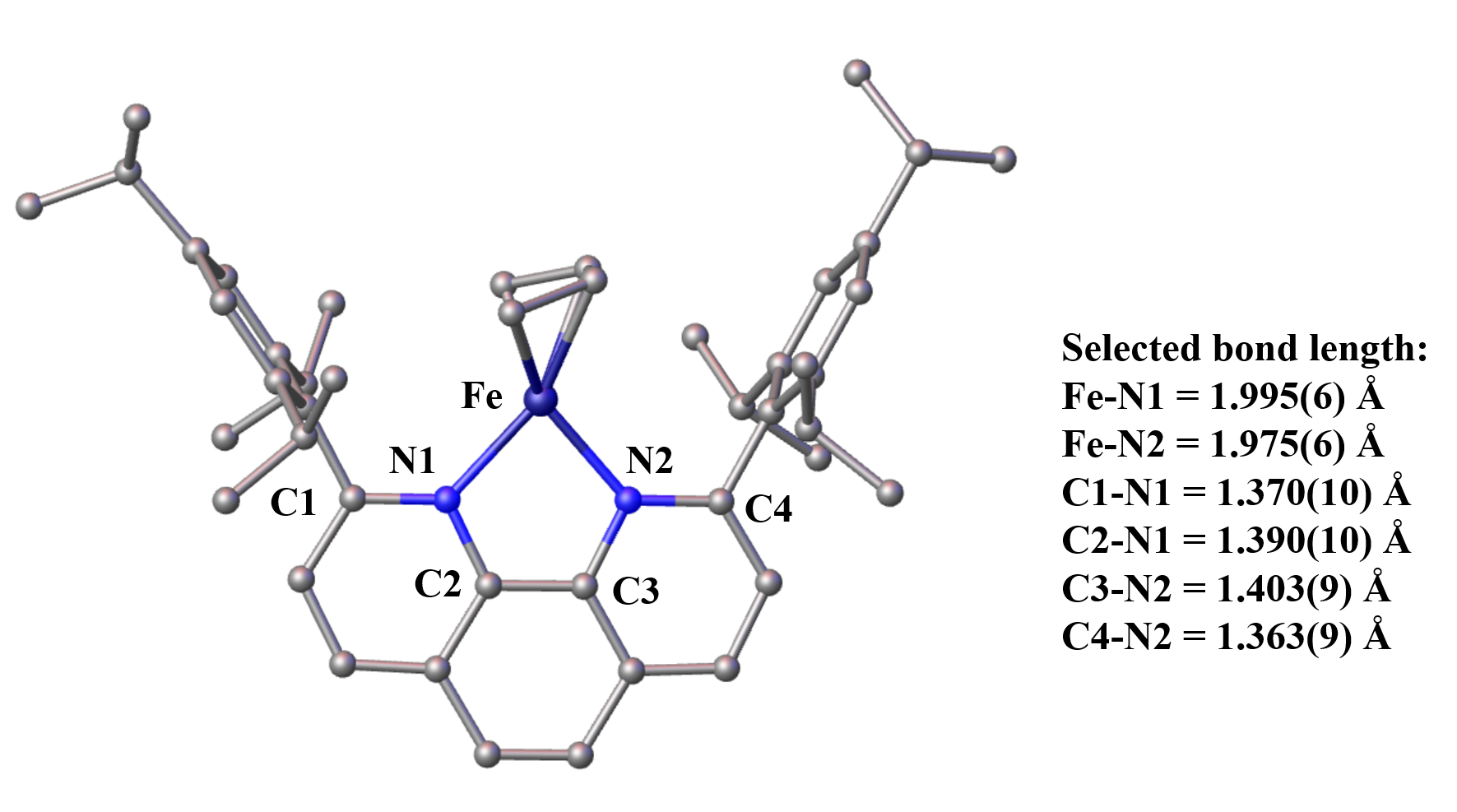
**

**Figure S23** Representation of the solid-state structure and selected bond length of **CA1**. Solvents, hydrogens and part of some other structures were omitted for clarity.

**Table S5** Crystal data and structure refinement for **CA2.**

| Empirical formula | C_50_H_70_FeN_2_OSi_2_ |
| --- | --- |
| Formula weight | 827.11 |
| Temperature/K | 113.15 |
| Crystal system | orthorhombic |
| Space group | Pna2_1_ |
| a/Å | 18.5455(4) |
| b/Å | 9.3818(2) |
| c/Å | 26.2247(5) |
| α/° | 90 |
| β/° | 90 |
| γ/° | 90 |
| Volume/Å^3^ | 4562.84(16) |
| Z | 4 |
| ρ_calc_g/cm^3^ | 1.204 |
| *μ*/mm^‑1^ | 0.421 |
| F(000) | 1784.0 |
| Crystal size/mm^3^ | 0.24 × 0.2 × 0.17 |
| Radiation | MoKα (λ = 0.71073) |
| 2Θ range for data collection/° | 4.66 to 62.612 |
| Index ranges | -26 ≤ h ≤ 25, -13 ≤ k ≤ 13, -36 ≤ l ≤ 36 |
| Reflections collected | 50690 |
| Independent reflections | 13666 [R_int_ = 0.0362, R_sigma_ = 0.0318] |
| Data/restraints/parameters | 13666/75/573 |
| Goodness-of-fit on F^2^ | 1.051 |
| Final R indexes [I>=2σ (I)] | R_1_ = 0.0452, wR_2_ = 0.1167 |
| Final R indexes [all data] | R_1_ = 0.0567, wR_2_ = 0.1279 |
| Largest diff. peak/hole / e Å^-3^ | 0.97/-0.78 |
| Flack parameter | 0.47(3) |

**
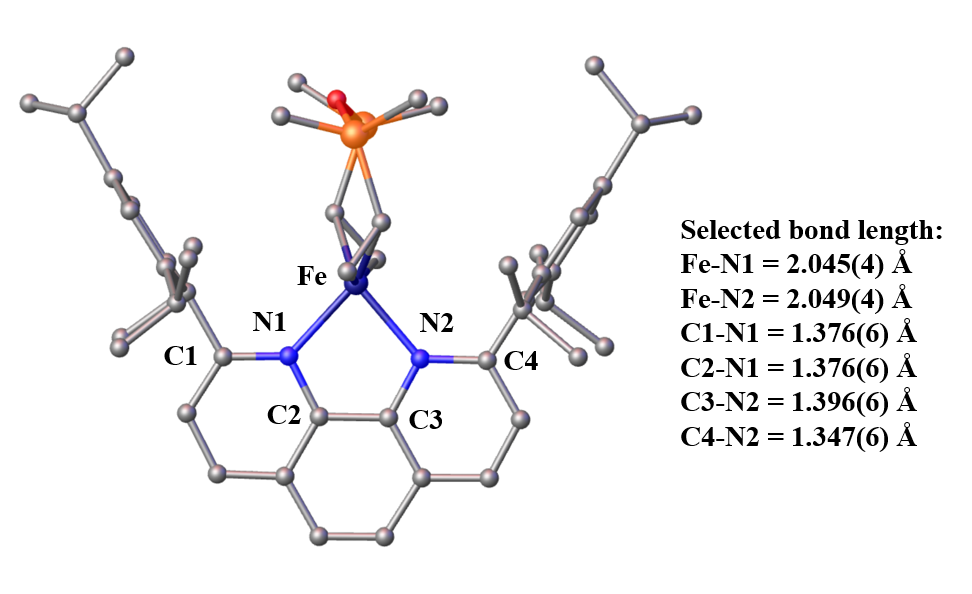
**

**Figure S24** Representation of the solid-state structure and selected bond length of **CA2**. Solvents, hydrogens and part of some other structures were omitted for clarity.

**Table S6** Crystal data and structure refinement for **CC1.**

| Empirical formula | C_34_H_34_FeN_2_ |
| --- | --- |
| Formula weight | 526.48 |
| Temperature/K | 150(2) |
| Crystal system | monoclinic |
| Space group | P2_1_/c |
| a/Å | 8.5779(2) |
| b/Å | 32.8325(8) |
| c/Å | 19.5779(4) |
| α/° | 90 |
| β/° | 90.270(2) |
| γ/° | 90 |
| Volume/Å^3^ | 5513.7(2) |
| Z | 8 |
| ρ_calc_g/cm^3^ | 1.268 |
| *μ*/mm^‑1^ | 0.572 |
| F(000) | 2224.0 |
| Crystal size/mm^3^ | ? × ? × ? |
| Radiation | MoKα (λ = 0.71073) |
| 2Θ range for data collection/° | 6.544 to 55.998 |
| Index ranges | -11 ≤ h ≤ 10, -34 ≤ k ≤ 43, -25 ≤ l ≤ 25 |
| Reflections collected | 59868 |
| Independent reflections | 12906 [R_int_ = 0.0912, R_sigma_ = 0.1067] |
| Data/restraints/parameters | 12906/53/679 |
| Goodness-of-fit on F^2^ | 1.017 |
| Final R indexes [I>=2σ (I)] | R_1_ = 0.0737, wR_2_ = 0.1536 |
| Final R indexes [all data] | R_1_ = 0.1450, wR_2_ = 0.1942 |
| Largest diff. peak/hole / e Å^-3^ | 0.99/-0.45 |


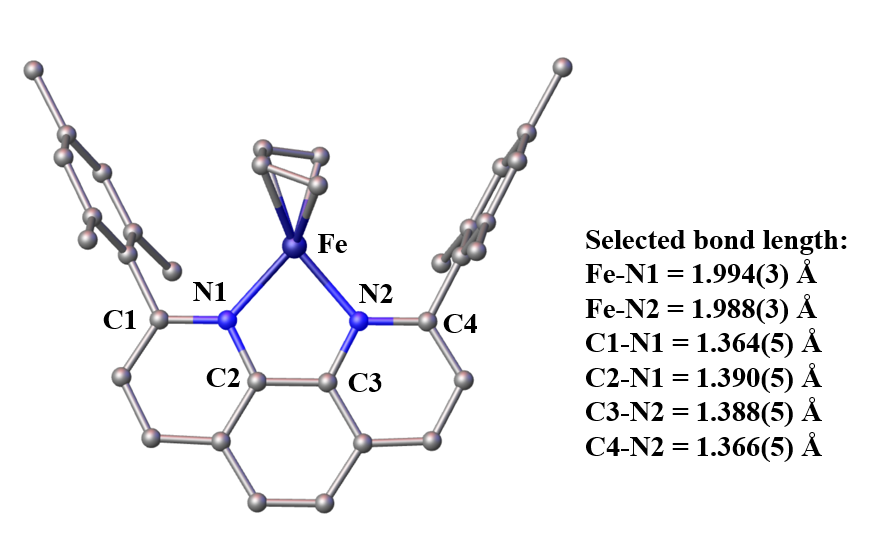


**Figure S25** Representation of the solid-state structure and selected bond length of **CC1**. Solvents, hydrogens and part of some other structures were omitted for clarity.

**Table S7** A comparison of Fe-N and C-N bonds length for solid structures **CA**, **CA1**, **CA2**, **CC**, and **CC1**.

| Structure | **CA** | **CA1** | **CA2** | **CC^1^** | **CC1** |
| --- | --- | --- | --- | --- | --- |
| Bonds/Å |  |  |  |  |  |
| Fe-N1 | 2.1456(14) | 1.995(6) | 2.045(4) | 2.1390(16) | 1.994(3) |
| Fe-N2 | 2.1468(15) | 1.975(6) | 2.049(4) | 2.1321(16) | 1.988(3) |
| C1-N1 | 1.341(2) | 1.370(10) | 1.376(6) | 1.336(2) | 1.364(5) |
| C2-N1 | 1.370(2) | 1.390(10) | 1.376(6) | 1.359(2) | 1.390(5) |
| C3-N2 | 1.370(2) | 1.403(9) | 1.396(6) | 1.364(2) | 1.388(5) |
| C4-N2 | 1.340(2) | 1.363(9) | 1.347(6) | 1.334(2) | 1.366(5) |

**^1^** Solid structure data obtained from our previous report[25].

The Fe-N bond lengths of the Fe(0) complex **CA2** (triplet state, μ_eff_ = 3.21 *μ_B_*) (average 2.047(4) Å) is shorter by about 0.099(4) Å, 4.6% compared to the Fe-N bond lengths of the Fe(II) complex **CA** (average 2.1462(15) Å). In addition to the reasons discussed in the main text, another possible factor is that the small electronegativity of silicon atoms and their large atomic radius have a significant hyperconjugation effect resulting in a higher electron density on the olefins and therefore amplifying the spin delocalization on the iron of the complex **CA2** (-0.69) compared to **CA1** (0.46) to the phenanthroline backbone. The Fe-N bond length pattern (**Table S7**) of **CC1**/**CC** is very similar to that of **CA1**/**CA** (main text). **CC1** bears a triplet ground state (S = 1, *μ*_eff_ = 3.28 *μ_B_*) and **CC** bears a quintet ground state (S = 2, *μ*_eff_ = 5.27 *μ_B_*). The Fe-N bond lengths of the complex **CC1** (average 1.991(3) Å) is shortened by 0.145(3) Å, 6.8% relative to the Fe-N bond lengths of the quintet complex **CC** (average 2.1356(16) Å) under the dual effect of the metal-ligand spin delocalization and spin state effect. Comparison of the C-N bond lengths of the 1,10-phenanthroline backbone revealed that the C-N bond lengths of all the Fe(0)complexes were longer than the corresponding Fe(II) precursors, again confirming the electronic structure that spin on Fe delocated to the ligand backbone, namely, the 1,10-phenanthroline backbone was reduced by the iron.

**8. Computational details**

**Table S8** A comparison of spin split energies (kcal/mol) computed at different theory methods and basis sets. Energies reported are the Gibbs free energy difference of triplet and quintet of the complex (ΔG_(S=1)_ ‒ ΔG_(S=2)_).

| **entry** | **opt+freq** | **sp** | **CA** | **CA1** | **CA2** | **CC** | **CC1** |
| --- | --- | --- | --- | --- | --- | --- | --- |
| **1** | b3lyp-D3(BJ)/6-311g* | wB97XD/def2TZVPP | -- | -- | -- | -- | -11.0 |
| **2** | b3lyp-D3(BJ)/6-311g* | wB97XD/def2TZVPP-CPCM(THF) | -- | -- | -- | -- | -11.7 |
| ***3*** | *wB97XD/6-31g*-TZVP(Fe)* | *wB97XD/def2TZVPP-CPCM(THF)* | *33.7* | *-4.3* | *-0.1* | *25.7* | *-3.9* |
| **4** | wB97XD/6-31g*-TZVP(Fe) | PBE0/def2TZVPP-CPCM(THF) | -- | -- | -- | -- | -4.1 |
| **5** | wB97XD/6-31g*-TZVP(Fe) | M062x/def2TZVPP-CPCM(THF) | -- | -- | -- | -- | -1.3 |
| **6** | TPSSh/6-31g*-TZVP(Fe) | TPSSh-D3(BJ)/def2TZVPP-CPCM(THF) | -- | -- | -6.4 | -- | -4.8 |
| **7** | TPSSh/6-31g*-TZVP(Fe) | wB97XD/def2TZVPP-CPCM(THF) | -- | -- | -1.0 | -- | -3.3 |

We have selected some widely used general functionals and basis sets for calculating open-shell transition metal complex systems for comparison [26]. Overall, keeping the basis set constant for structure optimization [6-31g*-TZVP(Fe)] and single point energy calculations [def2TZVPP-CPCM(THF)], there is little difference in the spin split energy gap between the triplet and quintuplet states of **CC1** at each calculation level (-1.3 ‒ 4.8 kcal/mol), and we finally choose wB97XD/def2TZVPP-CPCM(THF) || wB97XD/6-31g*-TZVP(Fe) for the calculation as mentioned in the General considerations. Since the spin split energy of **CA2** is small at the above levels, further calculations using the methods shown in **entry 6** and **entry 7** show that basically the triplet state has a lower energy than the quintet state, which agrees with the magnetic moment data.

**Figure S26** Energy profiles for catalyst precursor **CC** in catalytic cycle involving singlet (black), triplet (red), and quintet (blue) spin states, as determined by DFT calculations. Numbers in parenthesis indicate electronic energy. Numbers in square brackets indicate spin population on Fe and ligand, respectively.

Since the energies of the mono-alkyne coordinated complex **^5^Int-1'** and the mono-hydrosilane coordinated complex **^3^Int-1''** are 5.3 and 21.4 kcal/mol higher, respectively, than that of **^3^Int-1** with bis-alkyne coordination, it is likely that the catalyst precursors coordinate mainly with two alkynes after being reduced to Fe(0). According to CMAC, the iron center of oxidative addition transition state **Ts-1** had a lower charge in the triplet state (**^3^Ts-1**, 0.32) than in the corresponding quintet state (**^5^Ts-1**, 0.50), and thus, the triplet **^3^Ts-1** with a higher electron density on the iron center favored the oxidative addition process. The variation of the charge on the 1,10-phenanthroline ligand backbone from **Int2** to **Ts1** (ligand charge variation in triplet potential energy surface, **Int2**–**Ts1**: −0.27 to 0.35; quintet potential energy surface, **Int2**–**Ts1**: −0.34 to −0.20) revealed the origin of the above metal charge difference. The iron center of the triplet iron catalyst apparently took a larger number of electrons from the ligand than its quintet counterpart. Thus, the triplet iron catalyst was more favorable for the oxidative addition. The above phenomenon was further confirmed by the spin population analysis from **Int2** to **Ts1** (**Table S12** for visualization plots). The spin population changed from **^3^Int-2** (3, -1) to **^3^Ts-1** (2, 0), indicating a ligand *β*-electron transfer to the metal center, resulting in the charge on Fe changing from 0.63 to 0.32 (**Table S9 and 10**), promoting oxidative addition. In contrast, there was no significant change in the catalyst charge and spin population from **^5^Int-2** to **^5^Ts-1** under the quintet potential energy surface. In the reductive elimination process, an *α*-electron on Fe was transferred to the ligand during the process of **^5^Int-3** (4, 0) to **^5^Ts-2** (3, 1). However, in the triplet potential energy surface, there was no significant change in the electron spin and charge population from **^3^Int-3'** to **^3^Ts-2**. As a result, the metal charge of the quintet transition state **^5^Ts-2** was 0.47, which was higher than the metal charge of the triplet transition state **^3^Ts-2** (0.15), thus making it easier for reductive elimination to occur.

**Figure S27** Energy profiles for catalyst precursor **CD** in catalytic cycle involving singlet (black), triplet (red), and quintet (blue) spin states, as determined by DFT calculations. Numbers in parenthesis indicate electronic energy. Numbers in square brackets indicate spin population on Fe and ligand, respectively.

We also calculated the reaction process catalyzed by **CD** that afford *α*-selective (**Figure S27**), which is similar to the mechanism of the *β*-selective process, where the complex **CD** was reduced to a low-valent iron species, which later coordinates with the alkynes present in the system to produce the bis-alkyne complex **Int-1**, followed immediately by the coordination exchange of the hydrosilane with a alkyne to produce the intermediate **Int-2**. Subsequently, the hydrogen atom underwent the transition state LLHT (ligand to ligand hydrogen transfer) through **TS-1** and migrated from the silicon atom of **Int-2** to the carbon-carbon triple bond to generate **Int-3**. **Int-3** then underwent reductive elimination through **TS-2** to afford **Int-4**. Similar to the *β*-selective process, **^3^Ts-1** had a lower energy of 12.7 kcal/mol compared to **^1^Ts-1** and **^5^Ts-1**, so the oxidative addition process took place on the triplet potential energy surface. In the reduction elimination step, since the energy of **^3^Int-3** (6.0 kcal/mol) and **^1^Int-3** (13.4 kcal/mol) were already greater than the energy of **^5^Ts-2** (4.3 kcal/mol), so the energy of the corresponding triplet and singlet transition states were ment to be greater than the energy of **^5^Ts-2**, so the reductive elimination process took place on the quintet potential energy surface. The reaction energy barrier was 15.0 kcal/mol. In both *α*-selective and *β*-selective reactions the reaction energy barriers of the oxidative addition (LLHT) processes took place in the triplet potential energy surfaces, and the reductive elimination processes took place in the quintet potential energy surfaces, which meant that spin crossover played an important role in the iron-catalyzed alkyne hydrosilylation reaction.

**Table S9** Mulliken spin population and Mulliken charge on Fe and ligand.

|  | Fe | | Ligand | |
| --- | --- | --- | --- | --- |
|  | Charge | Spin population | Charge | Spin population |
| **^3^Int-1** | 0.83171 | 2.34947 | 0.147019 | 0.022668 |
| **^5^Int-1ʹ** | 0.59659 | 3.19503 | -0.386086 | 1.030187 |
| **^3^Int-2** | 0.62695 | 2.99107 | -0.274973 | -0.799968 |
| **^5^Int-2** | 0.53035 | 3.16728 | -0.335962 | 1.047704 |
| **^3^Ts-1** | 0.32314 | 2.21603 | 0.352663 | -0.020305 |
| **^5^Ts-1** | 0.50034 | 2.99423 | -0.203142 | 1.082492 |
| **^3^Int-3** | 0.34352 | 2.08527 | 0.392832 | -0.006264 |
| **^5^Int-3** | 0.62471 | 3.77499 | 0.306687 | 0.091968 |
| **^3^Ts-2** | 0.14890 | 2.11257 | 0.196359 | -0.065041 |
| **^5^Ts-2** | 0.47263 | 3.32950 | -0.151387 | 0.850698 |
| **^3^Int-4** | 0.29781 | 1.87578 | 0.049470 | 0.395503 |
| **^5^Int-4** | 0.52643 | 3.22814 | -0.394966 | 1.041658 |
| **M-^3^Int-1ʹ** | 0.60301 | 3.08780 | -0.328099 | -0.841144 |
| **M-^5^Int-1** | 0.92279 | 3.18548 | -0.512220 | 1.085452 |
| **M-^3^Int-2** | 0.60190 | 2.40686 | 0.169336 | -0.016035 |
| **M-^5^Int-2** | 0.66968 | 3.17183 | -0.431303 | 1.059767 |
| **M-^3^Ts-1** | 0.59360 | 2.21304 | 0.250488 | 0.011552 |
| **M-^5^Ts-1** | 0.75450 | 3.02312 | -0.374187 | 1.072143 |
| **M-^3^Int-3** | 0.41528 | 2.09228 | 0.212622 | 0.012598 |
| **M-^5^Int-3** | 0.52213 | 3.77423 | 0.257488 | 0.115386 |
| **M-^5^Ts-2** | 0.31277 | 3.55245 | 0.110694 | 0.357097 |
| **M-^3^Int-4** | 0.52024 | 3.13241 | -0.429899 | -0.808926 |
| **M-^5^Int-4** | 0.50065 | 3.21075 | -0.461292 | 1.050461 |

**Table S10** Selected Mulliken charge population on Fe and ligand (red, positive charges; blue, negative charges. Atoms were darker in color with more charges.).

**
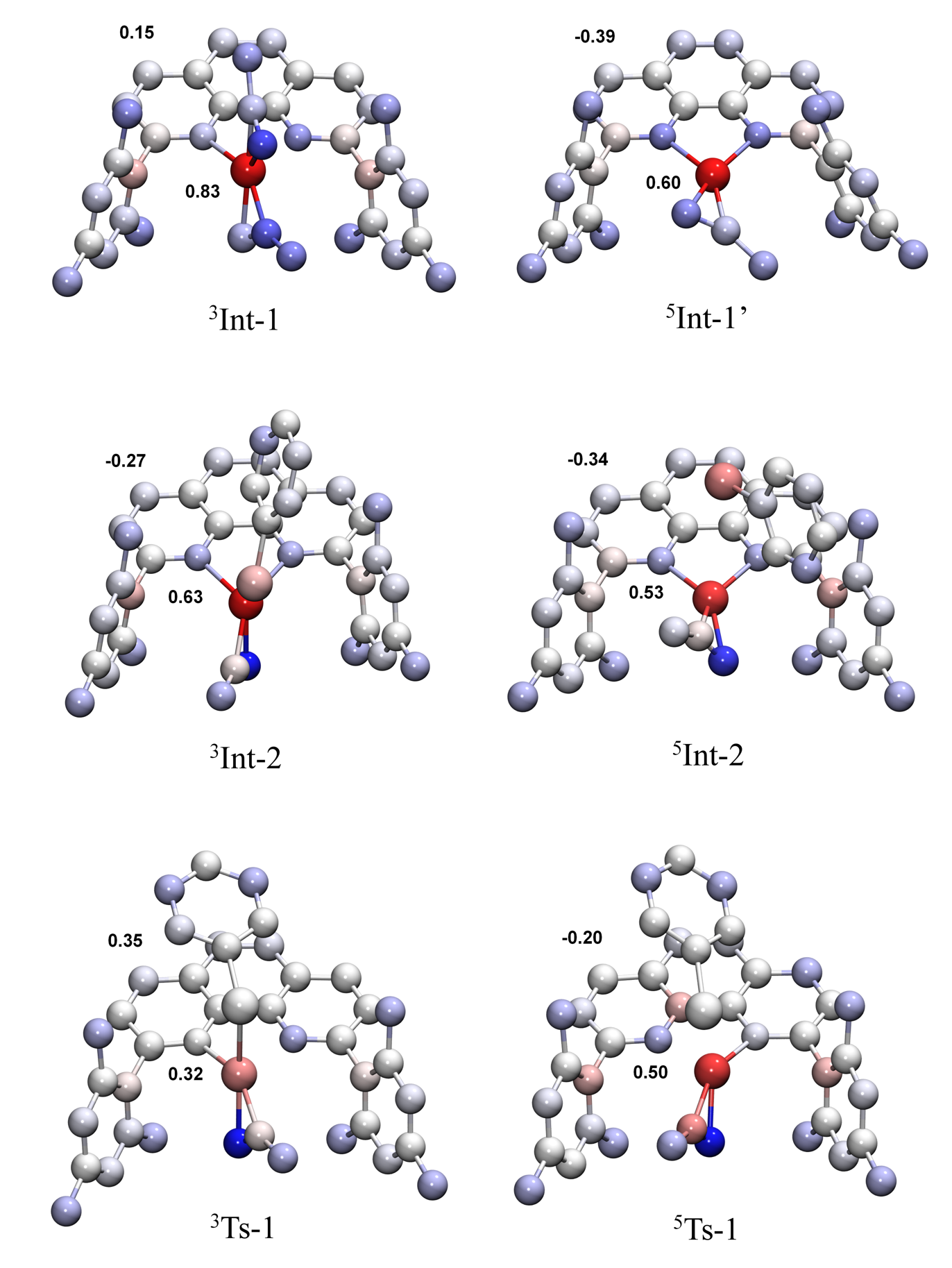
**

**
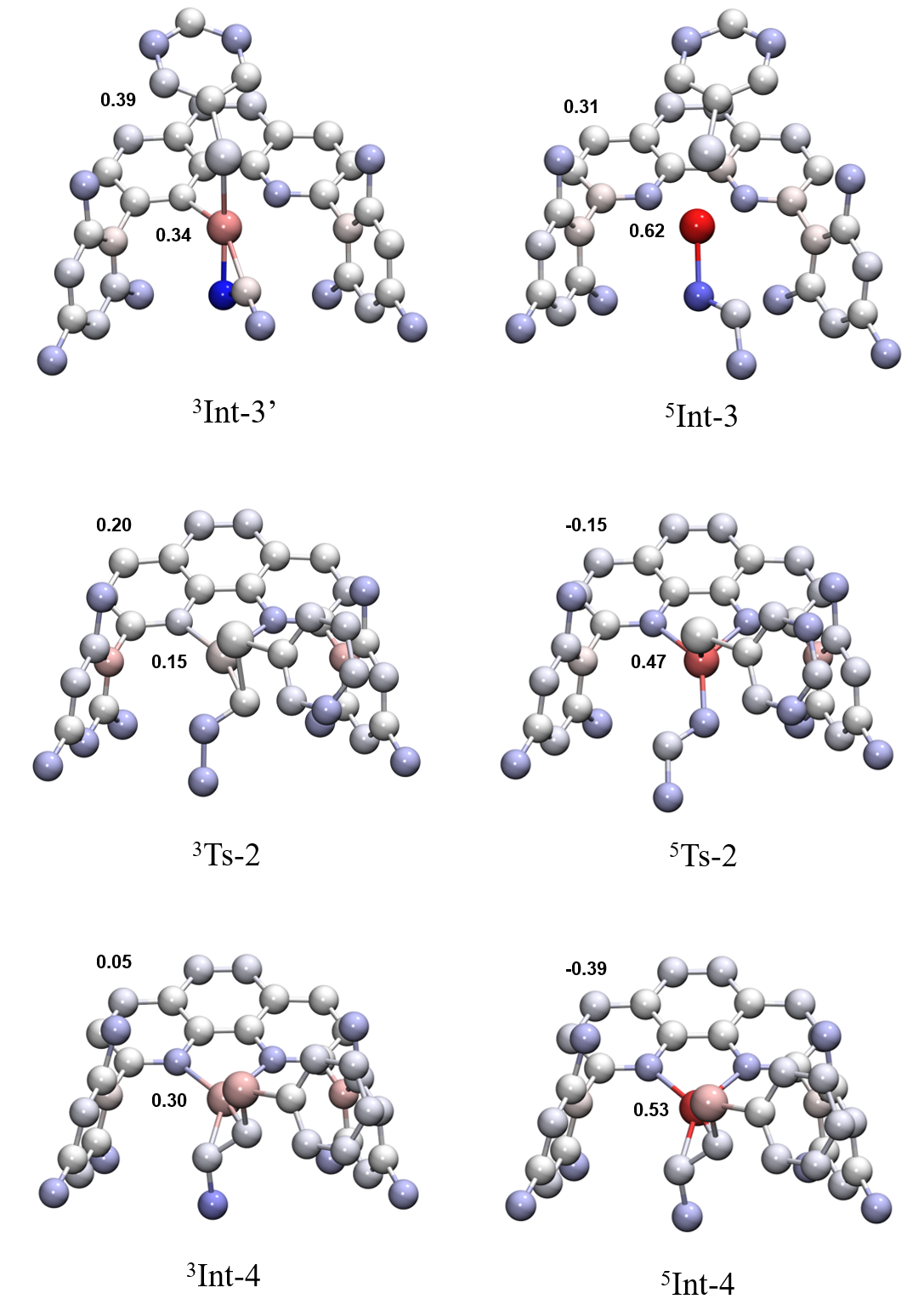
**

**
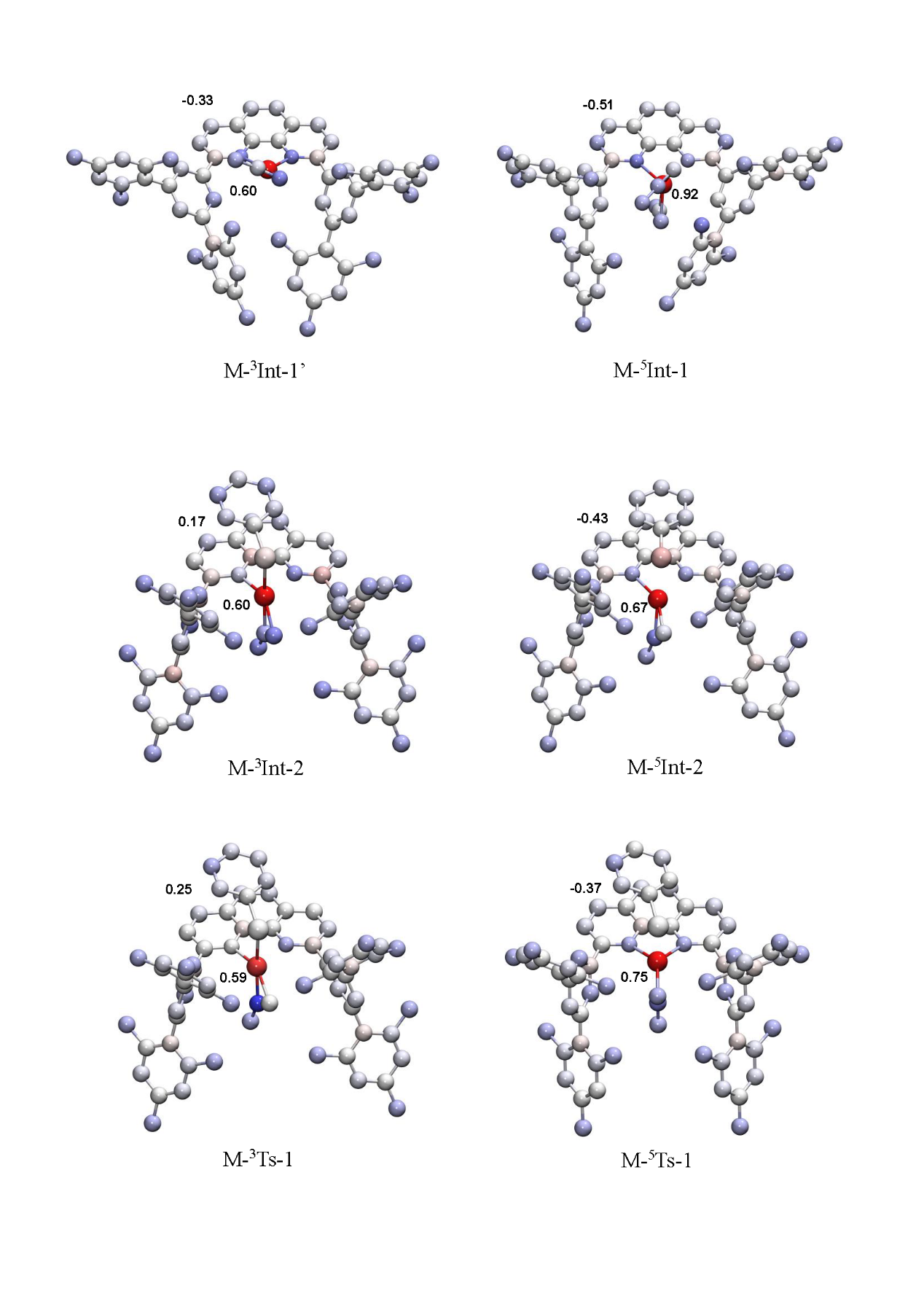
**

**
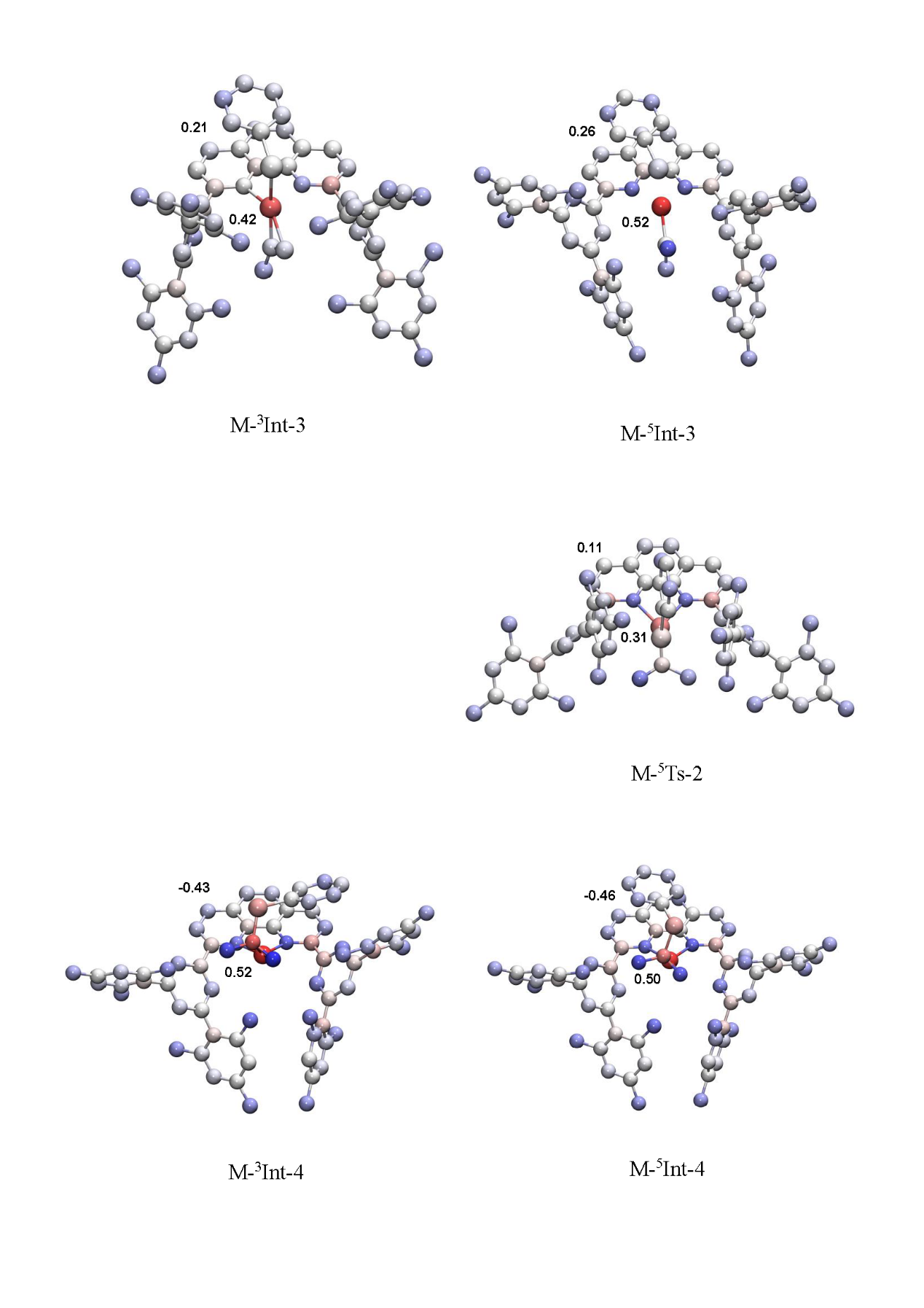
**

**Table S11** Mulliken spin population on Fe and ligand (green, positive spin density; blue, negative spin density) for catalyst precursor and pre-prapared Fe(0) species.

**
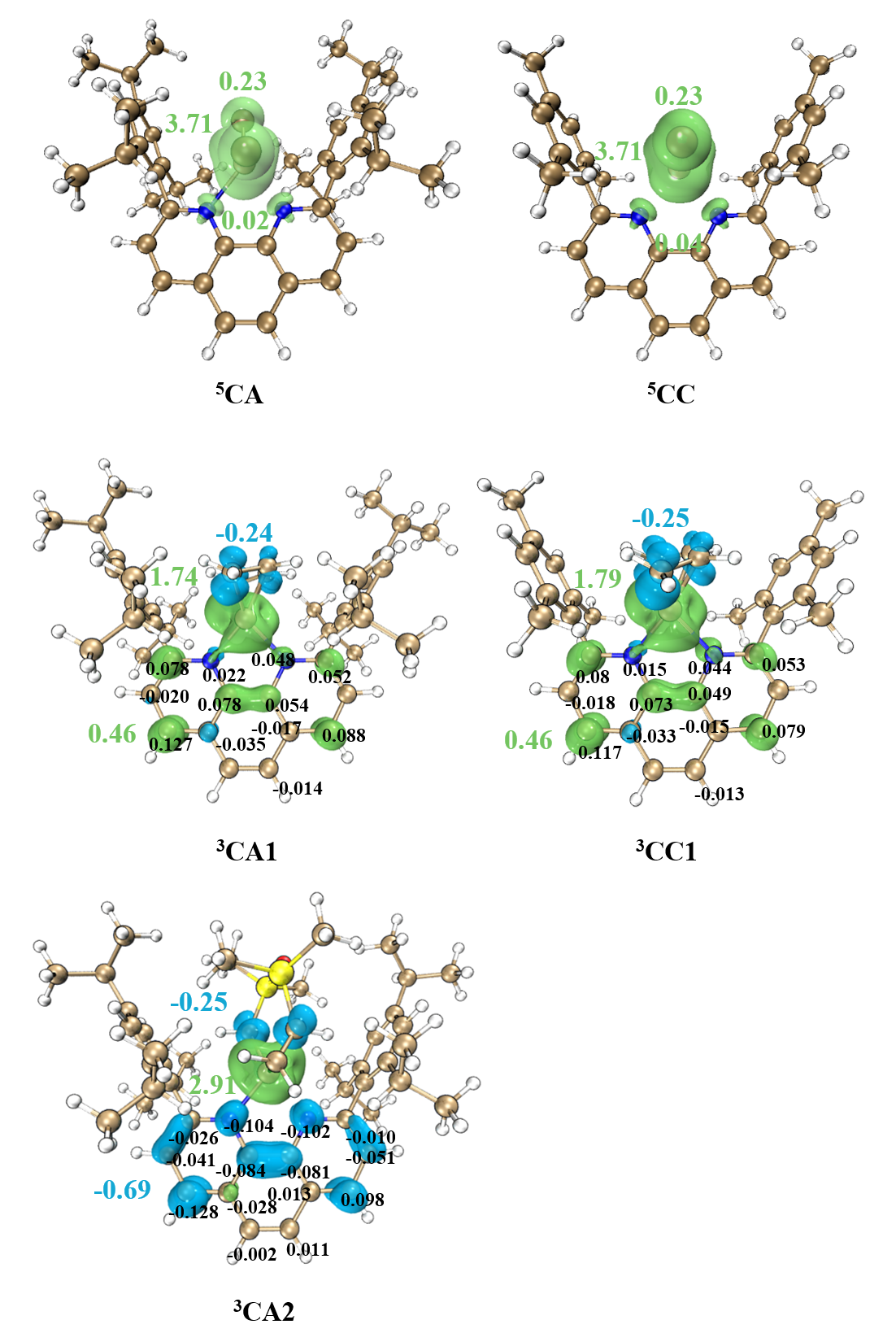
**

**Table S12** Frontier orbitals of **CA1** and **CA2** after wavefunction biorthogonalization. There is little difference between most *α* and *β* orbital shapes, so *α* orbitals were reported unless otherwise mentioned.

**CA1** possesses two unpaired electrons, each occupying one of the two highest occupied orbitals. One of these orbitals is primarily composed of Fe_3d_ orbitals, while the other orbital (HOMO-1) has 47% Fe_3d_-character. The remaining components are mainly attributed to the π* orbitals of the 1,10-phenanthroline ligand, implying a strong covalent interaction between Fe and 1,10-phenanthroline (especially the C-N bonds). This could explain the shorter Fe-N bonds and the longer C-N bonds in this complex compared to the divalent iron precursor **CA**. These two orbitals contribute significantly to the complex's main spin density. Examining HOMO-2, iron (59%) exhibits strong backbonding to the π* orbital of 1,3-butadiene, suggesting a pronounced π-acceptor capability of 1,3-butadiene.

**CA2** contains four unpaired electrons, each occupying one of the four highest occupied orbitals. Three of these orbitals are primarily composed of Fe_3d_ orbitals, while the remaining orbital is mainly composed of the π* orbitals of the 1,10-phenanthroline ligand, with 15% Fe_3d_ character, and is occupied by a *β* electron. This indicates a covalent interaction between Fe and the 1,10-phenanthroline ligand, although the lower Fe_3d_-character suggests that this interaction appears weaker compared to that of **CA1**. Consequently, the Fe-N bonds in **CA2** are shorter compared to **CA** but longer to **CA1**. This orbital occupancy aligns with the spin distribution. Examining the HOMO-3 orbital, iron also exhibits some backbonding to the enyl π* orbitals of the coordinated dvtms, but 83% Fe_3d_-character of this orbital (less enyl π* contributions) suggests a weaker backbonding interaction than that in **CA1** (59% Fe_3d_-character). This difference may be attributed to the lower π-acceptor capability of dvtms compared to 1,3-butadiene.

**Table S13** Mulliken spin population on Fe and ligand (red, positive spin density; yellow, negative spin density) for selected intermediates and transition states. Spin populations were plotted in a different way from **Figure S11** for species involved in the catalytic reaction.

**
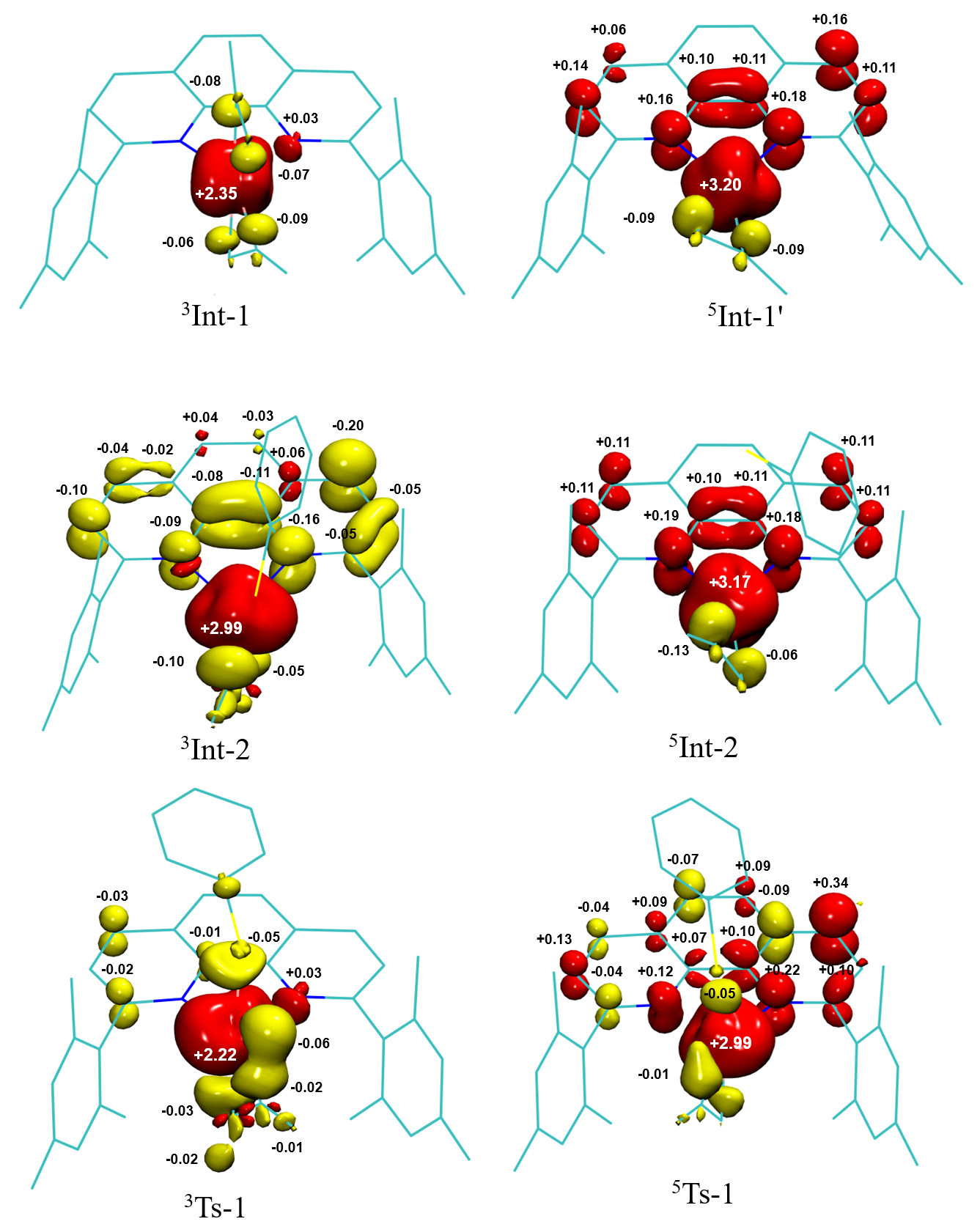
**

**
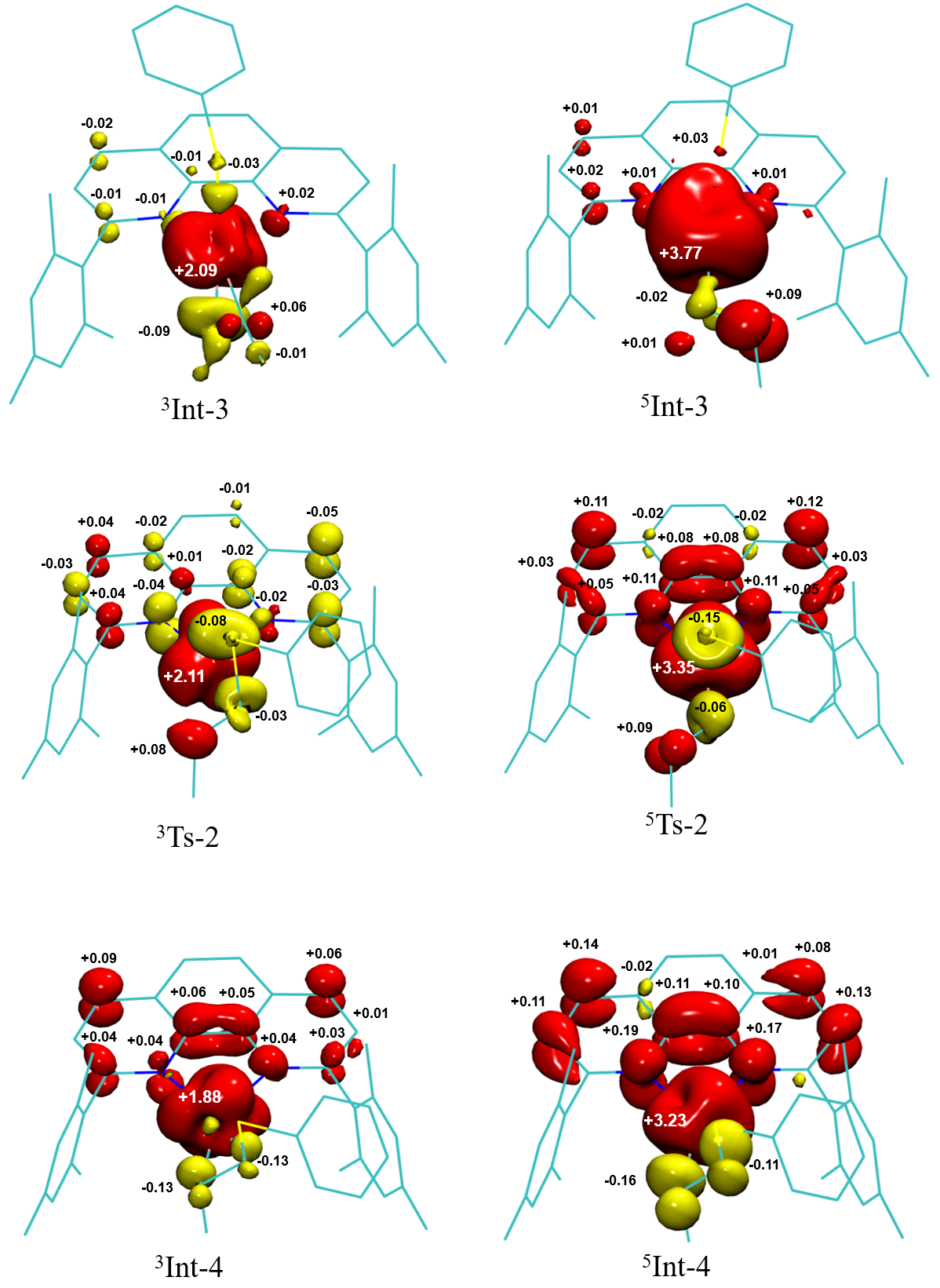
**

**
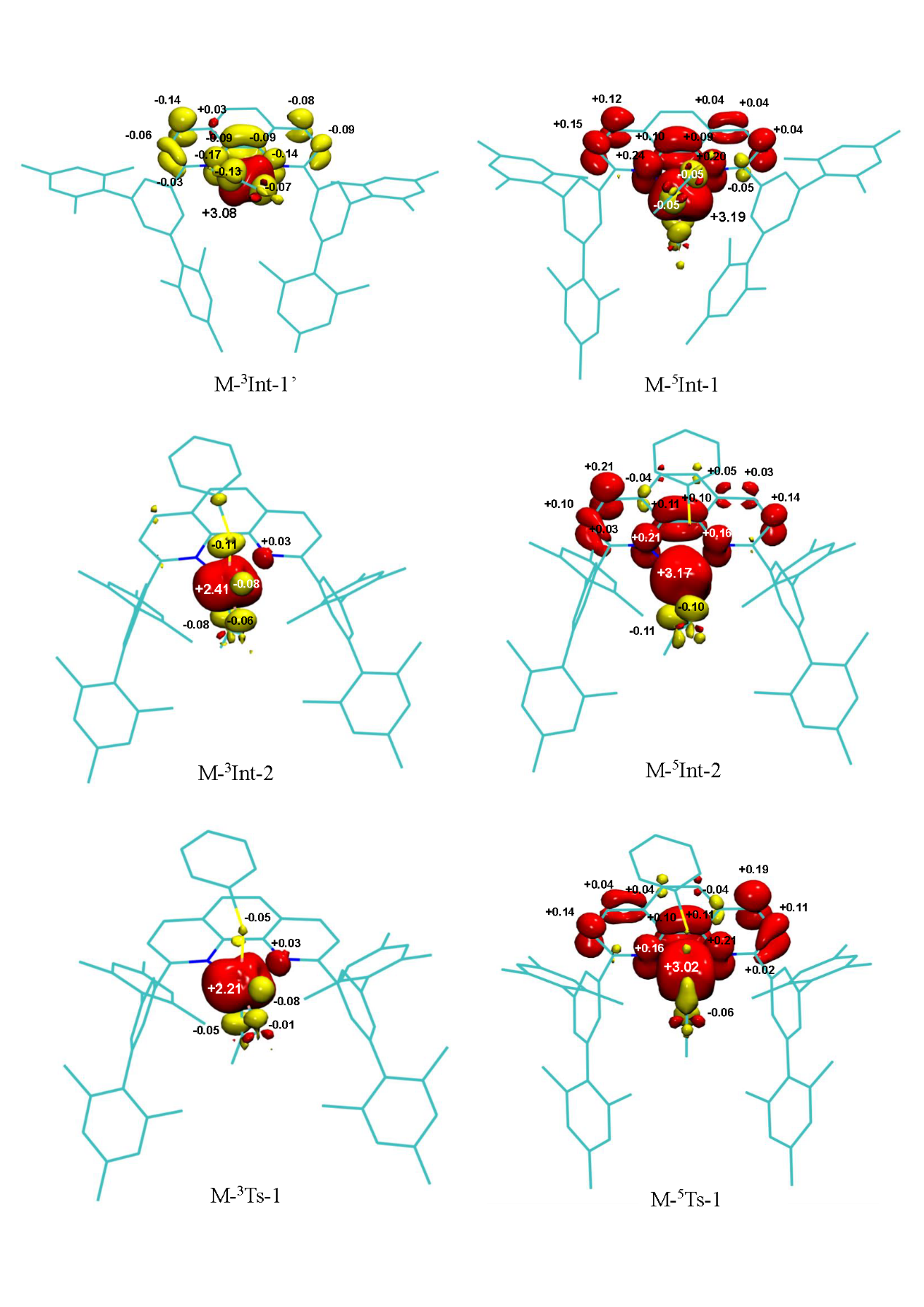
**

**
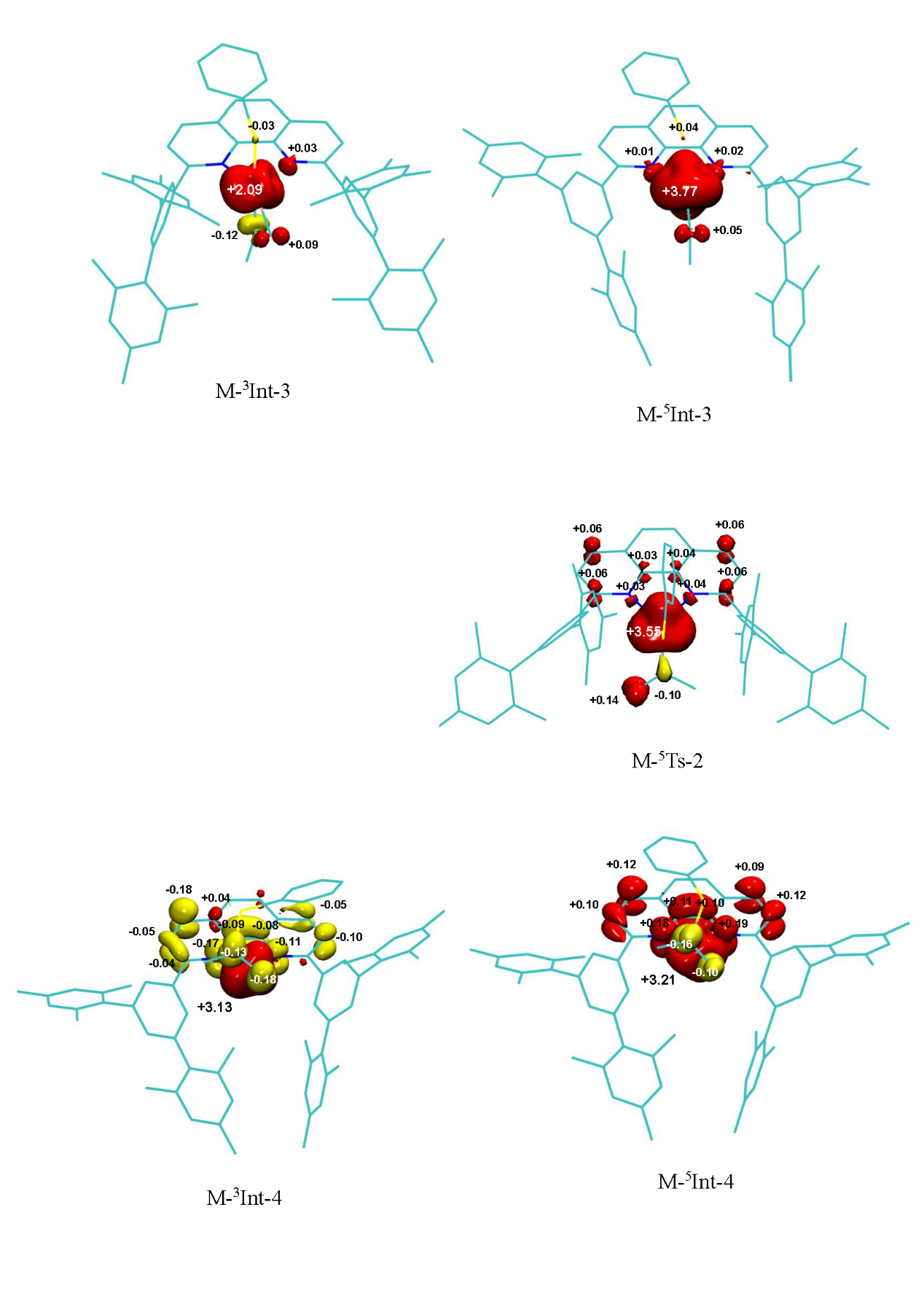
**

**Table S14** Selected frontier orbitals after wavefunction biorthogonalization. There is little difference between and *α* and *β* orbital shapes, so *α* orbitals were reported unless otherwise mentioned (**^3^Int-2**). Energies were reported in eV.

**
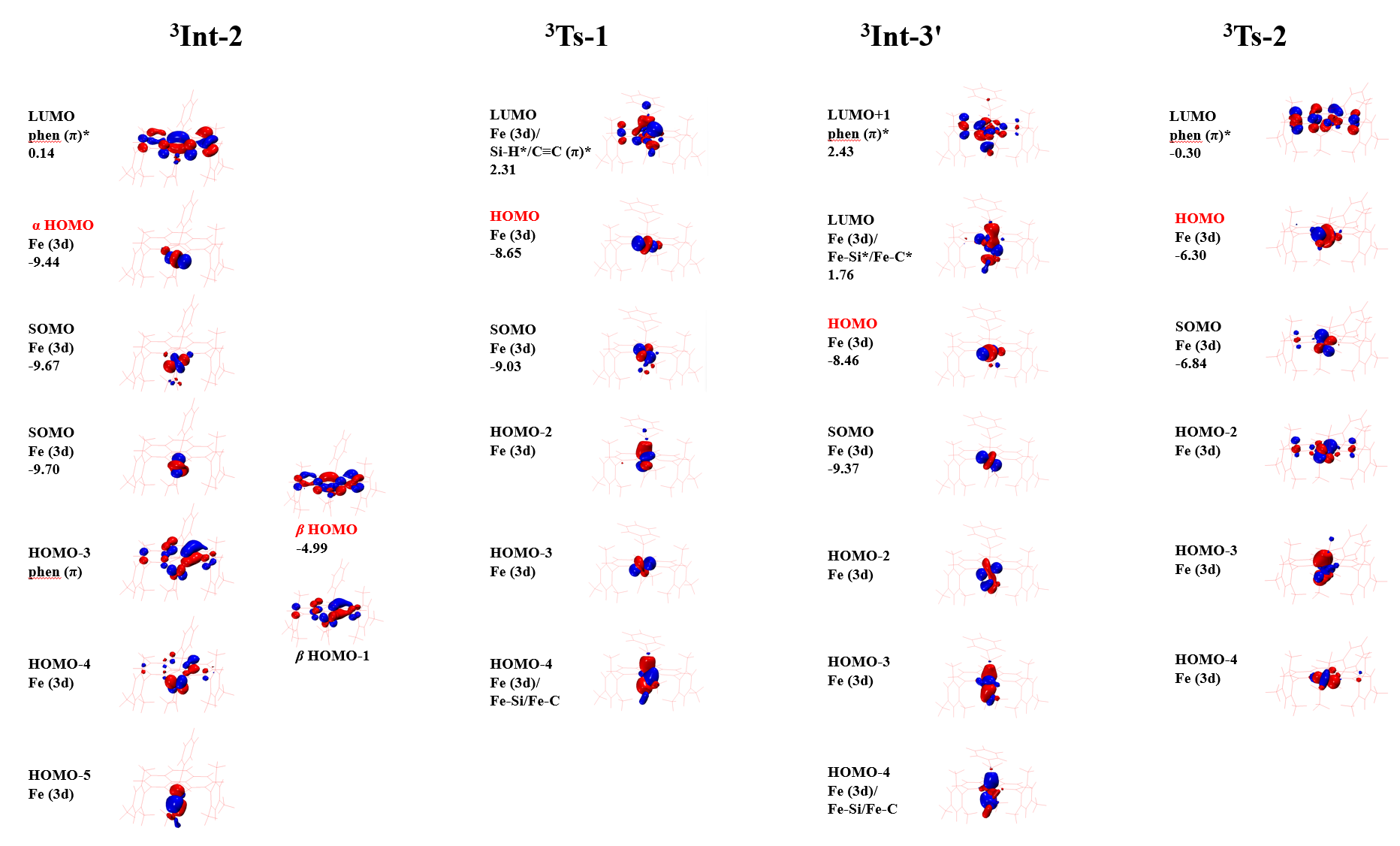
**

**
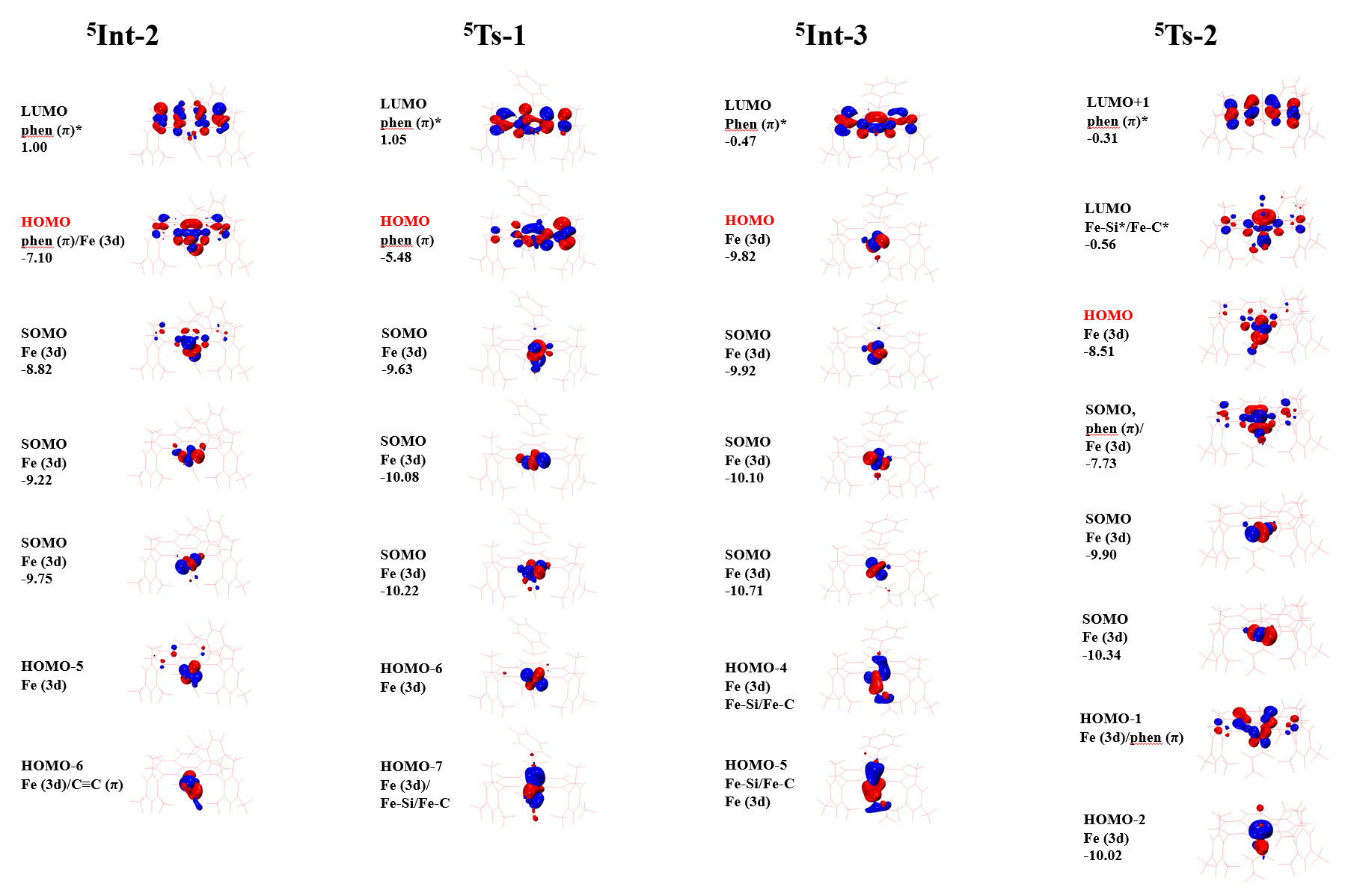
**

**Figure S28** Interaction region indicator (IRI) analysis for **Ts-1**. IRI analysis shows various intramolecular interactions (including chemical bonds) by different colors according to the color bar.

**Figure S29 (A)** IGMH analysis for *α* selectivity when catalyst **CD** was applied. **(B)** Geometry analysis of **Ts-1** with different spin states. Distance between the center of the phenyl on the silyl group and the plane of 1,10-phenanthroline were shown as an indicator of the π-π stacking interaction. Distance between atoms or fragments were reported in Å. In the quintet transition state **M-^5^Ts-1**, spin delocalization causes the phenyl group of the silyl to deviate from being parallel to the plane of 1,10-phenanthroline. This deviation increases the distance between these two fragments, thereby weakening the π-π stacking interaction.

**Table** **S15** Calculated Corrected Gibbs free energy and electronic energy of catalyst precursor and pre-prepared Fe(0) complexes.

| **Structures** | **Electronic energy/ Hartree** | **Thermal correction to Gibbs Free Energy (ΔG_corr_.)/ Hartree** |
| --- | --- | --- |
| **^3^CA**  **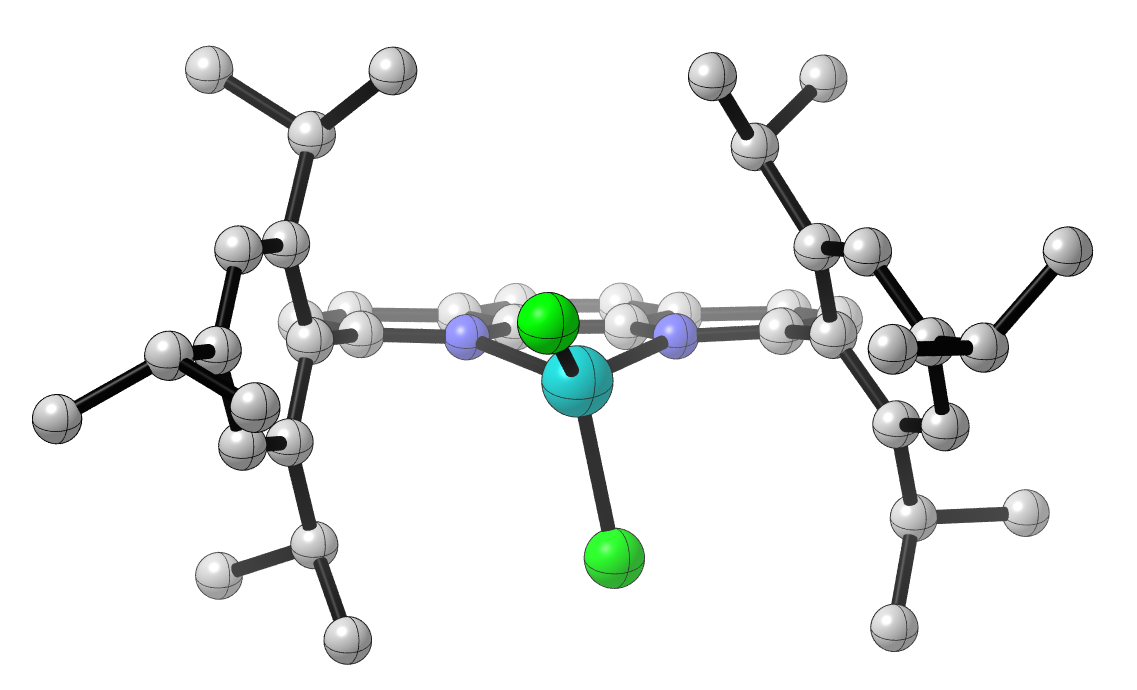** | E = -3454.5163426 | 0.434342 |
| **^5^CA**  **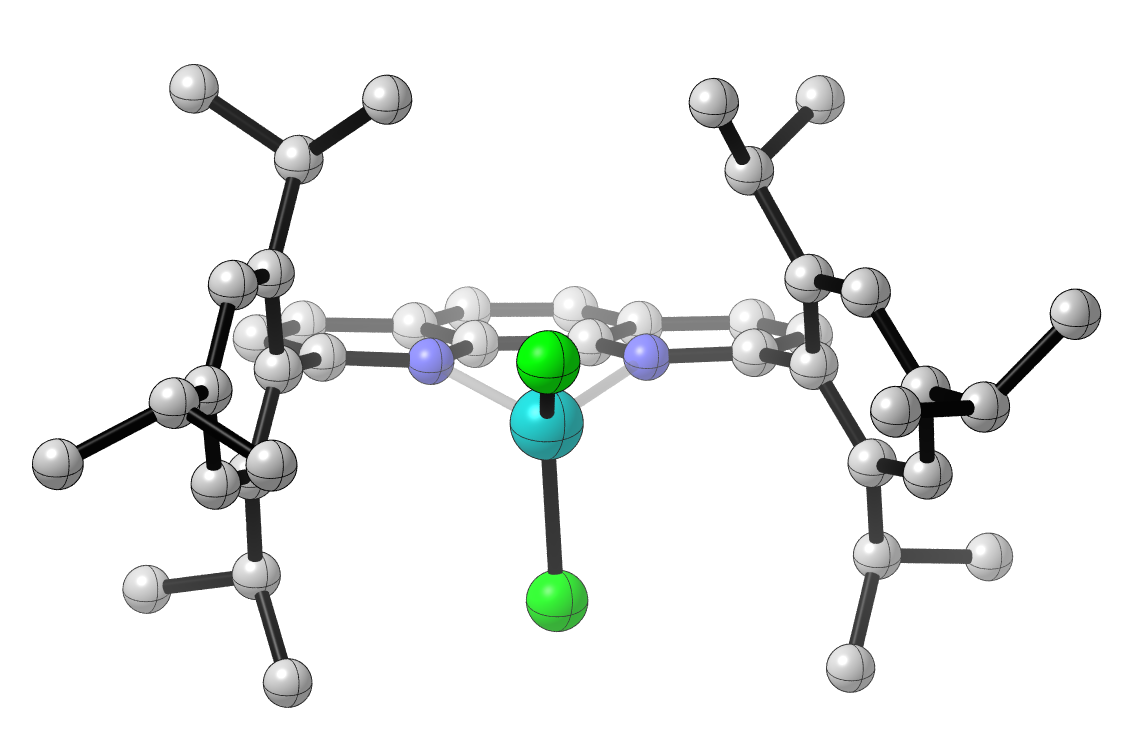** | E = -3454.5768270 | 0.432196 |
| **^3^CA1**  **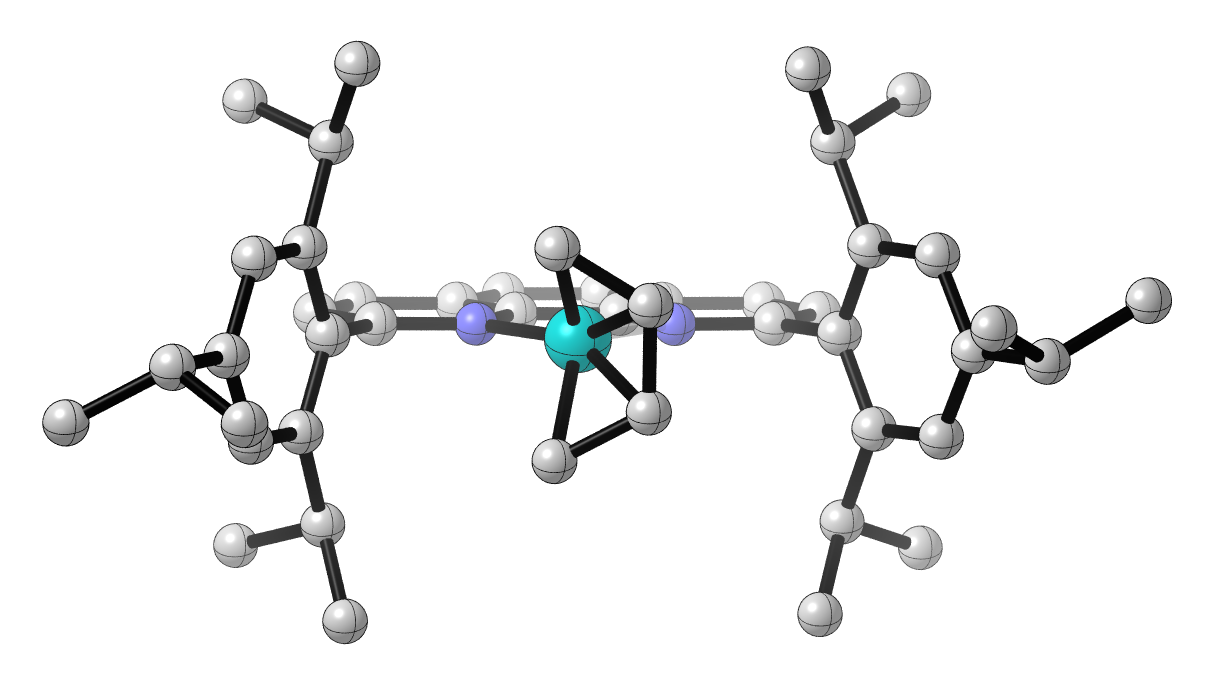** | E = -3161.2480680 | 0.842580 |
| **^5^CA1**  **^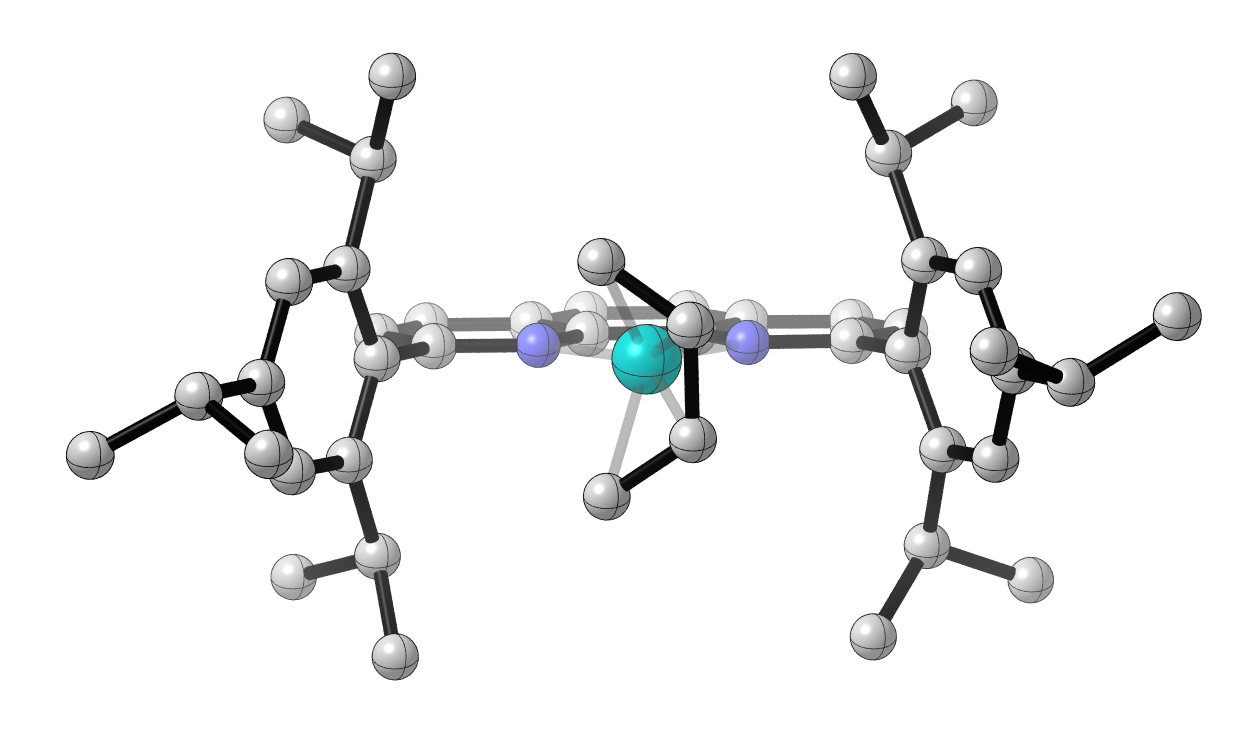^** | E = -3161.231840 | 0.838099 |
| **^3^CA2**  **^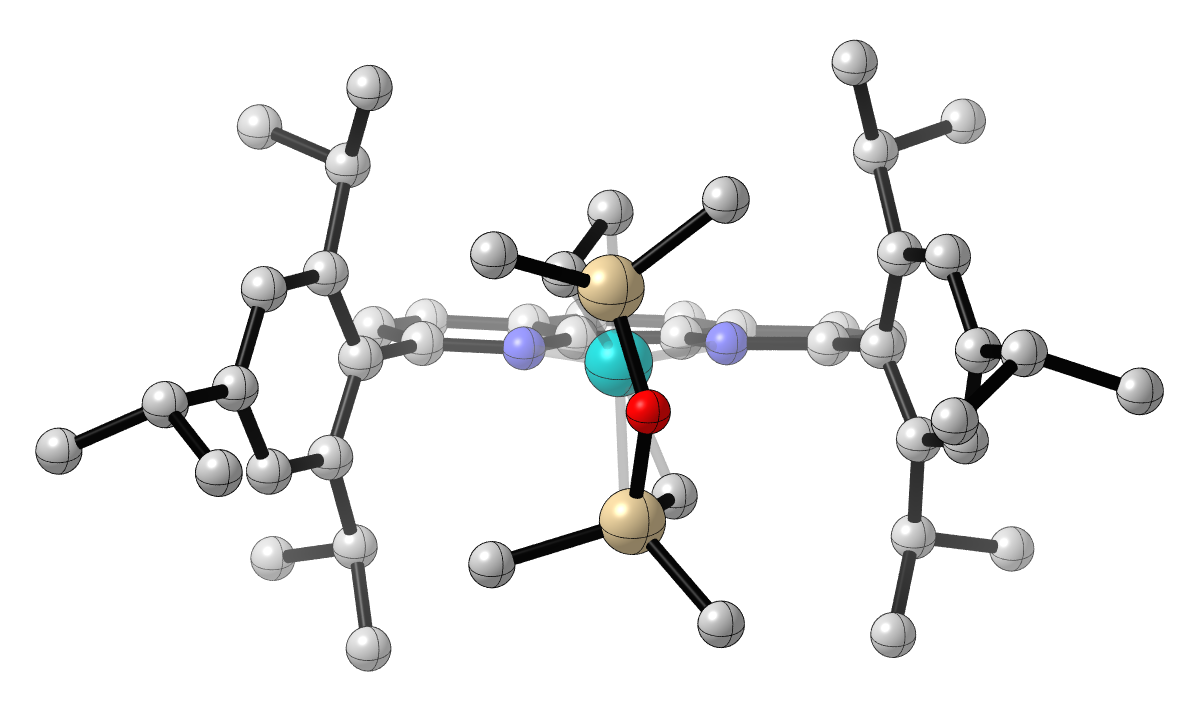^** | E = -3975.3529074 | 0.985469 |
| **^5^CA2**  **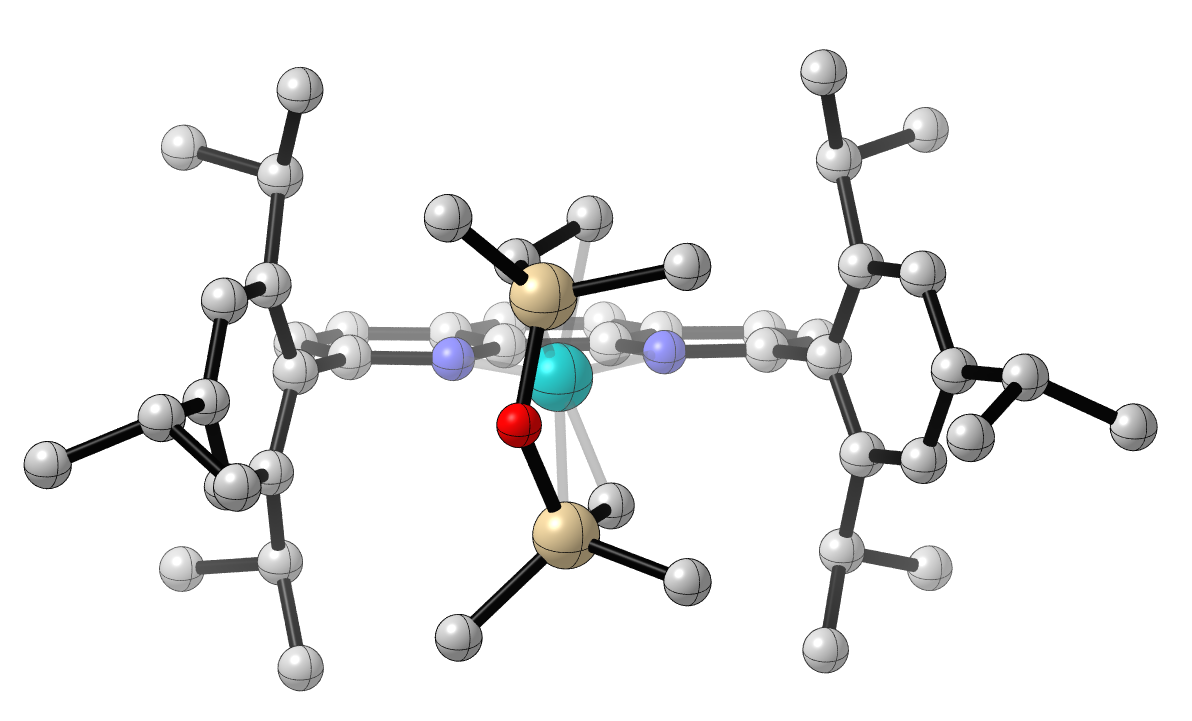** | E = -3975.3515536 | 0.987161 |
| **^3^CC**  **^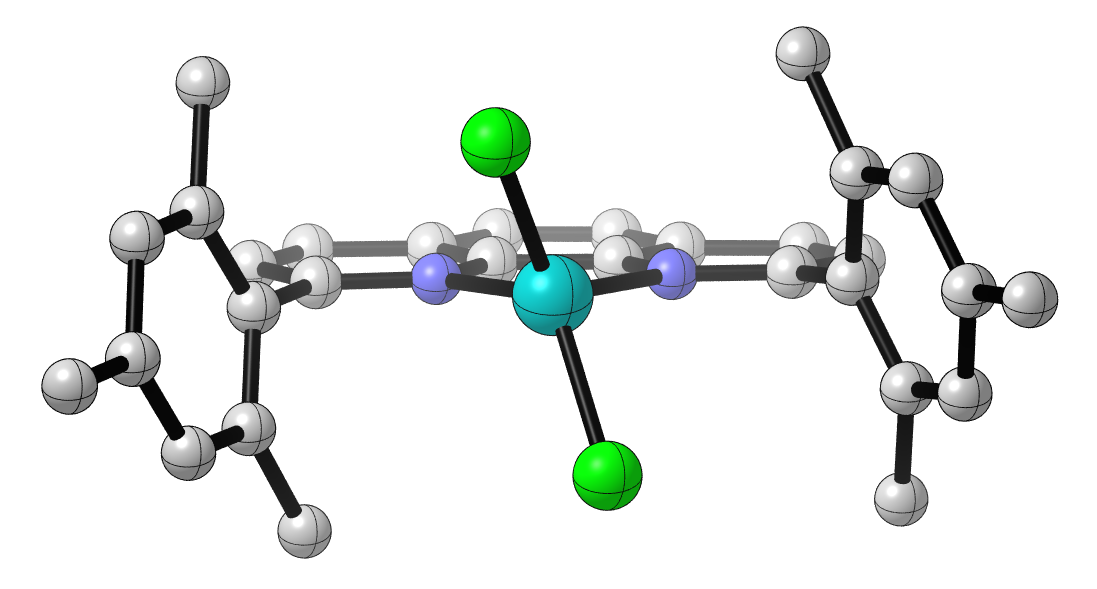^** | E = -3454.5163426 | 0.434342 |
| **^5^CC**  **^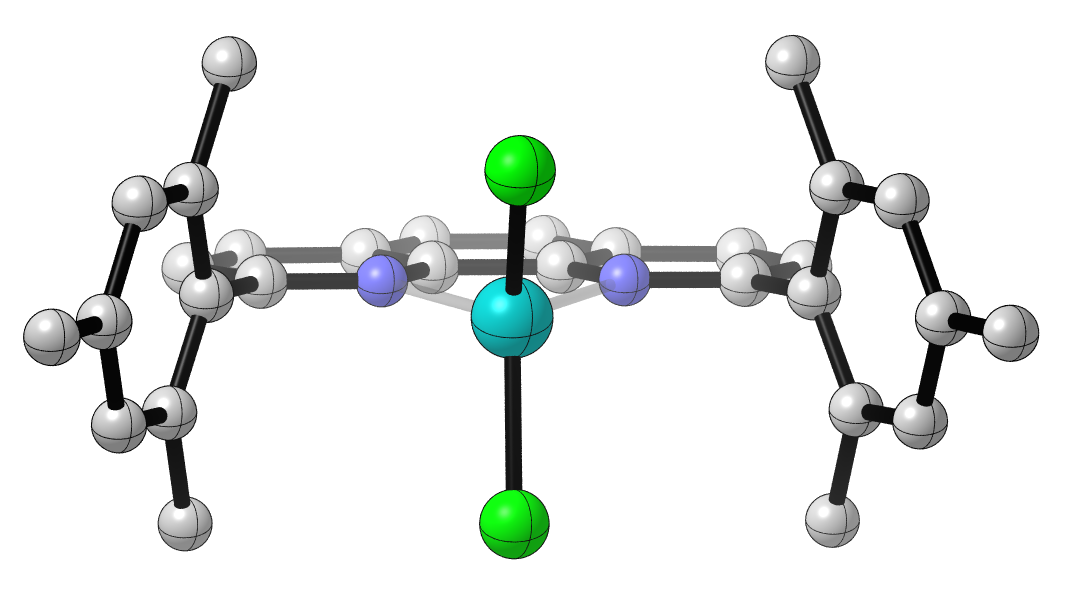^** | E = -3454.5768270 | 0.432196 |
| **^3^CC1**  **^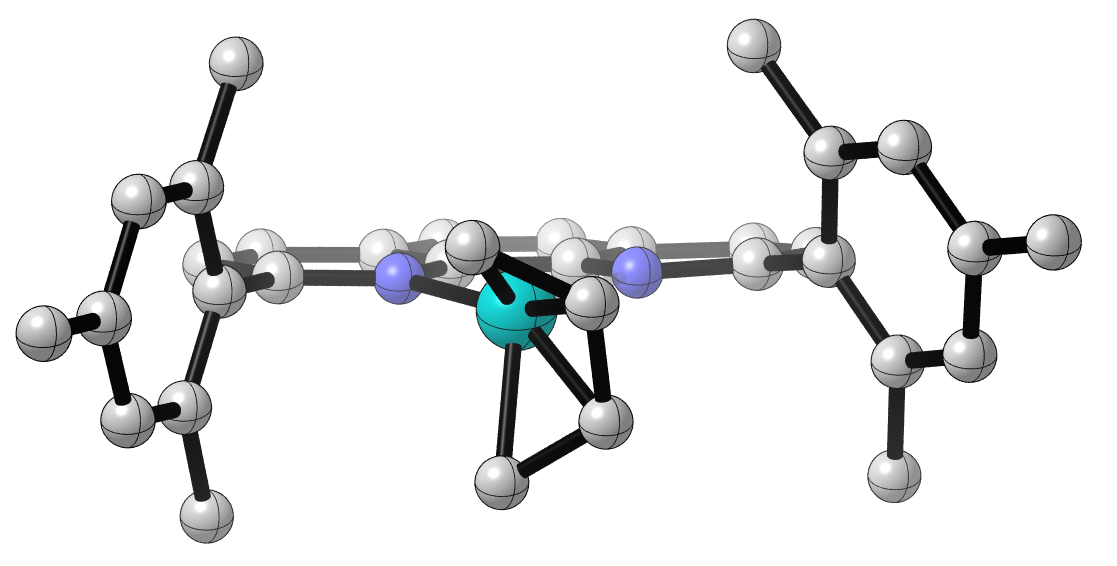^** | E = -2689.4619171 | 0.525877 |
| **^3^CC1**  (wB97XD/def2TZVPP) \|\| b3lyp-D3(BJ)/6-311g*)  **^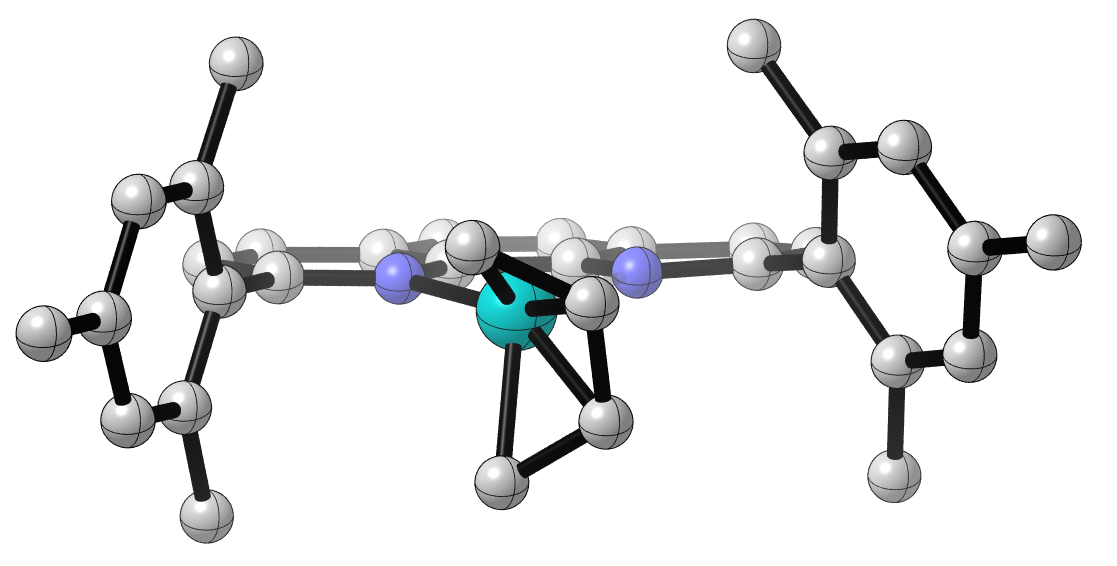^** | E = -2689.4383022 | 0.517460 |
| **^3^CC1**  (wB97XD/def2TZVPP-CPCM(THF) \|\| b3lyp-D3(BJ)/6-311g*) | E = -2689.4497429 | 0.517460 |
| **^3^CC1**  (PBE0/def2TZVPP-CPCM(THF) \|\| wB97XD/6-31g*-TZVP(Fe)) | E = -2689.4555312 | 0.513220 |
| **^3^CC1**  (M062x**/**def2TZVPP-CPCM(THF)) \|\| wB97XD/6-31g*-TZVP(Fe)) | E = -2689.2609010 | 0.525877 |
| **^3^CC1**  (TPSSh-D3(BJ)**/**def2TZVPP-CPCM(THF) \|\| TPSSh/6-31g*-TZVP(Fe)) | E = -2690.1755991 | 0.513220 |
| **^3^CC1**  (wB97XD**/**def2TZVPP-CPCM(THF) \|\| TPSSh/6-31g*-TZVP(Fe)) | E = -2689.4555312 | 0.513220 |
| **^5^CC1**  **^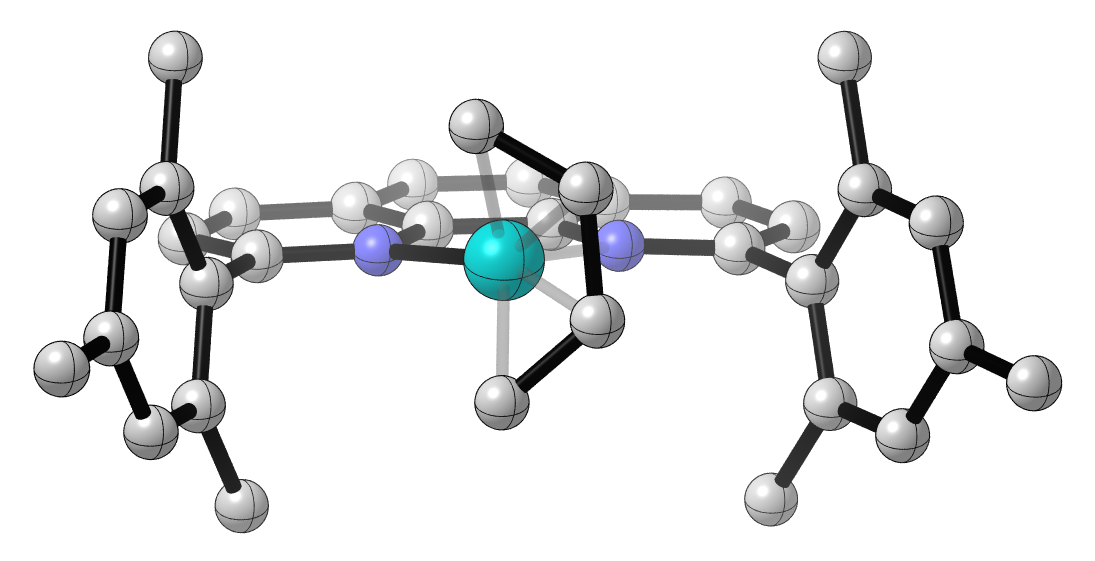^** | E = -2689.4542999 | 0.524475 |
| **^5^CC1**  (wB97XD/def2TZVPP) \|\| b3lyp-D3(BJ)/6-311g*) | E = -2689.4164785 | 0.513313 |
| **^5^CC1**  (wB97XD/def2TZVPP-CPCM(THF) \|\| b3lyp-D3(BJ)/6-311g*) | E = -2689.4269572 | 0.513313 |
| **^5^CC1**  (PBE0/def2TZVPP-CPCM(THF) \|\| wB97XD/6-31g*-TZVP(Fe)) | E = -2687.9645001 | 0.524475 |
| **^5^CC1**  (M062x**/**def2TZVPP-CPCM(THF)) \|\| wB97XD/6-31g*-TZVP(Fe)) | E = -2689.2573527 | 0.524475 |
| **^5^CC1**  (TPSSh-D3(BJ)**/**def2TZVPP-CPCM(THF) \|\| TPSSh/6-31g*-TZVP(Fe)) | E = -2690.1668860 | 0.512207 |
| **^5^CC1**  (wB97XD**/**def2TZVPP-CPCM(THF) \|\| TPSSh/6-31g*-TZVP(Fe)) | E = -2689.4492399 | 0.512207 |

**Table S16** Calculated Corrected Gibbs free energy and electronic energy of all the stationary points and MECP. H atoms were omitted for clarity except for some particular structures.

| **Structures** | **Electronic energy/ Hartree** | **Thermal correction to Gibbs Free Energy (ΔG_corr_.)/ Hartree** |
| --- | --- | --- |
| **Propyne** | E = -116.659585 | 0.033196 |
| **PhSiH_3_** | E = -522.950714 | 0.084772 |
| **3a** | E = -639.681029 | 0.143447 |
| 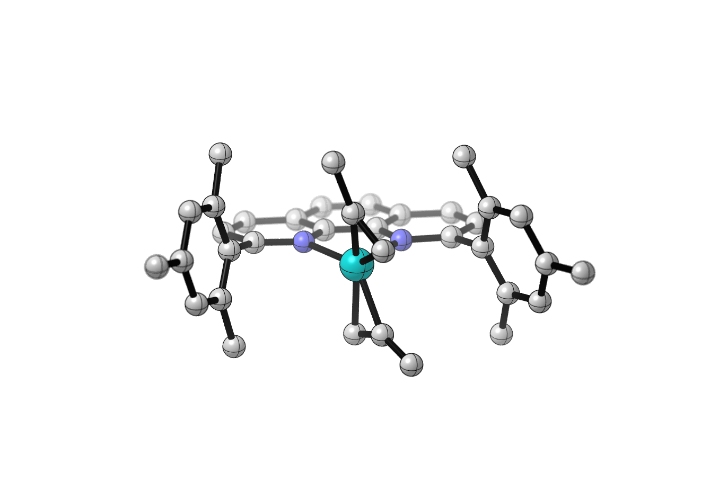**^1^Int-1** | E = -2766.767745 | 0.558970 |
| 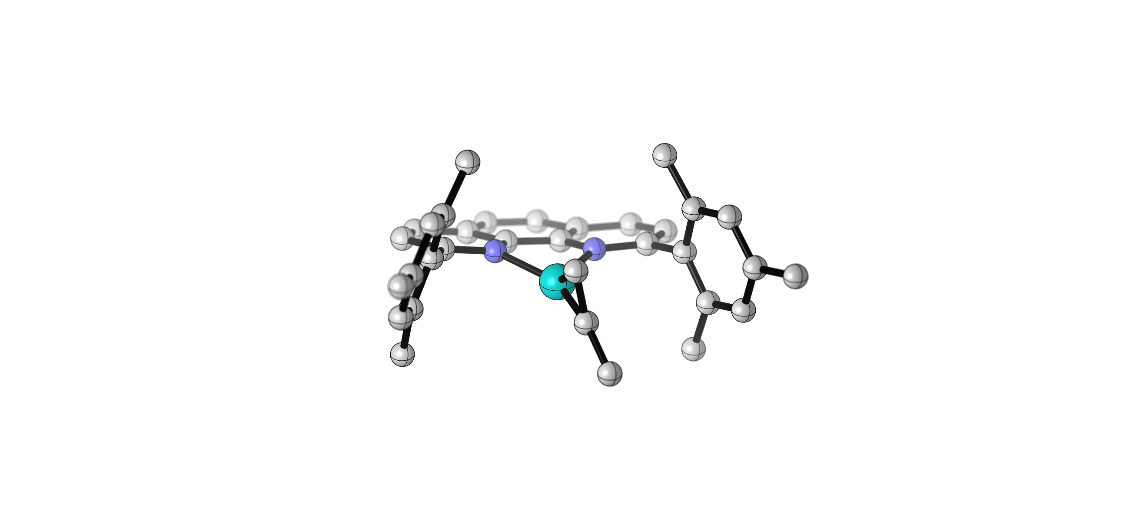**^1^Int-1ʹ** | E = -2650.065143 | 0.496674 |
| 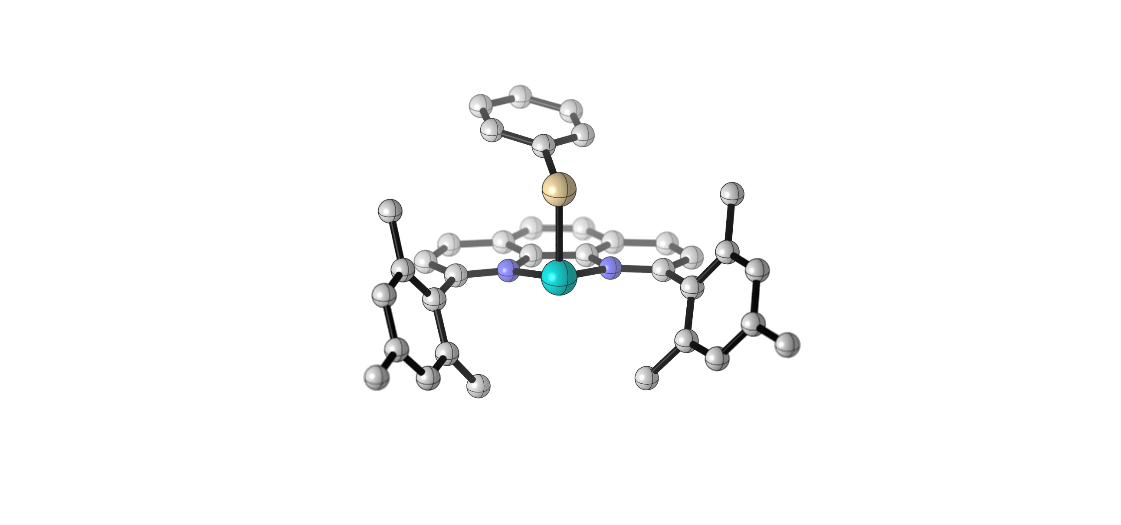**^1^Int-1ʺ** | E = -3056.347960 | 0.555251 |
| 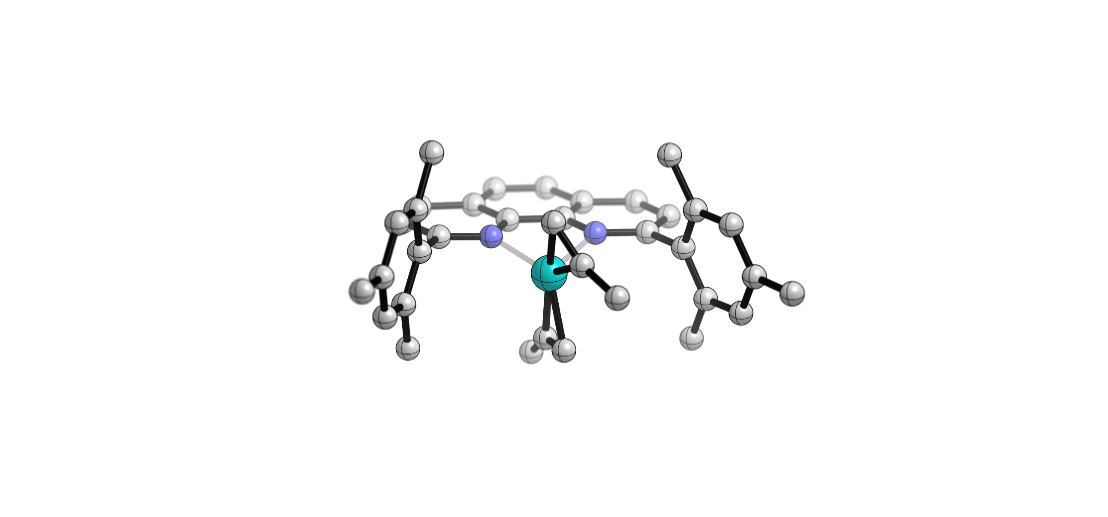**^3^Int-1** | E = -2766.805789 | 0.551784 |
| 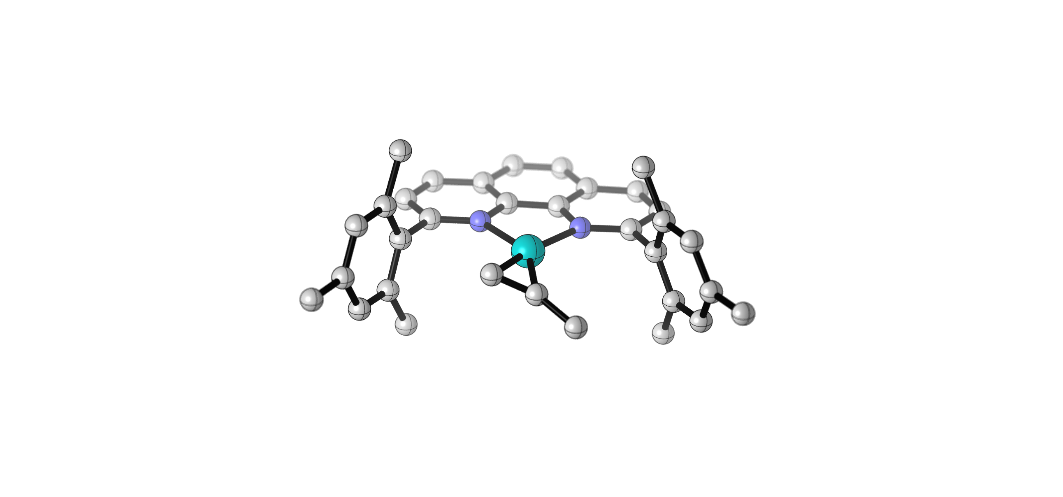**^3^Int-1ʹ** | E = -2650.057602 | 0.495453 |
| 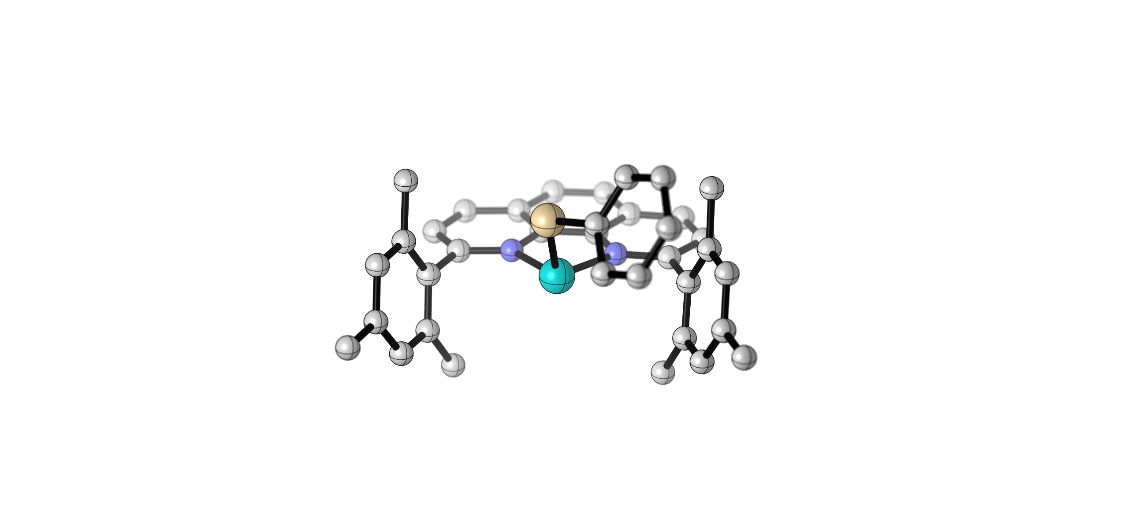**^3^Int-1ʺ** | E = -3056.383604 | 0.550466 |
| 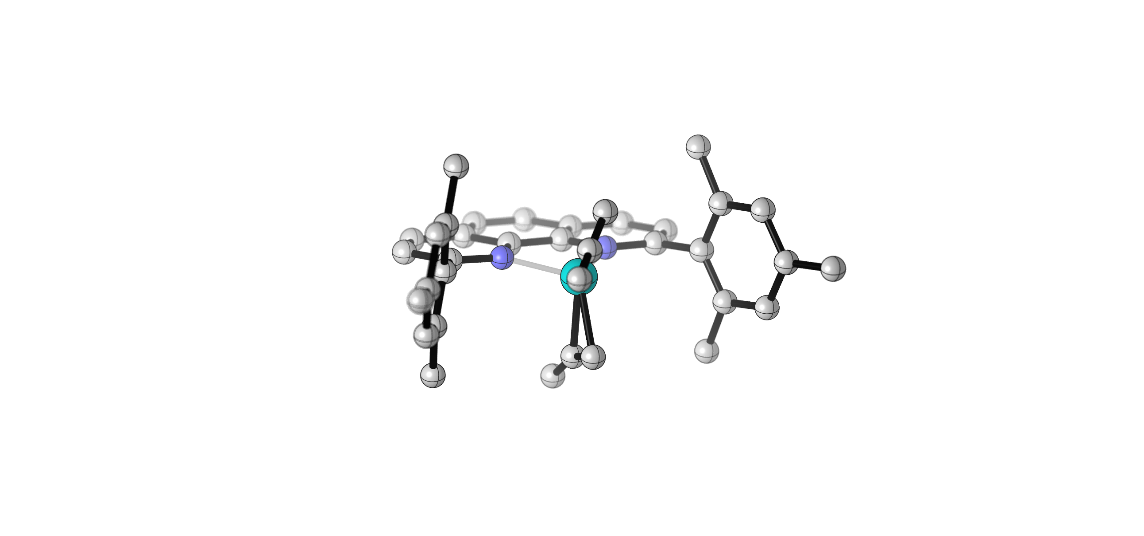**^5^Int-1** | E = -2766.792138 | 0.550980 |
| 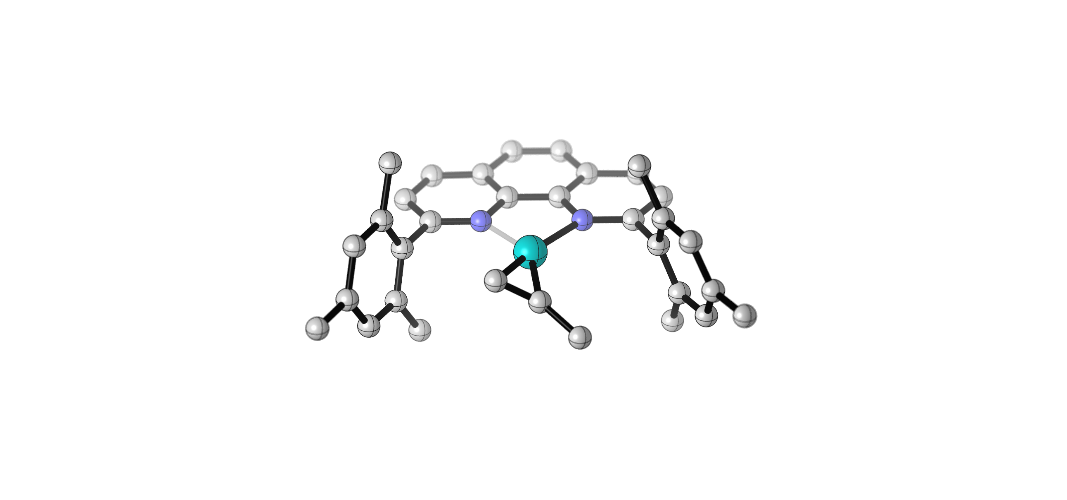**^5^Int-1ʹ** | E = -2650.113200 | 0.494023 |
| 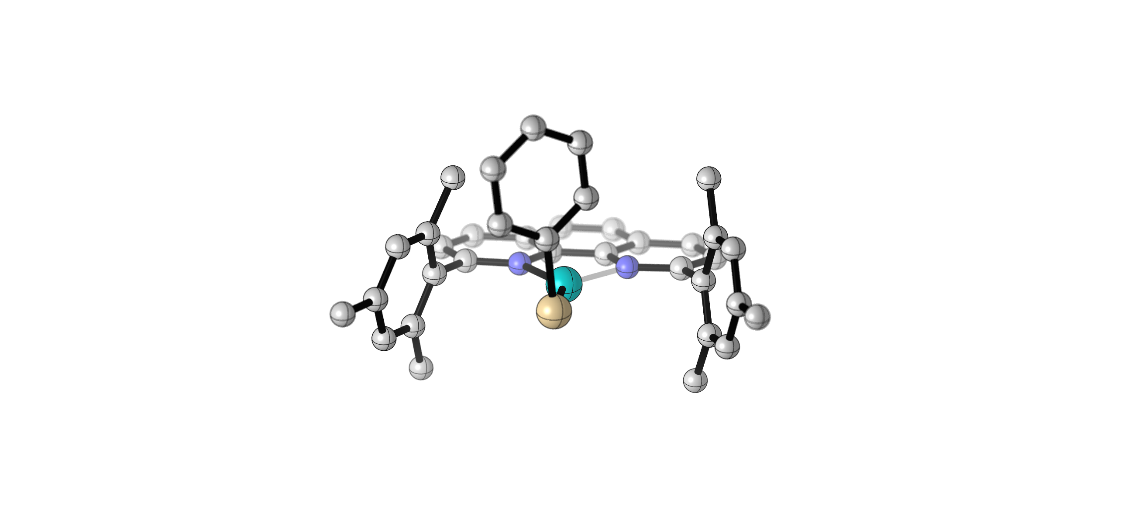**^5^Int-1ʺ** | E = -3056.377824 | 0.547831 |
| 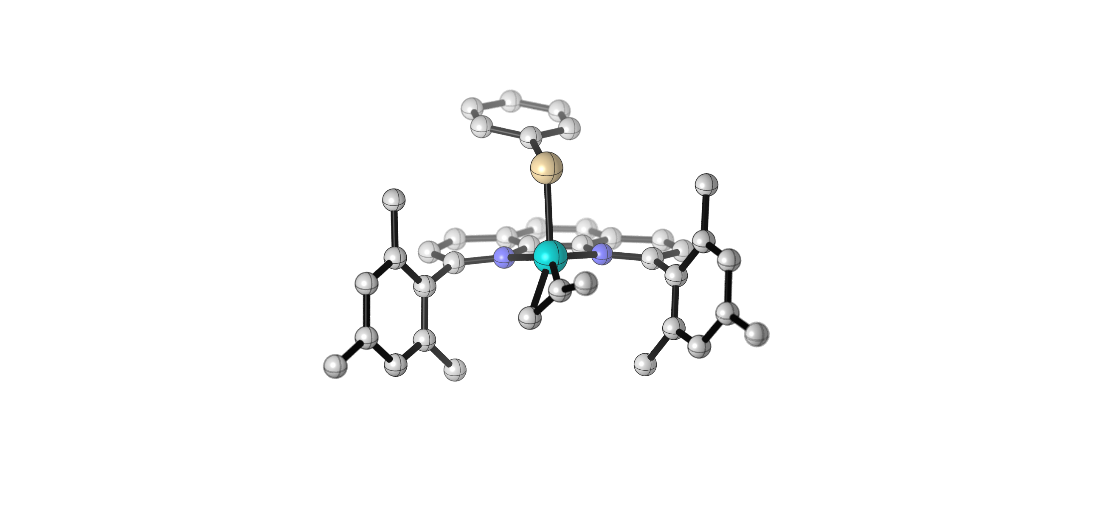**^1^Int-2** | E = -3173.062905 | 0.612003 |
| 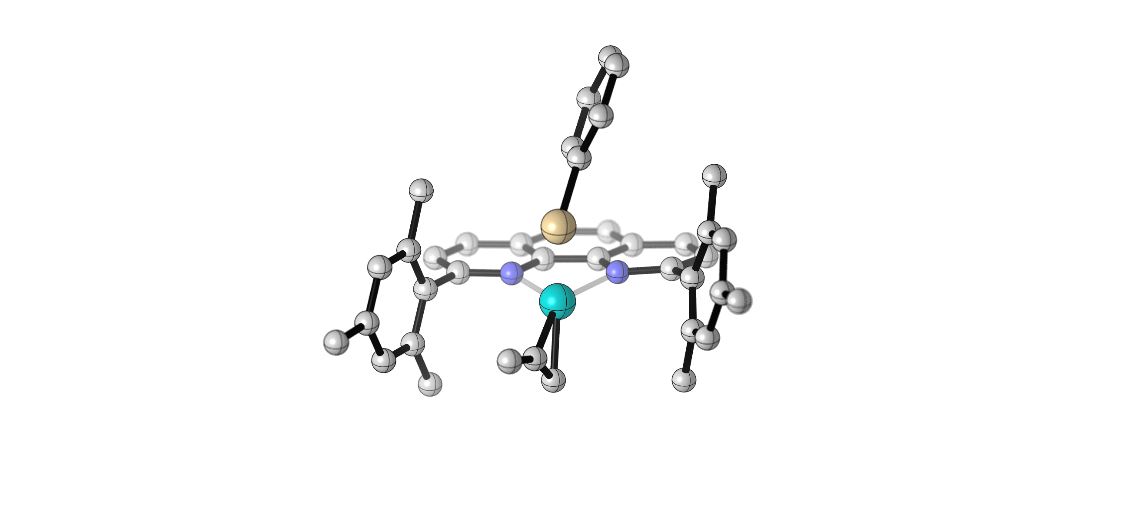**^3^Int-2** | E = -3173.085985 | 0.606282 |
| 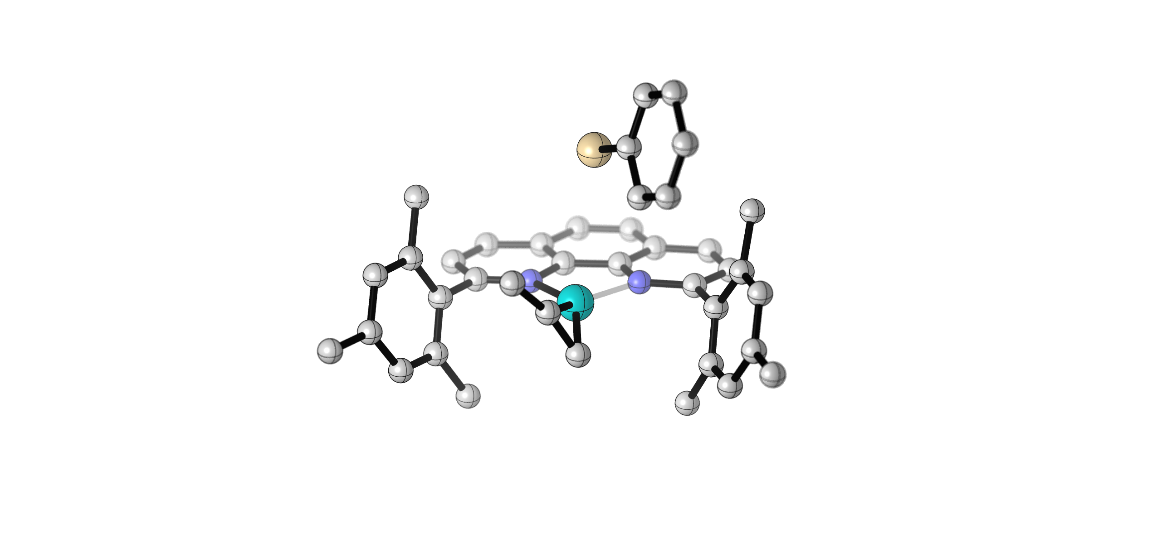**^5^Int-2** | E = -3173.078876 | 0.603810 |
| 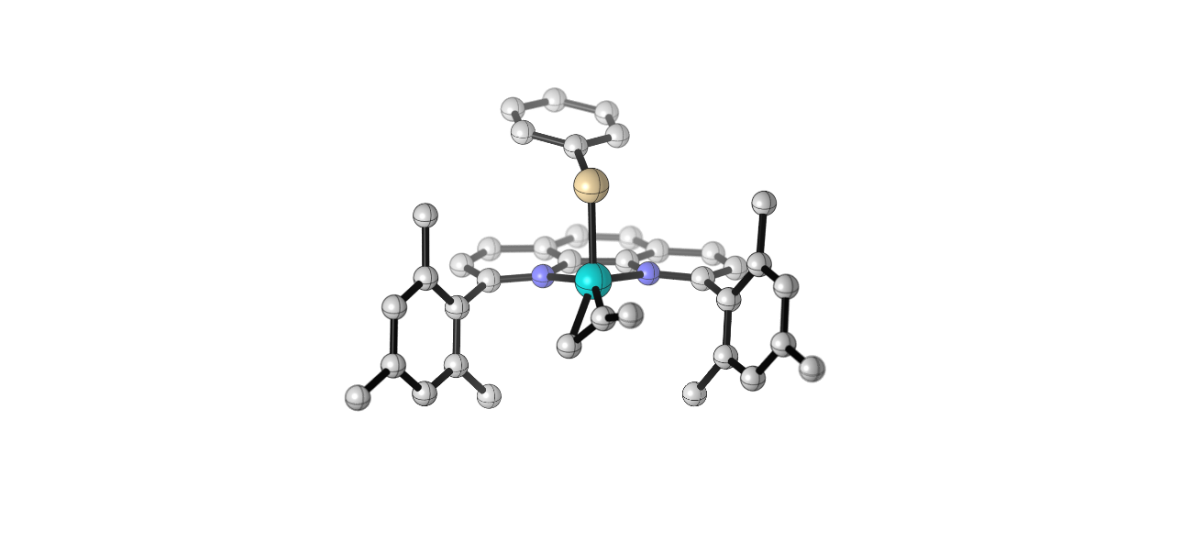**^1^TS-1** | E = -3173.062534 | 0.613636 |
| 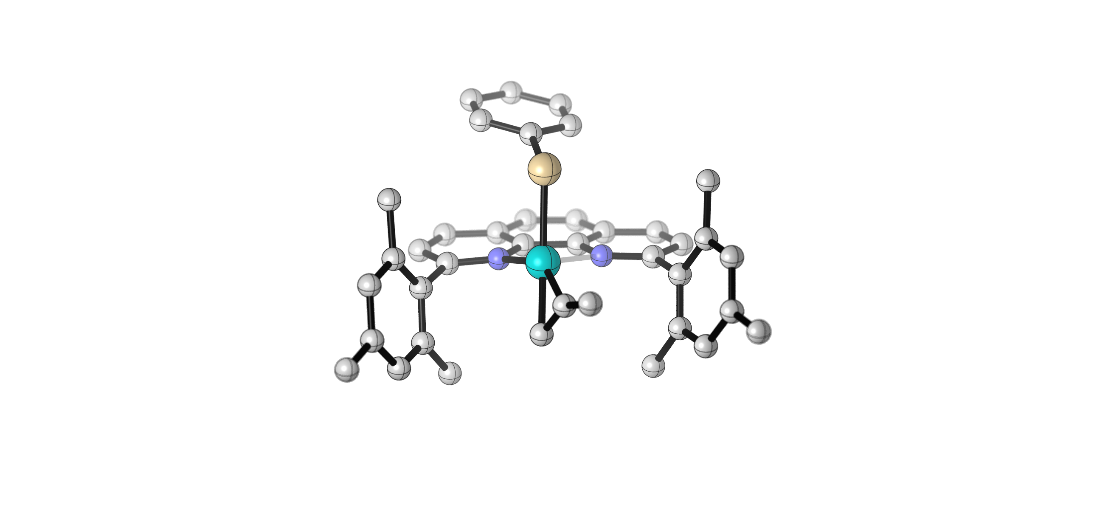**^3^TS-1** | E = -3173.075015 | 0.606851 |
| **^3^TS-1a**  **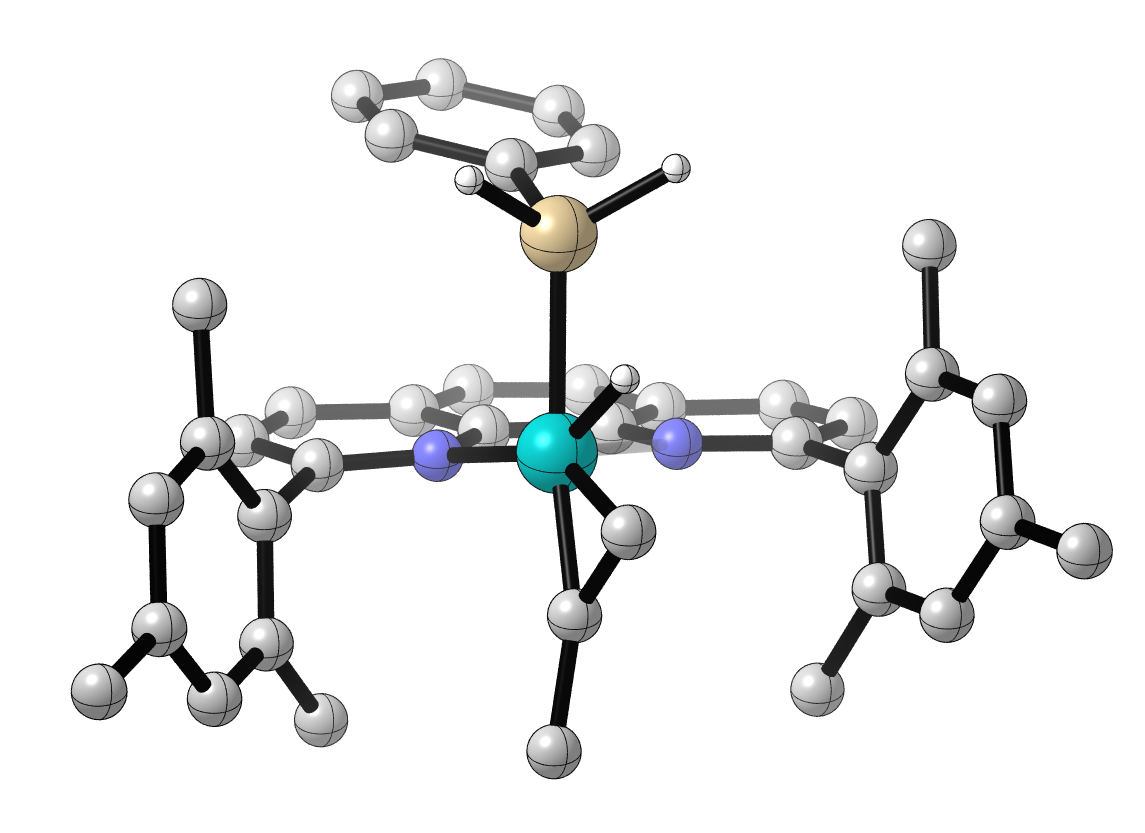** | E = -3173.071577 | 0.608934 |
| 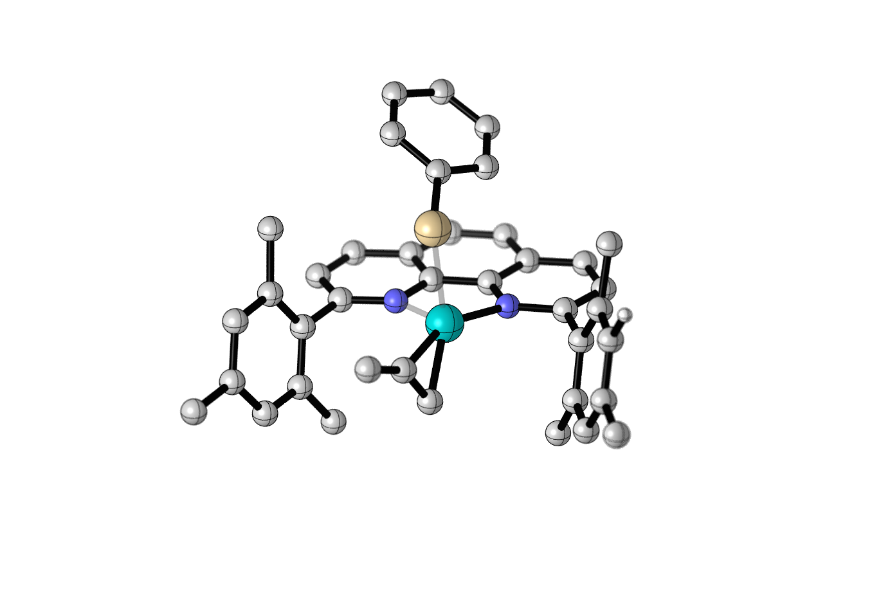**^5^TS-1** | E = -3173.053971 | 0.603202 |
| 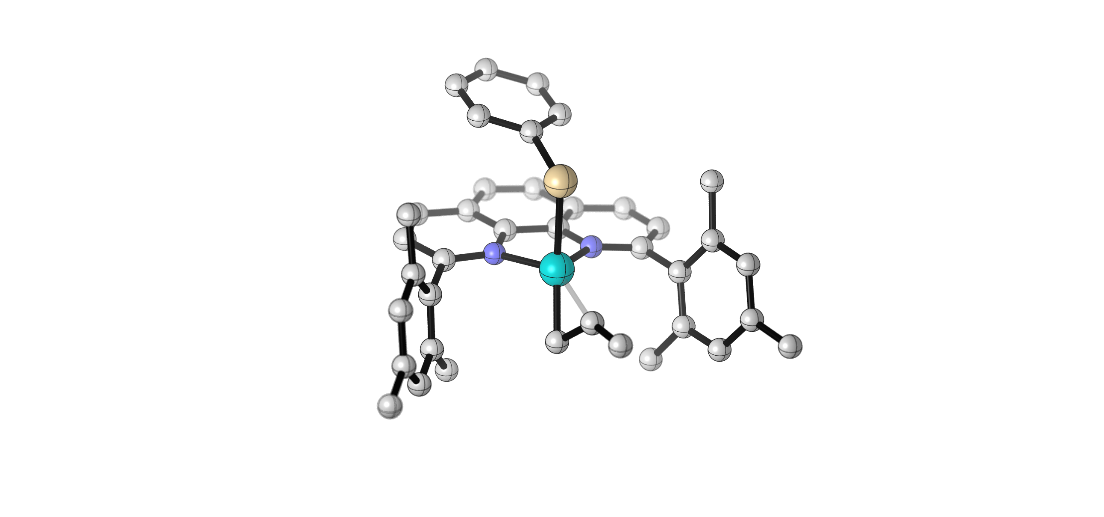**^1^Int-3ʹ** | E = -3173.075001 | 0.615904 |
| 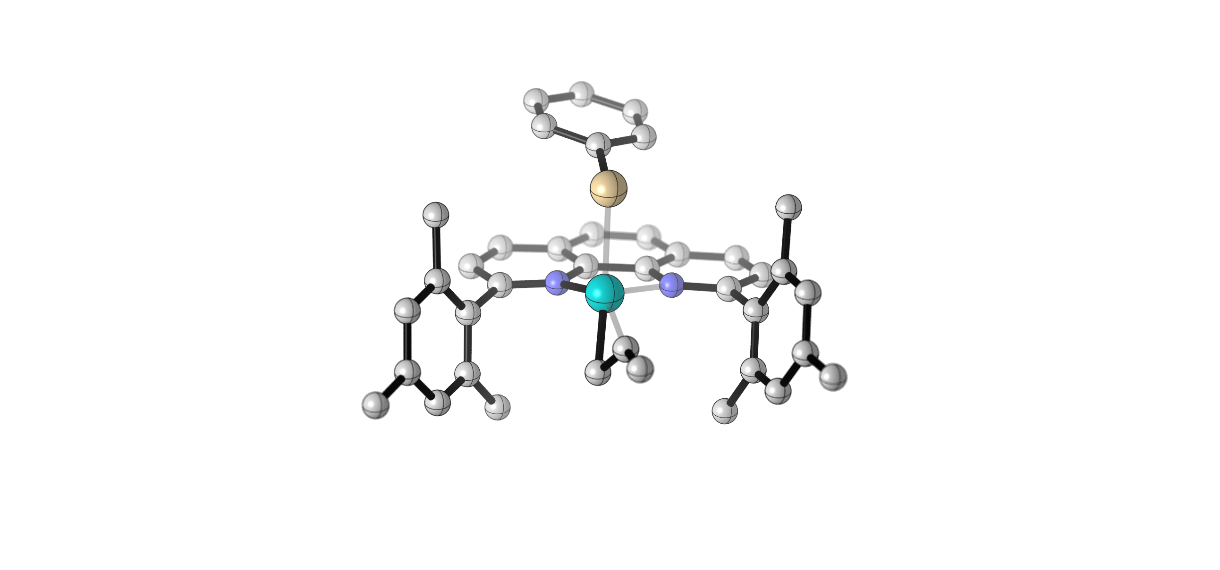**^3^Int-3ʹ** | E = -3173.089052 | 0.612112 |
| 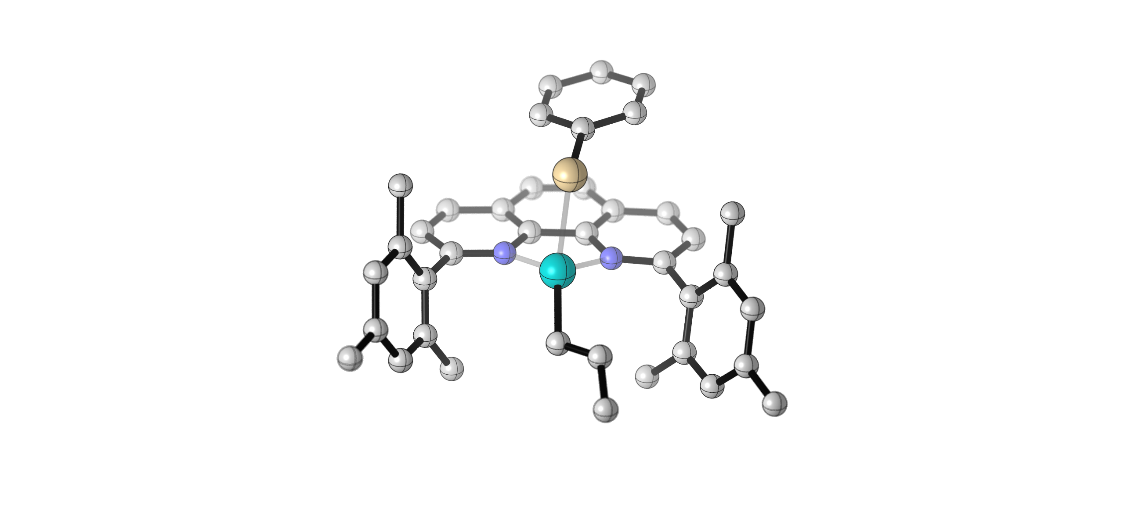**^5^Int-3** | E = -3173.122278 | 0.608580 |
| **^1^TS-2**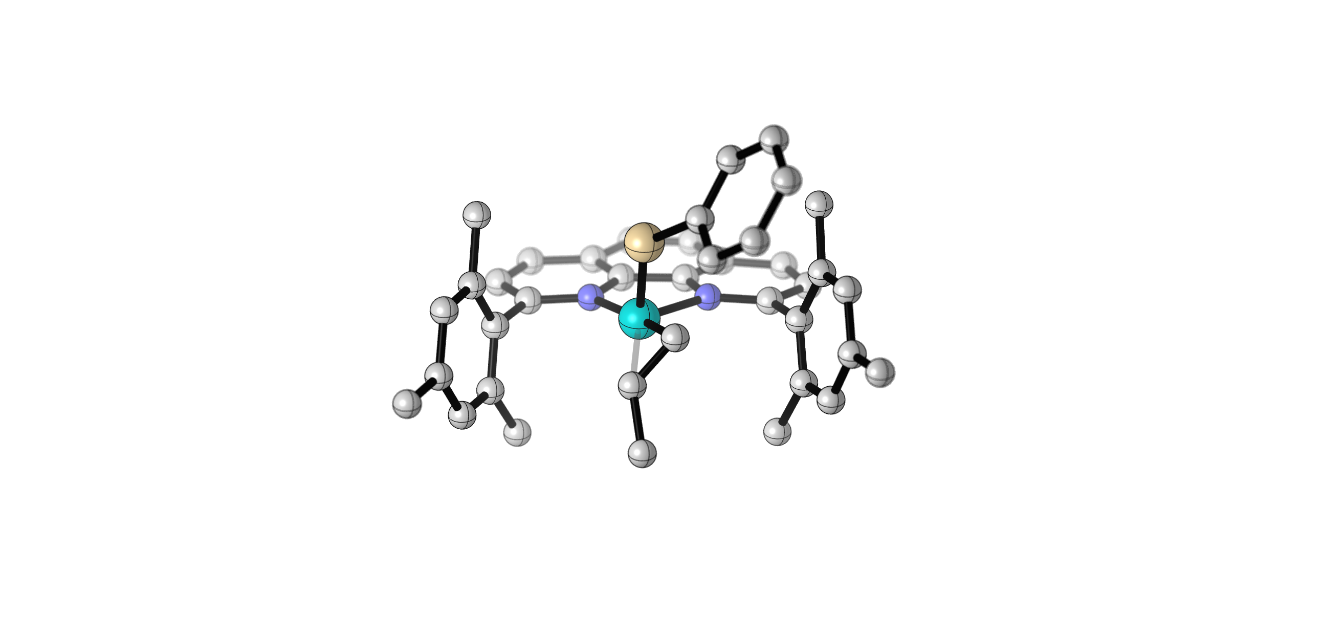 | E = -3173.048934 | 0.615895 |
| **^3^TS-2**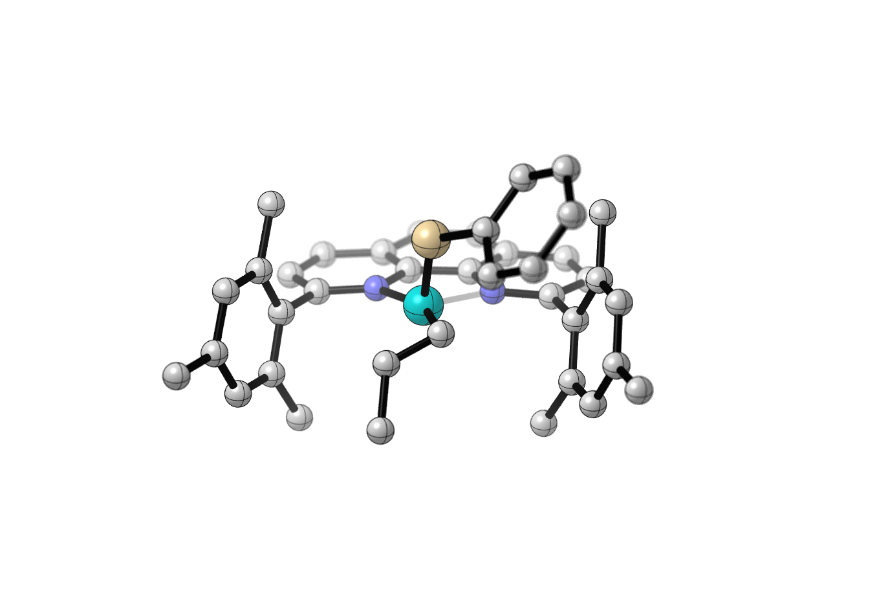 | E = -3173.066384 | 0.612420 |
| **^5^TS-2**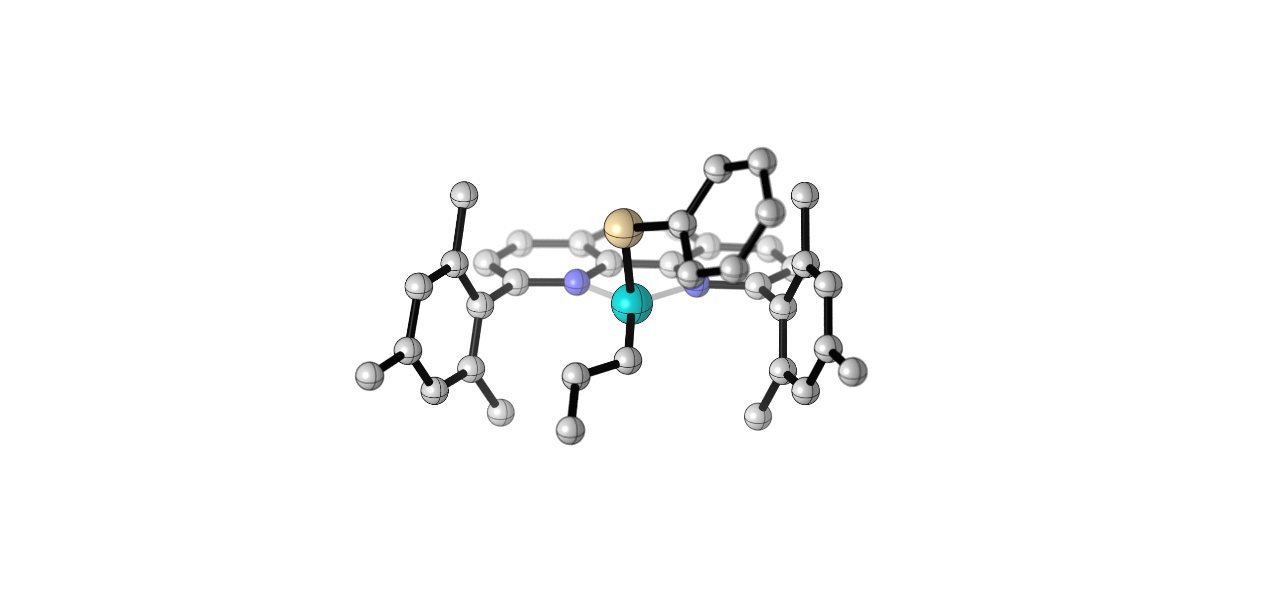 | E = -3173.102160 | 0.610600 |
| **^1^Int-4**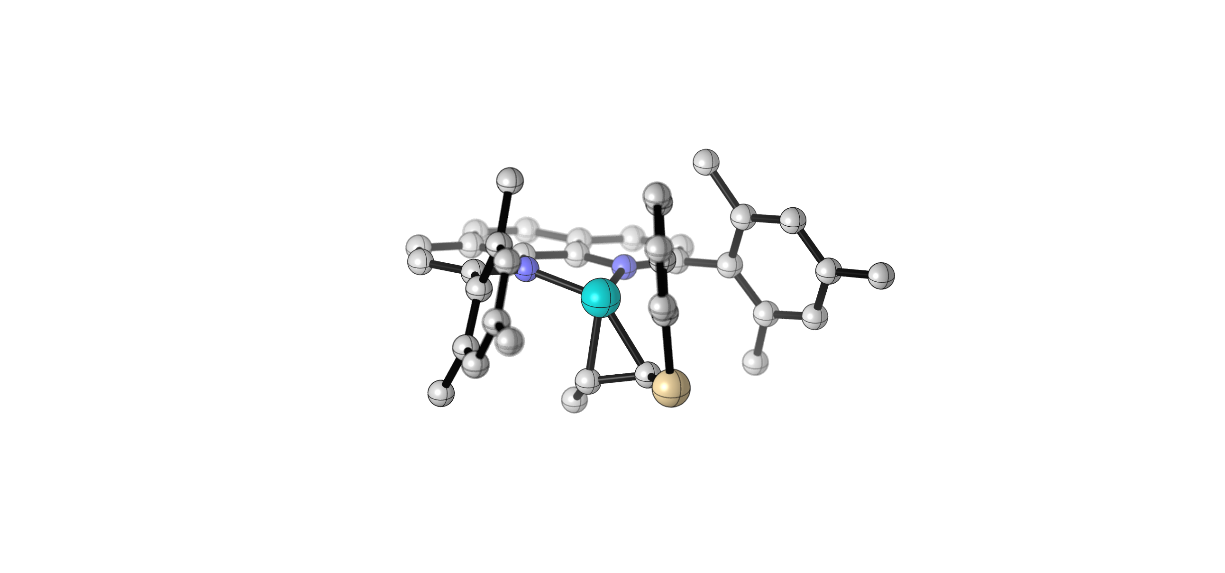 | E = -3173.110436 | 0.614487 |
| **^3^Int-4**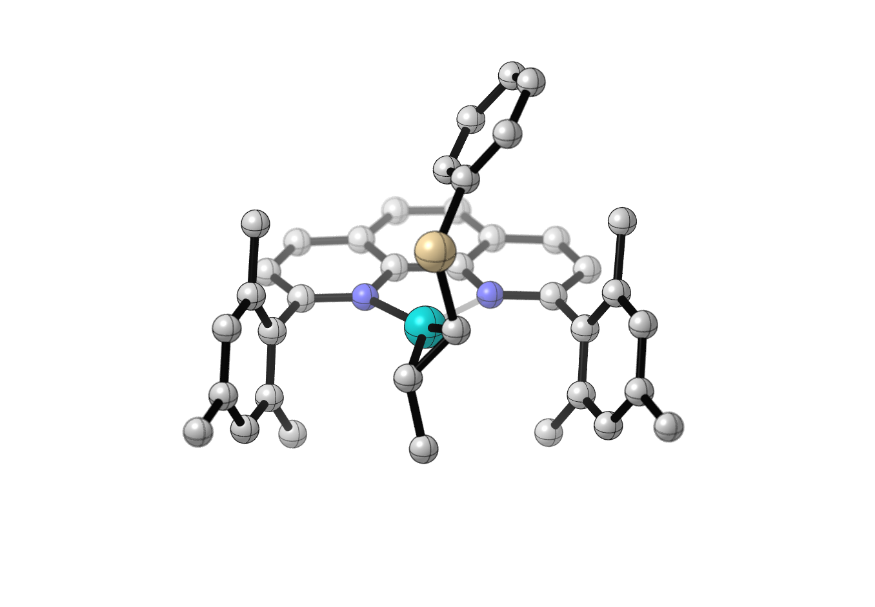 | E = -3173.101411 | 0.611783 |
| **^5^Int-4**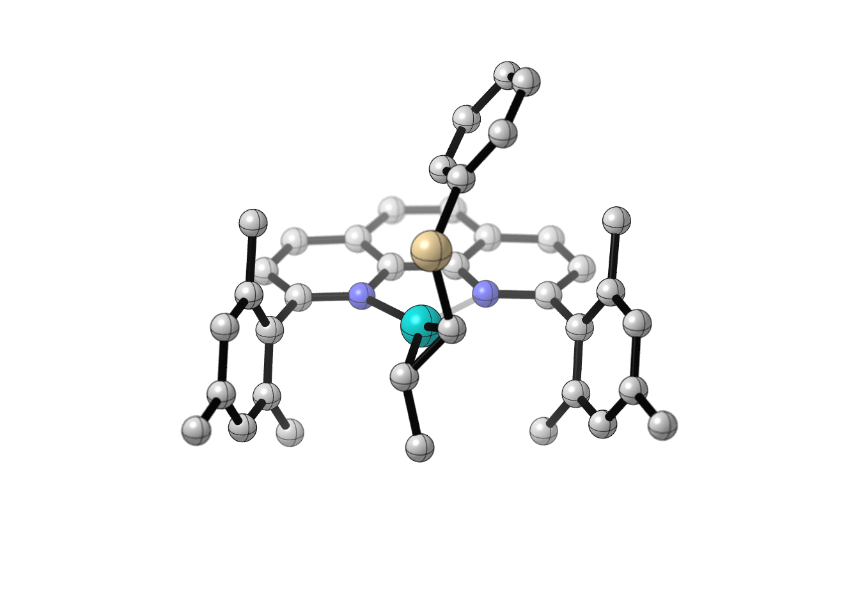 | E = -3173.145345 | 0.612524 |
| **M-^1^Int-1ʹʹ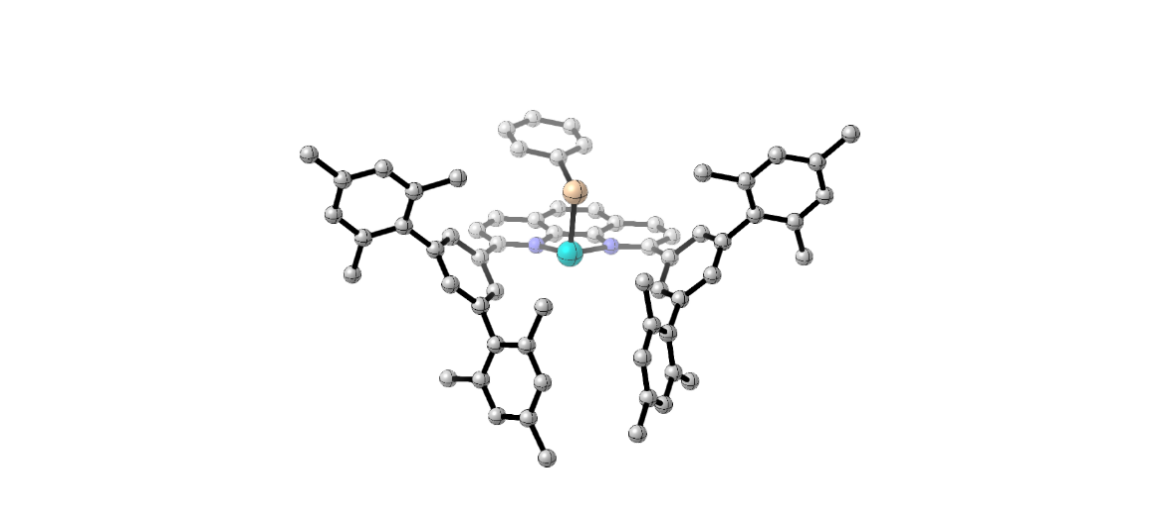** | E = -4216.511073 | 1.013247 |
| **M-^3^Int-1ʹʹ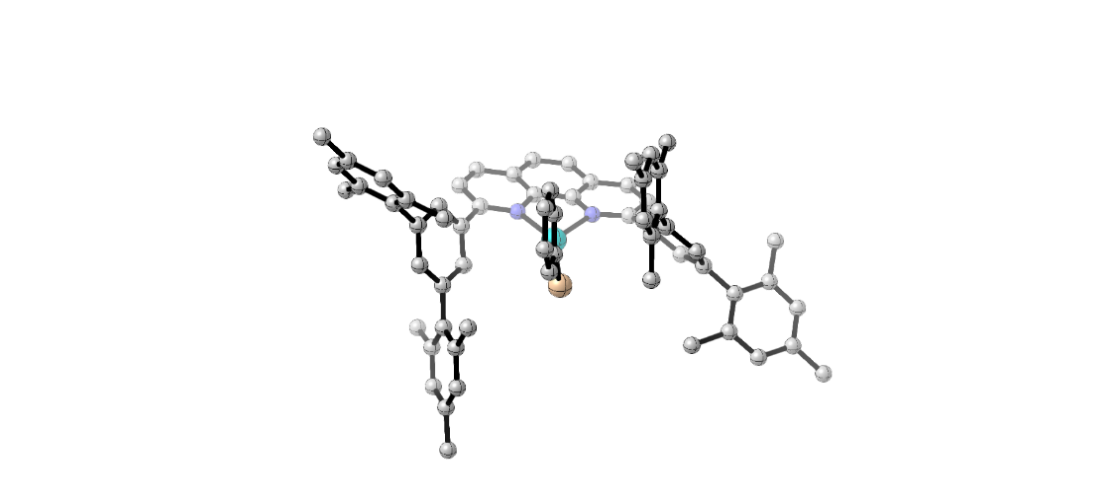** | E = -4216.489664 | 1.014190 |
| **M-^5^Int-1ʹʹ**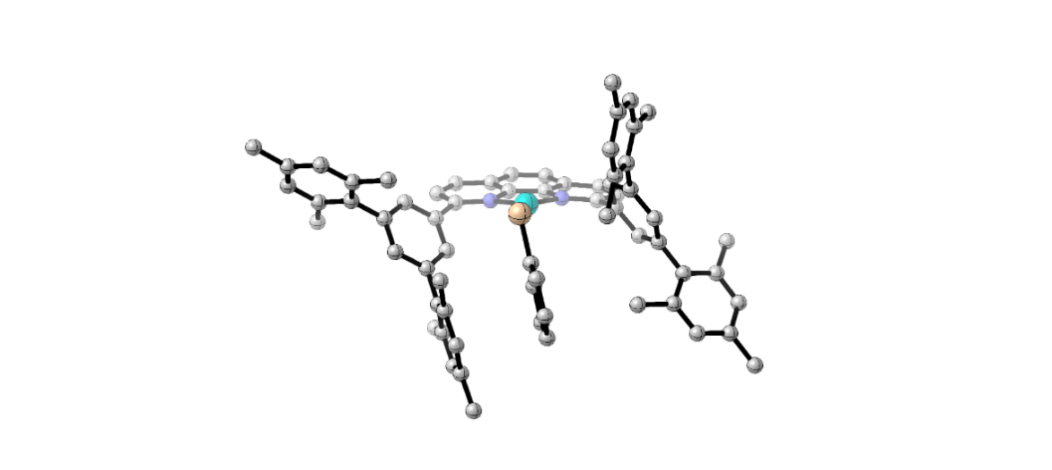 | E = -4216.532377 | 1.011919 |
| **M-^1^Int-1ʹ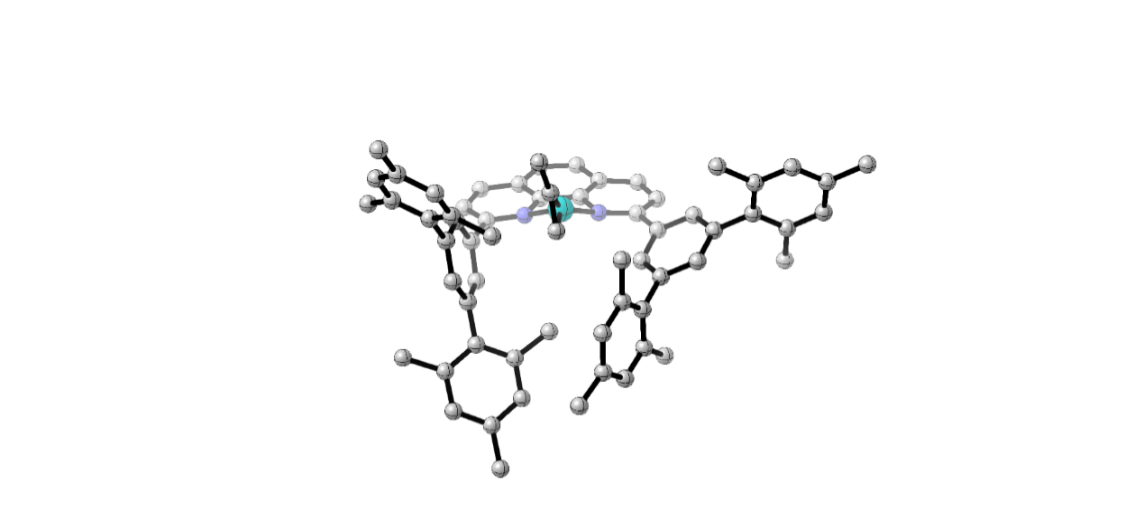** | E = -3810.222900 | 0.962186 |
| **M-^3^Int-1ʹ**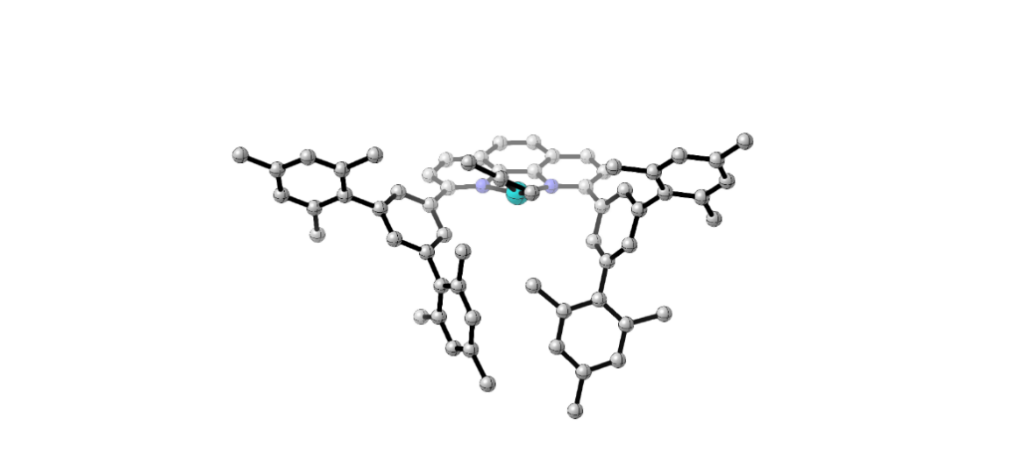 | E = -3810.283496 | 0.959114 |
| **M-^5^Int-1ʹ**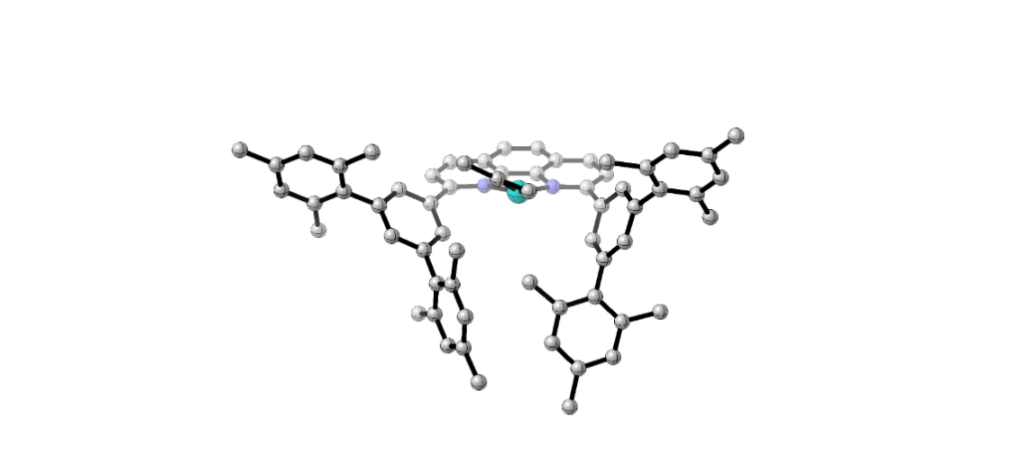 | E = -3810.261249 | 0.958468 |
| **M-^1^Int-1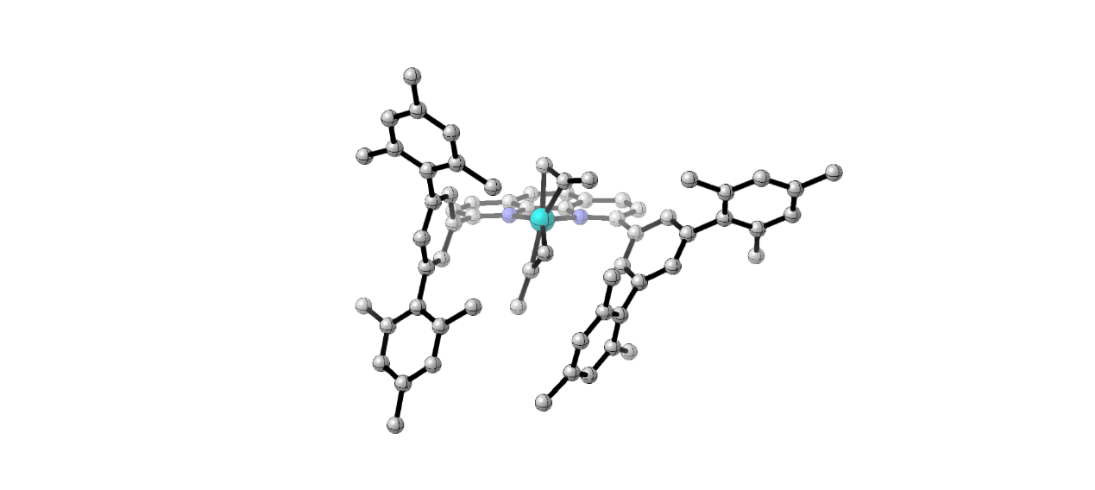** | E = -3926.926280 | 1.020295 |
| **M-^3^Int-1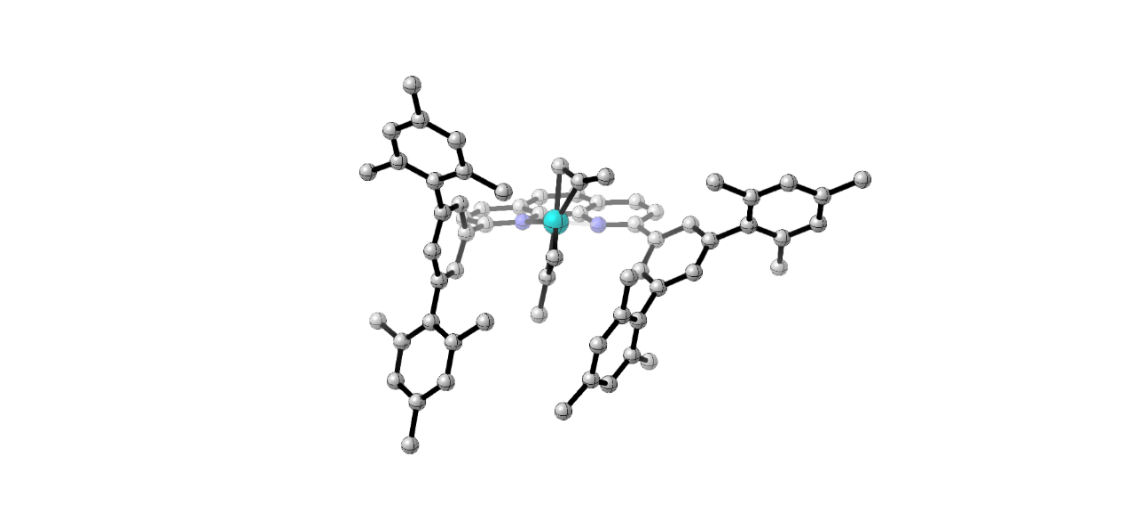** | E = -3925.908638 | 1.016164 |
| **M-^5^Int-1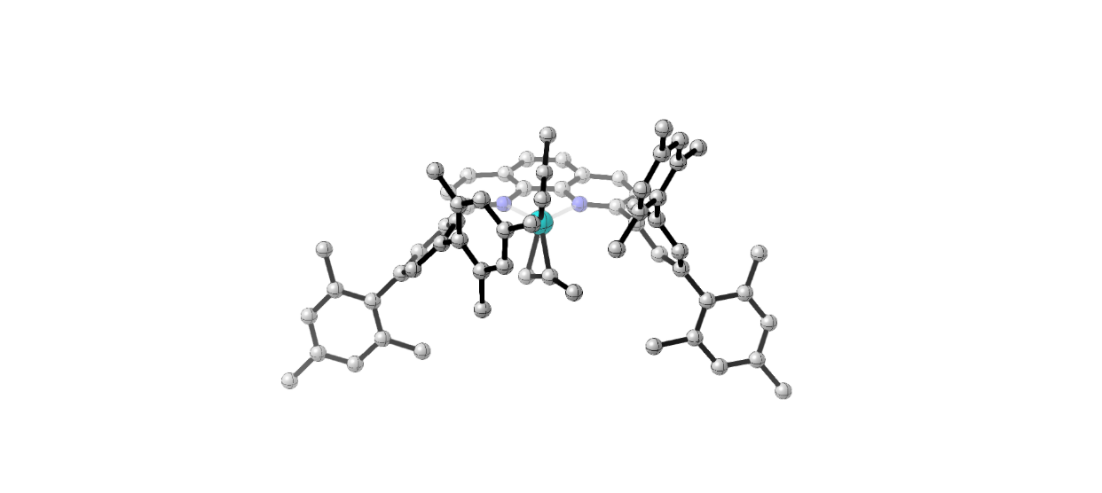** | E = -3926.950883 | 1.013496 |
| **M-^1^Int-2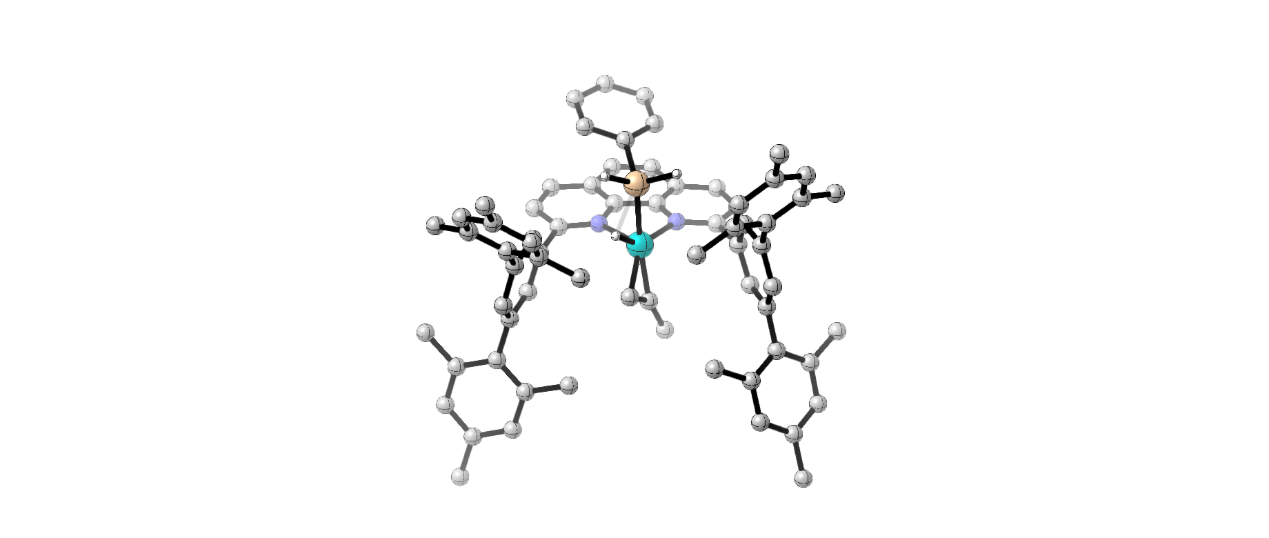** | E = -4333.222374 | 1.069447 |
| **M-^3^Int-2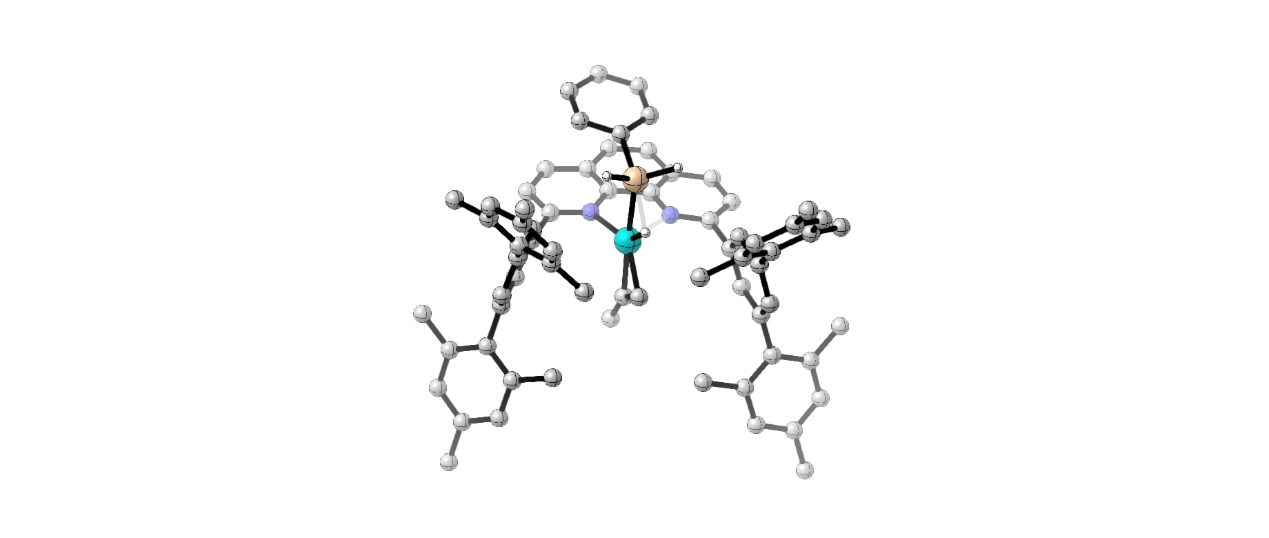** | E = -4333.241030 | 1.063088 |
| **M-^5^Int-2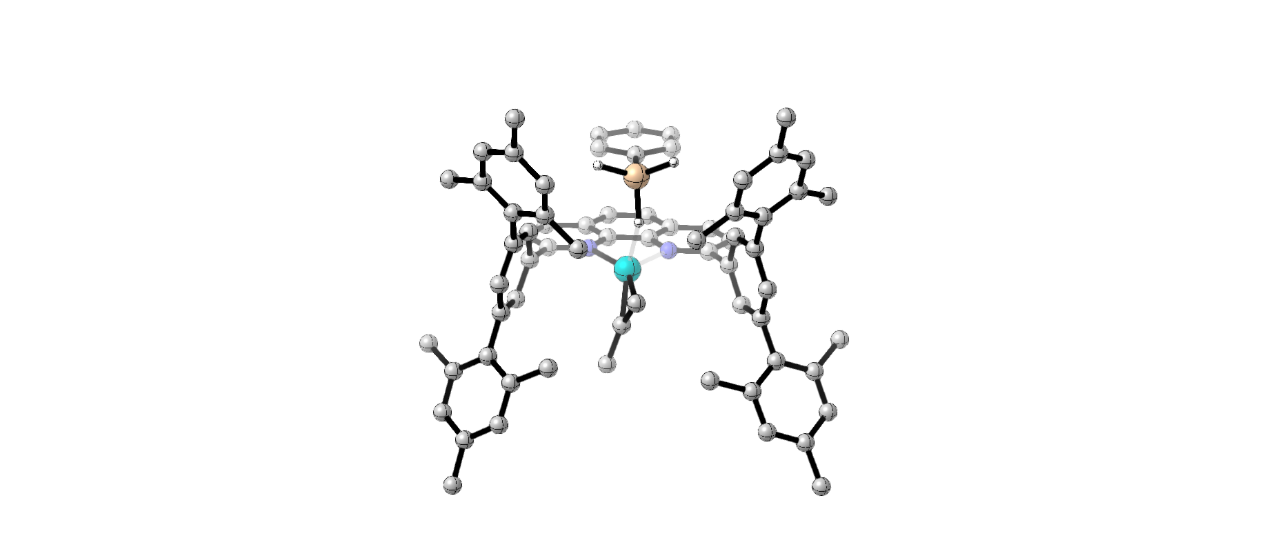** | E = -4333.236720 | 1.060493 |
| **M-^1^Ts-1^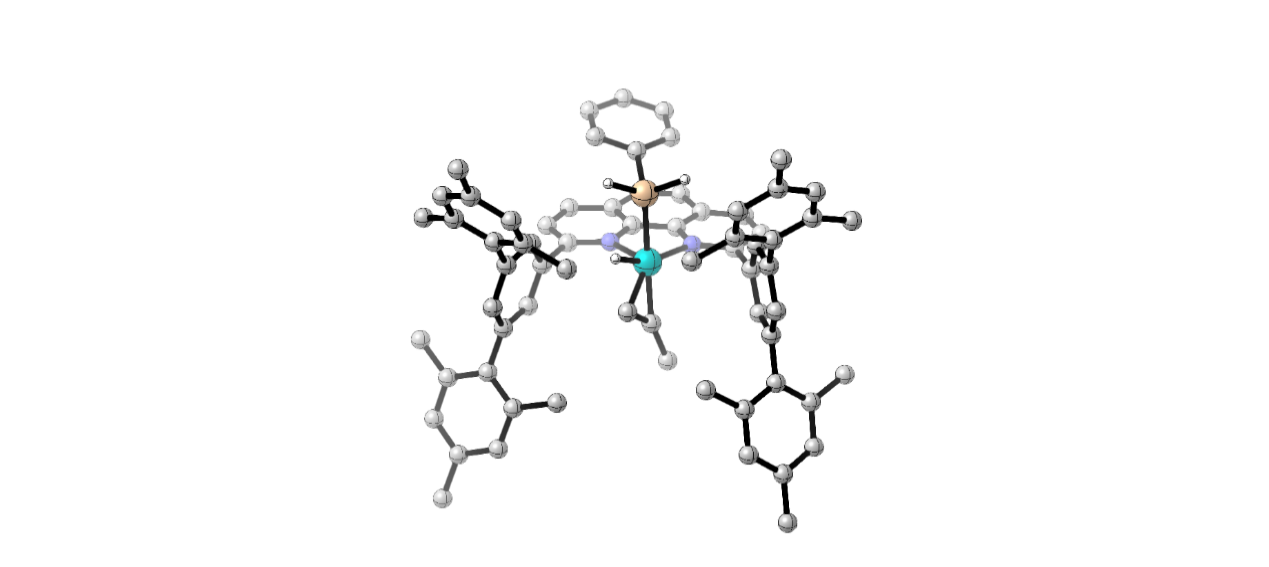^** | E = -4333.219924 | 1.067798 |
| **M-^3^Ts-1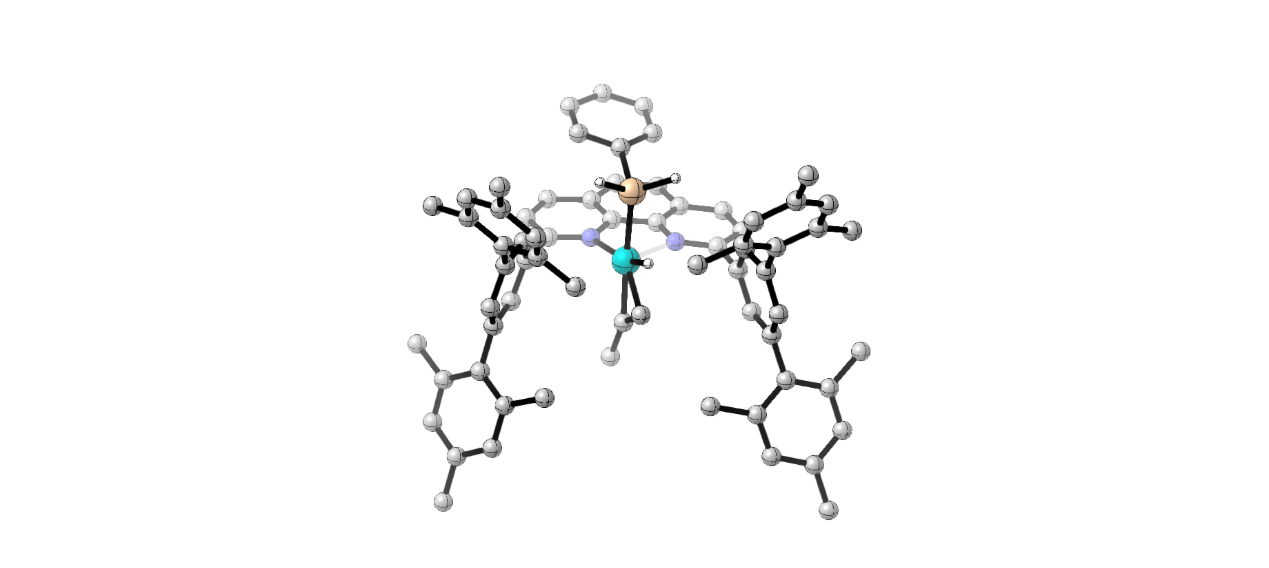** | E = -4333.235280 | 1.065214 |
| **M-^3^Ts-1a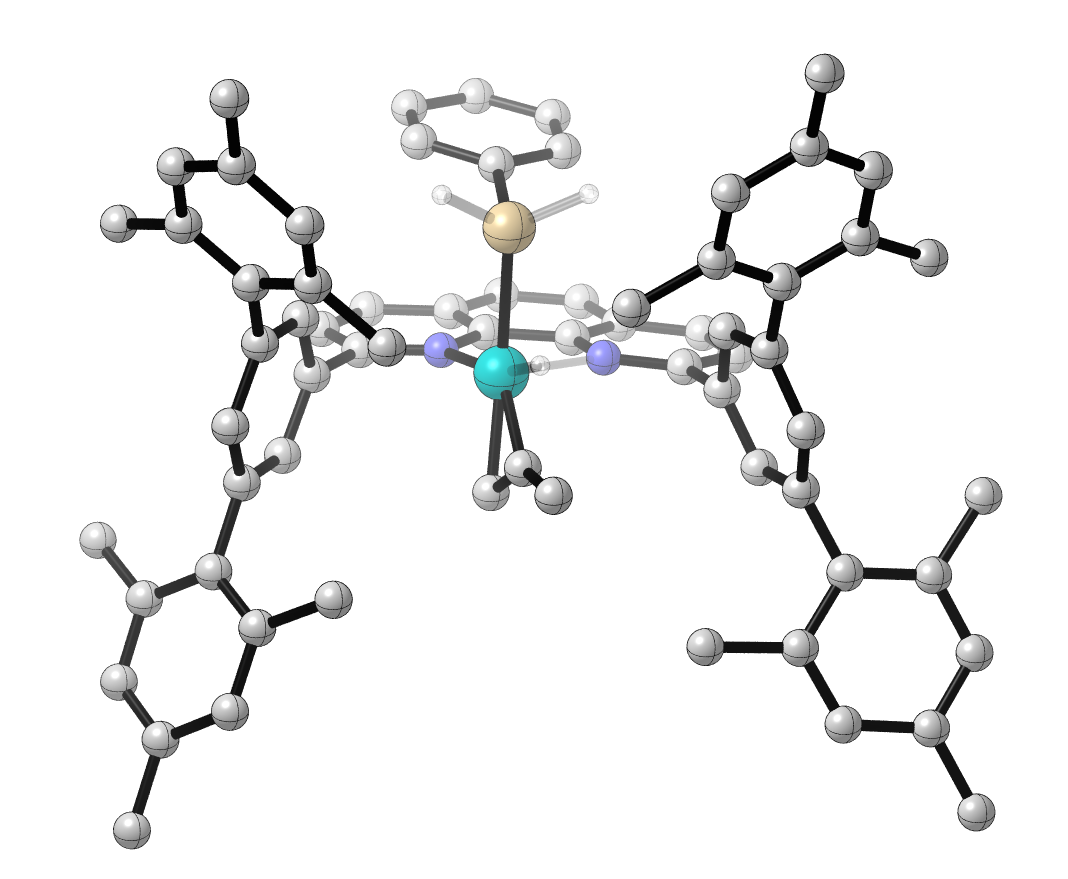** | E = -4333.234917 | 1.068882 |
| **M-^5^Ts-1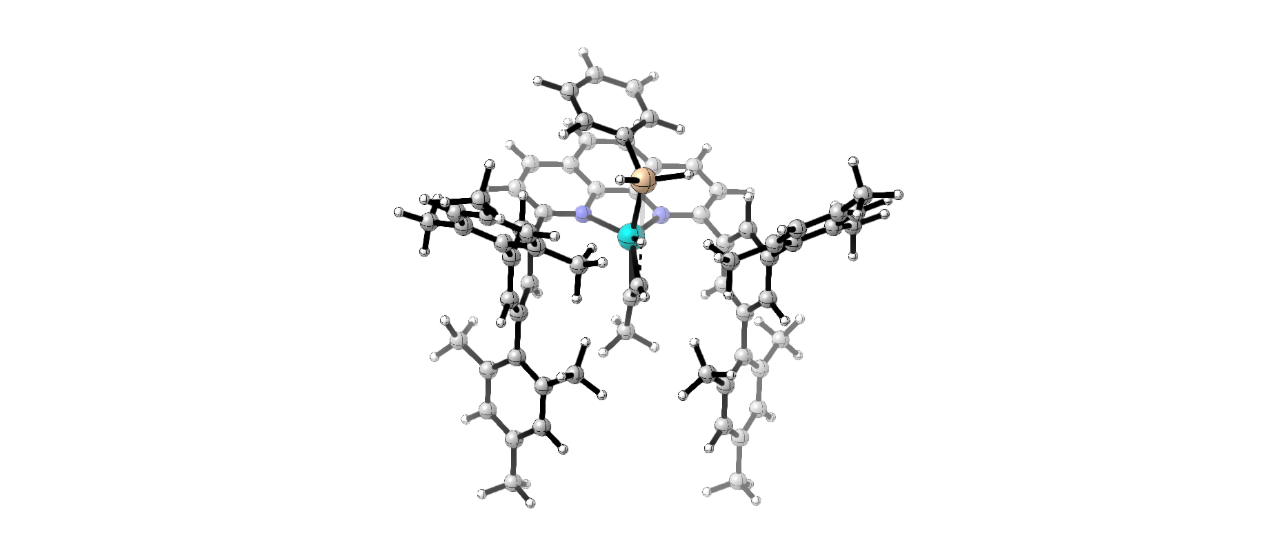** | E = -4333.205134 | 1.061594 |
| **M-^1^Int-3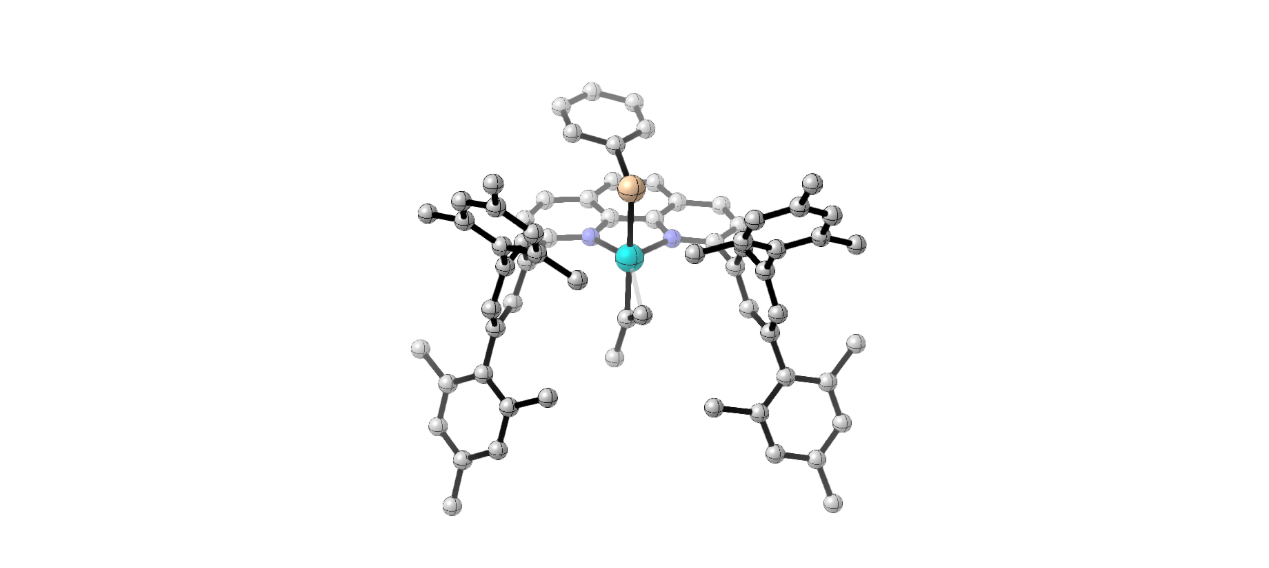** | E = -4333.237182 | 1.068136 |
| **M-^3^Int-3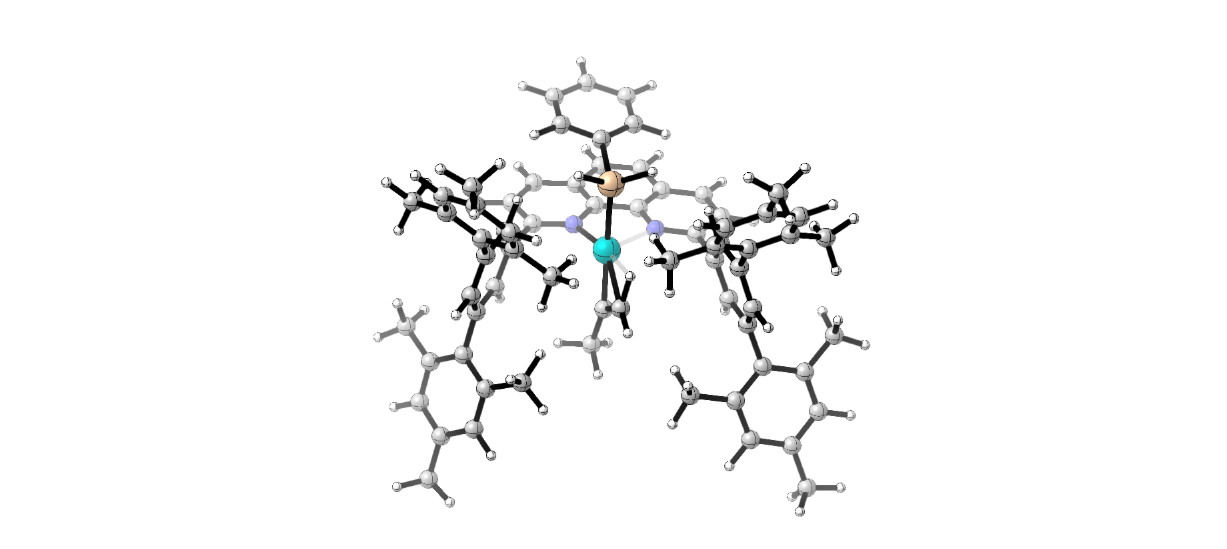** | E = -4333.251461 | 1.070706 |
| **M-^5^Int-3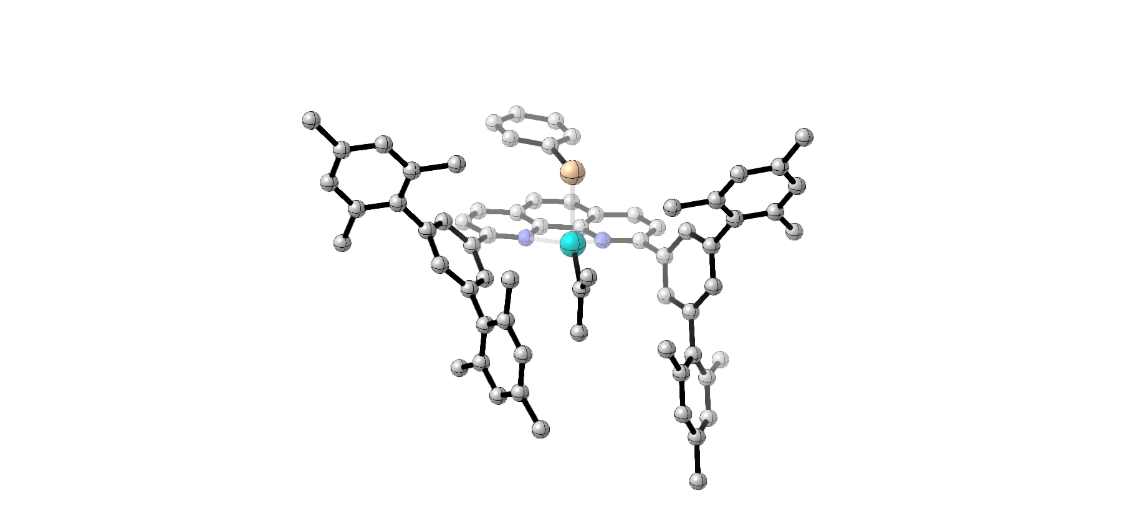** | E = -4333.277921 | 1.070541 |
| **M-^5^Ts-2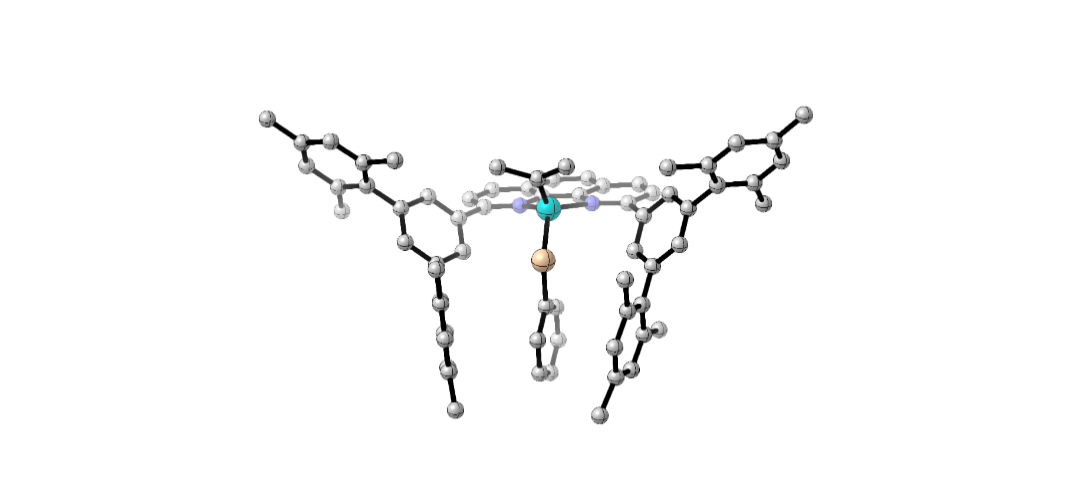** | E = -4333.255801 | 1.072375 |
| **M-^1^Int-4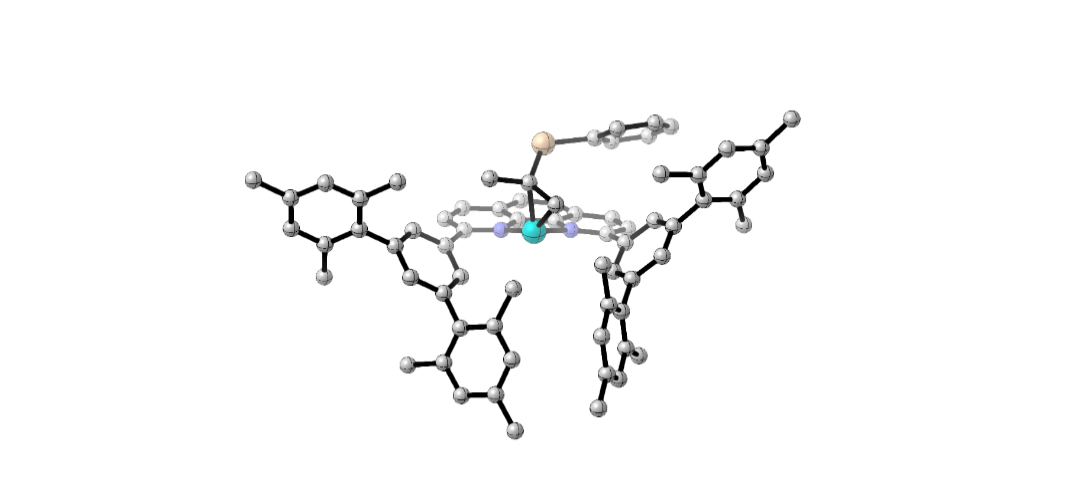** | E = -4333.248237 | 1.081405 |
| **M-^3^Int-4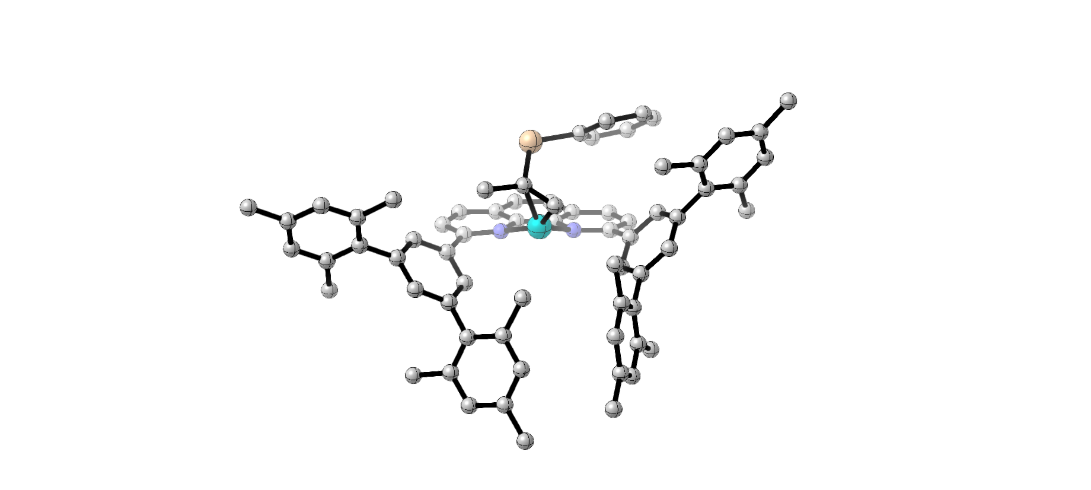** | E = -4333.305770 | 1.076613 |
| **M-^5^Int-4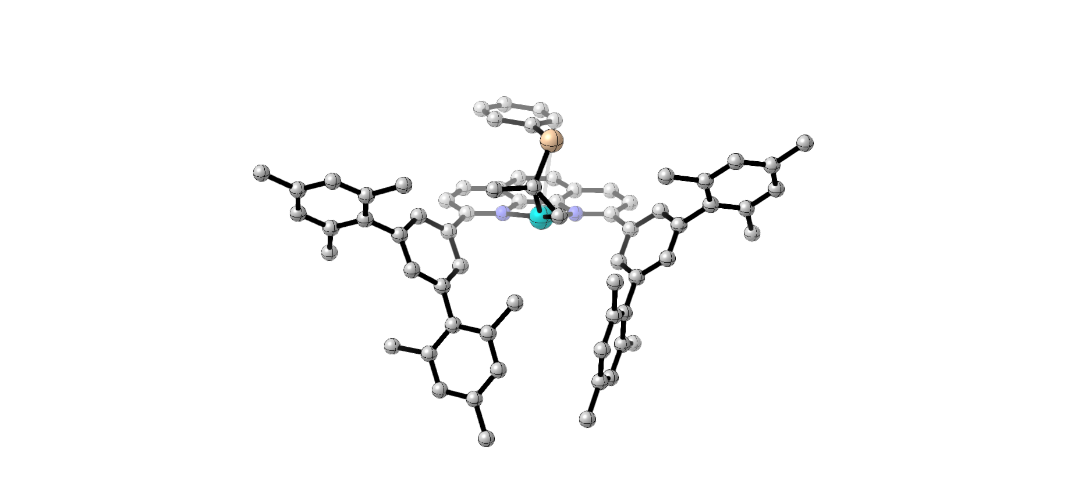** | E = -4333.300631 | 1.073426 |

**Table** **S17** Cartesian coordinates of calculated structures.

**^3^CA**

Fe -0.06614800 0.26198900 -0.57405900

Cl 0.02579700 -1.84436500 0.15703200

Cl 0.09457600 0.95027900 -2.72849300

N 1.16259900 1.65889100 0.06450200

N -1.47902500 1.64371600 0.17206100

C 2.48904700 1.58104400 0.14282800

C 3.25891400 2.74612000 0.36024100

C 2.65429900 3.97024000 0.50705900

C 1.24635100 4.05164000 0.48340200

C 0.55058200 2.85637200 0.26797100

C -0.88307500 2.84893600 0.30985000

C -2.79769500 1.53895900 0.27757300

C -3.59123000 2.68710100 0.49431300

C -2.99726500 3.92216200 0.61517400

C -1.59210900 4.03492600 0.53968300

C -0.85828800 5.25787600 0.70728300

C 0.50008600 5.26320200 0.68450500

C 3.13205700 0.24066000 0.06191500

C 3.73520200 -0.18351400 -1.12995900

C 4.34584000 -1.43858500 -1.14594600

C 4.36132200 -2.26406800 -0.02857900

C 3.75591900 -1.80939800 1.14290400

C 3.13002700 -0.57178700 1.21456100

C 3.75218600 0.68684300 -2.37789600

C 5.11948400 1.36782500 -2.53877900

C 3.38236600 -0.09380000 -3.64450300

C 4.98432400 -3.64605900 -0.09230800

C 6.09931100 -3.82211000 0.94682400

C 3.90948200 -4.73210000 0.05879500

C 2.50624100 -0.09799100 2.52195400

C 3.54150300 0.67583900 3.35330200

C 1.88342800 -1.22679900 3.34935500

C -3.34216000 0.15421300 0.21289800

C -3.42340100 -0.58176000 1.40491500

C -3.78149800 -1.92602900 1.32217700

C -4.05203100 -2.54034500 0.10459200

C -3.99220500 -1.77188700 -1.05731000

C -3.63208000 -0.42839500 -1.03372400

C -3.06107600 0.03482400 2.75061300

C -1.61211700 -0.30153100 3.13352300

C -4.02767900 -0.37105900 3.86992300

C -3.59070900 0.39463700 -2.31398200

C -3.16994100 -0.41208600 -3.54573900

C -4.95132800 1.06970200 -2.54778800

H 4.33647200 2.64138400 0.41143600

H 3.24579600 4.86778000 0.66352100

H -4.66540500 2.56824400 0.57945800

H -3.59907200 4.81061000 0.78557200

H -1.40939900 6.17977700 0.86739900

H 1.04789300 6.18981500 0.82786100

H 4.80909900 -1.79136600 -2.06366000

H 3.75003500 -2.45386800 2.01658100

H 2.98964900 1.46502700 -2.26374300

H 5.37642300 1.97777100 -1.66523400

H 5.12387300 2.01804300 -3.42089700

H 5.91121500 0.61958600 -2.66394600

H 4.15584700 -0.82215500 -3.91481800

H 3.27135200 0.59984600 -4.48447500

H 2.43121000 -0.61524700 -3.51375300

H 5.43564900 -3.75727800 -1.08687900

H 6.87101900 -3.05330700 0.83403800

H 6.57307400 -4.80428400 0.83894400

H 5.70348600 -3.75444500 1.96682500

H 3.43636200 -4.67891300 1.04593100

H 4.35062800 -5.72928400 -0.05309100

H 3.12189800 -4.61116500 -0.69115900

H 1.68950100 0.59002700 2.27865500

H 4.38253000 0.02363100 3.61604400

H 3.09319600 1.04593000 4.28255500

H 3.94280800 1.53488000 2.80451900

H 1.20152000 -1.82365800 2.73739500

H 1.31933700 -0.80035800 4.18642400

H 2.64554800 -1.88850900 3.77717600

H -3.81884900 -2.52456500 2.22856200

H -4.19870800 -2.24449100 -2.01244000

H -3.13125100 1.12543000 2.65613400

H -1.47351900 -1.38473900 3.21430100

H -1.35532500 0.15562600 4.09675200

H -0.90437700 0.05145900 2.37784400

H -5.06893600 -0.18121900 3.58922100

H -3.80678800 0.19716200 4.78023900

H -3.93275400 -1.43310900 4.12034400

H -2.83612300 1.17993200 -2.19152200

H -2.23638300 -0.95068900 -3.36229100

H -2.99924400 0.26783000 -4.38611800

H -3.94304500 -1.12796500 -3.84951600

H -5.73824600 0.31493300 -2.66332100

H -4.92642600 1.68010500 -3.45752900

H -5.23246400 1.71883300 -1.71090400

C -4.35218200 -4.02579300 0.03797200

H -4.40082100 -4.39681800 1.07018400

C -5.70565300 -4.31541800 -0.62310300

H -5.91911900 -5.39001400 -0.60163600

H -6.51918700 -3.79272900 -0.10884900

H -5.71144000 -3.99836200 -1.67242000

C -3.21207000 -4.76913400 -0.67303300

H -3.14042800 -4.46151700 -1.72267100

H -2.24917200 -4.55339300 -0.20019600

H -3.38351200 -5.85154800 -0.64912900

**^5^CA**

Fe -0.07398700 0.14308500 -0.68366400

Cl -0.10971400 -1.94604300 0.08765700

Cl -0.01520300 0.86209000 -2.82323500

N 1.26604100 1.65316400 0.14830000

N -1.41216500 1.66694700 0.15828800

C 2.58870600 1.57186400 0.22869200

C 3.36936100 2.72581700 0.46078900

C 2.76140200 3.95076800 0.59646000

C 1.35519000 4.04293300 0.53536100

C 0.65380900 2.84741700 0.31699300

C -0.78830800 2.85666900 0.31164800

C -2.73501800 1.59866500 0.25343600

C -3.50729500 2.76482400 0.44612600

C -2.88660900 3.98590800 0.56097700

C -1.47831100 4.06125000 0.51609600

C -0.73368600 5.27662100 0.69287700

C 0.62356500 5.26723000 0.70458800

C 3.20400800 0.22040200 0.10553700

C 3.77842700 -0.17815800 -1.11062400

C 4.35043200 -1.44902700 -1.18243300

C 4.34889300 -2.31944000 -0.09888100

C 3.75883900 -1.89684000 1.09187500

C 3.17444400 -0.64250700 1.21909300

C 3.79481800 0.73504700 -2.32897700

C 5.16058100 1.42395500 -2.46804200

C 3.42672100 0.00120500 -3.62402000

C 4.94016400 -3.71168500 -0.21939400

C 6.06300000 -3.94388900 0.80026900

C 3.84664200 -4.78200000 -0.09389800

C 2.56520500 -0.20724200 2.54623700

C 3.62929200 0.47873400 3.41704500

C 1.88869500 -1.34988300 3.31024400

C -3.32500300 0.23058800 0.22414700

C -3.38737900 -0.48296400 1.43184300

C -3.81139300 -1.80968100 1.38875200

C -4.16759000 -2.42874600 0.19566600

C -4.12586400 -1.68138200 -0.98025600

C -3.70272700 -0.35595700 -0.99578000

C -2.94849200 0.14040200 2.75198800

C -1.49539100 -0.23142600 3.08245800

C -3.87699600 -0.22141000 3.91810600

C -3.68258300 0.44181900 -2.29179900

C -3.30990000 -0.39843600 -3.51714600

C -5.03725300 1.13518100 -2.50470900

H 4.44581000 2.62038200 0.53162900

H 3.35159900 4.84701300 0.76688200

H -4.58483300 2.67055000 0.51832000

H -3.46785600 4.89190500 0.70925400

H -1.27942400 6.20539100 0.83091400

H 1.17955200 6.18841200 0.85228800

H 4.79470100 -1.77959900 -2.11752100

H 3.73443800 -2.57807600 1.93688600

H 3.03145200 1.50855800 -2.18691200

H 5.41852200 2.00195300 -1.57372900

H 5.16301100 2.10534200 -3.32621800

H 5.95166100 0.68034600 -2.62133200

H 4.20511500 -0.70914500 -3.92583200

H 3.30790700 0.72620500 -4.43592100

H 2.48063000 -0.53427200 -3.51391500

H 5.37865000 -3.79684500 -1.22221900

H 6.84674500 -3.18500500 0.70527500

H 6.51821600 -4.92982000 0.65367400

H 5.67924300 -3.90449600 1.82635100

H 3.38227600 -4.75329900 0.89836600

H 4.26871200 -5.78274300 -0.24148700

H 3.05545300 -4.62328600 -0.83298300

H 1.78175900 0.52822600 2.33539400

H 4.44059900 -0.22102400 3.64889700

H 3.19354300 0.82225200 4.36229600

H 4.06860500 1.34526200 2.91032100

H 1.18756000 -1.88613700 2.66419300

H 1.33587900 -0.94449800 4.16493700

H 2.61890200 -2.06487100 3.70692600

H -3.83714100 -2.39155500 2.30625700

H -4.40071600 -2.15695900 -1.91679700

H -2.99227200 1.23118500 2.64509800

H -1.38066700 -1.31749100 3.16157400

H -1.19184500 0.22119200 4.03427800

H -0.80628600 0.10501100 2.30222300

H -4.92492100 -0.01661700 3.67533200

H -3.60806100 0.36244300 4.80537200

H -3.79267900 -1.27926200 4.18936100

H -2.91229900 1.21636000 -2.20309200

H -2.38754800 -0.95895900 -3.34366700

H -3.14153900 0.25959300 -4.37533400

H -4.10747200 -1.10014300 -3.78896600

H -5.83923000 0.39162500 -2.58272300

H -5.02658400 1.72494200 -3.42825600

H -5.28515700 1.80692500 -1.67539500

C -4.53886400 -3.89946600 0.16946200

H -4.57033600 -4.24746700 1.21036300

C -5.92473000 -4.14002000 -0.44192300

H -6.18677100 -5.20261400 -0.38701700

H -6.69576700 -3.56781200 0.08481200

H -5.95040100 -3.84786000 -1.49812800

C -3.45697800 -4.70780400 -0.56133300

H -3.40505500 -4.42296200 -1.61854300

H -2.47092600 -4.52728100 -0.12272500

H -3.67557400 -5.78076800 -0.51012600

**^3^CA1**

Fe 0.10423400 0.02617800 0.00215100

C 4.63264300 -2.27840400 -0.19272200

C 2.77850800 1.59143400 -0.09045700

C 4.29471400 -1.61764800 -1.37051400

C -3.65919800 -0.33011600 1.35982000

C -4.83674700 -2.10642000 0.17464400

C -4.50506000 -1.49412900 -1.03254000

N 1.42259500 1.59870400 -0.04125700

C -3.76152700 -0.31659800 -1.08289800

C 3.77197200 -0.38780600 1.08003500

C 5.22952900 -3.67307200 -0.23324800

C -4.40664300 -1.50946900 1.35507800

C 0.78324200 2.82372600 -0.01441800

C 3.68909800 -0.36001700 -1.36216400

C -3.43982100 0.32608100 -2.42784300

C 3.34911700 0.34307100 -2.66929000

C -0.61988400 2.81337800 0.03506500

C -3.22399900 0.29517100 2.68084500

C 4.37310900 -1.64327500 1.02201800

C 1.50547200 4.05135900 -0.03673500

N -1.25298200 1.57925600 0.05460800

C -2.60403900 1.57158400 0.10332400

C -5.62961400 -3.40036500 0.20251000

C -4.84723800 -4.54407600 -0.45748400

C 3.52254000 0.29013500 2.42079400

C -3.25666300 -0.68990100 -3.56353100

C 3.52927100 2.75051500 -0.11366400

C 4.23775600 -4.70157700 0.32932500

C 3.43290800 0.25180200 -0.12538400

C 3.01627600 -0.66558100 3.50699700

C -1.34264600 4.04178900 0.06237800

C 2.88969700 4.00342200 -0.08627000

C 0.75270300 5.28107300 -0.00795500

C 2.73001700 -0.58677100 -3.71901300

C 4.79454400 1.01275400 2.88818700

C -4.25790200 1.32174600 3.17160300

C 4.59621700 1.04103800 -3.23102800

C -0.59870700 5.27541700 0.03889100

C -2.97211100 -0.73242800 3.79244000

C -2.72496100 3.98955700 0.11092500

C -4.52678800 1.33681400 -2.82763200

C 0.65585200 -1.89100500 0.69434300

C -7.01306600 -3.23422500 -0.44009400

C -3.35426600 2.74114800 0.13168600

C -0.52123600 -1.47768300 -1.46593600

C 6.57762200 -3.74972500 0.49407400

C -3.33562300 0.26558600 0.12734600

C 0.60000800 -1.88596900 -0.74228000

C -0.40426600 -1.48198700 1.50514800

H 4.49931100 -2.10693300 -2.31983600

H -4.82903400 -1.95438300 -1.96160000

H 5.40581600 -3.92127100 -1.28797700

H -4.65638500 -1.98675300 2.29879800

H -2.49273900 0.86590200 -2.31446100

H 2.60570300 1.11557600 -2.44864100

H -2.27868000 0.81899400 2.49804000

H 4.63421300 -2.14872600 1.94812800

H -5.78295600 -3.66568300 1.25668600

H -4.68115300 -4.34310100 -1.52228900

H -5.39745300 -5.48835300 -0.37612600

H -3.86803200 -4.67353400 0.01487400

H 2.74467400 1.04629800 2.27221500

H -2.60099000 -1.51639300 -3.27660800

H -2.81978100 -0.19295700 -4.43632800

H -4.21530100 -1.11532300 -3.88292200

H 4.61043600 2.67487300 -0.15595200

H 4.04310600 -4.51391100 1.39190400

H 4.63649300 -5.71792300 0.23420600

H 3.27986300 -4.65429000 -0.19916000

H 2.12313300 -1.20795500 3.18165100

H 2.75594600 -0.09874500 4.40748100

H 3.77535200 -1.40112300 3.79595100

H 3.46775900 4.92279200 -0.10462300

H 1.30124500 6.21917600 -0.02529700

H 3.44112200 -1.34119600 -4.07381100

H 2.41377800 -0.00370300 -4.59082200

H 1.85076300 -1.10537100 -3.32470200

H 5.61345200 0.29705800 3.02886700

H 4.62118400 1.52573100 3.84096700

H 5.11524300 1.75667300 2.15262000

H -4.39243900 2.13356600 2.45318400

H -3.93774200 1.76067800 4.12349000

H -5.22904000 0.83731300 3.32887900

H 4.99386100 1.76675000 -2.51464400

H 4.35836300 1.57193200 -4.15981800

H 5.38369100 0.30936800 -3.44779800

H -1.15507800 6.20887800 0.05998200

H -3.91064300 -1.14670700 4.17920100

H -2.46502200 -0.24794800 4.63382400

H -2.35119200 -1.56586200 3.45274500

H -3.30732900 4.90652500 0.13285700

H -5.49852200 0.83568700 -2.91215700

H -4.29146500 1.78794200 -3.79839200

H -4.61937300 2.14159500 -2.09476000

H 1.64333600 -2.01547900 1.12858600

H -7.57467200 -2.42498000 0.03767200

H -7.59314400 -4.15935800 -0.34834700

H -6.92683900 -2.99990300 -1.50744600

H -4.43469600 2.66203800 0.17205200

H -0.42647500 -1.34970400 -2.53984300

H 7.28444800 -3.01478700 0.09542500

H 7.01823300 -4.74680400 0.38303600

H 6.46093700 -3.55677100 1.56673000

H 1.55160700 -2.00382800 -1.25211400

H -0.22539000 -1.35939500 2.56903500

H -1.44154800 -1.65317200 1.22651600

H -1.53218800 -1.65152000 -1.10476600

**^3^CA1** (ORCA 303)

Fe -0.08875225 0.18637032 -0.52071545

C 4.634643480 -2.220069428 0.045167468

C 2.659383264 1.593839546 -0.279926607

C 4.730383263 -1.465207568 -1.122901127

C -3.24766093 -0.44727819 1.47974691

C -4.49956478 -2.32024595 0.53141653

C -4.55758752 -1.64080722 -0.68902732

N 1.300774281 1.612731160 -0.333170194

C -3.98265565 -0.37662526 -0.85984598

C 3.237791680 -0.467776587 1.023756760

C 5.295840069 -3.581374580 0.144904722

C -3.84837289 -1.70387567 1.60304037

C 0.680183055 2.852188738 -0.308387963

C 4.102886174 -0.218850008 -1.258783422

C -4.09314781 0.34172685 -2.19777095

C 4.282786321 0.567714534 -2.550487312

C -0.73139568 2.84383524 -0.19828272

C -2.55239640 0.20364724 2.66628235

C 3.892486913 -1.697130939 1.112192078

C 1.388568551 4.082702418 -0.342086955

N -1.33121996 1.59269702 -0.12920176

C -2.67704220 1.55305403 0.09234326

C -5.08590724 -3.71134400 0.68381022

C -4.28479986 -4.73281561 -0.14118703

C 2.473451671 0.086512845 2.217857658

C -4.06915488 -0.60401530 -3.40653281

C 3.410296203 2.780051470 -0.287233243

C 4.244116567 -4.693965454 0.289171036

C 3.340513330 0.274682645 -0.179250013

C 1.907070131 -0.993544180 3.143788416

C -1.45709093 4.06084761 -0.12491896

C 2.794838952 4.025393187 -0.347517716

C 0.636661592 5.302412330 -0.321109432

C 4.025571447 -0.267423782 -3.814007336

C 3.359740319 1.065693812 3.009312684

C -3.52734066 1.12148036 3.42501168

C 5.691684325 1.184569943 -2.609663963

C -0.72792530 5.29167646 -0.21713342

C -1.91090115 -0.80457353 3.62477238

C -2.84832973 3.98468354 0.07497698

C -5.37159445 1.19997813 -2.25177646

C 0.617217714 -1.731540152 -0.672349328

C -6.57781169 -3.75514460 0.32063106

C -3.43192698 2.72717919 0.20285033

C -0.46420639 -0.41507988 -2.45838569

C 6.322862429 -3.636608680 1.286534009

C -3.31800558 0.21676092 0.23625137

C 0.691036770 -1.059143327 -1.929430685

C -0.60307886 -1.73475965 0.06133150

H 5.309538519 -1.865468967 -1.958207338

H -5.05179870 -2.11433814 -1.53837336

H 5.834833263 -3.746399821 -0.803056270

H -3.79438126 -2.23578974 2.55477929

H -3.22482071 1.01538006 -2.27794732

H 3.552307391 1.390694751 -2.546940973

H -1.74209700 0.82857491 2.26057024

H 3.810507815 -2.273998064 2.033958621

H -4.98698533 -3.98504426 1.74773240

H -4.35103711 -4.50255995 -1.21518810

H -4.67344508 -5.75061562 0.01271451

H -3.22223975 -4.71961014 0.13921174

H 1.615508712 0.651453667 1.823407136

H -3.23008524 -1.31118515 -3.36436229

H -3.97941294 -0.02134262 -4.33442818

H -4.99881553 -1.18817759 -3.47793555

H 4.495277742 2.700456682 -0.237430086

H 3.678095631 -4.577225006 1.225302851

H 4.723578909 -5.683981072 0.304521572

H 3.525801182 -4.668671775 -0.542290300

H 1.300645892 -1.721315760 2.587462890

H 1.265410613 -0.528389234 3.904595858

H 2.700061729 -1.539848951 3.675457789

H 3.382365179 4.944145482 -0.365185809

H 1.180624407 6.247564196 -0.368278924

H 4.750516555 -1.088189840 -3.915791920

H 4.117908962 0.367319103 -4.707254926

H 3.019435924 -0.707205224 -3.816019294

H 4.235711349 0.542428926 3.422429656

H 2.796664027 1.507087562 3.845198628

H 3.722884329 1.883619975 2.372334700

H -3.94541650 1.89367550 2.76559828

H -3.01695136 1.62438169 4.26017421

H -4.36320314 0.53505121 3.83594168

H 5.896916873 1.812002039 -1.731987090

H 5.806655864 1.805104501 -3.510782801

H 6.456610781 0.393984905 -2.639539588

H -1.28737965 6.22824165 -0.18112906

H -2.66622451 -1.38817325 4.17179312

H -1.30063312 -0.27740492 4.37183589

H -1.26036233 -1.50588969 3.08611282

H -3.44452456 4.89440900 0.15154736

H -6.26146672 0.56021271 -2.14993554

H -5.43970252 1.73081875 -3.21319736

H -5.39580455 1.94613828 -1.44822803

H 1.553546324 -2.026993683 -0.196677712

H -7.15280823 -3.03193417 0.91615972

H -6.99095702 -4.75848665 0.50133519

H -6.73283908 -3.51701252 -0.74227564

H -4.49836888 2.63035498 0.40077336

H -0.34209064 0.25745644 -3.30987719

H 7.085667054 -2.853431434 1.171999322

H 6.829694904 -4.612718911 1.304881974

H 5.836536868 -3.493322798 2.262978932

H 1.679305008 -0.883233804 -2.352288132

H -0.57172751 -2.07423291 1.09796119

H -1.56763915 -1.89850524 -0.42726456

H -1.44313011 -0.89708003 -2.39989153

**^3^CA1** (ORCA 502)

Fe -0.05982999 0.07538809 -0.55274923

C 5.035226281 -2.137307800 -0.035296411

C 2.709478332 1.515626609 -0.136973652

C 4.975873856 -1.350814283 -1.191082485

C -3.51681985 -0.38501044 1.44862762

C -4.94039646 -2.18920460 0.58729685

C -4.86113619 -1.60897457 -0.68560909

N 1.349271447 1.510557794 -0.255386695

C -4.12904281 -0.43730529 -0.92988732

C 3.536091475 -0.542350616 1.072208659

C 5.879033207 -3.404990380 -0.004943426

C -4.26509314 -1.55662230 1.63763286

C 0.706098374 2.744472723 -0.239722605

C 4.221232503 -0.168694751 -1.258778237

C -4.15348386 0.17955979 -2.32879776

C 4.279248270 0.675841153 -2.533757197

C -0.71881506 2.73293329 -0.21523396

C -2.89818092 0.31217381 2.65944867

C 4.314728128 -1.707424994 1.088097807

C 1.402120653 3.987076341 -0.190243283

N -1.34017208 1.48356061 -0.19053246

C -2.70356617 1.46660719 -0.04257513

C -5.74403353 -3.45954153 0.83479880

C -5.18754712 -4.65522551 0.03826206

C 2.852833096 -0.077355938 2.357155896

C -4.02600849 -0.85749946 -3.46033771

C 3.437421728 2.715664420 -0.032075870

C 5.037086488 -4.655887698 0.309187093

C 3.472281371 0.224541585 -0.120022178

C 2.108370648 -1.203218600 3.094885084

C -1.44012977 3.95976310 -0.20995641

C 2.805744990 3.951115282 -0.084283620

C 0.654180237 5.209721680 -0.223581018

C 4.076071972 -0.136074072 -3.826724730

C 3.876147385 0.597599193 3.295141373

C -3.97315694 1.12527611 3.41273942

C 5.616999010 1.442913071 -2.616701932

C -0.71337665 5.19603697 -0.23249327

C -2.18401346 -0.64844413 3.62578763

C -2.84512455 3.90219894 -0.12352511

C -5.43833706 1.00880783 -2.54357926

C 0.626120455 -1.861740068 -0.656325428

C -7.24313344 -3.25561187 0.54164440

C -3.45097842 2.65582752 -0.01638427

C -0.33473613 -0.55502125 -2.53208287

C 7.060299362 -3.278176453 0.977133117

C -3.42888196 0.16733184 0.14622876

C 0.774589097 -1.209108486 -1.921511445

C -0.63005033 -1.85090353 0.01823744

H 5.548453139 -1.667523614 -2.067080833

H -5.39425991 -2.07777321 -1.51581359

H 6.299002554 -3.529865577 -1.017478676

H -4.33905118 -1.98646608 2.64017120

H -3.29352690 0.86471096 -2.40190693

H 3.468692050 1.419119984 -2.480262393

H -2.14502692 1.02451218 2.29020306

H 4.368836181 -2.292832424 2.009069023

H -5.64164006 -3.69524800 1.90773305

H -5.27742848 -4.48395852 -1.04563002

H -5.74540281 -5.57268297 0.28121256

H -4.12577502 -4.82872252 0.26810735

H 2.105288675 0.680972732 2.080848884

H -3.16125705 -1.52073069 -3.31743537

H -3.90596648 -0.34344025 -4.42587336

H -4.92551234 -1.48717870 -3.53450684

H 4.519505466 2.653521639 0.081017402

H 4.608189985 -4.601912231 1.321753210

H 5.661895960 -5.560757605 0.257875401

H 4.208787752 -4.771115390 -0.405672764

H 1.377286900 -1.696108974 2.438382815

H 1.567502740 -0.790241555 3.959556845

H 2.799481892 -1.970315054 3.475954614

H 3.374682807 4.880924935 -0.027522622

H 1.203631650 6.153567621 -0.214488320

H 4.890537323 -0.858975106 -3.984479110

H 4.066334519 0.542971172 -4.692753110

H 3.127516535 -0.691131883 -3.823925250

H 4.641917350 -0.125091455 3.618058571

H 3.374863322 0.986282661 4.195112539

H 4.387495052 1.435260610 2.798197658

H -4.46046641 1.85650814 2.75152110

H -3.52218051 1.67161074 4.25571893

H -4.75119881 0.45836659 3.81624993

H 5.788789227 2.065228403 -1.727528379

H 5.628479852 2.098500254 -3.501154525

H 6.459393339 0.739152135 -2.704299352

H -1.28120953 6.12894655 -0.24228923

H -2.88965028 -1.34147493 4.10877830

H -1.68846166 -0.07540446 4.42392562

H -1.41957554 -1.24612167 3.10941950

H -3.43259948 4.82174006 -0.10992551

H -6.32665948 0.36125722 -2.47690181

H -5.43153863 1.47359841 -3.54174981

H -5.54477519 1.80747515 -1.79657398

H 1.534855119 -2.157777988 -0.126003033

H -7.65684735 -2.42540491 1.13334028

H -7.81074916 -4.16667853 0.78597401

H -7.40997126 -3.03048719 -0.52306929

H -4.53139592 2.57783263 0.10383834

H -0.14462710 0.08150063 -3.40042757

H 7.686962599 -2.406280490 0.737459109

H 7.693972513 -4.177478238 0.935615165

H 6.702802976 -3.166136893 2.012558766

H 1.787466461 -1.057368937 -2.296212720

H -0.65174064 -2.19172492 1.05611759

H -1.56897940 -2.00549245 -0.52265338

H -1.32647214 -1.01573430 -2.52244050

**^5^CA1**

Fe -0.12356200 0.01800900 -0.03635300

C -4.57167100 -2.29027900 0.16812900

C -2.82320600 1.62992100 0.09789200

C -4.34804500 -1.59004900 1.34961900

C 3.66185000 -0.31955700 -1.34077200

C 4.79223800 -2.11494800 -0.13849100

C 4.44212600 -1.50331300 1.06400100

N -1.47515000 1.62505500 0.04308500

C 3.71089600 -0.31767500 1.10373700

C -3.73143200 -0.37970500 -1.08802900

C -5.09830700 -3.71304800 0.19835700

C 4.39530000 -1.50782000 -1.32518500

C -0.80675800 2.83814400 0.01520100

C -3.81990400 -0.29701600 1.35396900

C 3.36716300 0.32368000 2.44391800

C -3.58418700 0.42919500 2.67207800

C 0.59515300 2.82104600 -0.03626600

C 3.26422100 0.31643000 -2.66846600

C -4.28301800 -1.65756500 -1.04160700

C -1.52400600 4.07475800 0.04016600

N 1.24500300 1.59104600 -0.06002900

C 2.59184300 1.58420700 -0.09840400

C 5.57150700 -3.41730800 -0.15497000

C 4.77015000 -4.55288700 0.49613500

C -3.45654700 0.30107100 -2.42323600

C 3.16871600 -0.69192800 3.57725300

C -3.56823400 2.79602700 0.12587300

C -4.05766600 -4.69370100 -0.36050300

C -3.48280500 0.29098900 0.12447200

C -2.98547200 -0.65186300 -3.52691400

C 1.32511100 4.05090300 -0.06102000

C -2.90550000 4.03890000 0.09604100

C -0.76475000 5.30115100 0.00970300

C -2.23874400 0.02874100 3.29291400

C -4.70694400 1.06827500 -2.88075700

C 4.32518300 1.32662800 -3.13472400

C -4.72732700 0.22975400 3.67455300

C 0.58541400 5.28824900 -0.03785300

C 3.01779400 -0.70365400 -3.78827600

C 2.70520000 3.99918200 -0.10394300

C 4.44562200 1.33687700 2.86069100

C -0.66300100 -1.90969400 -0.74886600

C 6.94830900 -3.26353000 0.50480000

C 3.34548600 2.75197100 -0.12058300

C 0.47886700 -1.52133500 1.43242000

C -6.43697500 -3.84613100 -0.53894600

C 3.31749800 0.27419200 -0.11313200

C -0.63388500 -1.90738400 0.69063200

C 0.40598100 -1.50420900 -1.54361600

H -4.58722100 -2.07236800 2.29429000

H 4.74097000 -1.97105200 1.99773700

H -5.27108700 -3.97344500 1.25071700

H 4.66042000 -1.98380000 -2.26539900

H 2.42128400 0.86233500 2.31564000

H -3.53215700 1.50038200 2.45285000

H 2.32516200 0.85715800 -2.50326700

H -4.47584000 -2.18550700 -1.97166000

H 5.73532600 -3.68529200 -1.20690900

H 4.59525500 -4.35125500 1.55940900

H 5.31142500 -5.50271500 0.41986400

H 3.79459700 -4.67211200 0.01358500

H -2.65626200 1.03188900 -2.26675000

H 2.52236200 -1.52247600 3.28095800

H 2.71430000 -0.19582100 4.44152400

H 4.12362300 -1.11218400 3.91430300

H -4.64917100 2.73287100 0.18116600

H -3.86995000 -4.50150700 -1.42351700

H -4.40734000 -5.72766700 -0.26336500

H -3.10357500 -4.60013100 0.16894800

H -2.11757400 -1.24083500 -3.21420500

H -2.69774300 -0.07581300 -4.41292400

H -3.77461000 -1.34653200 -3.83631900

H -3.47053000 4.96676700 0.11710500

H -1.30867500 6.24203300 0.02760900

H -2.20025800 -1.05195500 3.48054800

H -2.08700500 0.54412900 4.24807900

H -1.40967500 0.28993900 2.62899600

H -5.54668700 0.37841500 -3.02736100

H -4.51847600 1.58469800 -3.82880600

H -5.00286900 1.81475100 -2.13806600

H 4.45603700 2.13479800 -2.41173500

H 4.03349400 1.77177100 -4.09284900

H 5.29158300 0.82670000 -3.27146000

H -5.69975400 0.44117000 3.21787400

H -4.59512500 0.90392400 4.52761100

H -4.75386400 -0.79197300 4.07050200

H 1.14648600 6.21904800 -0.05875800

H 3.95713900 -1.13078800 -4.15858700

H 2.53414200 -0.20900800 -4.63748700

H 2.37854300 -1.52924400 -3.46386300

H 3.28358700 4.91901000 -0.12242000

H 5.41679700 0.83747300 2.96081600

H 4.19352800 1.78728600 3.82754800

H 4.54708900 2.14130600 2.12880500

H -1.64235500 -2.03199300 -1.19965800

H 7.52405600 -2.46122200 0.03226500

H 7.52027000 -4.19465200 0.42290800

H 6.85038900 -3.02532900 1.57029900

H 4.42612600 2.67630900 -0.14965300

H 0.36850600 -1.39542100 2.50447900

H -7.17790900 -3.14561000 -0.14036200

H -6.83380800 -4.86245400 -0.43754700

H -6.32076700 -3.63973000 -1.60925500

H -1.59625600 -2.00653800 1.18623400

H 0.24335500 -1.38304800 -2.61014500

H 1.44114600 -1.65111400 -1.24379800

H 1.49373000 -1.68865700 1.07927200

**^3^CA2**

Fe -0.09897000 0.46771200 -0.15429300

Si -0.37321600 -2.54595900 1.62018700

Si -0.20121000 -2.83380700 -1.34796400

N -1.35236000 2.08689500 -0.06024600

N 1.39806300 2.05756800 -0.14194700

C -0.66484700 3.29360000 -0.16187000

C -2.72136500 2.14405600 0.01509800

C -3.41442300 3.33214800 0.00988400

C -2.73408600 4.55882300 -0.10417900

C -1.34889500 4.54400800 -0.20085200

C -0.58307600 5.75634600 -0.33489400

C 0.76672000 5.73549500 -0.41493800

C 1.48121300 4.49529000 -0.35531000

C 2.86706900 4.43127400 -0.41611300

C 3.50148200 3.20134600 -0.31923500

C 2.73773200 2.03856500 -0.16696600

C 0.74858400 3.27361200 -0.22286900

C -3.45171700 0.83910600 0.08319100

C -3.80229700 0.28433500 1.32834700

C -4.40320500 -0.97551900 1.35425400

C -4.67762800 -1.68700200 0.19121500

C -4.39680400 -1.08059800 -1.03321500

C -3.79709800 0.17524200 -1.11402600

C -3.58275800 1.05062400 2.63090800

C -3.30544900 0.16391500 3.85362700

C -4.80034900 1.93993200 2.93292900

C -5.27761700 -3.07896000 0.26427500

C -6.66542400 -3.13633500 -0.38748100

C -4.33596000 -4.12512800 -0.34746200

C -3.63566700 0.87242600 -2.46237400

C -4.85850900 1.76735900 -2.73122500

C -3.45404800 -0.06659300 -3.65948900

C 3.46535100 0.73570600 -0.00131900

C 3.83335100 -0.01883800 -1.13273600

C 4.50742500 -1.22536700 -0.94625900

C 4.86005300 -1.68587300 0.32075600

C 4.54043000 -0.89160200 1.41796900

C 3.85966500 0.31956900 1.28592100

C 3.60598200 0.50709500 -2.54508800

C 3.25514000 -0.57416800 -3.57479300

C 4.85239200 1.27656800 -3.01395100

C 5.60281700 -2.99743500 0.50236100

C 6.96510900 -2.97271500 -0.20400500

C 4.76193900 -4.19299900 0.03783400

C 3.64302200 1.19910800 2.51480400

C 3.38945200 0.41827500 3.81238700

C 4.85224300 2.12695000 2.72004300

C 0.45193400 0.18129500 1.92799200

C -0.58098100 -0.68585300 1.66394400

C 0.04880200 -0.01879500 -2.14504500

C 0.50644600 -1.10751600 -1.37122500

O -0.81794600 -3.20168100 0.16064000

C 1.23591700 -3.99305500 -1.70447900

C -1.52274300 -3.28669000 2.90152700

C 1.43169700 -2.95563700 1.95317900

C -1.58598200 -3.10456600 -2.57435100

H -4.49601800 3.30777300 0.07952500

H -3.27509700 5.49967900 -0.12352300

H -1.12506400 6.69761500 -0.37248000

H 1.33361700 6.65691400 -0.51892800

H 3.44338000 5.34609900 -0.52794400

H 4.58164700 3.12140900 -0.34775100

H -4.65718100 -1.42675900 2.30990700

H -4.65645500 -1.60380900 -1.94959000

H -2.71546500 1.70687200 2.48918700

H -4.20337400 -0.38075400 4.16739500

H -2.99984400 0.79201700 4.69725000

H -2.51321100 -0.56830100 3.67527600

H -4.97198900 2.67186900 2.14082800

H -4.65272500 2.48410500 3.87280000

H -5.70309400 1.32578800 3.03269700

H -5.39998200 -3.32143600 1.32829900

H -7.34410400 -2.40154800 0.05813700

H -7.10734100 -4.13189000 -0.26661300

H -6.60280500 -2.92846000 -1.46209400

H -4.24212600 -3.97762300 -1.42965200

H -4.72718700 -5.13565600 -0.18217100

H -3.33150400 -4.05859000 0.08284800

H -2.75113300 1.51690400 -2.40394800

H -5.77103600 1.16021000 -2.76656500

H -4.75129600 2.27938000 -3.69435100

H -4.98353600 2.52656500 -1.95681100

H -2.64848800 -0.78857800 -3.51324300

H -3.21990900 0.52239800 -4.55282800

H -4.37076600 -0.62761700 -3.87592500

H 4.77720400 -1.81682900 -1.81688300

H 4.83676200 -1.22989700 2.40726600

H 2.77540700 1.21958300 -2.51060900

H 4.11782900 -1.21181900 -3.80010500

H 2.95238000 -0.10018000 -4.51467200

H 2.43378600 -1.21170800 -3.23851400

H 5.08791400 2.10785200 -2.34409100

H 4.69627200 1.68371100 -4.01931200

H 5.72183600 0.60925200 -3.04575800

H 5.78783000 -3.12074100 1.57735000

H 6.84479300 -2.87320800 -1.28893300

H 7.51426400 -3.90147000 -0.01323200

H 7.57497300 -2.13283100 0.14427400

H 3.81002700 -4.23555000 0.57662000

H 5.29639700 -5.13364200 0.21173300

H 4.54016200 -4.12709200 -1.03373200

H 2.76797900 1.83143400 2.32842400

H 2.61992500 -0.35108400 3.69914000

H 3.06307800 1.10907100 4.59686400

H 4.30216900 -0.06953500 4.17279000

H 5.76440300 1.53781200 2.87046700

H 4.70331100 2.75690200 3.60430300

H 5.00776200 2.78247600 1.86010400

H 1.47768900 -0.16993600 1.94828700

H 0.28659000 1.18448000 2.31755800

H -1.58768500 -0.31129200 1.81496200

H -0.92594100 -0.07229900 -2.62499700

H 0.73281100 0.69148800 -2.60448600

H 1.57827700 -1.10358400 -1.14637500

H 1.61300700 -3.84649800 -2.72405200

H 0.94681200 -5.04414100 -1.59488700

H 2.06480300 -3.79658200 -1.01518500

H -1.31214000 -2.90152300 3.90575400

H -1.43307500 -4.37824000 2.92743800

H -2.56189700 -3.03927500 2.65432100

H 2.09684000 -2.43050500 1.25635900

H 1.60239400 -4.03154400 1.83159600

H 1.73393100 -2.67666100 2.96932200

H -2.47097000 -2.53649900 -2.27530200

H -1.86323700 -4.16468400 -2.59392500

H -1.29998700 -2.80631500 -3.58906700

**^3^CA2** (TPSSh/6-31g*-TZVP(Fe))

Fe -0.03196100 0.37163400 -0.00331400

Si 0.05210100 -2.78833700 1.57766000

Si 0.14053500 -2.81504500 -1.47421300

N -1.39332600 1.96987300 -0.03661100

N 1.32801400 1.98046700 -0.08774200

C -0.75362000 3.19622700 -0.11491600

C -2.75148200 1.98842700 0.07451400

C -3.48444500 3.18595600 0.12500000

C -2.85179800 4.41568000 0.01472600

C -1.45694300 4.43943700 -0.12343600

C -0.73635600 5.66956100 -0.26626900

C 0.61978800 5.66962100 -0.38353400

C 1.35855500 4.44250100 -0.33850600

C 2.75713400 4.42468700 -0.42719500

C 3.40841000 3.20308300 -0.33430400

C 2.69012600 2.00973000 -0.15281500

C 0.67246400 3.19890600 -0.18564300

C -3.55314300 0.71695700 0.10492400

C -3.97781900 0.17454600 1.34250700

C -4.79261100 -0.96751000 1.33258400

C -5.21920000 -1.57100000 0.14727200

C -4.84024400 -0.97679700 -1.06351100

C -4.03202900 0.16589400 -1.11258100

C -3.66320800 0.85378600 2.67622400

C -3.27190700 -0.12232200 3.80138300

C -4.86974400 1.70285900 3.13378600

C -6.09573300 -2.81525000 0.18134900

C -7.47602400 -2.55909500 -0.45471000

C -5.40141200 -4.01912400 -0.48391000

C -3.79620500 0.86499200 -2.45307500

C -4.95967200 1.83648800 -2.75376900

C -3.63405600 -0.09156500 -3.64804500

C 3.51405000 0.76169600 0.00174900

C 4.02111600 0.09832400 -1.14644200

C 4.85591000 -1.01240800 -0.96858200

C 5.23166200 -1.46710900 0.30044000

C 4.77799600 -0.75155500 1.41275700

C 3.93795000 0.36481700 1.29446200

C 3.76785100 0.62357000 -2.56280500

C 3.50780400 -0.47472800 -3.61108800

C 4.96413200 1.48275900 -3.02966200

C 6.12518400 -2.68768000 0.47337200

C 7.50321200 -2.48875500 -0.18646100

C 5.44382200 -3.96516800 -0.05494900

C 3.60934900 1.19756100 2.53591700

C 3.42081600 0.37111600 3.82138100

C 4.71996700 2.24358500 2.78005000

C -0.01647000 0.10794100 2.06379800

C -0.59958300 -1.03820600 1.47380700

C -0.07866500 0.04932000 -2.07128200

C 0.63659300 -1.01312100 -1.47651300

O 0.24020100 -3.46774000 0.06130400

C 1.36218500 -3.80898300 -2.52110200

C -1.22769600 -3.84632500 2.48493300

C 1.70736200 -2.91900300 2.47029500

C -1.60327900 -3.09318600 -2.13937700

H -4.56244400 3.12313800 0.22686600

H -3.41532800 5.34466100 0.02722400

H -1.30053000 6.59864600 -0.28113600

H 1.17214700 6.59862300 -0.49996000

H 3.30911200 5.35229800 -0.55158600

H 4.49038900 3.14741400 -0.37990000

H -5.11438100 -1.39584100 2.27949700

H -5.19746400 -1.40683600 -1.99594600

H -2.82014900 1.53624600 2.51735100

H -4.11397300 -0.76230700 4.09081200

H -2.97561600 0.44465200 4.69197100

H -2.43541200 -0.76294500 3.51039900

H -5.13223500 2.46645200 2.39467500

H -4.64357900 2.20825900 4.08068600

H -5.74936100 1.06641800 3.28903900

H -6.25920400 -3.06682600 1.23824700

H -7.98875200 -1.72404000 0.03523900

H -8.10904500 -3.45045200 -0.36855800

H -7.38207500 -2.31819200 -1.52053700

H -5.21396700 -3.82761700 -1.54712000

H -6.03019800 -4.91457300 -0.40850300

H -4.43863200 -4.23318000 -0.00767300

H -2.87804300 1.45777500 -2.36976800

H -5.90565800 1.28615300 -2.82541500

H -4.78975700 2.34956300 -3.70845200

H -5.06720600 2.59658800 -1.97473200

H -2.88777300 -0.86764900 -3.45958900

H -3.32426200 0.47561800 -4.53338100

H -4.57995000 -0.58757300 -3.89720000

H 5.23179900 -1.53000800 -1.84770100

H 5.09967300 -1.07058400 2.40144000

H 2.88229400 1.26944600 -2.53110600

H 4.39823300 -1.09261300 -3.77768400

H 3.25388100 -0.00909600 -4.57077400

H 2.68208500 -1.12962800 -3.32277500

H 5.14868400 2.32692100 -2.35874800

H 4.77814700 1.88078000 -4.03475500

H 5.87684500 0.87576200 -3.06721600

H 6.28866300 -2.81908700 1.55182800

H 7.40768800 -2.36648800 -1.27211100

H 8.14455600 -3.35933300 -0.00363700

H 8.00745000 -1.60039000 0.20954400

H 4.47908200 -4.13101800 0.43597100

H 6.07862500 -4.84138500 0.12476000

H 5.26441400 -3.89679100 -1.13480700

H 2.67553100 1.73862000 2.34384500

H 2.70866200 -0.44775700 3.68908800

H 3.05143200 1.02043700 4.62361600

H 4.36881800 -0.05967200 4.16546700

H 5.68056600 1.74505100 2.95780900

H 4.48258200 2.85165800 3.66177300

H 4.83821400 2.91611000 1.92572600

H 0.99778400 0.05426000 2.45229200

H -0.61773800 0.91052800 2.48760900

H -1.68620800 -0.99376600 1.34702800

H -1.09352800 -0.11460200 -2.42708300

H 0.42553400 0.90344300 -2.52189500

H 1.71335700 -0.85683100 -1.37560200

H 1.34006200 -3.50409900 -3.57518300

H 1.12561600 -4.87974800 -2.47677800

H 2.38905600 -3.68264900 -2.15651900

H -1.35009300 -3.52700100 3.52803800

H -0.93010600 -4.90258200 2.48979900

H -2.21016100 -3.78090500 2.00038900

H 2.47979500 -2.30542600 1.99322400

H 2.05263000 -3.96083000 2.44877200

H 1.63110200 -2.61783300 3.52282400

H -2.35279600 -2.49063500 -1.61342500

H -1.88025600 -4.14781900 -2.01404400

H -1.66953500 -2.86135000 -3.21011000

**^3^CA2** (ORCA 303)

Fe 0.04080219 0.50302916 0.09206303

Si 0.15528934 -2.70537404 1.48695678

Si 0.30808746 -2.61108462 -1.49832574

N -1.3613038 2.0778564 -0.0653397

N 1.34895444 2.08246539 -0.05471653

C -0.7263680 3.2980149 -0.1823750

C -2.7132623 2.0611780 -0.0234102

C -3.4700657 3.2458043 -0.0801899

C -2.8484418 4.4764365 -0.2195421

C -1.4444274 4.5256612 -0.2846034

C -0.7197684 5.7483419 -0.4372452

C 0.64575941 5.75015800 -0.47199898

C 1.39351825 4.53534691 -0.34551719

C 2.79972196 4.51239952 -0.34797777

C 3.44126384 3.28775057 -0.19646712

C 2.71571388 2.10112132 -0.05114464

C 0.69875752 3.30066208 -0.19859588

C -3.4272202 0.7498752 0.0502755

C -3.7385783 0.1846780 1.3060030

C -4.4260950 -1.0347049 1.3437615

C -4.8285546 -1.6939776 0.1811353

C -4.5477027 -1.0917632 -1.0497699

C -3.8652251 0.1253066 -1.1411633

C -3.4188128 0.9200501 2.5993867

C -3.0552323 -0.0079550 3.7660489

C -4.6030333 1.8212370 2.9957817

C -5.5896274 -3.0048478 0.2580515

C -7.0197527 -2.8446515 -0.2846796

C -4.8521476 -4.1431647 -0.4619505

C -3.7040468 0.8020967 -2.4962693

C -4.9423940 1.6642289 -2.8071591

C -3.4695764 -0.1776927 -3.6543024

C 3.43589557 0.80108492 0.09605325

C 3.86228409 0.09669383 -1.05645857

C 4.43676488 -1.16802877 -0.89577670

C 4.63431029 -1.73172448 0.36811760

C 4.31765524 -0.96330822 1.49070018

C 3.72358860 0.29885335 1.38498896

C 3.77205543 0.72912218 -2.44166879

C 3.53065037 -0.26351534 -3.58658035

C 5.06223234 1.52206007 -2.72949521

C 5.17627727 -3.13843015 0.53133642

C 6.63241444 -3.24643263 0.05223193

C 4.28155416 -4.16844589 -0.17647241

C 3.48542628 1.15067545 2.62543239

C 3.28890231 0.35262308 3.92006432

C 4.65716969 2.13443127 2.81799833

C 0.03438640 0.15770601 2.11237108

C -0.5094692 -0.9718816 1.4350830

C -0.1465365 0.1950772 -2.1441678

C 0.68338886 -0.79430859 -1.61817392

O 0.72007684 -3.21077373 -0.00182001

C 1.39921627 -3.52304514 -2.72466824

C -1.2811626 -3.8253573 1.9636177

C 1.57557335 -2.91172892 2.68680630

C -1.5000350 -2.9677181 -1.8407409

H -4.5548832 3.1704590 -0.0280239

H -3.4285669 5.3983746 -0.2811817

H -1.2826089 6.6797793 -0.5206646

H 1.19807393 6.68456865 -0.58782361

H 3.36366788 5.43865969 -0.46169836

H 4.52823950 3.23006021 -0.19411799

H -4.6559907 -1.4890769 2.3090966

H -4.8746708 -1.5827075 -1.9673637

H -2.5533609 1.5711626 2.4014851

H -3.9304409 -0.5745602 4.1165443

H -2.6909980 0.5864973 4.6162091

H -2.2687749 -0.7193472 3.4842917

H -4.8442540 2.5444474 2.2059145

H -4.3719644 2.3818521 3.9139168

H -5.4990234 1.2099312 3.1815094

H -5.6614527 -3.2717682 1.3257205

H -7.5597026 -2.0499777 0.2494170

H -7.5843321 -3.7823219 -0.1742901

H -7.0051772 -2.5833273 -1.3534336

H -4.7211304 -3.9191909 -1.5307759

H -5.4176561 -5.0829135 -0.3790273

H -3.8544396 -4.3043096 -0.0319239

H -2.8340862 1.4742852 -2.4339364

H -5.8385480 1.0291449 -2.8747906

H -4.8171378 2.1849035 -3.7683432

H -5.1203511 2.4191448 -2.0314826

H -2.6555846 -0.8807340 -3.4397658

H -3.2139421 0.3755444 -4.5688731

H -4.3727296 -0.7677456 -3.8687080

H 4.72512475 -1.73381662 -1.78279399

H 4.52904475 -1.37710025 2.47787252

H 2.93318195 1.44352989 -2.42743635

H 4.38895038 -0.93785486 -3.72271038

H 3.39891389 0.28684841 -4.52924130

H 2.63511879 -0.87466847 -3.42269606

H 5.26070900 2.27554363 -1.95786913

H 4.99124839 2.03452112 -3.70043022

H 5.92479139 0.83950142 -2.76337011

H 5.15781611 -3.36071226 1.61154990

H 6.70589848 -3.02770953 -1.02387138

H 7.02056831 -4.26272225 0.21630135

H 7.28166785 -2.53779628 0.58600974

H 3.22595471 -4.04363676 0.10085752

H 4.59803798 -5.19128398 0.07689153

H 4.35244787 -4.06315472 -1.26962189

H 2.57496897 1.74480756 2.44697139

H 2.50898988 -0.41410873 3.83160840

H 3.00318744 1.03523185 4.73294569

H 4.21817540 -0.14907864 4.22837223

H 5.59908555 1.58199694 2.95422547

H 4.49079040 2.75695517 3.70974671

H 4.77231146 2.79853030 1.95350499

H 1.04345066 0.08761226 2.52002662

H -0.5948206 0.9014863 2.6064505

H -1.6004705 -0.9561110 1.2983296

H -1.1855449 -0.0223153 -2.3859372

H 0.24609855 1.13031797 -2.55155824

H 1.74870103 -0.56056184 -1.56605598

H 1.17149162 -3.21890253 -3.75707385

H 1.25677567 -4.61122285 -2.64570260

H 2.45843539 -3.30428765 -2.52901063

H -1.6245277 -3.6090602 2.9867421

H -1.0014828 -4.8881057 1.9111480

H -2.1328249 -3.6600976 1.2870700

H 2.43379855 -2.31131346 2.35797674

H 1.89424038 -3.96404107 2.72646729

H 1.29511376 -2.59655563 3.70234573

H -2.1721019 -2.3319541 -1.2476427

H -1.7223835 -4.0160825 -1.5933194

H -1.7447232 -2.8188605 -2.9025225

**^3^CA2** (ORCA 502)

Fe -0.03492158 0.31073528 -0.17861032

Si 0.146943284 -2.924426934 1.141612725

Si 0.107945456 -2.649216629 -1.877572732

N -1.39494411 1.92340931 -0.11722188

N 1.355766690 1.841032907 -0.168102141

C -0.70890783 3.11555122 -0.27605292

C -2.74638550 1.98447731 0.01436212

C -3.44066657 3.21423070 0.00789260

C -2.77161872 4.40570117 -0.19700423

C -1.37481363 4.37926194 -0.35815903

C -0.62575139 5.57585746 -0.58890624

C 0.734763678 5.531664479 -0.678424525

C 1.440023796 4.293237589 -0.531329691

C 2.845367488 4.253172323 -0.528285500

C 3.466859526 3.034751297 -0.306835565

C 2.726269060 1.853152521 -0.128941419

C 0.723778506 3.071392177 -0.334309872

C -3.59671955 0.74600815 0.11690451

C -3.94063790 0.21840728 1.38704971

C -4.80283715 -0.88802818 1.44854763

C -5.36007393 -1.46756505 0.30376688

C -5.06105604 -0.88491127 -0.93519317

C -4.21235962 0.22486150 -1.05477372

C -3.50309972 0.89429171 2.68542977

C -3.10760993 -0.08606606 3.80282125

C -4.61728623 1.83802536 3.19229247

C -6.28714026 -2.67113461 0.41605994

C -7.69184827 -2.37311305 -0.14217916

C -5.68455591 -3.92008001 -0.25576234

C -4.08285489 0.91598004 -2.41464962

C -5.30671008 1.82564071 -2.66696913

C -3.92607971 -0.04888259 -3.60271493

C 3.540839738 0.612667240 0.125104328

C 4.178408058 -0.053061503 -0.959054818

C 5.028036555 -1.134544099 -0.683847273

C 5.307264421 -1.552246768 0.623615354

C 4.725559176 -0.836229835 1.675567253

C 3.862223162 0.249513249 1.459926854

C 4.057307185 0.437555883 -2.404807660

C 3.926341156 -0.691114471 -3.444061023

C 5.271216412 1.317374359 -2.781578040

C 6.246988516 -2.717850542 0.904128200

C 7.694262972 -2.392052792 0.485161194

C 5.766630054 -4.023948434 0.244159815

C 3.398706671 1.086291780 2.655122108

C 3.113080515 0.267751842 3.926702057

C 4.443795308 2.176993929 2.981819398

C -0.04687652 -0.07655633 1.85528831

C -0.58139555 -1.18957921 1.14416378

C -0.30646021 0.23234813 -2.24344473

C 0.550775329 -0.822432814 -1.836472613

O 0.589689939 -3.401021416 -0.430497235

C 1.068524370 -3.578581711 -3.225016991

C -1.17140866 -4.15267657 1.74534287

C 1.689104494 -3.102745117 2.220546141

C -1.74418135 -2.94262276 -2.14418420

H -4.52230151 3.19973427 0.13589202

H -3.30559684 5.35721421 -0.23703197

H -1.16791438 6.52022090 -0.67131797

H 1.318612935 6.439690920 -0.844057891

H 3.420187563 5.169678509 -0.671859730

H 4.553117170 2.971644173 -0.257715702

H -5.06681350 -1.30312804 2.42453638

H -5.52395539 -1.29041689 -1.83764687

H -2.62496097 1.51887898 2.45827393

H -3.97676125 -0.65162759 4.17133610

H -2.69670453 0.47271529 4.65729059

H -2.34718817 -0.80365908 3.46568394

H -4.88430350 2.59300947 2.43963415

H -4.28816210 2.36230192 4.10275265

H -5.52482006 1.26464564 3.43768709

H -6.39397802 -2.88919459 1.49227157

H -8.14190663 -1.49940699 0.35224940

H -8.35710227 -3.23601875 0.01439291

H -7.65423092 -2.17065347 -1.22360864

H -5.55882417 -3.76350656 -1.33831979

H -6.34542270 -4.78928701 -0.11528392

H -4.70026563 -4.16614963 0.16937418

H -3.18983528 1.55941276 -2.38443075

H -6.22300978 1.21956349 -2.74214150

H -5.18351792 2.37479812 -3.61326294

H -5.45147383 2.55968184 -1.86280448

H -3.08314482 -0.73880261 -3.46706585

H -3.75296390 0.52465199 -4.52570468

H -4.83449810 -0.65049149 -3.75656086

H 5.506637068 -1.651717079 -1.518463831

H 4.970967739 -1.125383516 2.700248643

H 3.154192887 1.064946570 -2.473596745

H 4.859615054 -1.266965834 -3.535593849

H 3.710312228 -0.260282751 -4.433219142

H 3.119483232 -1.391828865 -3.194324486

H 5.387674057 2.176524857 -2.108123642

H 5.158263576 1.700186447 -3.807654823

H 6.198054473 0.724368217 -2.739839781

H 6.243281766 -2.873006125 1.996410311

H 7.764532067 -2.237142163 -0.602720599

H 8.367675645 -3.221262438 0.751440807

H 8.058178338 -1.481122124 0.983209701

H 4.744670585 -4.279385823 0.560548531

H 6.429169818 -4.858807567 0.519325938

H 5.771546130 -3.939462338 -0.853510455

H 2.465758940 1.593699427 2.364174886

H 2.433999522 -0.574704875 3.736383057

H 2.650261906 0.914446846 4.687108664

H 4.038847685 -0.137508481 4.362572218

H 5.399354114 1.715179251 3.275925942

H 4.095232124 2.800958864 3.819480663

H 4.632352113 2.834266117 2.122299753

H 0.965138385 -0.143807447 2.260478386

H -0.68605171 0.64453624 2.36961389

H -1.67645418 -1.18857553 1.02952074

H -1.35200397 0.02706434 -2.48590960

H 0.087225752 1.150512302 -2.692248352

H 1.625368131 -0.622421298 -1.916491595

H 0.870317082 -3.149173623 -4.218813792

H 0.765393376 -4.637196811 -3.243845958

H 2.152803013 -3.538963106 -3.044309375

H -1.45556518 -3.93968394 2.78751614

H -0.79207768 -5.18540004 1.69897234

H -2.07977214 -4.09368643 1.12669533

H 2.479590564 -2.394981556 1.933024327

H 2.090671651 -4.123498408 2.124364027

H 1.444278990 -2.932375814 3.279926339

H -2.36568454 -2.32496254 -1.48015321

H -1.97869398 -4.00062703 -1.95010575

H -2.03084451 -2.72219276 -3.18337251

**^5^CA2**

Fe -0.08572700 0.42839800 -0.04787700

Si -0.30347900 -2.58334900 1.60437300

Si -0.11486000 -2.75808200 -1.43298400

N -1.43002000 2.08013900 -0.08980600

N 1.35577900 2.05508500 -0.09461300

C -0.73016700 3.28423800 -0.17924100

C -2.78386900 2.11511100 -0.02749700

C -3.50114900 3.29864500 -0.05974900

C -2.82141500 4.52289200 -0.18052800

C -1.43622300 4.53130000 -0.24322200

C -0.68245900 5.74736300 -0.36409300

C 0.67043200 5.73389000 -0.40757500

C 1.40370700 4.50412400 -0.32444400

C 2.78903600 4.46527100 -0.35836000

C 3.44534900 3.23271500 -0.25604600

C 2.70451900 2.06293500 -0.11586000

C 0.67899500 3.27090400 -0.20063300

C -3.48565100 0.79669500 0.06937900

C -3.78772700 0.24988300 1.33173400

C -4.32878200 -1.03650100 1.38959400

C -4.59485700 -1.77870400 0.24274000

C -4.38461800 -1.17319400 -0.99726700

C -3.84727200 0.10876400 -1.10905300

C -3.59361700 1.06197700 2.60990900

C -3.28399100 0.22767400 3.86152300

C -4.84612200 1.91683400 2.87899200

C -5.11961700 -3.19832200 0.35122000

C -6.55900300 -3.31165300 -0.17251300

C -4.19120700 -4.20097300 -0.35031700

C -3.78507400 0.81209700 -2.46137800

C -5.09061700 1.60041500 -2.68252800

C -3.55498300 -0.10591400 -3.66692000

C 3.44631800 0.76631000 0.02084000

C 3.81029500 0.03692500 -1.13198000

C 4.51719100 -1.15659600 -0.98039800

C 4.90229100 -1.63220800 0.27273100

C 4.57664000 -0.86954000 1.39060800

C 3.86694000 0.32972100 1.29319100

C 3.54428700 0.58570100 -2.53047000

C 3.24097900 -0.48465300 -3.58838700

C 4.74400800 1.43136700 -2.99583900

C 5.69502900 -2.91887200 0.41954800

C 7.05498700 -2.82341600 -0.28847300

C 4.90401800 -4.14082300 -0.06728200

C 3.66222000 1.18627400 2.53938400

C 3.44616900 0.37998000 3.82947600

C 4.86482600 2.12749700 2.73601600

C 0.37814900 0.18059800 2.06610600

C -0.58990600 -0.73759100 1.70970300

C -0.07652700 0.08610500 -2.13647800

C 0.51062400 -0.99429300 -1.46441600

O -0.66833500 -3.15785600 0.08840000

C 1.35545800 -3.85120300 -1.83857100

C -1.46755200 -3.43481800 2.79700500

C 1.49520600 -2.95429800 1.98677900

C -1.51193800 -3.06652100 -2.63164200

H -4.58007200 3.26717700 -0.00532900

H -3.36968600 5.45819700 -0.22447200

H -1.22877700 6.68407300 -0.42018000

H 1.23088100 6.65917700 -0.50167900

H 3.35299700 5.38715200 -0.45776900

H 4.52462100 3.17334300 -0.27559600

H -4.53683000 -1.48324200 2.35545900

H -4.65375100 -1.71293700 -1.89751100

H -2.75542000 1.74601200 2.44503400

H -4.14622100 -0.36750600 4.17399400

H -3.03885900 0.89283400 4.69347400

H -2.44162700 -0.44995700 3.72102700

H -5.06214200 2.58778000 2.04894100

H -4.71074100 2.52495600 3.77820300

H -5.71887300 1.27535000 3.03252300

H -5.13543100 -3.45217000 1.41774300

H -7.22711900 -2.62454300 0.35261800

H -6.94193900 -4.32782000 -0.04065200

H -6.61066000 -3.07437700 -1.23914100

H -4.20432900 -4.05792700 -1.43406700

H -4.51466000 -5.22693100 -0.15146200

H -3.15720600 -4.09144000 -0.01819400

H -2.96449600 1.53372600 -2.42629400

H -5.94470700 0.91794200 -2.72371400

H -5.05179500 2.15226000 -3.62630700

H -5.27065300 2.31632300 -1.88199600

H -2.66580800 -0.72512600 -3.56413900

H -3.43467600 0.49806000 -4.56995400

H -4.40409700 -0.77248500 -3.84113400

H 4.78712700 -1.72108300 -1.86511800

H 4.89585400 -1.21986200 2.36591000

H 2.67961300 1.25068200 -2.46836900

H 4.12391300 -1.08594800 -3.82195000

H 2.92894000 -0.00377500 -4.51911800

H 2.44261200 -1.15844000 -3.28086900

H 4.94326100 2.25635100 -2.31286700

H 4.55497800 1.85267400 -3.98753300

H 5.64590000 0.81523800 -3.05631500

H 5.88870600 -3.05603400 1.48965500

H 6.93243400 -2.71019800 -1.36926600

H 7.64682300 -3.72688200 -0.11562000

H 7.62847800 -1.96596700 0.07164400

H 3.96009700 -4.23942500 0.47244700

H 5.47822300 -5.05937300 0.08393700

H 4.67250600 -4.06749500 -1.13327200

H 2.77986800 1.81036100 2.37676600

H 2.68326900 -0.39253200 3.72451700

H 3.13397100 1.04865500 4.63566800

H 4.36720300 -0.10789900 4.15927100

H 5.78401100 1.55052700 2.87322500

H 4.72193000 2.75238300 3.62251200

H 5.00217300 2.78593600 1.87959200

H 1.41386400 -0.11355300 2.14675800

H 0.13592200 1.15554900 2.47896600

H -1.62356800 -0.43381400 1.81849600

H -1.08065400 0.00108900 -2.53155100

H 0.51060900 0.89379200 -2.55952500

H 1.58719100 -0.93058000 -1.30871000

H 1.70312300 -3.68700500 -2.86312900

H 1.10185400 -4.91054800 -1.73852000

H 2.19622000 -3.64655400 -1.17202400

H -1.28650500 -3.12247000 3.82942900

H -1.35371400 -4.52146100 2.74927700

H -2.50643700 -3.19853000 2.55346100

H 2.17928500 -2.40688200 1.33324700

H 1.69761100 -4.02109000 1.85445200

H 1.75445600 -2.69530000 3.01714500

H -2.40939000 -2.52291800 -2.33894200

H -1.76401300 -4.13048600 -2.64627000

H -1.24365400 -2.77173600 -3.64965000

**^3^CC**

C 3.37598000 -0.30256400 -0.06986900

N 1.42662800 1.12088400 -0.09513200

C 2.75101400 1.04738100 -0.11480400

C 3.53744200 2.21954500 -0.18254800

C 2.93136600 3.45124600 -0.22927500

C 0.81641200 2.32751800 -0.13807400

C 1.52318700 3.53799500 -0.20831300

C 0.79651700 4.77366000 -0.25500600

C -0.56009300 4.77927900 -0.23587200

C -3.34030600 2.28748500 -0.09690600

C -2.58843500 1.09259600 -0.04842500

N -1.25497500 1.12487400 -0.04325400

C -0.62198200 2.33134500 -0.11266200

C -1.30658000 3.55479700 -0.16680700

C -2.71513400 3.50637200 -0.15019400

C -3.29538000 -0.21627700 -0.03930900

C 3.55716000 -1.01280300 -1.26563400

C 4.13553500 -2.27861100 -1.20063500

C 4.52586800 -2.84468100 0.01099100

C 4.34675400 -2.10669200 1.18062800

C 3.77646100 -0.83620500 1.16530900

C -3.78242500 -0.74308100 1.16650100

C -4.44233100 -1.96961500 1.12903900

C -4.64106300 -2.66055400 -0.06501900

C -4.19733300 -2.08064700 -1.25195300

C -3.53027300 -0.85898100 -1.26468900

C 3.58013300 -0.06574800 2.44690400

C 3.11429000 -0.44199000 -2.58897500

C 5.10411000 -4.23605900 0.06255900

C -3.10501100 -0.22966600 -2.56729000

C -3.62506200 0.00409300 2.46638200

C -5.30903500 -4.01190500 -0.07050500

H 4.61704000 2.11881800 -0.19905000

H 3.52407000 4.36044600 -0.28241500

H 1.35309100 5.70471500 -0.30704800

H -1.10882900 5.71567400 -0.27295900

H -4.42146900 2.20538800 -0.09490800

H -3.28831500 4.42849000 -0.18632800

H 4.27435800 -2.83926900 -2.12219000

H 4.65310300 -2.53402700 2.13282200

H -4.81056700 -2.39596000 2.05951900

H -4.36843800 -2.59450400 -2.19494700

H 4.02652000 -0.59699600 3.29170900

H 4.03944200 0.92881800 2.39556600

H 2.51284800 0.06541500 2.65939500

H 2.02341500 -0.50179200 -2.67736500

H 3.40907600 0.60755500 -2.70332700

H 3.54748500 -1.00588700 -3.41963600

H 5.58957000 -4.50231200 -0.88140800

H 5.84155200 -4.33323300 0.86573500

H 4.31471800 -4.97411100 0.24788000

H -3.37457700 -0.86967500 -3.41139800

H -3.58854200 0.74469600 -2.71374700

H -2.02135700 -0.08466500 -2.59788600

H -2.56812700 0.16074000 2.70388500

H -4.11325500 0.98584300 2.42125800

H -4.07565400 -0.55416000 3.29136900

H -6.05020600 -4.09618700 0.73066900

H -5.81093600 -4.20566700 -1.02360300

H -4.56757600 -4.80564300 0.07960800

Cl -0.11686700 -0.60359600 2.42787200

Cl -0.16472100 -1.77824200 -1.57456200

Fe -0.03245100 -0.40249800 0.18607400

**^5^CC**

C -3.34295600 -0.19132200 -0.02668800

N -1.35304000 1.18509800 -0.02647600

C -2.68094700 1.14183500 -0.03158500

C -3.44906700 2.32764600 -0.04752800

C -2.82285400 3.54848400 -0.05940100

C -0.72246800 2.38444800 -0.04065900

C -1.41331300 3.60796300 -0.05738900

C -0.67805700 4.84009200 -0.07220500

C 0.67806600 4.84009300 -0.07227100

C 3.44908300 2.32765200 -0.04776500

C 2.68096600 1.14184100 -0.03168600

N 1.35305900 1.18510000 -0.02649900

C 0.72248400 2.38444900 -0.04069900

C 1.41332600 3.60796500 -0.05750700

C 2.82286600 3.54848900 -0.05963500

C 3.34297200 -0.19131900 -0.02666900

C -3.69970000 -0.77903500 1.19650000

C -4.29569800 -2.03718500 1.17435000

C -4.54400700 -2.70993500 -0.02151600

C -4.20620600 -2.08542900 -1.22023000

C -3.60685500 -0.82820200 -1.24765100

C 3.60703700 -0.82825300 -1.24756400

C 4.20636700 -2.08548900 -1.22000400

C 4.54397500 -2.70995400 -0.02121700

C 4.29549100 -2.03715500 1.17458800

C 3.69951600 -0.77899500 1.19659900

C -3.24992700 -0.17474100 -2.55886000

C -3.44006000 -0.07205400 2.50289900

C -5.14267400 -4.09352300 -0.01315000

C 3.43969900 -0.07194800 2.50292600

C 3.25026200 -0.17486500 -2.55885100

C 5.14262400 -4.09354800 -0.01268500

H -4.53026500 2.24422000 -0.05109300

H -3.39865600 4.47002500 -0.07169500

H -1.23154000 5.77449500 -0.08380000

H 1.23154600 5.77449700 -0.08392100

H 4.53028000 2.24422700 -0.05141300

H 3.39866500 4.47003100 -0.07201200

H -4.56389100 -2.50828500 2.11731200

H -4.40318600 -2.59320600 -2.16151900

H 4.40347300 -2.59330800 -2.16124400

H 4.56352600 -2.50822600 2.11760900

H -3.62398400 -0.76425200 -3.40003600

H -3.67541500 0.83318400 -2.63724100

H -2.16303400 -0.09687800 -2.66854800

H -2.36411500 0.03936600 2.67498800

H -3.89388700 0.92638800 2.51821700

H -3.85179200 -0.64082000 3.34072100

H -5.87405300 -4.20824800 0.79345100

H -5.64073200 -4.31910900 -0.96107800

H -4.36239400 -4.84865000 0.13888000

H 3.85134200 -0.64065800 3.34082900

H 3.89350400 0.92650400 2.51823500

H 2.36373200 0.03946000 2.67488700

H 2.16337900 -0.09701900 -2.66865600

H 3.67574600 0.83306000 -2.63724300

H 3.62441800 -0.76442000 -3.39995200

H 5.64049900 -4.31933700 -0.96065900

H 5.87415900 -4.20811200 0.79380000

H 4.36236800 -4.84863500 0.13966000

Cl 0.00017600 -1.65366500 -1.86878600

Cl -0.00016100 -1.27034900 2.19768900

Fe 0.00001000 -0.54028800 0.07516600

**^3^CC1**

Fe -0.12051800 -0.37682600 -0.21240400

C 4.80507500 -2.57826200 0.22748900

C 2.67503000 1.13713400 0.04602600

C 4.67171800 -1.89262900 -0.97884200

C -3.59589800 -0.85096900 1.29827900

C -4.71403000 -2.64221800 0.08875200

C -4.48601400 -1.95766400 -1.10272400

N 1.33978900 1.15842000 0.00923100

C -3.82701900 -0.72813200 -1.12615200

C 3.48629600 -0.84837000 1.32128000

C -4.26863400 -2.06905100 1.28046700

C 0.71640700 2.37182800 0.03722100

C 3.97127600 -0.69056100 -1.06254700

C -0.71792600 2.37050200 0.05436600

C 4.21046700 -2.03789600 1.36712500

C 1.41296300 3.59746900 0.06642700

N -1.33952600 1.15108600 0.02386600

C -2.68289600 1.13058600 0.08499200

C 3.43937200 2.31704700 0.07223300

C 3.38010800 -0.17364500 0.09723700

C -1.41737800 3.59221100 0.10908100

C 2.81580000 3.54508600 0.07411500

C 0.67483400 4.82850000 0.10064400

C -0.67963900 4.82484000 0.12336800

C -2.81945700 3.54141800 0.15423300

C 0.62828000 -2.25961700 0.07981200

C -3.43877200 2.30921400 0.15025400

C -0.47104600 -1.43273200 -1.97074200

C -3.37678600 -0.18609300 0.08303900

C 0.68541000 -1.91329600 -1.29136400

C -0.58615000 -2.12623700 0.81189700

H 5.11961900 -2.30706900 -1.87961800

H -4.82679700 -2.39121400 -2.04060000

H -4.43472900 -2.59373100 2.21928700

H 4.29835800 -2.56504400 2.31496200

H 4.52064600 2.23317000 0.09988800

H 3.39212500 4.46603600 0.09526700

H 1.22762400 5.76364300 0.11519900

H -1.23531900 5.75777900 0.15661900

H -3.39606100 4.46090800 0.19780300

H 1.56778900 -2.44453000 0.59471700

H -4.51930200 2.22257100 0.19521200

H -0.32864000 -1.04705000 -2.97993500

H 1.66685700 -1.84928800 -1.75626500

H -0.53401300 -2.27295000 1.89058200

H -1.52756500 -2.46519500 0.37981100

H -1.43173600 -1.93050800 -1.83108200

C 2.77710500 -0.33353500 2.54788100

H 1.69215400 -0.42759700 2.41482200

H 3.06391600 -0.90149500 3.43732100

H 2.99298500 0.72471900 2.73275500

C 5.53693300 -3.89507400 0.29158500

H 6.30628400 -3.96380600 -0.48374100

H 6.01856400 -4.03760800 1.26417300

H 4.84174600 -4.72999300 0.14256900

C 3.81278300 0.01821200 -2.38531500

H 4.23207200 -0.57636000 -3.20168700

H 2.75325300 0.19605500 -2.60258000

H 4.31333700 0.99350200 -2.38677800

C -3.06475700 -0.28033300 2.58769400

H -3.44692500 0.73016300 2.77356600

H -3.33760800 -0.90989300 3.43941100

H -1.97126400 -0.21984500 2.53952900

C -5.39870000 -3.98595400 0.09597000

H -6.14419800 -4.04942800 0.89586400

H -5.90280400 -4.18284500 -0.85501600

H -4.67178200 -4.79026800 0.26004600

C -3.58820000 -0.00411100 -2.42713600

H -3.89423400 -0.61804500 -3.27904000

H -4.14732500 0.93832300 -2.47113000

H -2.52473300 0.23182800 -2.54095500

**^3^CC1** (b3lyp-D3(BJ)/6-311g*)

Fe -0.11577500 -0.33442600 -0.25186300

C 4.76059800 -2.60911700 0.32616300

C 2.66803200 1.12680000 0.01879700

C 4.71756500 -1.91577000 -0.88141200

C -3.57301400 -0.90774300 1.31025100

C -4.65547300 -2.69374900 0.05793300

C -4.45317100 -1.96829800 -1.11488100

N 1.32025100 1.14443900 -0.00822300

C -3.82385400 -0.72230700 -1.10803000

C 3.38785700 -0.86703700 1.33520000

C -4.21639100 -2.14184100 1.26235500

C 0.69869900 2.37382800 0.03929500

C 4.03581900 -0.70416200 -1.00902300

C -0.71890200 2.37489500 0.09298900

C 4.09337700 -2.06445900 1.42526300

C 1.40786200 3.60079000 0.05539300

N -1.34336300 1.14346400 0.06058900

C -2.69466000 1.12026700 0.14304300

C 3.42726800 2.30207500 0.02372600

C 3.36539500 -0.18482200 0.10641500

C -1.43118400 3.59603900 0.17170400

C 2.80731000 3.54270900 0.03023300

C 0.66689700 4.82645200 0.11428700

C -0.69061800 4.82392300 0.17394900

C -2.82882700 3.53506700 0.24821700

C 0.61435200 -2.24461200 -0.08459000

C -3.44668300 2.29105500 0.24078500

C -0.40799400 -1.23215300 -2.06796000

C -3.37213900 -0.20366700 0.11295600

C 0.73225800 -1.78592600 -1.41434100

C -0.64096100 -2.13902100 0.58148900

H 5.22139200 -2.32947700 -1.75056100

H -4.79048900 -2.38139900 -2.06147500

H -4.36397600 -2.69522800 2.18585600

H 4.11180400 -2.59386200 2.37412600

H 4.50726900 2.21900700 0.04393200

H 3.39024800 4.45780100 0.03925800

H 1.21710700 5.76203400 0.11963500

H -1.24160700 5.75749000 0.22712400

H -3.41128800 4.44777200 0.31435300

H 1.52180800 -2.47108800 0.46306400

H -4.52488600 2.20405300 0.30211900

H -0.23900600 -0.71439700 -3.00936400

H 1.72496800 -1.68344300 -1.83718200

H -0.65098800 -2.32338000 1.65330300

H -1.55701500 -2.45255500 0.08974100

H -1.36702000 -1.74186600 -2.02165400

C 2.63788100 -0.32915000 2.52696600

H 1.56304300 -0.30962400 2.32835500

H 2.81031300 -0.94349400 3.41180800

H 2.93453100 0.69714800 2.76124100

C 5.47506000 -3.93131900 0.44032000

H 6.17997100 -4.07923700 -0.38033600

H 6.03011700 -4.00747600 1.37893900

H 4.76431700 -4.76406200 0.41675000

C 4.00271200 0.01296800 -2.33649100

H 4.33658800 -0.64251200 -3.14274100

H 2.99574900 0.36099800 -2.57843300

H 4.65069100 0.89477900 -2.33755900

C -3.06619800 -0.35247000 2.61563600

H -3.52183800 0.61436700 2.84869500

H -3.27604700 -1.03301900 3.44241600

H -1.98565200 -0.19475900 2.57065200

C -5.29691000 -4.05750300 0.02690100

H -5.94476900 -4.21633100 0.89268600

H -5.89778300 -4.19922800 -0.87398400

H -4.53770800 -4.84675900 0.04146000

C -3.63511200 0.04932000 -2.38975400

H -3.85414400 -0.57376100 -3.25859400

H -4.29446900 0.92213500 -2.43022800

H -2.61039400 0.41274800 -2.48422000

**^3^CC1** (TPSSh/6-31g*-TZVP(Fe))

Fe 0.00867900 -0.33122500 -0.34237300

N 1.30218900 1.13559800 0.03676400

N -1.36314300 1.14967100 0.01040200

C 3.37469500 -0.19133700 0.13851700

C 3.46876500 -0.90925900 1.35239400

C 4.18621800 -2.10885100 1.37561000

H 4.26066200 -2.65801800 2.31310900

C 4.80760700 -2.62209900 0.22848500

C 4.71111000 -1.88808200 -0.95754700

H 5.19487100 -2.26404400 -1.85776300

C 4.01586800 -0.67151200 -1.02094100

C 2.66686800 1.12406500 0.11132300

C 3.41720500 2.30058100 0.17576500

H 4.49808900 2.21642100 0.23177600

C 2.79647400 3.55488600 0.17979700

H 3.38002500 4.46991500 0.22958600

C 1.39687800 3.60835400 0.14104500

C 0.65048900 4.83592000 0.18003000

H 1.20402300 5.77113200 0.21526500

C -0.71477100 4.83943600 0.18939400

H -1.26453600 5.77646800 0.23313500

C -1.45973400 3.61221800 0.15425800

C -2.85869700 3.54935700 0.21132000

H -3.44475900 4.46233100 0.27273300

C -3.47864100 2.29479900 0.21603400

H -4.55791300 2.20763300 0.29415100

C -2.71880500 1.12431300 0.12166100

C -0.73653400 2.38628100 0.07747500

C 0.67644400 2.38095600 0.08004300

C -3.39293500 -0.20880000 0.16079400

C -4.08299700 -0.68940000 -0.97141600

C -4.72997400 -1.92962500 -0.89586400

H -5.25390900 -2.30337600 -1.77418600

C -4.72505300 -2.69562300 0.27565800

C -4.04947000 -2.18850400 1.39322500

H -4.04456600 -2.76232600 2.31866200

C -3.37769500 -0.96087600 1.35650900

C -4.13276900 0.11137000 -2.25544300

H -4.78628600 0.98696400 -2.15712400

H -3.14091300 0.48395600 -2.53411800

H -4.51609900 -0.50006800 -3.07877100

C -2.65477000 -0.45454800 2.58502300

H -2.98887300 0.55346100 2.85780100

H -2.82767900 -1.11711900 3.43881300

H -1.57390100 -0.39671200 2.40844800

C 2.80556400 -0.39179300 2.60785700

H 3.02125500 -1.04304000 3.46086900

H 3.14810600 0.62093000 2.85241900

H 1.71857800 -0.34263400 2.47379300

C 3.98842000 0.11226200 -2.31612400

H 4.22113600 -0.53683400 -3.16690500

H 3.01070100 0.57257800 -2.48828400

H 4.73020700 0.92150800 -2.30487500

C 5.54522100 -3.94107100 0.27402500

H 4.84326200 -4.78515200 0.29019000

H 6.19280000 -4.06599800 -0.59991100

H 6.16695500 -4.01948000 1.17340500

C -5.40909400 -4.04286000 0.32769600

H -5.80343000 -4.25137300 1.32817100

H -6.23854700 -4.09718200 -0.38532600

H -4.70782400 -4.85011400 0.07781800

C 0.51120400 -1.10192000 -2.28818300

C -0.62386900 -1.73662600 -1.74282300

H 0.40216000 -0.54233800 -3.21355900

H 1.51067000 -1.49254600 -2.10399700

H -1.59593200 -1.61511200 -2.21247900

C -0.59594800 -2.32148900 -0.42657900

C 0.55429100 -2.30645400 0.38954200

H -1.55512200 -2.57834500 0.01634800

H 0.45570200 -2.63973500 1.41912000

H 1.55270500 -2.41122800 -0.02812300

**^5^CC1**

Fe -0.02129800 -0.38383500 -0.16597800

N 1.32897400 1.18744600 0.01282000

N -1.38049100 1.20367300 -0.02813000

C 3.36058500 -0.14439100 0.09057000

C 3.55445200 -0.80490200 1.31187600

C 4.21006900 -2.03588400 1.31392800

H 4.35898500 -2.55224500 2.26019400

C 4.67169300 -2.62187000 0.13612800

C 4.47824500 -1.93799800 -1.06380900

H 4.83875500 -2.37801100 -1.99154600

C 3.83329400 -0.70220700 -1.10585000

C 2.67314500 1.17959900 0.06955100

C 3.43234700 2.34166500 0.11754000

H 4.51251200 2.26214800 0.16569700

C 2.78904700 3.58959700 0.10459400

H 3.36621600 4.50994400 0.13762200

C 1.40780500 3.64303700 0.05716700

C 0.65960600 4.87631100 0.05353500

H 1.21432900 5.81095400 0.07524300

C -0.69220500 4.88226500 0.02987300

H -1.23952000 5.82142600 0.03253900

C -1.44919400 3.65313400 0.00947200

C -2.83188300 3.60435100 0.01400200

H -3.40625400 4.52675200 0.02505500

C -3.48433000 2.35435100 0.02032500

H -4.56585300 2.28163800 0.04692300

C -2.72782100 1.19546000 0.00375200

C -0.72348500 2.42262800 -0.00486000

C 0.67808000 2.41427500 0.01899700

C -3.38207600 -0.14456300 0.04597500

C -3.74481300 -0.78524300 -1.14706000

C -4.33859500 -2.04509800 -1.08159500

H -4.61840800 -2.54678800 -2.00609500

C -4.58322000 -2.67652700 0.13825000

C -4.21847300 -2.01576000 1.31069900

H -4.40084200 -2.49479300 2.27062500

C -3.61162800 -0.75989200 1.28470700

C -3.47102300 -0.13006800 -2.47823200

H -3.98241600 0.83483000 -2.55928300

H -2.39968000 0.06837800 -2.60071200

H -3.79920600 -0.76528800 -3.30612500

C -3.17363100 -0.09222000 2.56402800

H -3.60782500 0.90839900 2.66110700

H -3.46519900 -0.68225100 3.43765000

H -2.08431600 0.03094600 2.58036600

C 3.04001200 -0.20505100 2.59583800

H 3.28598000 -0.83774900 3.45350200

H 3.46619000 0.78964100 2.76660800

H 1.95155300 -0.08547900 2.55410600

C 3.62846100 0.01417800 -2.41725800

H 3.96709700 -0.59846500 -3.25780600

H 2.56969600 0.25224200 -2.56777200

H 4.17560500 0.96293000 -2.44085500

C 5.33804500 -3.97474100 0.15522300

H 4.60255200 -4.77296700 -0.00255200

H 6.09101700 -4.06054900 -0.63462100

H 5.82846800 -4.16399000 1.11514700

C -5.19859900 -4.05278300 0.18385000

H -5.68127500 -4.24173500 1.14753900

H -5.94890200 -4.18051600 -0.60309800

H -4.43525000 -4.82688500 0.03874100

C 0.60509900 -1.71868700 -1.82711800

C -0.54680800 -2.17075700 -1.18872500

H 0.54981000 -1.45779300 -2.87939300

H 1.59976400 -1.95434900 -1.45579100

H -1.48512900 -2.18411800 -1.73609100

C -0.64467500 -2.36784200 0.23542600

C 0.40511000 -2.13028100 1.12169200

H -1.64765300 -2.50444500 0.63125100

H 0.20312600 -2.17023000 2.18765200

H 1.44463800 -2.28421900 0.84181200

**^5^CC1** (b3lyp-D3(BJ)/6-311g*)

Fe 0.10124600 -0.33666400 -0.20100100

N 1.39011600 1.18027500 0.04259000

N -1.31517100 1.20580300 -0.02373500

C 3.36881300 -0.20390100 0.12256500

C 3.46669500 -0.91793300 1.32849400

C 4.03607300 -2.19062900 1.31316300

H 4.10950500 -2.74534300 2.24472800

C 4.50325200 -2.77223700 0.13339100

C 4.40164500 -2.04003700 -1.04872400

H 4.76051100 -2.47577500 -1.97707800

C 3.84526500 -0.75981000 -1.07412700

C 2.74121200 1.14760700 0.11402400

C 3.50850000 2.29766200 0.17949500

H 4.58624900 2.20993100 0.23773800

C 2.87797600 3.55952100 0.16909600

H 3.46654200 4.46992700 0.21596500

C 1.49138000 3.63301700 0.10601600

C 0.75464700 4.86824200 0.09929000

H 1.31369800 5.79848300 0.13334600

C -0.60216900 4.88462400 0.05772000

H -1.13974400 5.82810700 0.05827800

C -1.36479100 3.66802600 0.02151300

C -2.75336800 3.62641400 0.00942200

H -3.32239600 4.55075600 0.01715700

C -3.40878400 2.38454100 0.00901200

H -4.48947900 2.31900100 0.02873400

C -2.65923300 1.21155600 0.00052200

C -0.65198900 2.42884700 0.01084300

C 0.74938300 2.41513200 0.05157800

C -3.34570800 -0.10972700 0.06276600

C -3.82398700 -0.71225700 -1.10852700

C -4.46572700 -1.94859100 -1.01772100

H -4.82693300 -2.42250700 -1.92639300

C -4.65458400 -2.58840600 0.20704100

C -4.17896100 -1.96166800 1.36018100

H -4.31539700 -2.44659900 2.32314300

C -3.51876700 -0.73433500 1.30855000

C -3.63274200 -0.04437600 -2.44746000

H -4.24371200 0.85836200 -2.53405500

H -2.59517500 0.26705700 -2.59186600

H -3.90599600 -0.71411000 -3.26505000

C -2.96812700 -0.10650000 2.56355100

H -3.32197700 0.92007700 2.68760700

H -3.25372500 -0.67716800 3.44871100

H -1.87617700 -0.05822600 2.52719100

C 2.94131400 -0.32788000 2.61185400

H 3.16110800 -0.97456000 3.46284200

H 3.37621500 0.65594800 2.80528600

H 1.85808500 -0.18748900 2.56173500

C 3.74372400 0.00341900 -2.37081700

H 3.98018000 -0.63665500 -3.22276700

H 2.73938000 0.40782500 -2.51479600

H 4.43024300 0.85434900 -2.38714000

C 5.07178600 -4.16835600 0.13701000

H 4.27300200 -4.91746400 0.12011800

H 5.70488100 -4.34667900 -0.73468100

H 5.67047000 -4.35498800 1.03201100

C -5.32113600 -3.93846300 0.28324800

H -5.91735000 -4.04018000 1.19319600

H -5.97911600 -4.11048900 -0.57121800

H -4.57867200 -4.74372500 0.29071700

C 0.53710300 -1.53537900 -1.96002300

C -0.62785600 -2.03315200 -1.38153900

H 0.51178200 -1.20697800 -2.99210300

H 1.51989800 -1.83017400 -1.60037200

H -1.55259500 -1.98313800 -1.94504200

C -0.76416300 -2.34876300 0.00461800

C 0.23638100 -2.19301600 0.96016700

H -1.77910800 -2.50759100 0.34994600

H -0.00877300 -2.35456900 2.00304200

H 1.28920300 -2.31385400 0.71793900

**^5^CC1** (TPSSh/6-31g*-TZVP(Fe))

Fe -0.03673000 -0.40007600 -0.10575800

N 1.32937200 1.14342300 0.00845700

N -1.39688000 1.16606400 0.00766800

C 3.41834800 -0.16050600 0.04527400

C 3.69956800 -0.78528300 1.27934800

C 4.43662400 -1.97585900 1.28462500

H 4.64983900 -2.45690100 2.23805400

C 4.90959400 -2.55796300 0.10173700

C 4.64214800 -1.90525400 -1.10693500

H 5.01461600 -2.33196300 -2.03695700

C 3.91283000 -0.70948800 -1.15620200

C 2.68741300 1.14532900 0.02525700

C 3.43997500 2.32216600 0.03846000

H 4.52224500 2.24753400 0.05264900

C 2.79622500 3.57221100 0.03980700

H 3.37007500 4.49523000 0.05088200

C 1.40206000 3.61693900 0.03406300

C 0.65786400 4.84733300 0.04448600

H 1.21479100 5.78142900 0.05222900

C -0.70539000 4.85493000 0.04763900

H -1.25261900 5.79466200 0.05819100

C -1.45766000 3.62954800 0.04007500

C -2.85304500 3.58627100 0.05362000

H -3.42386000 4.51107300 0.06886300

C -3.50583900 2.33699000 0.05386000

H -4.58849800 2.26851900 0.07336600

C -2.75369600 1.16291600 0.03213400

C -0.73443900 2.39353100 0.02160300

C 0.67476400 2.38235700 0.02045800

C -3.44510500 -0.16415600 0.04128800

C -3.85890600 -0.74941100 -1.17337500

C -4.53468700 -1.97640300 -1.14073200

H -4.85422700 -2.42622500 -2.07955800

C -4.81622900 -2.63214900 0.06385300

C -4.40798900 -2.02425400 1.25802900

H -4.62904700 -2.51143400 2.20658000

C -3.72599600 -0.80078800 1.26909200

C -3.58386200 -0.06576100 -2.49520300

H -4.11000600 0.89366700 -2.56367600

H -2.51550300 0.14936900 -2.61437700

H -3.90551800 -0.69232400 -3.33321500

C -3.29834500 -0.17895400 2.58021200

H -3.72067800 0.82539500 2.70086400

H -3.62132700 -0.79269700 3.42699100

H -2.20800900 -0.07264000 2.62864400

C 3.22560900 -0.17565000 2.57945800

H 3.50257600 -0.80613600 3.43037100

H 3.66332800 0.81840800 2.73188100

H 2.13709900 -0.04982700 2.57977400

C 3.67911000 -0.01718300 -2.48120700

H 3.97337300 -0.66283000 -3.31497700

H 2.62581400 0.25519700 -2.60748400

H 4.26024300 0.91065800 -2.55037800

C 5.67195200 -3.86322900 0.13015500

H 4.98549300 -4.72041900 0.13195900

H 6.32136800 -3.96477700 -0.74577400

H 6.29407900 -3.94242100 1.02847000

C -5.52368500 -3.96833300 0.07571600

H -6.14553300 -4.08204600 0.97028500

H -6.16461800 -4.08790800 -0.80416700

H -4.80238900 -4.79641600 0.07162000

C 0.58927000 -1.75683900 -1.73572400

C -0.54529500 -2.21232300 -1.05567400

H 0.50457600 -1.51729800 -2.79169400

H 1.59494400 -1.97772800 -1.38678000

H -1.49872400 -2.24749100 -1.57615600

C -0.59261400 -2.37051900 0.38299000

C 0.49244300 -2.08942700 1.22182500

H -1.57885400 -2.50544800 0.81983700

H 0.33346000 -2.09467700 2.29624700

H 1.52093000 -2.24072400 0.90420700

**^1^Int-1**

Fe 0.02134300 -0.27720300 0.34994500

N 1.42260200 1.14471700 0.10734200

N -1.17616700 1.20585100 -0.05668700

C 3.38437200 -0.29860200 0.12443900

C 3.56124200 -1.07405900 1.27578200

C 4.15549300 -2.33232700 1.14911300

H 4.28891000 -2.93833400 2.04330500

C 4.58130900 -2.82617000 -0.07859400

C 4.43360800 -2.01399800 -1.20455700

H 4.78457900 -2.37299300 -2.17037200

C 3.84895800 -0.75504300 -1.12261100

C 2.75518700 1.05240500 0.17087700

C 3.56982200 2.19970400 0.20006600

H 4.64408100 2.06362000 0.26708900

C 3.01540600 3.45891000 0.12888900

H 3.63965100 4.34795800 0.14606800

C 1.62056700 3.57716300 0.01586100

C 0.92404100 4.82690400 -0.10756000

H 1.50243300 5.74658500 -0.10550800

C -0.42623200 4.86069300 -0.23411100

H -0.94682000 5.80858700 -0.33761700

C -1.20490900 3.65244800 -0.23549500

C -2.60148700 3.62369900 -0.37382600

H -3.16055200 4.54763900 -0.49032100

C -3.23345400 2.40159300 -0.36841500

H -4.30926200 2.32806900 -0.48818100

C -2.50951400 1.20465300 -0.20861500

C -0.54613700 2.41915000 -0.09173500

C 0.87471500 2.38509900 0.01859700

C -3.27112200 -0.07498100 -0.24886300

C -3.34049700 -0.78982400 -1.45440200

C -4.09872900 -1.95665500 -1.49701600

H -4.13966700 -2.52408500 -2.42475800

C -4.80389600 -2.41399400 -0.38379400

C -4.74439200 -1.66600500 0.78983900

H -5.28850100 -2.00727300 1.66844400

C -3.99094900 -0.49449400 0.87651000

C -2.62887400 -0.28955400 -2.68456500

H -2.64949100 -1.03905800 -3.48066900

H -3.10131900 0.62436400 -3.06639200

H -1.58353300 -0.06021400 -2.46120300

C -3.93523800 0.28378500 2.16745100

H -2.89684300 0.43948600 2.48023200

H -4.39946800 1.27176900 2.06141300

H -4.45930500 -0.24826400 2.96689400

C 3.14129200 -0.59866200 2.64313700

H 2.17926500 -1.03853100 2.92407800

H 3.88453200 -0.88768000 3.39354300

H 3.01944700 0.48741900 2.68141900

C 3.74213900 0.11366300 -2.35291000

H 3.94108900 -0.46602200 -3.25886700

H 2.74592300 0.55599700 -2.44666300

H 4.46436800 0.93851300 -2.31867600

C 5.17745600 -4.20560900 -0.20145800

H 4.41396400 -4.93198400 -0.50477300

H 5.97145200 -4.23158900 -0.95490600

H 5.59842100 -4.54708800 0.74928700

C -5.63250400 -3.67200800 -0.46102500

H -5.13757800 -4.43603600 -1.06948500

H -5.81140900 -4.09438300 0.53257800

H -6.60978500 -3.47303600 -0.91765900

C -0.58832900 -1.93569400 -0.40600700

H -1.25119600 -2.76796100 -0.20852900

C 0.22368200 -1.40558700 -1.24685600

C 0.81040700 -1.59792800 -2.60111700

H 0.27026700 -2.36039800 -3.17768400

H 0.80523500 -0.66681600 -3.18053100

H 1.85515200 -1.91813200 -2.52033300

C -1.47920500 -2.16605300 2.52781600

H -0.90256800 -3.08074100 2.34157700

H -2.42698700 -2.27623300 1.99127100

H -1.68803500 -2.10253400 3.60259900

C -0.72516700 -0.98759800 2.04765600

C -0.03516900 0.04990200 2.29881400

H 0.33023400 0.73313400 3.05230400

**^1^Int-1ʹ**

Fe -0.02020300 -0.13298500 -0.50199600

N 1.19090200 1.26867600 0.00738700

N -1.35666400 1.16140100 0.01585000

C 3.17704800 -0.09828000 0.19748800

C 3.21462000 -0.77988600 1.42076100

C 3.79995400 -2.04310000 1.46560200

H 3.82780200 -2.57830100 2.41268600

C 4.33475900 -2.64215300 0.32637500

C 4.26188900 -1.95140900 -0.88203800

H 4.65224600 -2.41579100 -1.78570400

C 3.68132400 -0.68778200 -0.96875600

C 2.53022100 1.24093000 0.13125100

C 3.26918100 2.42660400 0.20008100

H 4.34644300 2.35370500 0.30535600

C 2.64402700 3.66599500 0.12401900

H 3.22615000 4.58247700 0.16277200

C 1.24963700 3.71904800 0.01852900

C 0.44809700 4.91767800 -0.02036200

H 0.95537200 5.87847700 -0.02299900

C -0.90951300 4.86195100 -0.03247700

H -1.49213000 5.77913600 -0.04519200

C -1.61410600 3.60333900 -0.00137100

C -3.00143000 3.44317400 0.08386000

H -3.65521900 4.31020500 0.11143000

C -3.52607200 2.15674600 0.15809400

H -4.59567900 2.00111200 0.25390000

C -2.69569800 1.03375100 0.11703500

C -0.84212900 2.42776600 -0.01670600

C 0.57295100 2.48652600 -0.01320600

C -3.22478400 -0.35499500 0.19542400

C -3.80128100 -0.94900400 -0.93617700

C -4.26467100 -2.25937000 -0.84358400

H -4.70895900 -2.72591900 -1.72068100

C -4.15711300 -2.99128900 0.33795000

C -3.56662400 -2.38223900 1.44320400

H -3.46745900 -2.94199600 2.37114900

C -3.08750300 -1.07490900 1.39041000

C -3.86401800 -0.20694200 -2.24840100

H -4.32552400 -0.82312900 -3.02520400

H -4.43434800 0.72504000 -2.17052600

H -2.85323400 0.05694800 -2.58212400

C -2.39717400 -0.46441200 2.58291300

H -2.80756800 0.52188800 2.82701200

H -2.49408100 -1.10369400 3.46513000

H -1.33148700 -0.33093400 2.36292400

C 2.57399100 -0.18464700 2.64867000

H 2.76025900 -0.80312900 3.53119100

H 2.94743600 0.82494800 2.85401900

H 1.49005100 -0.10955100 2.50090500

C 3.51861800 -0.00805400 -2.30378400

H 4.00980300 -0.57540900 -3.09953900

H 2.45072700 0.06494900 -2.54519100

H 3.92944100 1.00749200 -2.30435200

C 4.98822800 -3.99925600 0.40046100

H 4.86563000 -4.55027100 -0.53737600

H 6.06475800 -3.90589100 0.58946300

H 4.56272900 -4.60326600 1.20789200

C -4.63495900 -4.42039900 0.40212100

H -5.56754000 -4.55556800 -0.15564200

H -3.89125200 -5.09971900 -0.03171000

H -4.80856600 -4.73722000 1.43515800

C 0.07289500 -1.82575800 0.13334900

C 0.36851800 -1.84266100 -1.15159000

H -0.03303500 -2.50122400 0.98050300

C 0.77391300 -2.82723900 -2.19633600

H 0.54505400 -3.86535300 -1.92153300

H 0.30219300 -2.61510200 -3.16251000

H 1.85880100 -2.75618500 -2.34881900

**^1^Int-1ʺ**

Fe -0.12359300 0.83574000 0.11672700

N -1.20334400 -0.63333900 -0.65411500

N 1.35000600 -0.20360400 -0.68025800

C -3.41652500 0.32802700 -0.25144900

C -4.26249300 0.15521300 0.85349900

C -5.12845900 1.19124100 1.20295500

H -5.77166400 1.06670700 2.07143000

C -5.18168900 2.37976900 0.48130500

C -4.32473100 2.52804700 -0.60861800

H -4.33936700 3.45866400 -1.17303600

C -3.43353300 1.52798700 -0.98125000

C -2.53278300 -0.78565600 -0.68782000

C -3.11360000 -1.97373700 -1.18007300

H -4.19560500 -2.04351300 -1.19896500

C -2.33384900 -3.01082300 -1.63896300

H -2.78619200 -3.92473600 -2.01368800

C -0.93642900 -2.86245400 -1.63360000

C -0.00623500 -3.86160800 -2.08458400

H -0.40237400 -4.80466200 -2.45043500

C 1.33434900 -3.64733400 -2.04915500

H 2.02430100 -4.41629000 -2.38400600

C 1.87062100 -2.40013900 -1.57889600

C 3.23740200 -2.06414700 -1.54939800

H 3.98147700 -2.78617500 -1.87232100

C 3.61277700 -0.80003200 -1.13974800

H 4.65492400 -0.49779600 -1.14819700

C 2.64267300 0.12709200 -0.71981300

C 0.97287300 -1.42235800 -1.13312800

C -0.43414300 -1.64954900 -1.14336200

C 2.93491800 1.54635400 -0.37332000

C 2.45927800 2.54529500 -1.25167000

C 2.68184600 3.88139700 -0.93591300

H 2.32537900 4.65074500 -1.61775400

C 3.35864900 4.25915400 0.22527300

C 3.82558800 3.25805300 1.06877400

H 4.35556000 3.53235700 1.97854600

C 3.62342400 1.90325300 0.79463600

C 1.75606900 2.19341300 -2.54387200

H 1.62045300 3.08414700 -3.16293100

H 2.32757500 1.45889500 -3.12220200

H 0.76707200 1.75239900 -2.37190300

C 4.09898900 0.88334000 1.79795500

H 4.28507600 -0.09469300 1.34855600

H 5.01829900 1.21866600 2.28760600

H 3.33646500 0.74800800 2.57452800

C -4.21811400 -1.09602700 1.69850800

H -4.77371300 -0.94922900 2.62876400

H -4.65423400 -1.96152400 1.18622200

H -3.18760400 -1.34985600 1.96681800

C -2.46935500 1.78554900 -2.11148700

H -2.85286900 2.55453100 -2.78877900

H -1.52027400 2.15936200 -1.69874000

H -2.25094700 0.88913100 -2.69913900

C -6.13506900 3.48451600 0.86077400

H -5.61239800 4.44273500 0.95329400

H -6.91548500 3.60992300 0.10060900

H -6.62815700 3.27697600 1.81506200

C 3.55731300 5.71624300 0.55615100

H 3.82926400 6.29317700 -0.33404500

H 2.63510700 6.15041500 0.96003100

H 4.34332500 5.85287200 1.30476200

C 0.67649600 -1.90920600 2.07156200

C 2.04189900 -2.19785200 2.20946000

C -0.15685100 -2.97403800 1.69481400

C 2.55623500 -3.46933700 1.96690000

H 2.72294500 -1.41084100 2.52023100

C 0.34324400 -4.25085400 1.45066800

H -1.22576100 -2.79979800 1.57280400

C 1.70678900 -4.50264900 1.57948900

H 3.62109400 -3.65504400 2.08528600

H -0.33213500 -5.04900000 1.15212200

H 2.10275000 -5.49616600 1.38654900

Si 0.00025400 -0.12278300 2.14703200

H -1.29822100 -0.28274100 2.88424400

H 0.91290000 0.56571800 3.13382200

H -1.17918200 1.70810400 0.87247600

**^3^Int-1**

Fe -0.03565600 -0.33997900 -0.45172400

N -1.22699200 1.17972600 0.28085200

N 1.45618100 1.06475300 0.15329600

C -3.30962700 -0.07030200 0.32930100

C -3.98340100 -0.45549200 -0.84128800

C -4.70954100 -1.64639700 -0.84136100

H -5.23680100 -1.94376200 -1.74617800

C -4.77501100 -2.46446700 0.28724400

C -4.08205900 -2.07098600 1.42962900

H -4.11654400 -2.70045400 2.31686400

C -3.34420600 -0.88718700 1.46979300

C -2.58069800 1.23102400 0.37744400

C -3.26904500 2.41624200 0.53319800

H -4.35021000 2.38827200 0.62093400

C -2.56933300 3.64074100 0.58051700

H -3.10040700 4.58123100 0.69362900

C -1.18410900 3.62640500 0.49198200

C -0.36894500 4.81714700 0.53490800

H -0.87203100 5.77615300 0.62818800

C 0.98279300 4.75680100 0.46867700

H 1.57849900 5.66535700 0.50768800

C 1.66419000 3.49709000 0.35257000

C 3.04634900 3.36868600 0.30034900

H 3.67309700 4.25626200 0.33932000

C 3.61858100 2.10043700 0.21686600

H 4.69408300 1.96261500 0.20529200

C 2.78941000 0.97592700 0.15263600

C 0.87740800 2.30812800 0.29103600

C -0.52674400 2.37033900 0.35801300

C 3.38834600 -0.39171100 0.14616400

C 3.61746100 -1.01453500 1.38667600

C 4.15189000 -2.29802100 1.40743100

H 4.32338500 -2.78548100 2.36555300

C 4.47389400 -2.97577700 0.22883400

C 4.26053400 -2.32871600 -0.98282000

H 4.51815100 -2.83600400 -1.91054900

C 3.72522700 -1.03863700 -1.04677900

C 3.27319400 -0.30622200 2.67485600

H 3.48164100 -0.94172200 3.54053700

H 3.84802900 0.61988800 2.78535400

H 2.21478700 -0.02133200 2.70149800

C 3.51169000 -0.38181700 -2.38785700

H 3.67576600 0.69889600 -2.33523300

H 4.19389000 -0.79457000 -3.13731300

H 2.48676300 -0.54313700 -2.73997800

C -3.91992300 0.39881900 -2.08400200

H -4.39857300 -0.10430500 -2.92958500

H -4.41671200 1.36329000 -1.93507800

H -2.87926800 0.60838000 -2.35429900

C -2.59425600 -0.49593700 2.71718400

H -2.85644000 -1.14890000 3.55484800

H -1.51415300 -0.56633100 2.54814500

H -2.81067900 0.53940600 3.00093800

C -5.54158400 -3.76275900 0.24883800

H -4.98328000 -4.53072600 -0.30108700

H -5.72747000 -4.14784700 1.25606500

H -6.50784600 -3.64120000 -0.25204300

C 5.05544200 -4.36616300 0.28635300

H 4.41687000 -5.03618300 0.87277300

H 5.16710600 -4.79631500 -0.71337900

H 6.04347500 -4.36059900 0.76127400

C 0.41676100 -1.79934400 0.90161400

H 1.13971300 -1.85918100 1.69310400

C -0.46971800 -2.25768900 0.14915200

C -1.38937300 -3.25848800 -0.42365500

H -0.89141200 -3.78212000 -1.24793600

H -1.67626200 -3.99746000 0.33146800

H -2.29585300 -2.79015400 -0.81413100

C 0.36288600 1.63721900 -2.92463800

H -0.32554800 2.39513800 -2.53564400

H 0.30157800 1.63077100 -4.01838700

H 1.37269600 1.93877600 -2.62664100

C 0.03232700 0.31694200 -2.35350600

C -0.33238100 -0.88956800 -2.35803600

H -0.63786600 -1.77893900 -2.88415100

**^3^Int-1ʹ**

Fe -0.03017700 -0.38846300 -0.03481700

N 1.23926100 1.18627700 -0.00146700

N -1.45717000 1.10845500 0.00346100

C 3.33719400 -0.07169700 0.08748500

C 3.52037900 -0.72485700 1.31650100

C 4.30434400 -1.87788400 1.35589600

H 4.45220300 -2.38213300 2.30895800

C 4.90603900 -2.39563900 0.20906100

C 4.68676300 -1.74440200 -1.00343600

H 5.12853800 -2.14858600 -1.91220100

C 3.90780500 -0.59069700 -1.08423900

C 2.59681000 1.22407100 0.03914000

C 3.30452800 2.40973900 0.05016100

H 4.38804600 2.37171500 0.08370100

C 2.62310200 3.64181500 0.02176500

H 3.17154800 4.57932100 0.02588200

C 1.23797700 3.64532200 -0.00224400

C 0.44777900 4.85279400 -0.02071700

H 0.97118300 5.80526700 -0.02177200

C -0.90399000 4.81204300 -0.03696000

H -1.48475800 5.73067500 -0.05252100

C -1.61189300 3.55801600 -0.03183100

C -2.99346500 3.46003200 -0.04721900

H -3.60049600 4.36140300 -0.06385900

C -3.59590800 2.19566300 -0.04218500

H -4.67455700 2.08547400 -0.05609000

C -2.80456200 1.05471700 -0.01683200

C -0.84856100 2.35242000 -0.01047000

C 0.55441500 2.39526900 -0.00487400

C -3.44662200 -0.29146600 -0.00876300

C -3.66600700 -0.96417700 -1.21978100

C -4.26933600 -2.21943500 -1.18995400

H -4.44064200 -2.74481800 -2.12749800

C -4.65098400 -2.82119600 0.00967900

C -4.41416400 -2.13419700 1.19919800

H -4.69929000 -2.59160300 2.14441800

C -3.81474500 -0.87415900 1.21019700

C -3.21711000 -0.35938300 -2.52574300

H -3.54099100 -0.96861700 -3.37432100

H -3.61299000 0.65343400 -2.65686100

H -2.12362400 -0.28496200 -2.55803000

C -3.53017700 -0.17110800 2.51370400

H -4.01539900 0.80994100 2.55585600

H -3.87857800 -0.76269100 3.36506600

H -2.45373000 -0.00176700 2.63237200

C 2.87959000 -0.19361500 2.57447400

H 3.24238100 -0.72771100 3.45718400

H 3.08634600 0.87373300 2.70600300

H 1.78970400 -0.30520700 2.53385700

C 3.67284500 0.08047900 -2.41517900

H 4.06471300 -0.52770300 -3.23567600

H 2.60302100 0.24231200 -2.58692900

H 4.15468600 1.06322800 -2.45910500

C 5.77740400 -3.62466900 0.28019300

H 5.78983200 -4.15947400 -0.67463900

H 6.81374400 -3.35810800 0.52121800

H 5.42913600 -4.31749900 1.05292000

C -5.27897000 -4.19286100 0.01398500

H -6.03844300 -4.28444500 -0.76968300

H -4.52639400 -4.96947100 -0.16776700

H -5.75543500 -4.41051400 0.97459400

C -0.82917100 -2.14597100 0.08728300

C 0.37278200 -2.27007300 -0.32848700

H -1.77068600 -2.62415700 0.31621200

C 1.49025800 -3.15451000 -0.73820200

H 1.15110800 -4.18858400 -0.86743600

H 1.94427900 -2.81013400 -1.67180900

H 2.27941200 -3.13370300 0.01957000

**^3^Int-1ʺ**

Fe -0.38843800 0.03503300 0.35309700

N -0.03324500 -1.96518000 0.16862600

N -2.28032800 -0.59977800 -0.07524400

C 2.29490500 -1.80400700 0.73424300

C 3.26416100 -1.45048800 -0.21517600

C 4.33274800 -0.64808900 0.18094400

H 5.06968700 -0.34915600 -0.56006500

C 4.45389800 -0.18276900 1.48701600

C 3.47021800 -0.53499100 2.41121900

H 3.54977900 -0.17562000 3.43577000

C 2.38932000 -1.33966700 2.05772300

C 1.12740800 -2.63653700 0.32805900

C 1.22172300 -4.00508700 0.13216300

H 2.17906500 -4.49356600 0.27773300

C 0.08482600 -4.73919100 -0.24834900

H 0.15014800 -5.81206400 -0.40661700

C -1.12670900 -4.08155300 -0.41284400

C -2.36472200 -4.72343500 -0.78660100

H -2.35274700 -5.79548000 -0.96570900

C -3.51827400 -4.02270600 -0.90938100

H -4.43980100 -4.52770700 -1.18706700

C -3.56562300 -2.59876000 -0.67262000

C -4.71327500 -1.82145800 -0.75644400

H -5.66306700 -2.27797100 -1.02035400

C -4.63531500 -0.44297000 -0.48361700

H -5.51862900 0.18517800 -0.52571500

C -3.41826000 0.12553000 -0.14951200

C -2.35100100 -1.95472500 -0.31715300

C -1.15778800 -2.67965700 -0.18764500

C -3.25073800 1.57119800 0.17295500

C -3.10659900 1.96311400 1.51358300

C -2.87294700 3.30789900 1.80009400

H -2.76424200 3.61514900 2.83838800

C -2.77418600 4.26418800 0.79145700

C -2.92553000 3.84988200 -0.53160900

H -2.84419300 4.58408700 -1.33062000

C -3.15878900 2.51627600 -0.86005800

C -3.22137900 0.95315800 2.63107800

H -3.12718900 1.43580500 3.60777300

H -4.18522100 0.43363400 2.59479800

H -2.44859400 0.17894300 2.55654100

C -3.28139700 2.09344300 -2.30322800

H -4.29842800 1.76077900 -2.53869500

H -3.02828600 2.91884000 -2.97450200

H -2.61000900 1.25709600 -2.52687700

C 3.14488100 -1.90247400 -1.64981000

H 3.90210400 -1.41618700 -2.27050000

H 3.26687900 -2.98714800 -1.74394700

H 2.15984400 -1.65278500 -2.06000800

C 1.35200200 -1.71912000 3.08854800

H 1.63816000 -1.36290600 4.08210800

H 0.36733900 -1.29872300 2.84964500

H 1.21998100 -2.80558600 3.13638600

C 5.62447800 0.67251300 1.90059400

H 5.29726900 1.52820000 2.50160600

H 6.33528400 0.09846300 2.50717300

H 6.15798300 1.05850500 1.02748400

C -2.48198800 5.70851300 1.11307300

H -2.67911700 5.93155900 2.16605600

H -1.43011000 5.94596500 0.91387200

H -3.09102900 6.38295800 0.50215800

C 2.35109800 1.78516000 -1.40447100

C 2.98219700 2.48353500 -0.36681400

C 3.11601800 1.45444000 -2.53043600

C 4.31993000 2.85529900 -0.45585100

H 2.42423400 2.73489000 0.53289700

C 4.45998100 1.81056200 -2.62167000

H 2.65659800 0.90512600 -3.34914900

C 5.06373900 2.51636800 -1.58419700

H 4.78713300 3.39922800 0.36040700

H 5.03371100 1.54129200 -3.50440500

H 6.11026200 2.80033200 -1.65438400

Si 0.51873100 1.35565900 -1.34095500

H -0.27097800 2.58619500 -1.62740300

H 0.29383300 0.43359600 -2.50684400

H 0.41043200 1.44335600 0.42966900

**^5^Int-1**

Fe -0.05073200 -0.41941600 0.22832200

N 1.23509900 1.23126400 -0.13475100

N -1.48178400 1.09756500 -0.15922100

C 3.30664800 -0.02300700 -0.15218200

C 3.81488400 -0.54408800 1.04609500

C 4.52707900 -1.74534900 1.01092000

H 4.92911500 -2.14704200 1.93931700

C 4.74423600 -2.43489700 -0.18013800

C 4.22742300 -1.89730400 -1.35843800

H 4.39001100 -2.41998500 -2.29912300

C 3.50852700 -0.70378200 -1.36527100

C 2.57824000 1.28234700 -0.17718900

C 3.27944200 2.47300200 -0.28502900

H 4.36303100 2.45050200 -0.31920700

C 2.56656700 3.68903100 -0.35579600

H 3.09637400 4.63487900 -0.43148400

C 1.18474800 3.67260800 -0.34562600

C 0.36918900 4.86238500 -0.43099100

H 0.87297200 5.82301800 -0.50359400

C -0.98131700 4.79647500 -0.42559800

H -1.57669100 5.70388500 -0.49124900

C -1.67333700 3.53367500 -0.33696300

C -3.05081000 3.41407500 -0.33221400

H -3.67041200 4.30494600 -0.39677400

C -3.64037300 2.14044200 -0.25063200

H -4.71680600 2.01159400 -0.25719200

C -2.82068000 1.02067300 -0.16691700

C -0.88222100 2.34228300 -0.25008200

C 0.51617900 2.41082500 -0.24653500

C -3.39509300 -0.35684200 -0.12046700

C -3.60288200 -1.03998000 -1.33230300

C -4.06307900 -2.35158700 -1.29742300

H -4.21540700 -2.88446100 -2.23442400

C -4.33805400 -2.99876400 -0.09052500

C -4.16157500 -2.28998500 1.09250700

H -4.39215700 -2.77060300 2.04153300

C -3.69786100 -0.97091000 1.10041000

C -3.36414200 -0.34954700 -2.65319600

H -3.33743700 -1.07243700 -3.47467700

H -4.16260500 0.37098100 -2.86547500

H -2.42660400 0.21512900 -2.65116100

C -3.53603400 -0.24838400 2.41552700

H -3.65858700 0.83201500 2.29852900

H -4.27492600 -0.59987400 3.14260800

H -2.54162400 -0.42663800 2.83813200

C 3.59429200 0.16171300 2.36093900

H 4.29643900 -0.19488100 3.12046800

H 3.71443700 1.24437800 2.25934600

H 2.57835400 -0.02397600 2.72734000

C 2.96531200 -0.14602600 -2.65625300

H 3.22442100 -0.78968900 -3.50189200

H 1.87482300 -0.05807700 -2.60968900

H 3.36205300 0.85658700 -2.84961900

C 5.48167600 -3.75040500 -0.19010300

H 4.78675800 -4.58704400 -0.04378900

H 5.99420600 -3.91336000 -1.14337500

H 6.22683800 -3.79858600 0.61011800

C -4.83399600 -4.42329200 -0.08607400

H -4.15135400 -5.08027100 -0.63665800

H -4.92849400 -4.81236800 0.93221400

H -5.81645600 -4.50124200 -0.56624500

C -0.29012700 -1.48449400 -1.46406700

H -0.83411200 -1.35686600 -2.38234300

C 0.45013900 -2.14285600 -0.69145200

C 1.26531800 -3.28684900 -0.23639900

H 0.65201500 -3.94418100 0.39061800

H 1.63571300 -3.86694500 -1.08793100

H 2.11984300 -2.94988500 0.35595100

C -0.06570600 1.28212400 2.92693100

H -0.02753300 1.15344700 4.01398600

H -0.98215300 1.81941100 2.66162800

H 0.77742500 1.90437400 2.60988700

C -0.02677500 -0.01297500 2.22752500

C 0.01492700 -1.25562000 2.05067500

H 0.03976700 -2.27200600 2.40363300

**^5^Int-1ʹ**

Fe -0.02710700 -0.38883400 -0.00183100

N 1.26167400 1.20023400 0.02802500

N -1.45851800 1.11954600 -0.01897300

C 3.34070000 -0.06805900 0.10987700

C 3.49364700 -0.73138800 1.33723600

C 4.24701700 -1.90488900 1.37868600

H 4.37261100 -2.41658000 2.33093400

C 4.84757600 -2.43215700 0.23610400

C 4.65934800 -1.76858200 -0.97540000

H 5.10089600 -2.17945400 -1.88127300

C 3.91133900 -0.59527900 -1.05884300

C 2.61417100 1.23537800 0.05324300

C 3.32570900 2.41990300 0.04303400

H 4.40909200 2.38539200 0.07275100

C 2.63534300 3.64916000 0.00514200

H 3.18100000 4.58860800 -0.00042300

C 1.25136700 3.65355400 -0.01883500

C 0.46096800 4.86150200 -0.04914700

H 0.98425000 5.81406000 -0.05910300

C -0.89049100 4.82078900 -0.06334300

H -1.47030900 5.73999900 -0.08509000

C -1.60106200 3.56735200 -0.05174000

C -2.98196300 3.47220200 -0.07188100

H -3.58489100 4.37630400 -0.09629500

C -3.59353300 2.20963600 -0.06280700

H -4.67226000 2.10387900 -0.08339600

C -2.80266400 1.06784000 -0.03569700

C -0.83777100 2.35952600 -0.02383900

C 0.56540000 2.40215200 -0.00638600

C -3.43890800 -0.28166200 -0.03171100

C -3.57720900 -0.99110400 -1.23400300

C -4.17287800 -2.25018200 -1.20559400

H -4.28405100 -2.80174600 -2.13725200

C -4.62326600 -2.82212200 -0.01572500

C -4.46460600 -2.10051000 1.16629800

H -4.80537700 -2.53369700 2.10452200

C -3.87702100 -0.83477300 1.17850100

C -3.04481900 -0.43025000 -2.52816800

H -3.45004400 -0.97106200 -3.38825200

H -3.28715700 0.63118000 -2.64092300

H -1.95174700 -0.51832300 -2.55829200

C -3.67728800 -0.09711100 2.47916500

H -4.18478500 0.87326100 2.47430700

H -4.05735300 -0.67852100 3.32394400

H -2.61272600 0.09967800 2.65075100

C 2.85424000 -0.19193300 2.59254900

H 3.23591800 -0.70518800 3.47968900

H 3.04161000 0.88091500 2.70544400

H 1.76639100 -0.32598200 2.56566100

C 3.70620100 0.08533400 -2.39014300

H 4.09696600 -0.52752500 -3.20766500

H 2.64189500 0.26796800 -2.57525500

H 4.20624600 1.05913100 -2.42444600

C 5.68775900 -3.68265100 0.30870300

H 5.66557100 -4.23205100 -0.63774400

H 6.73568900 -3.43966300 0.52289500

H 5.33852400 -4.35412800 1.09951900

C -5.24560500 -4.19635500 -0.01380400

H -6.00703000 -4.28782700 -0.79586700

H -4.49109200 -4.96962100 -0.20096400

H -5.71945400 -4.41874600 0.94695300

C -0.80588700 -2.14881500 0.22271600

C 0.34266000 -2.27635200 -0.31907600

H -1.72585700 -2.60928500 0.55290900

C 1.43089700 -3.14650700 -0.82586800

H 1.07350600 -4.16892200 -0.99262000

H 1.85177900 -2.75887600 -1.75794800

H 2.24864000 -3.17384400 -0.09869800

**^1^Int-2**

Fe 0.28461400 0.61316900 -0.34595800

N 1.17172600 -0.89470000 0.56351500

N -1.34080000 -0.22163100 0.53029600

C 3.46413100 -0.06056100 0.32463600

C 4.25356500 -0.17337700 -0.82778500

C 5.20833700 0.81097100 -1.08437200

H 5.81319200 0.73413400 -1.98565300

C 5.40393700 1.88699100 -0.21997000

C 4.60954000 1.96983600 0.92247900

H 4.74687600 2.80388900 1.60745700

C 3.64179500 1.01135200 1.21485500

C 2.48681300 -1.12308300 0.69332700

C 2.96356000 -2.31660400 1.26418800

H 4.03708700 -2.45557800 1.33508800

C 2.09183100 -3.27112000 1.74191400

H 2.45986300 -4.19471700 2.17955000

C 0.71329900 -3.01639300 1.68355700

C -0.29461100 -3.91178000 2.17806700

H 0.02084200 -4.85562100 2.61331700

C -1.61009100 -3.59330700 2.09676300

H -2.36790600 -4.28099900 2.46061700

C -2.03755000 -2.33953700 1.54193500

C -3.37609700 -1.92552800 1.47240300

H -4.17101300 -2.58275100 1.81234400

C -3.64886200 -0.65958900 1.00421200

H -4.66331500 -0.27475600 0.98952200

C -2.61424200 0.19316600 0.57534800

C -1.06899200 -1.45256400 1.04491200

C 0.31084800 -1.80651600 1.09422000

C -2.93136500 1.62976600 0.32515500

C -2.76001600 2.51498200 1.40554400

C -3.07966000 3.85740500 1.23243200

H -2.94013200 4.54582900 2.06405900

C -3.57041500 4.34384200 0.01923000

C -3.75955400 3.44185600 -1.02212000

H -4.15629100 3.79891300 -1.97018400

C -3.45624300 2.08533200 -0.88759400

C -2.21118500 2.02409500 2.72225800

H -2.15854600 2.83652700 3.45268600

H -2.83349700 1.22740800 3.14640000

H -1.20372400 1.61395300 2.58638400

C -3.68616600 1.14536900 -2.04119200

H -4.25425600 0.26098100 -1.73189000

H -4.23919800 1.63820800 -2.84593700

H -2.73383000 0.79767000 -2.45424200

C 4.07327400 -1.32634400 -1.78265500

H 4.73616800 -1.22559200 -2.64654700

H 4.29411300 -2.28688900 -1.30231600

H 3.04385000 -1.37622900 -2.15184900

C 2.80872600 1.12825000 2.46587000

H 3.15685200 1.95549700 3.09078700

H 1.75938700 1.30920800 2.20840100

H 2.85190000 0.21000600 3.06319800

C 6.46346700 2.92239000 -0.50162800

H 6.20370700 3.88878700 -0.05871600

H 7.42998800 2.61701700 -0.08236700

H 6.60352800 3.06781800 -1.57737700

C -3.87901000 5.81028500 -0.14835200

H -4.52588800 6.17555300 0.65702000

H -2.95986800 6.40748800 -0.12579600

H -4.38049200 6.00652000 -1.10065400

C 0.21587900 2.40008300 0.30917500

C 1.08520200 2.29819100 -0.64052900

H -0.23802200 3.14916100 0.94378100

C 2.05562400 2.98378100 -1.52834600

H 2.13775700 4.05331700 -1.30362800

H 3.04374800 2.52466700 -1.42787100

H 1.75194700 2.86719900 -2.57465700

H 1.28422200 0.49050800 -1.45448900

Si -0.03990700 -0.65000800 -2.26883000

H -0.81995400 0.12505200 -3.29023400

H 1.12408900 -1.16781700 -3.06732000

C -1.00305300 -2.26586800 -1.98696100

C -0.31862700 -3.41389100 -1.55858300

C -2.39698800 -2.35760300 -2.09856900

C -0.99097200 -4.58951300 -1.23738200

H 0.76651700 -3.38633800 -1.46534100

C -3.07966300 -3.53008500 -1.78337400

H -2.96390300 -1.49900100 -2.44536500

C -2.37849200 -4.64989700 -1.34422100

H -0.43270300 -5.45892500 -0.89989300

H -4.16137500 -3.57077500 -1.88445700

H -2.90730700 -5.56600400 -1.09505900

**^3^Int-2**

Fe -0.37161900 -0.37215400 -0.83283400

N 1.07226300 1.10506600 -0.99046800

N -1.51608000 1.33561000 -0.35251900

C 2.98613900 -0.36898900 -1.17081700

C 3.76700600 -0.71741300 -0.05376300

C 4.19557300 -2.03757300 0.08147300

H 4.77955600 -2.31266100 0.95798000

C 3.88507500 -3.01288700 -0.86410700

C 3.16287600 -2.62937500 -1.99255400

H 2.94177900 -3.36776200 -2.76123900

C 2.72703500 -1.31704800 -2.17412900

C 2.40653900 1.00309700 -1.25466100

C 3.19617800 2.10041100 -1.53153300

H 4.24857900 1.94679100 -1.74485700

C 2.64722000 3.39589800 -1.50787600

H 3.26290100 4.26519000 -1.71988700

C 1.30733600 3.54868200 -1.18257500

C 0.65594500 4.83271600 -1.07401700

H 1.24742500 5.72178200 -1.27756100

C -0.64644000 4.94483900 -0.72097400

H -1.11427300 5.92213900 -0.63464400

C -1.44324900 3.77733000 -0.44726000

C -2.77600700 3.81785200 -0.06026600

H -3.27773200 4.77540800 0.05208400

C -3.46329100 2.62296700 0.18466600

H -4.50427300 2.62403800 0.48923900

C -2.80362200 1.40955700 0.02394400

C -0.82258800 2.50141600 -0.57605700

C 0.53476900 2.38147800 -0.92730700

C -3.49031400 0.10156700 0.24344400

C -4.11556200 -0.53835100 -0.83737600

C -4.68719500 -1.79266300 -0.63685800

H -5.16686800 -2.29395900 -1.47539600

C -4.64874800 -2.42498400 0.60567600

C -4.03649900 -1.76167600 1.66743700

H -4.00581200 -2.23608900 2.64620900

C -3.45894300 -0.50117200 1.50821200

C -4.15793300 0.11740700 -2.19376600

H -4.65820100 -0.52403400 -2.92478100

H -4.68869400 1.07516500 -2.15557800

H -3.14427300 0.32204700 -2.55412300

C -2.81627500 0.19707800 2.68088900

H -3.33110900 1.13593500 2.91346400

H -2.83684400 -0.43347800 3.57443700

H -1.77187200 0.45346300 2.47479100

C 4.18320100 0.30473200 0.97556500

H 5.02633400 0.90093600 0.60623400

H 3.37763300 1.00082900 1.21780300

H 4.49774800 -0.18261900 1.90220900

C 2.09145000 -0.92003700 -3.48406600

H 1.56035900 -1.76425500 -3.93302300

H 1.38760500 -0.09405200 -3.37002000

H 2.86961900 -0.60021800 -4.18848400

C 4.35807500 -4.43516200 -0.69532700

H 3.63063800 -5.14793000 -1.09725300

H 5.30542300 -4.59948200 -1.22315900

H 4.52373800 -4.67658500 0.35932100

C -5.22542800 -3.80754400 0.78066200

H -6.16880800 -3.91938500 0.23628100

H -4.53394600 -4.56839900 0.39725200

H -5.41261000 -4.03242400 1.83507600

C -0.93864200 -1.28042900 -2.46580200

C -1.07160600 -2.11018900 -1.51578100

H -1.04419300 -1.05440400 -3.51555800

C -1.45405000 -3.43080400 -0.96486700

H -2.03124600 -4.01556900 -1.69000600

H -0.55662700 -4.00198300 -0.69792200

H -2.05575500 -3.31106000 -0.05810500

H -0.74357400 -0.89274500 0.79033700

Si 0.42216300 -1.66122100 1.41341200

H -0.32026500 -2.70084100 2.17907400

H 1.36303100 -2.36789500 0.51630700

C 1.24488800 -0.45127200 2.57755300

C 2.12913200 -0.88644000 3.57363800

C 0.99647300 0.92343900 2.46431800

C 2.75179800 0.02192500 4.42454400

H 2.34023200 -1.94774800 3.68876400

C 1.61527700 1.83578900 3.31513000

H 0.32557600 1.29090300 1.69258600

C 2.49755500 1.38569500 4.29322600

H 3.43785300 -0.33233800 5.18868300

H 1.41450400 2.89716600 3.20366400

H 2.98608900 2.09530500 4.95480000

**^5^Int-2**

Fe -0.40598400 -0.01872700 -0.71099700

N 0.67939800 1.72618700 -0.45518100

N -1.93158700 1.18215700 -0.06245600

C 2.80706600 0.90946100 -1.29036300

C 3.78471000 0.28187900 -0.50420200

C 4.50421700 -0.78326400 -1.04960300

H 5.24496900 -1.28474400 -0.42981300

C 4.29711700 -1.21788200 -2.35525700

C 3.36285200 -0.53713600 -3.13635800

H 3.21549500 -0.83682400 -4.17251000

C 2.61400200 0.52091900 -2.62838300

C 1.97560000 2.00939500 -0.71983600

C 2.52587800 3.25957500 -0.48560100

H 3.57307100 3.42562400 -0.71163400

C 1.72631600 4.28654000 0.04317100

H 2.14869800 5.26866100 0.23756900

C 0.38799100 4.04416500 0.30215800

C -0.51702800 5.03802900 0.82524100

H -0.12220700 6.02864600 1.03556600

C -1.82125300 4.75665500 1.04691700

H -2.49158000 5.51671400 1.43982200

C -2.36546800 3.45020100 0.76529300

C -3.69456200 3.11306200 0.95927000

H -4.39300500 3.85262900 1.34115100

C -4.13034000 1.80911000 0.65759300

H -5.16484800 1.51844500 0.80266500

C -3.23047400 0.88070400 0.16347900

C -1.48420900 2.45505900 0.24852900

C -0.12755600 2.74195700 0.03134500

C -3.66987800 -0.51269700 -0.14249100

C -3.82170500 -0.93292200 -1.47118800

C -4.24410500 -2.23971300 -1.72171400

H -4.35879800 -2.56535000 -2.75372100

C -4.52349100 -3.13189700 -0.69142000

C -4.36092000 -2.69233500 0.62373200

H -4.55937700 -3.38180200 1.44237400

C -3.93306200 -1.39964800 0.91656900

C -3.51041300 -0.01808300 -2.62912800

H -4.06913400 -0.31551400 -3.52174400

H -3.74911600 1.02492800 -2.40241900

H -2.44212900 -0.06465500 -2.87867300

C -3.73198600 -0.98011300 2.35297100

H -2.75027200 -0.51584600 2.49474500

H -4.48001500 -0.24535000 2.66926400

H -3.80106600 -1.84226200 3.02288200

C 4.10412400 0.74937700 0.89542200

H 4.55768200 -0.05558200 1.48065300

H 4.81005000 1.58856200 0.86902800

H 3.21498800 1.09561400 1.42767200

C 1.62997000 1.24136100 -3.51618200

H 1.81455000 1.01076600 -4.56967800

H 0.60054600 0.94969900 -3.27865100

H 1.69672800 2.32633100 -3.38498600

C 5.05281700 -2.39911600 -2.90998000

H 4.48648100 -3.32798000 -2.76816500

H 5.23410700 -2.28789100 -3.98388000

H 6.02009900 -2.52479800 -2.41327500

C -4.99249300 -4.53635700 -0.97782300

H -4.83431500 -4.80246800 -2.02719300

H -4.45866200 -5.26578900 -0.35913600

H -6.06220200 -4.64710300 -0.76364800

C 0.01911500 -1.47621100 -1.91530200

C -0.40254600 -1.97307800 -0.81091000

H 0.41858400 -1.70470000 -2.89529100

C -0.73662900 -3.16470000 0.00662800

H -0.41482500 -4.09202400 -0.48168300

H -0.26127300 -3.10693800 0.99188900

H -1.81873500 -3.21017800 0.16611500

H -0.43274600 -0.19319600 1.83615600

Si 0.58351700 0.09236400 2.88343700

H 1.12095000 1.45788400 2.65351900

H -0.07006200 0.06401900 4.21912300

C 1.91896400 -1.22010300 2.80466600

C 2.64734200 -1.57880900 3.94801200

C 2.24111300 -1.84852000 1.59414100

C 3.66524100 -2.52609900 3.88342000

H 2.41955700 -1.11442300 4.90510700

C 3.25335200 -2.80159700 1.52541400

H 1.71665500 -1.58565900 0.67886700

C 3.96870800 -3.14085300 2.67019100

H 4.21855700 -2.78843600 4.78068800

H 3.48497700 -3.26700100 0.57187100

H 4.75900800 -3.88449700 2.62011800

**^1^Ts-1**

Fe 0.25503800 0.63894200 -0.27861100

N 1.19923500 -0.85208400 0.58886700

N -1.33399200 -0.26890100 0.55392600

C 3.46178300 0.05414500 0.34537400

C 4.25442100 -0.04359900 -0.80554600

C 5.16269600 0.97941200 -1.08262700

H 5.77056800 0.91285800 -1.98257200

C 5.31043000 2.07744800 -0.23817600

C 4.52381400 2.13809400 0.91242900

H 4.63343300 2.98376200 1.58831900

C 3.59810200 1.14499500 1.22137000

C 2.52259500 -1.03950200 0.71999600

C 3.03800600 -2.21931900 1.28269500

H 4.11549100 -2.32311400 1.35351400

C 2.19885200 -3.20814100 1.75041300

H 2.59728600 -4.12264300 2.18028500

C 0.81319900 -3.00077300 1.68795700

C -0.16488900 -3.93837500 2.16423400

H 0.18246100 -4.87431700 2.59222200

C -1.49057400 -3.67041400 2.07099500

H -2.22561300 -4.39049000 2.41841500

C -1.95990700 -2.42773300 1.52480600

C -3.31252400 -2.06751600 1.43916500

H -4.08514800 -2.75989000 1.76006200

C -3.62973000 -0.80929200 0.97805700

H -4.65931100 -0.46811700 0.94797800

C -2.62507700 0.09098800 0.57645500

C -1.02032900 -1.49627100 1.05437800

C 0.37033000 -1.79928100 1.11016100

C -3.00409600 1.51144400 0.32081600

C -2.87447300 2.41178200 1.39415300

C -3.26300300 3.73527200 1.21244500

H -3.15494300 4.43611000 2.03837600

C -3.78289300 4.18698200 -0.00186400

C -3.92206700 3.27057900 -1.03890300

H -4.33356200 3.60159800 -1.99012900

C -3.55033500 1.93227100 -0.89586900

C -2.28763500 1.96229500 2.70861800

H -2.30778300 2.77116000 3.44459300

H -2.83475800 1.10963500 3.12700700

H -1.24718600 1.64693400 2.56738100

C -3.72430500 0.97742700 -2.04745900

H -4.22809400 0.05609400 -1.73552600

H -4.31570100 1.43146600 -2.84775600

H -2.75327200 0.69566100 -2.46754300

C 4.14484400 -1.23270100 -1.72697100

H 4.68334900 -1.05367600 -2.66177300

H 4.56668700 -2.13448400 -1.26665000

H 3.10187500 -1.44659400 -1.97793500

C 2.76842900 1.24227200 2.47655800

H 3.10424100 2.07385100 3.10256500

H 1.71480900 1.40577100 2.22481800

H 2.83015400 0.32316400 3.07042000

C 6.26867700 3.19216400 -0.57396200

H 7.05355900 2.85330900 -1.25704100

H 5.74293800 4.02266900 -1.06109300

H 6.74747100 3.59182500 0.32580300

C -4.20576000 5.62494200 -0.16818800

H -5.13724300 5.82671600 0.37433000

H -3.44568400 6.31046800 0.22169500

H -4.37453300 5.87064200 -1.22087800

C 0.08946200 2.44906200 0.28759100

C 0.98974800 2.35433900 -0.64004500

H -0.41048500 3.23641900 0.83613000

C 1.92033400 3.11468800 -1.51554300

H 1.83992100 4.19250000 -1.34052800

H 2.95266900 2.80042600 -1.33130100

H 1.70248700 2.91092000 -2.56980800

H 1.37147300 0.81509900 -1.24830100

Si 0.02437500 -0.60277900 -2.22407900

H -0.79212700 0.11586800 -3.26313000

H 1.25656600 -0.99103100 -2.99392800

C -0.83609800 -2.28443300 -2.00295200

C -0.10158400 -3.40026800 -1.57249300

C -2.22138100 -2.44887200 -2.14188200

C -0.71704800 -4.61385900 -1.27861300

H 0.97828200 -3.31660600 -1.45470600

C -2.84777000 -3.65830700 -1.85151200

H -2.82575900 -1.61669900 -2.49072200

C -2.09719800 -4.74564900 -1.41191700

H -0.11974600 -5.45669500 -0.94016100

H -3.92390700 -3.75416000 -1.97270200

H -2.58209300 -5.69089500 -1.18318400

**^3^Ts-1**

Fe 0.08835000 0.77729400 -0.29431000

N 1.20322300 -0.91426200 0.67803500

N -1.39422300 -0.27390600 0.60817200

C 3.45731200 -0.09076900 0.35730900

C 4.16811600 -0.16253300 -0.84766700

C 5.04550500 0.87410700 -1.16931800

H 5.58917600 0.82965400 -2.11079800

C 5.23967900 1.96035500 -0.31866100

C 4.54078700 1.99069600 0.88762300

H 4.69049000 2.82552900 1.56922400

C 3.64918900 0.98212900 1.24299200

C 2.50863800 -1.17251400 0.74683200

C 2.99263500 -2.39992700 1.23522400

H 4.06452500 -2.56418200 1.26962200

C 2.10855100 -3.35804500 1.68125200

H 2.46625000 -4.30872600 2.06767900

C 0.73089800 -3.09087100 1.64783700

C -0.26596900 -4.02188300 2.09359900

H 0.05884900 -4.98332000 2.48135400

C -1.58291800 -3.71512700 2.01895000

H -2.33616000 -4.42662400 2.34449200

C -2.02153700 -2.44355700 1.51537700

C -3.37335600 -2.06889600 1.45187000

H -4.14515200 -2.76852000 1.75897900

C -3.69285500 -0.79697100 1.03260500

H -4.72094500 -0.45051000 1.01679000

C -2.68145200 0.09562000 0.64082000

C -1.06703800 -1.50995100 1.07393900

C 0.33034400 -1.84309800 1.12954100

C -2.99678800 1.52622100 0.36093800

C -2.78276700 2.44599500 1.40535400

C -3.04246100 3.79199600 1.17805500

H -2.87267700 4.50662900 1.98118600

C -3.51562600 4.24961200 -0.05351700

C -3.75734000 3.31555800 -1.05465000

H -4.14980600 3.65073500 -2.01256800

C -3.51188700 1.95283900 -0.86781300

C -2.28669700 1.97746900 2.75066200

H -2.18641400 2.81696100 3.44486900

H -2.97492500 1.25003300 3.19742600

H -1.31086400 1.49074500 2.65187200

C -3.79012700 0.98567500 -1.98998700

H -4.21341200 0.04594100 -1.62188700

H -4.49320000 1.41545100 -2.70966800

H -2.86908400 0.74163400 -2.53006400

C 4.00873700 -1.33909300 -1.77845500

H 4.49561900 -1.14704200 -2.73856900

H 4.45521600 -2.24648700 -1.35378700

H 2.95444200 -1.55091900 -1.97758500

C 2.91547000 1.03938900 2.55848300

H 3.24833200 1.89410600 3.15410300

H 1.83784900 1.13616200 2.38999600

H 3.08036200 0.12892900 3.14655200

C 6.14962800 3.09731700 -0.70894200

H 5.57779300 3.90309600 -1.18592500

H 6.65205800 3.52536700 0.16437700

H 6.91581800 2.77215400 -1.41948500

C -3.74505100 5.72148400 -0.28610800

H -4.37619900 5.89577000 -1.16273500

H -4.22482900 6.19216100 0.57863000

H -2.79302800 6.23907400 -0.45406200

C 0.38139900 2.41281500 0.72159500

C 1.05556800 2.48735100 -0.37188500

H 0.17181400 3.03456700 1.58281300

C 1.95088900 3.32193500 -1.21764800

H 2.22323100 4.24857600 -0.70129000

H 2.86696400 2.77489700 -1.46355800

H 1.45628500 3.57874300 -2.16155900

H 0.96899300 1.19457900 -1.45994000

Si -0.04848300 -0.47145400 -2.32430500

H -0.90399000 0.17462200 -3.37868900

H 1.21892400 -0.77705400 -3.06632500

C -0.83112600 -2.18958300 -2.08633700

C -0.05918000 -3.27758400 -1.65146300

C -2.21194200 -2.39959000 -2.21335000

C -0.63348300 -4.50988100 -1.35071000

H 1.01728000 -3.15775200 -1.53398400

C -2.79747200 -3.62638600 -1.91144600

H -2.84583100 -1.59055200 -2.56519000

C -2.00896900 -4.68775700 -1.47458700

H -0.00634800 -5.33046600 -1.01135100

H -3.87097400 -3.75640700 -2.02366200

H -2.46160600 -5.64697400 -1.23829000

**^3^Ts-1a**

Fe -0.03343300 -0.80662100 -0.38090600

N -1.39295300 0.64288500 0.67694800

N 1.28089800 0.45081000 0.55022200

C -3.48627400 -0.50004800 0.26767300

C -4.17702000 -0.43653900 -0.95086900

C -4.83103100 -1.57949000 -1.40708700

H -5.35015400 -1.54252600 -2.36281700

C -4.83438600 -2.76550900 -0.67349700

C -4.17361500 -2.78745300 0.55272700

H -4.17370100 -3.70184200 1.14242300

C -3.49948000 -1.67002400 1.04033800

C -2.72364500 0.67899500 0.76712400

C -3.39405000 1.76608300 1.35511000

H -4.47771400 1.74712000 1.40496100

C -2.67307300 2.82242700 1.86768400

H -3.17457100 3.67097600 2.32521600

C -1.27155000 2.79295800 1.80151600

C -0.43829800 3.85268300 2.29317200

H -0.91254000 4.71606400 2.75118300

C 0.90819400 3.78755700 2.16844000

H 1.53815600 4.59824900 2.52291300

C 1.54346500 2.64779200 1.56914500

C 2.93627300 2.53481500 1.43818100

H 3.58070000 3.34557900 1.76521500

C 3.46282600 1.37241500 0.92281300

H 4.53492000 1.22388200 0.84799000

C 2.61537600 0.32898300 0.51595500

C 0.75453700 1.58343500 1.09478000

C -0.67757400 1.66812000 1.19304700

C 3.19768800 -1.00036200 0.17065200

C 3.23537200 -1.95938000 1.19782200

C 3.79291000 -3.20706200 0.93441400

H 3.81960900 -3.95163100 1.72778200

C 4.31893600 -3.52325000 -0.31781800

C 4.29813700 -2.54483200 -1.30794900

H 4.72113000 -2.76756900 -2.28543800

C 3.75648200 -1.27850300 -1.08336100

C 2.69567400 -1.63362000 2.56764800

H 2.77980900 -2.49319200 3.23838200

H 3.23935500 -0.79512500 3.01911400

H 1.64105300 -1.34744100 2.50570900

C 3.78087000 -0.24988300 -2.18403700

H 4.07743500 0.73536300 -1.80948700

H 4.48339600 -0.53888000 -2.97089500

H 2.79262400 -0.14246200 -2.64265800

C -4.23207200 0.83997500 -1.75330900

H -4.66450400 0.66168700 -2.74173300

H -4.84742100 1.59719800 -1.25202100

H -3.23718800 1.26833900 -1.90082000

C -2.80387400 -1.71766700 2.37642300

H -2.89179100 -2.70936700 2.82906400

H -1.74043600 -1.48643300 2.26515400

H -3.23206100 -0.98654300 3.07305500

C -5.56517300 -3.98149500 -1.18570600

H -5.23848300 -4.89016700 -0.67107300

H -6.64650900 -3.88270400 -1.03156100

H -5.39988800 -4.12157800 -2.25906000

C 4.93778600 -4.87338700 -0.57914500

H 4.53627800 -5.63372000 0.09777700

H 4.75509800 -5.20219500 -1.60703200

H 6.02431200 -4.84046600 -0.43222900

C -0.14172400 -2.56144800 0.51279300

C -0.75639400 -2.58529700 -0.62062100

H -0.79743600 -1.19668800 -1.64089300

Si -0.15775300 0.56203600 -2.34063100

H 0.79259400 0.13735900 -3.42302000

H -1.46316500 0.68395500 -3.06646300

C 0.31940200 2.37212800 -2.00148400

C -0.61401800 3.28764400 -1.49262700

C 1.63886200 2.82356400 -2.14910500

C -0.25041100 4.58463200 -1.14049800

H -1.65075100 2.98169900 -1.35857200

C 2.01450600 4.11708500 -1.79597300

H 2.38973900 2.15328700 -2.55742100

C 1.06981400 5.00333500 -1.28557200

H -0.99782900 5.26795100 -0.74541500

H 3.04612800 4.43506400 -1.92474200

H 1.35711700 6.01426700 -1.00916200

C 0.14600800 -3.51626200 1.62218200

H -0.51139000 -4.39416200 1.58614400

H 1.18123600 -3.86746600 1.54536700

H 0.03899600 -3.04995000 2.60737100

H -1.33669300 -3.20683500 -1.28598900

**^5^Ts-1**

Fe 0.24807500 -0.70080500 -0.05126600

N 1.45894500 0.70657800 -0.78306200

N -1.22448100 0.65719400 -0.90133600

C 3.43134600 -0.59572300 -0.32420200

C 3.93468500 -0.77702300 0.97362300

C 4.38759700 -2.04228800 1.35015300

H 4.76531900 -2.18724000 2.36048200

C 4.36906500 -3.12139100 0.46954600

C 3.89403700 -2.91021800 -0.82426600

H 3.88770800 -3.73787300 -1.53136000

C 3.42660800 -1.66504700 -1.23810700

C 2.83026900 0.70390800 -0.73684900

C 3.56648900 1.81042300 -1.07523000

H 4.64921000 1.75841000 -1.02815200

C 2.91423900 2.99988000 -1.49708700

H 3.48792600 3.88292200 -1.76092900

C 1.51949500 3.01447500 -1.59051300

C 0.75408600 4.16292300 -1.99999500

H 1.29637100 5.06496700 -2.27195700

C -0.60625400 4.14667000 -2.03700900

H -1.15963400 5.03126700 -2.34139500

C -1.33392200 2.97038000 -1.68111200

C -2.72637400 2.86722900 -1.70504500

H -3.32183600 3.72573500 -2.00661500

C -3.34159800 1.67506000 -1.35696300

H -4.41959500 1.56266200 -1.38399100

C -2.54828800 0.58506700 -0.95476400

C -0.59610900 1.81892200 -1.28090900

C 0.80987900 1.84131900 -1.23307100

C -3.16848700 -0.72663000 -0.60209700

C -3.09745700 -1.78067600 -1.52633200

C -3.64737800 -3.01478400 -1.18426300

H -3.59002900 -3.83285100 -1.89931600

C -4.26614900 -3.22586100 0.04657000

C -4.33812100 -2.15917200 0.94077300

H -4.82345500 -2.30270000 1.90485600

C -3.80412300 -0.90696400 0.63447000

C -2.43320900 -1.58918300 -2.86637700

H -2.58420100 -2.46287400 -3.50680300

H -2.83148200 -0.70985700 -3.38436200

H -1.35582500 -1.42956500 -2.74819200

C -3.94813400 0.22500800 1.62049300

H -3.17852600 0.98725700 1.48376500

H -4.91999400 0.71995300 1.50207500

H -3.88611900 -0.14318100 2.64956600

C 4.01113600 0.36912800 1.95216500

H 4.75667400 1.10764900 1.63811000

H 3.05687600 0.89561600 2.03842500

H 4.28588500 0.01219700 2.94905200

C 2.94014800 -1.46460800 -2.65191600

H 1.87738800 -1.20246800 -2.67048500

H 3.47740800 -0.64140400 -3.13569400

H 3.08694400 -2.37019600 -3.24830000

C 4.88285500 -4.47429300 0.89474800

H 4.73956600 -4.63515100 1.96796700

H 4.37264600 -5.28166700 0.35949400

H 5.95588200 -4.57073400 0.68858100

C -4.83944100 -4.57152300 0.41462600

H -4.34941400 -4.97845100 1.30695800

H -5.90987900 -4.49774200 0.63717400

H -4.71423800 -5.29607200 -0.39548000

C 0.18237900 -2.51015500 -0.79638200

C -0.33330400 -2.66256200 0.36695700

H 0.42769600 -3.09391000 -1.66892100

C -0.90530400 -3.62816600 1.34866900

H -0.26601500 -3.69981700 2.23545400

H -1.89899400 -3.30043900 1.66920700

H -0.98932300 -4.61660100 0.88933500

H -0.44976200 -1.48433600 1.19154000

Si -0.02671300 0.04133800 2.29489300

H -1.15282000 -0.45343800 3.14774800

H 1.21693800 -0.33226700 3.03243100

C -0.23763500 1.91408300 2.27306300

C -1.39324800 2.49306900 2.81783800

C 0.71273700 2.77336400 1.70294100

C -1.61127300 3.86734000 2.76068900

H -2.13467000 1.86179200 3.30069500

C 0.50215300 4.14682200 1.64486100

H 1.62787200 2.37246300 1.27785700

C -0.66690900 4.69687400 2.16343700

H -2.51856600 4.28901700 3.18432400

H 1.24802700 4.78255100 1.17711800

H -0.83809500 5.76811600 2.10614300

**^1^Int-3**

Fe 0.20522000 0.62134500 -0.10229400

N 1.12677600 -0.91729500 0.65146400

N -1.37336500 -0.27752600 0.58253600

C 3.40642400 -0.10684100 0.34210900

C 4.08536600 -0.21902800 -0.87964300

C 4.95331200 0.80574500 -1.25784400

H 5.46757000 0.73271600 -2.21374600

C 5.18322600 1.91064300 -0.44125000

C 4.53645600 1.96838300 0.79341500

H 4.72463600 2.81200400 1.45444300

C 3.64903700 0.97627700 1.20298900

C 2.44580100 -1.16168500 0.76526800

C 2.91955600 -2.36830300 1.30085000

H 3.99328100 -2.51090400 1.36210200

C 2.04862200 -3.34196200 1.74827400

H 2.41832400 -4.27738400 2.15806700

C 0.67158300 -3.09332800 1.67717800

C -0.34454600 -4.01552400 2.10479500

H -0.03303100 -4.97379000 2.51046200

C -1.66115600 -3.71014900 1.99283900

H -2.42020300 -4.42156400 2.30448600

C -2.08822100 -2.43997800 1.47361000

C -3.42536100 -2.03189800 1.36743000

H -4.22817800 -2.70519400 1.65261900

C -3.69233800 -0.75029500 0.93177700

H -4.71000400 -0.37664500 0.88602000

C -2.65389600 0.12536400 0.57291900

C -1.10828400 -1.52592700 1.05738400

C 0.26949000 -1.86217400 1.13352600

C -2.94382900 1.56219000 0.30209600

C -2.74625100 2.46932300 1.36150400

C -2.99841200 3.81893700 1.14470700

H -2.83894500 4.52398700 1.95860400

C -3.45490700 4.29154200 -0.08758400

C -3.68349900 3.36930700 -1.10233400

H -4.06083100 3.71608000 -2.06217600

C -3.44562300 2.00361600 -0.92712300

C -2.27720000 1.98686500 2.71154700

H -2.21591800 2.81562500 3.42293200

H -2.96225600 1.23693600 3.12452500

H -1.28777000 1.52486200 2.63113500

C -3.72070500 1.05013300 -2.06131900

H -4.18090600 0.12143200 -1.70965800

H -4.39209500 1.50356900 -2.79648000

H -2.79247300 0.78111800 -2.57597400

C 3.92176000 -1.43441500 -1.75753000

H 4.37013100 -1.26909700 -2.74105200

H 4.40888900 -2.30945200 -1.31018400

H 2.86931300 -1.68313400 -1.91217500

C 2.97684600 1.06016200 2.54989600

H 3.32416100 1.93657400 3.10416600

H 1.89063200 1.13794100 2.43306800

H 3.18769500 0.16977900 3.15359600

C 6.09056900 3.02936600 -0.88757400

H 6.84388900 2.67515000 -1.59793800

H 5.51653600 3.82099100 -1.38555000

H 6.60856800 3.48620500 -0.03834800

C -3.71741100 5.76232700 -0.29216700

H -4.56896200 6.09908000 0.31095600

H -2.85022900 6.36251400 0.00487200

H -3.93951000 5.98598800 -1.33977900

C 0.37539800 2.32959000 0.71588700

C 1.15153600 2.49432800 -0.35280800

H 0.05435000 3.14130100 1.36660200

C 1.82240900 3.67127700 -1.00042400

H 1.52097300 4.59968600 -0.50732400

H 2.91181100 3.57212200 -0.92882900

H 1.56516700 3.73765800 -2.06344800

H 1.46576000 1.54421400 -0.93061700

Si 0.01593000 -0.48120100 -2.20758000

H -0.85158600 0.23540900 -3.20990100

H 1.26175800 -0.73200200 -3.01413400

C -0.79090200 -2.19778100 -2.06851800

C -0.05523600 -3.30987400 -1.62966700

C -2.17014000 -2.38172900 -2.24776700

C -0.66063200 -4.53775400 -1.37586000

H 1.01815300 -3.21394400 -1.46966600

C -2.78735900 -3.60349300 -1.99242400

H -2.77685500 -1.55286100 -2.60149500

C -2.03390800 -4.68821700 -1.55093000

H -0.06018800 -5.37642600 -1.03231500

H -3.85867900 -3.71135100 -2.14309100

H -2.51105800 -5.64373100 -1.35044100

**^3^Int-3ʹ**

Fe -0.11063200 0.75318700 0.22398200

N -1.21993200 -0.92781400 -0.71376100

N 1.37659600 -0.29253300 -0.61590700

C -3.46483300 -0.11010100 -0.36562400

C -4.12436600 -0.20020900 0.86920200

C -4.95404900 0.84952900 1.25986200

H -5.45129200 0.79545300 2.22610300

C -5.16066900 1.96312900 0.44667500

C -4.51743800 2.01016000 -0.78873100

H -4.67098600 2.87029800 -1.43708500

C -3.66773400 0.98987800 -1.21227700

C -2.52513400 -1.18482400 -0.79197500

C -3.00676500 -2.40846300 -1.29097800

H -4.07856300 -2.57059200 -1.33548400

C -2.12002200 -3.36871100 -1.72688800

H -2.47569000 -4.31809100 -2.11816800

C -0.74144400 -3.10827800 -1.67042600

C 0.25932500 -4.04590600 -2.09273900

H -0.06237200 -5.00828800 -2.48056900

C 1.57616900 -3.74527600 -1.99343500

H 2.33210000 -4.46263200 -2.29890100

C 2.01092200 -2.47246900 -1.49023800

C 3.36281500 -2.10133200 -1.40371300

H 4.13791100 -2.80640600 -1.68911800

C 3.67892200 -0.82756700 -0.99026100

H 4.70786700 -0.48535800 -0.95631500

C 2.66402200 0.07450100 -0.62631700

C 1.05275700 -1.53212900 -1.07459500

C -0.34424400 -1.86033100 -1.15133900

C 2.98740400 1.50329600 -0.34806900

C 2.78560000 2.42507900 -1.39246600

C 3.06831200 3.76736200 -1.16574800

H 2.91015400 4.48376900 -1.96962800

C 3.55365700 4.21701800 0.06334500

C 3.78275800 3.27907400 1.06473700

H 4.18475000 3.60808500 2.02083800

C 3.51396100 1.92117700 0.87984800

C 2.27961600 1.96547100 -2.73696400

H 2.22119800 2.80188800 -3.43951800

H 2.93858600 1.20445100 -3.17160100

H 1.28205700 1.52451300 -2.63913900

C 3.77683000 0.94965900 2.00186400

H 4.18924600 0.00425400 1.63583900

H 4.48239800 1.37048200 2.72438200

H 2.85020500 0.71652500 2.53706800

C -3.94944700 -1.40389000 1.76147700

H -4.43249500 -1.24539600 2.72950900

H -4.38934600 -2.30154100 1.31067000

H -2.89229500 -1.61073300 1.95026400

C -2.97958600 1.07682800 -2.55094900

H -3.35058500 1.93079000 -3.12459500

H -1.89912900 1.19779600 -2.41721000

H -3.14349600 0.17001700 -3.14431800

C -6.07516300 3.07843200 0.88822900

H -5.84857900 4.01179700 0.36351600

H -7.12441600 2.83160100 0.68565900

H -5.98403300 3.26277600 1.96355600

C 3.81075200 5.68405800 0.29797700

H 4.19619200 6.17083900 -0.60388000

H 2.88484600 6.20007000 0.57936800

H 4.53408600 5.83998400 1.10426800

C -0.37706300 2.40110300 -0.72285000

C -1.14397600 2.62177800 0.33554900

H -0.11978000 3.14872500 -1.47077100

C -1.83872500 3.82340600 0.91070800

H -1.69846900 4.69048100 0.25900100

H -2.91251200 3.63662400 1.02288100

H -1.43900200 4.06485400 1.90258200

H -1.35397900 1.72087900 1.03228500

Si 0.05010000 -0.47196100 2.33437200

H 0.92145900 0.13372000 3.40586500

H -1.22284500 -0.72878900 3.09636900

C 0.80065700 -2.20895200 2.11110800

C 0.02591700 -3.28537000 1.65113300

C 2.17680200 -2.43910700 2.25921400

C 0.59063400 -4.52212000 1.34988300

H -1.04696300 -3.15377200 1.51524100

C 2.75407300 -3.66902300 1.95342100

H 2.81381000 -1.64141400 2.63197700

C 1.96172900 -4.71789900 1.49385400

H -0.04093300 -5.33233700 0.99377300

H 3.82420200 -3.81186300 2.08247100

H 2.40720800 -5.68032900 1.25672000

**^5^Int-3**

Fe -0.17961200 -0.73586100 0.32478900

N 1.15383100 0.70387000 -0.72384800

N -1.53468100 0.60328800 -0.75208800

C 3.19562900 -0.56948700 -0.43531000

C 3.94719600 -0.73686500 0.73815800

C 4.62976100 -1.93800100 0.93095600

H 5.20097400 -2.07487400 1.84673800

C 4.59511300 -2.96303800 -0.01088800

C 3.85550700 -2.76461000 -1.17451800

H 3.81484000 -3.55522200 -1.92081700

C 3.14106000 -1.59161000 -1.39982300

C 2.48360700 0.70851300 -0.72288200

C 3.20915100 1.86431400 -1.08094300

H 4.29295200 1.82650200 -1.06020100

C 2.54286700 3.00047300 -1.47336800

H 3.08893800 3.89570700 -1.75717000

C 1.13719800 2.98958400 -1.54013600

C 0.36013400 4.09816500 -2.01651100

H 0.88115000 5.00347300 -2.31368200

C -0.98997500 4.01915200 -2.10164800

H -1.57249100 4.85874900 -2.46965700

C -1.68327200 2.83383700 -1.68495300

C -3.08327000 2.69841800 -1.72280800

H -3.69252500 3.51601000 -2.09862100

C -3.66694600 1.53019700 -1.28746200

H -4.74229900 1.39183000 -1.31579600

C -2.85946300 0.48567100 -0.79824800

C -0.94903400 1.74056100 -1.19116700

C 0.49028100 1.80889700 -1.13956400

C -3.44167100 -0.80443000 -0.33741300

C -3.31305800 -1.93853500 -1.15477600

C -3.83814700 -3.14695800 -0.70483600

H -3.74092600 -4.02982500 -1.33312700

C -4.47007500 -3.25571500 0.53285600

C -4.59168200 -2.11160000 1.31895000

H -5.08324900 -2.18041500 2.28689100

C -4.08857300 -0.87945700 0.90524800

C -2.60576500 -1.86594500 -2.48551000

H -2.78506500 -2.77224400 -3.07053500

H -2.93798500 -1.00639500 -3.07834600

H -1.52358900 -1.76951100 -2.34036500

C -4.20814100 0.32923800 1.80051600

H -4.70842800 1.16579600 1.29997000

H -4.77761300 0.08987000 2.70255500

H -3.21903400 0.67644600 2.11940800

C 3.98525200 0.31516200 1.81766500

H 4.83035200 0.15046600 2.49217900

H 4.06469700 1.32921200 1.41481700

H 3.06774300 0.27435400 2.41575900

C 2.29864400 -1.46507100 -2.64332000

H 2.60292400 -2.20042900 -3.39352900

H 1.24900000 -1.65513200 -2.38906600

H 2.36281200 -0.46949200 -3.09457300

C 5.29819300 -4.27208400 0.24012900

H 4.60924100 -4.99448000 0.69504800

H 5.66808600 -4.71272400 -0.69122000

H 6.14573300 -4.14937800 0.92154800

C -4.97913500 -4.58761300 1.02205100

H -5.33245800 -5.20961300 0.19345300

H -4.18039000 -5.13979900 1.53141100

H -5.80192500 -4.46564900 1.73326000

C -0.02709900 -2.71128900 -0.06418200

H -0.77231700 -3.30509700 -0.61370800

C 1.00503500 -3.42205100 0.42067500

H 1.79031200 -2.91419800 0.98944800

C 1.23326100 -4.90151300 0.25671000

H 2.17604800 -5.09623200 -0.27469800

H 1.30928100 -5.40596800 1.22955700

H 0.41959100 -5.36943500 -0.30894600

Si -0.08405300 0.51298200 2.53367100

H -1.28731200 0.47691700 3.43802900

H 1.04194900 0.19795600 3.48129000

C 0.15444200 2.37326000 2.17417200

C -0.90925200 3.15718100 1.69748200

C 1.41085700 2.99283100 2.24574000

C -0.73217500 4.48434500 1.31656500

H -1.90445500 2.72000500 1.61847100

C 1.60273400 4.31642000 1.85462700

H 2.25989700 2.43341300 2.62895000

C 0.52977900 5.06989500 1.38695800

H -1.57989100 5.06116800 0.95479300

H 2.59154200 4.76327900 1.92603300

H 0.67269200 6.10403400 1.08495200

**^1^Ts-2**

Fe 0.61481600 0.04154000 0.27816100

N 0.01769300 1.94471800 0.22131100

N 2.25750400 0.78352900 -0.23173700

C -2.31059700 1.69652800 0.93455100

C -3.27663000 1.39562700 -0.03712800

C -4.32585000 0.54601200 0.30335200

H -5.03861500 0.25286700 -0.46448600

C -4.46021900 0.03307200 1.59358800

C -3.51794600 0.39588400 2.55594400

H -3.61322200 0.00690200 3.56811600

C -2.43104700 1.21256200 2.24308700

C -1.14793000 2.53934300 0.54341900

C -1.31942800 3.92814700 0.48282100

H -2.28515200 4.33561100 0.76240300

C -0.29397400 4.75431100 0.05743200

H -0.43197300 5.83011800 -0.00584400

C 0.92934900 4.17610100 -0.28618500

C 2.08236400 4.91563400 -0.72858000

H 1.99056700 5.99303800 -0.83573300

C 3.25701600 4.29425900 -1.00184000

H 4.11887000 4.86866600 -1.33056900

C 3.40296300 2.86908400 -0.85026500

C 4.58968900 2.16309200 -1.06528800

H 5.48986300 2.67646100 -1.38819000

C 4.58792600 0.78835000 -0.83431700

H 5.49402300 0.20532600 -0.96845500

C 3.43927400 0.12932300 -0.41741000

C 2.27002300 2.13992900 -0.43807300

C 1.04138200 2.77441700 -0.16971900

C 3.40099600 -1.32938800 -0.12189300

C 3.54273300 -1.76846400 1.20479000

C 3.41613300 -3.12937800 1.48133900

H 3.51973100 -3.47217700 2.50909400

C 3.14165200 -4.05638400 0.47648100

C 3.00846900 -3.59632500 -0.83377000

H 2.78395800 -4.30667400 -1.62666300

C 3.14147800 -2.24635300 -1.15268500

C 3.76332400 -0.77703100 2.31909300

H 2.88826700 -0.12268600 2.42122500

H 3.92621400 -1.28483900 3.27411900

H 4.62437600 -0.13086500 2.11903800

C 2.99888300 -1.77287100 -2.57715000

H 3.96161800 -1.43413700 -2.97761000

H 2.62311000 -2.57293400 -3.22072400

H 2.30360900 -0.93001700 -2.64446300

C -3.17496300 1.96813200 -1.42901400

H -3.94256600 1.54284100 -2.07905100

H -3.29962000 3.05725500 -1.41674800

H -2.19840700 1.75971800 -1.87982000

C -1.36290300 1.49921800 3.26643800

H -1.65330900 1.13239500 4.25526400

H -0.42527900 1.00543300 2.98072500

H -1.15326700 2.57170500 3.34545900

C -5.60077100 -0.89626300 1.92146500

H -6.55527700 -0.35759400 1.95164300

H -5.68895100 -1.67714600 1.15820600

H -5.45927600 -1.37956900 2.89304400

C 3.00584100 -5.52525400 0.78952300

H 3.91281600 -6.07185500 0.50446800

H 2.84047800 -5.69265700 1.85820000

H 2.16912600 -5.97135600 0.24202700

C -0.90763600 -1.05123000 0.65644500

H -1.95400600 -0.93342400 0.91780200

C -0.03912400 -1.69834900 1.47682100

H 0.96054400 -1.96828600 1.09525400

C -0.31372600 -2.22280600 2.85728300

H -0.27424400 -3.31929900 2.87430600

H 0.44066800 -1.86106800 3.56563200

H -1.30033100 -1.90230100 3.20662500

Si -0.58553100 -1.03233700 -1.42890200

H -0.39588400 -0.00225300 -2.51079000

H 0.22893300 -2.23302900 -1.79569400

C -2.38996900 -1.54047000 -1.79715400

C -3.09686000 -2.47972400 -1.02902400

C -3.06084700 -0.98134600 -2.89202800

C -4.41358500 -2.82193300 -1.32167700

H -2.61251100 -2.94960500 -0.17605200

C -4.38371900 -1.30763800 -3.18927500

H -2.54256700 -0.26117900 -3.52120600

C -5.06794200 -2.22547600 -2.39858200

H -4.93245700 -3.55431800 -0.70810200

H -4.87738800 -0.84614600 -4.04065000

H -6.09963100 -2.48200100 -2.62326700

**^3^Ts-2**

Fe 0.49324900 0.05571700 0.12840200

N 0.00696900 2.10742200 0.19608200

N 2.27700100 0.81043100 -0.20749800

C -2.28411500 1.86417400 0.90341700

C -3.28740800 1.52295500 -0.01675700

C -4.29736100 0.65265100 0.38484200

H -5.04497900 0.34004500 -0.34081200

C -4.34997800 0.14315600 1.68201800

C -3.35579200 0.51826900 2.58406300

H -3.37922800 0.12412500 3.59828000

C -2.30956500 1.36228300 2.21127800

C -1.14850000 2.72332400 0.46466600

C -1.29159500 4.11288300 0.34049100

H -2.25004100 4.56334900 0.57541200

C -0.22046700 4.88282800 -0.07717700

H -0.31909700 5.95991400 -0.18297200

C 1.00601300 4.26393300 -0.34970400

C 2.19254400 4.96889500 -0.74955500

H 2.13483900 6.04504300 -0.88824700

C 3.36460100 4.31127100 -0.93285500

H 4.26041100 4.85457400 -1.22108700

C 3.46564900 2.88779900 -0.74198900

C 4.66273200 2.17044700 -0.86712200

H 5.58269600 2.68089500 -1.13557100

C 4.64499500 0.80114800 -0.62798600

H 5.55275200 0.21108000 -0.70403500

C 3.45733000 0.15080400 -0.30416500

C 2.30304300 2.17223500 -0.39088600

C 1.07527800 2.86303400 -0.18598000

C 3.41658500 -1.32195300 -0.08131800

C 3.53571800 -1.83447900 1.21832500

C 3.47284400 -3.21391600 1.40656800

H 3.56173500 -3.61490500 2.41453700

C 3.27166800 -4.08881700 0.33945400

C 3.13488400 -3.55257200 -0.94083500

H 2.95643500 -4.21950600 -1.78167700

C 3.21031800 -2.17930800 -1.17184000

C 3.65114700 -0.90346700 2.39812400

H 4.46653300 -0.18337700 2.27185900

H 2.72495000 -0.32502000 2.50919300

H 3.82156700 -1.45780600 3.32568900

C 3.04139200 -1.62138900 -2.56241900

H 3.96125600 -1.13876200 -2.91253600

H 2.77645700 -2.40969100 -3.27222900

H 2.24794800 -0.86619300 -2.58186000

C -3.25625800 2.05877300 -1.42674500

H -4.06938100 1.63276900 -2.01918500

H -3.35466900 3.15031500 -1.44342700

H -2.31162500 1.80991300 -1.92401500

C -1.19536900 1.66764600 3.18066100

H -1.46356800 1.36390200 4.19664800

H -0.28448700 1.12756400 2.89281600

H -0.94687000 2.73431900 3.19536700

C -5.46114900 -0.79203900 2.08455900

H -5.23070100 -1.30966000 3.02089200

H -6.40228400 -0.24741300 2.22720000

H -5.62987100 -1.54430400 1.30714600

C 3.21226800 -5.57903000 0.56291900

H 4.20804700 -6.02962500 0.47032300

H 2.83537300 -5.81726800 1.56267000

H 2.56181600 -6.06556400 -0.17065400

C -0.78997600 -1.21823100 0.59379600

H -1.70366300 -1.00660600 1.15424000

C 0.10776200 -2.14427700 1.05652300

H 0.88373700 -2.53179100 0.39926700

C 0.04326500 -2.78459400 2.41444900

H -0.06782300 -3.87369300 2.33180200

H 0.97386900 -2.60361700 2.96630000

H -0.78991200 -2.39021300 3.00550500

Si -0.81587700 -1.13117900 -1.48961400

H -0.74617700 -0.05192500 -2.53893300

H 0.00278700 -2.28217500 -1.98121000

C -2.64409200 -1.63459900 -1.66874000

C -3.26877700 -2.55232200 -0.81077500

C -3.42454100 -1.08364700 -2.69360500

C -4.60984500 -2.89415800 -0.96031000

H -2.69793600 -3.00661300 -0.00451500

C -4.77174600 -1.40886100 -2.84541700

H -2.97263900 -0.37443200 -3.38353400

C -5.37062000 -2.31406600 -1.97395700

H -5.06411100 -3.61381900 -0.28372200

H -5.35184400 -0.95821100 -3.64662700

H -6.41959900 -2.57345700 -2.08831300

**^5^Ts-2**

Fe 0.32937400 0.13471300 0.26765700

N -0.17035900 2.14727400 0.27078800

N 2.18718200 0.99010800 -0.12618400

C -2.47988400 1.74962500 0.82313100

C -3.39387200 1.37809800 -0.17295100

C -4.38678300 0.45092600 0.13812000

H -5.07292700 0.13173500 -0.64205200

C -4.49049300 -0.11003900 1.40729300

C -3.57673500 0.28454800 2.38506400

H -3.64756400 -0.14386400 3.38345600

C -2.56611300 1.20410500 2.11446700

C -1.38158700 2.69838000 0.48769800

C -1.59101500 4.06868300 0.39776400

H -2.58319200 4.46209900 0.58989500

C -0.53072100 4.91796000 0.06197400

H -0.68536800 5.99039400 -0.01651700

C 0.72996500 4.37669400 -0.17285300

C 1.89085800 5.15259800 -0.53137000

H 1.77949000 6.22894700 -0.63041400

C 3.09168300 4.56319500 -0.74536700

H 3.95771600 5.16075600 -1.01742100

C 3.26122800 3.13673700 -0.62056900

C 4.46879700 2.47544200 -0.82093000

H 5.35982400 3.03537300 -1.09071000

C 4.52119900 1.08643300 -0.66066600

H 5.45074900 0.54363800 -0.79293000

C 3.37635000 0.38073100 -0.31249000

C 2.13002200 2.35785500 -0.26903900

C 0.87934300 2.97226100 -0.05149000

C 3.42718000 -1.09475900 -0.10111100

C 3.57927600 -1.60438300 1.19796100

C 3.67787600 -2.98365100 1.37049500

H 3.80243100 -3.38267500 2.37530700

C 3.59792600 -3.86437600 0.29241200

C 3.42646700 -3.33363400 -0.98521700

H 3.35258900 -4.00735100 -1.83633200

C 3.34754200 -1.95838600 -1.20313300

C 3.59966200 -0.68398500 2.39200300

H 3.87511800 -1.22524500 3.30145400

H 4.30515200 0.14238700 2.25512500

H 2.60783800 -0.24312700 2.54871500

C 3.19085100 -1.41368400 -2.60146700

H 4.12696400 -0.96929200 -2.95978500

H 2.90157300 -2.20500300 -3.29836100

H 2.42464000 -0.63322800 -2.64451600

C -3.29528400 1.94815000 -1.56660100

H -4.02626100 1.47641900 -2.22789000

H -3.47161200 3.02952100 -1.57386300

H -2.29953800 1.77981400 -1.99319900

C -1.57024600 1.57478200 3.18603600

H -1.92475800 1.26343000 4.17284100

H -0.60434900 1.08561600 3.00846300

H -1.38569700 2.65373500 3.21172900

C -5.55810400 -1.12613500 1.72304600

H -6.12648500 -1.39245900 0.82769200

H -5.11645200 -2.04527700 2.12444300

H -6.26043300 -0.74449700 2.47365700

C 3.64101900 -5.35573800 0.50999700

H 4.07553800 -5.87320200 -0.35097900

H 4.22941400 -5.61377600 1.39625100

H 2.63011000 -5.75544300 0.65785600

C -0.36089600 -1.44914500 1.18607300

H -1.26066600 -1.28096000 1.79255700

C 0.24222900 -2.64550800 1.27617000

H 1.08316900 -2.87001000 0.62047700

C -0.14456700 -3.75786800 2.20867600

H -0.41887500 -4.66058800 1.64626700

H 0.69950000 -4.03312600 2.85439600

H -0.98984200 -3.47630700 2.84559700

Si -0.49069100 -1.08091700 -1.58945800

H -0.31664600 -0.21717100 -2.81144800

H 0.38552600 -2.26941100 -1.81398200

C -2.28859800 -1.65106700 -1.70290300

C -2.86379500 -2.54731200 -0.78966000

C -3.08784800 -1.21096300 -2.76747900

C -4.17585500 -2.98620600 -0.93504100

H -2.27608300 -2.90643700 0.04961400

C -4.40632300 -1.63851400 -2.91461300

H -2.67408700 -0.52000200 -3.49836700

C -4.95459800 -2.52990100 -1.99657200

H -4.59393100 -3.68518700 -0.21541700

H -5.00168300 -1.28094900 -3.75065800

H -5.97962100 -2.87233600 -2.11099800

**^1^Int-4**

Fe 0.00971900 0.27026100 0.31146900

N -1.63627200 1.25969700 -0.13951700

N 0.85060900 1.83751600 -0.31018100

C -3.24325800 -0.56786800 -0.16134500

C -3.01151900 -1.29755000 -1.34149900

C -3.22927400 -2.67107900 -1.33786500

H -3.02865100 -3.24048200 -2.24296200

C -3.67684800 -3.33636900 -0.19556100

C -3.95790600 -2.58225800 0.94012700

H -4.33133800 -3.08127700 1.83199700

C -3.76342300 -1.20013600 0.97358300

C -2.93322100 0.88884200 -0.17607100

C -3.96231600 1.82508200 -0.31578600

H -4.98528300 1.46372600 -0.32191300

C -3.68294300 3.17684400 -0.45363400

H -4.48349100 3.90512400 -0.54855100

C -2.34838400 3.58347300 -0.50243900

C -1.90759400 4.94097200 -0.69568500

H -2.66118600 5.71755200 -0.79425800

C -0.58791800 5.25190000 -0.75616400

H -0.27472100 6.28219500 -0.90180100

C 0.43081900 4.23917400 -0.63542100

C 1.80645000 4.47710900 -0.69248400

H 2.19037300 5.48099200 -0.84513300

C 2.66931000 3.39293500 -0.54476300

H 3.74500900 3.53530700 -0.58149700

C 2.18445000 2.10430000 -0.35501000

C 0.00522400 2.90983800 -0.44455800

C -1.36294700 2.58453300 -0.35976100

C 3.06536600 0.91935400 -0.17310100

C 3.69098100 0.68713300 1.06140600

C 4.43154700 -0.48440800 1.22862400

H 4.89924800 -0.67798200 2.19195700

C 4.58503500 -1.40723200 0.19781700

C 3.99475600 -1.12957200 -1.03498600

H 4.11791900 -1.83341900 -1.85395100

C 3.22732600 0.01232400 -1.23612500

C 3.61010400 1.68816900 2.18886500

H 3.74871800 1.19692400 3.15683500

H 4.39361900 2.44955800 2.08763500

H 2.65149400 2.21290700 2.20623500

C 2.60771300 0.29448500 -2.58158500

H 3.00910900 1.21912300 -3.01264700

H 2.79979200 -0.52418600 -3.28041900

H 1.52390900 0.41936100 -2.49095600

C -2.53429600 -0.60345800 -2.59496800

H -3.24398200 0.16844100 -2.91395500

H -1.57266700 -0.10249100 -2.43466800

H -2.41901000 -1.31817200 -3.41478700

C -4.13647100 -0.42675500 2.21542000

H -3.96353300 -1.02454100 3.11549800

H -3.56783100 0.50132800 2.31198000

H -5.20046700 -0.15881600 2.19581300

C -3.82637500 -4.83625200 -0.19083300

H -4.21070700 -5.20338600 -1.14822700

H -2.85594600 -5.31711700 -0.01956600

H -4.50596000 -5.16925600 0.59945600

C 5.34100300 -2.69288800 0.41231600

H 5.83427900 -3.02465800 -0.50701000

H 6.10277500 -2.58852900 1.19127900

H 4.65551100 -3.49061400 0.72293300

C -0.52169300 -0.66358900 2.04791800

H -1.58819500 -0.65902000 2.26206800

C 0.17689900 0.58195900 2.22837700

H 1.24458500 0.50503400 2.47000100

C -0.47206400 1.79090200 2.86147900

H -0.36885200 1.78011000 3.95532300

H -0.02448500 2.72409500 2.49577900

H -1.54286200 1.82787100 2.62954400

Si 0.30213100 -2.30696900 2.08167900

H 1.55191900 -2.36305200 2.90166400

H -0.59011400 -3.40369400 2.56862400

C 0.82362700 -2.72125800 0.30798700

C 0.21478800 -2.02586800 -0.74744200

C 1.78498900 -3.68543000 -0.01826500

C 0.53146000 -2.29825100 -2.07876800

H -0.64560400 -1.36930800 -0.52170500

C 2.11009300 -3.96217600 -1.34215400

H 2.28266200 -4.23615300 0.77745400

C 1.48387200 -3.26595700 -2.37590300

H 0.03462400 -1.76004900 -2.87959200

H 2.85523300 -4.71913900 -1.57213000

H 1.73703000 -3.47986900 -3.41063300

**^3^Int-4**

Fe -0.61844900 -0.06137600 0.33777500

N -0.40674400 -2.11177900 0.07627200

N -2.52573400 -0.49707100 -0.21633800

C 1.90569500 -2.23515700 0.74238700

C 2.88687800 -1.84325800 -0.17795100

C 4.01090100 -1.15799600 0.28548200

H 4.76148900 -0.82810100 -0.42947200

C 4.17833300 -0.86160700 1.63616100

C 3.19072200 -1.27019400 2.53426800

H 3.31020300 -1.04895200 3.59338600

C 2.05111100 -1.94974200 2.10917600

C 0.66330300 -2.90891700 0.26526100

C 0.61789200 -4.27857400 0.04556100

H 1.51216900 -4.86899200 0.21135100

C -0.57864700 -4.87359700 -0.38225900

H -0.62873300 -5.94459100 -0.55977500

C -1.70517400 -4.08807000 -0.57187800

C -2.98362800 -4.59915200 -1.00019600

H -3.07438600 -5.66570800 -1.18875600

C -4.04921100 -3.78061300 -1.16620900

H -5.00442600 -4.18258300 -1.49391200

C -3.95811600 -2.36052400 -0.91941600

C -5.01363700 -1.47639100 -1.08312600

H -5.98286500 -1.84024000 -1.41219500

C -4.81101300 -0.10373800 -0.82941900

H -5.61303600 0.61438100 -0.96191500

C -3.57424900 0.34197900 -0.40918400

C -2.70494400 -1.84324500 -0.48599700

C -1.59577300 -2.68825600 -0.32883600

C -3.29358300 1.78227100 -0.14261100

C -3.50915900 2.30874400 1.13728800

C -3.17836100 3.64198200 1.38308400

H -3.34011300 4.05217300 2.37788400

C -2.63412700 4.45462900 0.39101800

C -2.42040800 3.90390600 -0.87407300

H -1.98341600 4.52174600 -1.65613200

C -2.73891700 2.57798500 -1.15833800

C -4.05725400 1.43719700 2.23992700

H -4.10046000 1.98077400 3.18822100

H -5.06521000 1.07894200 2.00416900

H -3.43060800 0.54902300 2.38147200

C -2.46173600 1.99722000 -2.52267400

H -3.36557400 1.56166400 -2.96167300

H -2.07719000 2.76033200 -3.20501000

H -1.71871300 1.19336600 -2.46075600

C 2.72701400 -2.13670000 -1.64925300

H 3.52687100 -1.66450200 -2.22594600

H 2.74929700 -3.21453900 -1.84553100

H 1.76894000 -1.76126300 -2.02561000

C 0.98138900 -2.34726100 3.09612500

H 1.29584400 -2.14102900 4.12320200

H 0.05111500 -1.79799600 2.90744000

H 0.74161500 -3.41300500 3.01570400

C 5.38126600 -0.09146700 2.11820500

H 6.16643000 -0.06595300 1.35758500

H 5.11271500 0.94729200 2.34591900

H 5.79716500 -0.53165300 3.03097300

C -2.27342700 5.89260000 0.66806500

H -2.87088300 6.57422100 0.05180500

H -2.44400400 6.15164400 1.71720900

H -1.21913900 6.08493700 0.43986000

C 1.01850200 1.11718300 0.47294200

H 1.78732600 0.59298600 1.04649000

C -0.05957400 1.69947100 1.19838000

H -0.62165600 2.50497200 0.72180100

C -0.10062700 1.71881200 2.71090200

H 0.36310200 2.63603500 3.09911800

H -1.13086500 1.69543000 3.08386000

H 0.44185700 0.86731000 3.13776400

Si 1.46704300 1.60894000 -1.26284000

H 1.01941900 0.63600400 -2.30587700

H 0.79816500 2.90191100 -1.59264700

C 3.33485500 1.70903500 -1.44460300

C 4.12927000 2.24583000 -0.42249400

C 3.98007800 1.18648500 -2.57254800

C 5.51736300 2.26259400 -0.52283000

H 3.65731200 2.63943000 0.47540200

C 5.36952100 1.19113200 -2.67654300

H 3.39010100 0.75245900 -3.37687700

C 6.14073800 1.72836000 -1.64876800

H 6.11493200 2.68124500 0.28219800

H 5.84963800 0.77330600 -3.55698600

H 7.22440000 1.73238400 -1.72583800

**^5^Int-4**

Fe -0.59810100 -0.07058700 0.34503900

N -0.42373200 -2.12697400 0.08388900

N -2.52303700 -0.46909500 -0.25371100

C 1.88825800 -2.27171700 0.72688900

C 2.85333500 -1.85268200 -0.19900800

C 3.98124300 -1.17287400 0.26272100

H 4.72162700 -0.82719000 -0.45539900

C 4.16557000 -0.90257700 1.61686600

C 3.19265600 -1.33467900 2.51989900

H 3.32581300 -1.13470500 3.58162500

C 2.05171100 -2.01383000 2.09696000

C 0.63971800 -2.93570600 0.25055200

C 0.58090400 -4.30097200 0.01230500

H 1.46806400 -4.90451800 0.16793800

C -0.62870100 -4.87601800 -0.41585500

H -0.69423500 -5.94487500 -0.60124200

C -1.74474400 -4.07540300 -0.59686400

C -3.03155800 -4.56817300 -1.02541400

H -3.13509200 -5.63248400 -1.22034900

C -4.08922800 -3.73795500 -1.18023600

H -5.05077000 -4.12832700 -1.50368400

C -3.98043000 -2.31949700 -0.92939900

C -5.02594200 -1.42350700 -1.08115800

H -6.00096400 -1.77780900 -1.40438900

C -4.81390000 -0.05299300 -0.82043800

H -5.61164500 0.67181900 -0.93910100

C -3.56409700 0.37721300 -0.41708700

C -2.71554300 -1.81713800 -0.50505600

C -1.61966800 -2.67745600 -0.34210900

C -3.26501200 1.81208100 -0.13935000

C -3.48163500 2.33161200 1.14327000

C -3.13275200 3.65798000 1.40165400

H -3.29575400 4.06292900 2.39838200

C -2.56884400 4.46988300 0.42001300

C -2.35481500 3.92573400 -0.84783000

H -1.90398300 4.54309800 -1.62234700

C -2.69262300 2.60745300 -1.14535100

C -4.05361700 1.46215400 2.23565900

H -5.07564200 1.14527700 2.00100800

H -3.45995600 0.54932400 2.36028800

H -4.07178400 1.99241200 3.19224500

C -2.42365300 2.03606600 -2.51529000

H -3.33218400 1.60889000 -2.95293200

H -2.03888800 2.80294100 -3.19330600

H -1.68475900 1.22804600 -2.46401400

C 2.66559200 -2.10313500 -1.67496100

H 3.50342800 -1.69614500 -2.24706300

H 2.58244000 -3.17345200 -1.89193800

H 1.74523800 -1.63118500 -2.03845500

C 1.00111400 -2.44438300 3.09056900

H 1.32485700 -2.24872600 4.11684900

H 0.05946400 -1.90879900 2.92128400

H 0.77910300 -3.51272900 2.99572600

C 5.37077900 -0.13494300 2.09732000

H 5.10509800 0.90503400 2.32315700

H 5.78601600 -0.57454300 3.01057900

H 6.15565600 -0.11256100 1.33625300

C -2.18981400 5.90048600 0.71037300

H -2.77831300 6.59502500 0.09995400

H -2.35805400 6.15221200 1.76167300

H -1.13309600 6.08168600 0.48446800

C 1.02784900 1.12469700 0.49374400

H 1.80937500 0.57651000 1.02694300

C -0.03521600 1.65825100 1.27140800

H -0.62537600 2.47108900 0.84369000

C -0.04086800 1.60751000 2.78323800

H 0.40433500 2.52003900 3.20267900

H -1.06056300 1.53481200 3.17854100

H 0.53721800 0.75420900 3.15608200

Si 1.45144400 1.68407000 -1.22866500

H 0.96047300 0.76201400 -2.29831500

H 0.81305900 3.00592700 -1.49421600

C 3.31796300 1.73694500 -1.43741000

C 4.14225300 2.24537800 -0.42429900

C 3.93142600 1.20151000 -2.57690500

C 5.52854600 2.22253700 -0.54461900

H 3.69516900 2.64846700 0.48214700

C 5.31874800 1.16682500 -2.70101600

H 3.31725500 0.78822600 -3.37389200

C 6.11987100 1.67646400 -1.68218400

H 6.14983800 2.61935900 0.25345400

H 5.77413700 0.73976700 -3.59009600

H 7.20193900 1.64883700 -1.77478700

**^3^Ts-1a**

Fe 0.08835000 0.77729400 -0.29431000

N 1.20322300 -0.91426200 0.67803500

N -1.39422300 -0.27390600 0.60817200

C 3.45731200 -0.09076900 0.35730900

C 4.16811600 -0.16253300 -0.84766700

C 5.04550500 0.87410700 -1.16931800

H 5.58917600 0.82965400 -2.11079800

C 5.23967900 1.96035500 -0.31866100

C 4.54078700 1.99069600 0.88762300

H 4.69049000 2.82552900 1.56922400

C 3.64918900 0.98212900 1.24299200

C 2.50863800 -1.17251400 0.74683200

C 2.99263500 -2.39992700 1.23522400

H 4.06452500 -2.56418200 1.26962200

C 2.10855100 -3.35804500 1.68125200

H 2.46625000 -4.30872600 2.06767900

C 0.73089800 -3.09087100 1.64783700

C -0.26596900 -4.02188300 2.09359900

H 0.05884900 -4.98332000 2.48135400

C -1.58291800 -3.71512700 2.01895000

H -2.33616000 -4.42662400 2.34449200

C -2.02153700 -2.44355700 1.51537700

C -3.37335600 -2.06889600 1.45187000

H -4.14515200 -2.76852000 1.75897900

C -3.69285500 -0.79697100 1.03260500

H -4.72094500 -0.45051000 1.01679000

C -2.68145200 0.09562000 0.64082000

C -1.06703800 -1.50995100 1.07393900

C 0.33034400 -1.84309800 1.12954100

C -2.99678800 1.52622100 0.36093800

C -2.78276700 2.44599500 1.40535400

C -3.04246100 3.79199600 1.17805500

H -2.87267700 4.50662900 1.98118600

C -3.51562600 4.24961200 -0.05351700

C -3.75734000 3.31555800 -1.05465000

H -4.14980600 3.65073500 -2.01256800

C -3.51188700 1.95283900 -0.86781300

C -2.28669700 1.97746900 2.75066200

H -2.18641400 2.81696100 3.44486900

H -2.97492500 1.25003300 3.19742600

H -1.31086400 1.49074500 2.65187200

C -3.79012700 0.98567500 -1.98998700

H -4.21341200 0.04594100 -1.62188700

H -4.49320000 1.41545100 -2.70966800

H -2.86908400 0.74163400 -2.53006400

C 4.00873700 -1.33909300 -1.77845500

H 4.49561900 -1.14704200 -2.73856900

H 4.45521600 -2.24648700 -1.35378700

H 2.95444200 -1.55091900 -1.97758500

C 2.91547000 1.03938900 2.55848300

H 3.24833200 1.89410600 3.15410300

H 1.83784900 1.13616200 2.38999600

H 3.08036200 0.12892900 3.14655200

C 6.14962800 3.09731700 -0.70894200

H 5.57779300 3.90309600 -1.18592500

H 6.65205800 3.52536700 0.16437700

H 6.91581800 2.77215400 -1.41948500

C -3.74505100 5.72148400 -0.28610800

H -4.37619900 5.89577000 -1.16273500

H -4.22482900 6.19216100 0.57863000

H -2.79302800 6.23907400 -0.45406200

C 0.38139900 2.41281500 0.72159500

C 1.05556800 2.48735100 -0.37188500

H 0.17181400 3.03456700 1.58281300

C 1.95088900 3.32193500 -1.21764800

H 2.22323100 4.24857600 -0.70129000

H 2.86696400 2.77489700 -1.46355800

H 1.45628500 3.57874300 -2.16155900

H 0.96899300 1.19457900 -1.45994000

Si -0.04848300 -0.47145400 -2.32430500

H -0.90399000 0.17462200 -3.37868900

H 1.21892400 -0.77705400 -3.06632500

C -0.83112600 -2.18958300 -2.08633700

C -0.05918000 -3.27758400 -1.65146300

C -2.21194200 -2.39959000 -2.21335000

C -0.63348300 -4.50988100 -1.35071000

H 1.01728000 -3.15775200 -1.53398400

C -2.79747200 -3.62638600 -1.91144600

H -2.84583100 -1.59055200 -2.56519000

C -2.00896900 -4.68775700 -1.47458700

H -0.00634800 -5.33046600 -1.01135100

H -3.87097400 -3.75640700 -2.02366200

H -2.46160600 -5.64697400 -1.23829000

**^3^Ts-1aʹ**

Fe -0.03343300 -0.80662100 -0.38090600

N -1.39295300 0.64288500 0.67694800

N 1.28089800 0.45081000 0.55022200

C -3.48627400 -0.50004800 0.26767300

C -4.17702000 -0.43653900 -0.95086900

C -4.83103100 -1.57949000 -1.40708700

H -5.35015400 -1.54252600 -2.36281700

C -4.83438600 -2.76550900 -0.67349700

C -4.17361500 -2.78745300 0.55272700

H -4.17370100 -3.70184200 1.14242300

C -3.49948000 -1.67002400 1.04033800

C -2.72364500 0.67899500 0.76712400

C -3.39405000 1.76608300 1.35511000

H -4.47771400 1.74712000 1.40496100

C -2.67307300 2.82242700 1.86768400

H -3.17457100 3.67097600 2.32521600

C -1.27155000 2.79295800 1.80151600

C -0.43829800 3.85268300 2.29317200

H -0.91254000 4.71606400 2.75118300

C 0.90819400 3.78755700 2.16844000

H 1.53815600 4.59824900 2.52291300

C 1.54346500 2.64779200 1.56914500

C 2.93627300 2.53481500 1.43818100

H 3.58070000 3.34557900 1.76521500

C 3.46282600 1.37241500 0.92281300

H 4.53492000 1.22388200 0.84799000

C 2.61537600 0.32898300 0.51595500

C 0.75453700 1.58343500 1.09478000

C -0.67757400 1.66812000 1.19304700

C 3.19768800 -1.00036200 0.17065200

C 3.23537200 -1.95938000 1.19782200

C 3.79291000 -3.20706200 0.93441400

H 3.81960900 -3.95163100 1.72778200

C 4.31893600 -3.52325000 -0.31781800

C 4.29813700 -2.54483200 -1.30794900

H 4.72113000 -2.76756900 -2.28543800

C 3.75648200 -1.27850300 -1.08336100

C 2.69567400 -1.63362000 2.56764800

H 2.77980900 -2.49319200 3.23838200

H 3.23935500 -0.79512500 3.01911400

H 1.64105300 -1.34744100 2.50570900

C 3.78087000 -0.24988300 -2.18403700

H 4.07743500 0.73536300 -1.80948700

H 4.48339600 -0.53888000 -2.97089500

H 2.79262400 -0.14246200 -2.64265800

C -4.23207200 0.83997500 -1.75330900

H -4.66450400 0.66168700 -2.74173300

H -4.84742100 1.59719800 -1.25202100

H -3.23718800 1.26833900 -1.90082000

C -2.80387400 -1.71766700 2.37642300

H -2.89179100 -2.70936700 2.82906400

H -1.74043600 -1.48643300 2.26515400

H -3.23206100 -0.98654300 3.07305500

C -5.56517300 -3.98149500 -1.18570600

H -5.23848300 -4.89016700 -0.67107300

H -6.64650900 -3.88270400 -1.03156100

H -5.39988800 -4.12157800 -2.25906000

C 4.93778600 -4.87338700 -0.57914500

H 4.53627800 -5.63372000 0.09777700

H 4.75509800 -5.20219500 -1.60703200

H 6.02431200 -4.84046600 -0.43222900

C -0.14172400 -2.56144800 0.51279300

C -0.75639400 -2.58529700 -0.62062100

H -0.79743600 -1.19668800 -1.64089300

Si -0.15775300 0.56203600 -2.34063100

H 0.79259400 0.13735900 -3.42302000

H -1.46316500 0.68395500 -3.06646300

C 0.31940200 2.37212800 -2.00148400

C -0.61401800 3.28764400 -1.49262700

C 1.63886200 2.82356400 -2.14910500

C -0.25041100 4.58463200 -1.14049800

H -1.65075100 2.98169900 -1.35857200

C 2.01450600 4.11708500 -1.79597300

H 2.38973900 2.15328700 -2.55742100

C 1.06981400 5.00333500 -1.28557200

H -0.99782900 5.26795100 -0.74541500

H 3.04612800 4.43506400 -1.92474200

H 1.35711700 6.01426700 -1.00916200

C 0.14600800 -3.51626200 1.62218200

H -0.51139000 -4.39416200 1.58614400

H 1.18123600 -3.86746600 1.54536700

H 0.03899600 -3.04995000 2.60737100

H -1.33669300 -3.20683500 -1.28598900

**^3^Ts-1c**

Fe 0.08526000 0.71582100 -0.42801500

N 1.20234000 -0.93585700 0.51431500

N -1.38728200 -0.35202100 0.42864100

C 2.49925200 -1.20920000 0.50430100

C 3.03361900 -2.37359700 1.06917300

H 4.10331700 -2.54768700 1.03114800

C 2.18105700 -3.28487500 1.66197200

H 2.56504800 -4.19979700 2.10481300

C 0.80097600 -3.02624700 1.67924600

C -0.17505500 -3.92137300 2.23780300

H 0.16961800 -4.84583700 2.69236900

C -1.49725800 -3.62990600 2.18517300

H -2.23015500 -4.31882700 2.59514900

C -1.97162900 -2.41484200 1.57901100

C -3.33083600 -2.07661300 1.48695700

H -4.08551500 -2.74976300 1.88282100

C -3.68216300 -0.88669100 0.88300600

H -4.72056000 -0.58841400 0.79062900

C -2.68387500 -0.05406000 0.37052000

C -1.03656100 -1.51533000 1.03549600

C 0.36359900 -1.82630500 1.08500900

C 0.26005400 2.23105700 0.76286600

C 1.00773800 2.44653100 -0.26406000

H -0.00656000 2.71340400 1.69399500

C 1.93388100 3.41157300 -0.92028700

H 2.08379500 4.29601400 -0.29291500

H 2.90876900 2.94994000 -1.11287000

H 1.53405100 3.73379600 -1.88838400

H 1.01135100 1.31936000 -1.48140700

Si 0.06180600 -0.57509600 -2.44242800

H -0.66267400 0.03242800 -3.61140200

H 1.40564900 -0.96743100 -2.98746600

C -0.82086100 -2.23325300 -2.14914000

C -0.12865300 -3.35421200 -1.66588400

C -2.21807100 -2.33584400 -2.23687000

C -0.79373300 -4.51689100 -1.28313700

H 0.95616200 -3.31387500 -1.57638500

C -2.89266100 -3.49268600 -1.85622800

H -2.79277700 -1.49015600 -2.61058200

C -2.18139300 -4.58900800 -1.37313200

H -0.22831500 -5.36624300 -0.90802300

H -3.97580300 -3.54084600 -1.93775100

H -2.70388300 -5.49365800 -1.07379200

H 3.13496800 -0.47363200 0.02049100

H -2.93310800 0.88485900 -0.11264400

**^3^Ts-1cʹ**

Fe 0.08967800 0.72747600 -0.48288300

N 1.19335000 -0.91009500 0.48519500

N -1.39151100 -0.32380800 0.38962300

C 2.49097200 -1.18168900 0.48476700

C 3.02439600 -2.33294800 1.07676200

H 4.09433000 -2.50690000 1.04511500

C 2.17139900 -3.23250100 1.68714700

H 2.55530900 -4.13764200 2.14966400

C 0.79117600 -2.97453200 1.69605200

C -0.18673000 -3.85706100 2.27151500

H 0.15631200 -4.77220200 2.74574800

C -1.50881600 -3.56622100 2.21051900

H -2.24269300 -4.24625600 2.63333400

C -1.98146800 -2.36342000 1.57884900

C -3.33985200 -2.02454600 1.47848700

H -4.09648800 -2.68841600 1.88608300

C -3.68830600 -0.84581300 0.85077500

H -4.72613800 -0.54810900 0.75050000

C -2.68793700 -0.02512600 0.32367700

C -1.04435500 -1.47636000 1.01867700

C 0.35488500 -1.78797100 1.07512400

C 0.30479200 2.19572500 0.76611600

C 1.00288500 2.44067400 -0.28710900

H 0.97377900 1.36707200 -1.55727500

Si 0.05674500 -0.57635200 -2.48546600

H -0.68126300 0.00680600 -3.65821600

H 1.39872700 -0.96986400 -3.03426600

C -0.81620000 -2.23524000 -2.16270400

C -0.11819300 -3.34471800 -1.66140900

C -2.21317200 -2.34612000 -2.24534700

C -0.77708300 -4.50370600 -1.25712300

H 0.96662200 -3.29798900 -1.57498600

C -2.88178900 -3.49917200 -1.84316700

H -2.79247400 -1.50970800 -2.63268900

C -2.16460400 -4.58381400 -1.34262800

H -0.20688800 -5.34387900 -0.86869000

H -3.96487100 -3.55375600 -1.92174300

H -2.68223700 -5.48547500 -1.02631100

H 3.12626900 -0.45577700 -0.01386000

H -2.93593000 0.90334000 -0.18041100

C -0.07101900 2.69134100 2.11347500

H 0.27877100 1.98790900 2.87799600

H 0.34535400 3.68084600 2.33832800

H -1.16101900 2.74302900 2.21440900

H 1.64314900 3.16763900 -0.76417700

**^3^Ts-1b**

Fe -0.41364600 -0.16759500 0.76772600

N 1.08675100 -0.56626900 2.39855400

N -1.58879000 -0.53730900 2.35426600

C 3.13037400 -0.11435400 1.15661400

C 3.21923600 -1.01387200 0.09494400

C 3.97966100 -0.70842100 -1.03469300

C 4.62954300 0.52798800 -1.09694500

C 4.55669600 1.44010600 -0.04178800

C 3.80626400 1.10228100 1.08755600

C 2.41402000 -0.49321400 2.40719300

C 3.16313300 -0.77494300 3.56689800

H 4.24431300 -0.70033200 3.52340900

C 2.51880900 -1.15681900 4.72232300

H 3.08165100 -1.39520600 5.62082400

C 1.11645500 -1.23601300 4.73426100

C 0.35338300 -1.62399000 5.88760700

H 0.88672500 -1.89326700 6.79493900

C -1.00046900 -1.65422600 5.84822200

H -1.57263900 -1.95075500 6.72258400

C -1.71579200 -1.28132400 4.65965700

C -3.11767100 -1.23616500 4.58475700

H -3.71510100 -1.52006700 5.44631300

C -3.71487400 -0.81560100 3.41663000

H -4.79345300 -0.75055000 3.32471400

C -2.92187600 -0.46634500 2.31369100

C -0.99168800 -0.91880200 3.50984600

C 0.44675800 -0.91113400 3.53894800

C -3.51519800 0.02367200 1.03995300

C -3.89838400 1.35896000 0.92190300

C -4.36289300 1.85754900 -0.29518700

C -4.44805400 0.99309800 -1.38862700

C -4.07031400 -0.34524300 -1.28837500

C -3.61088600 -0.82437100 -0.06133400

C -0.46110300 1.76471900 0.72320600

C 0.13178300 1.36157000 -0.34577600

H 0.32912000 -0.27591500 -0.55843100

Si -0.36299700 -2.47808100 0.11282000

H -1.36225200 -2.87788800 -0.93730800

H 0.92526200 -3.05447100 -0.40007000

C -0.78582000 -3.57485900 1.60955700

C 0.19890500 -3.92209500 2.54777000

C -2.11084400 -3.90978900 1.92799900

C -0.11909100 -4.55885800 3.74489200

H 1.24127800 -3.68084000 2.34303300

C -2.44160800 -4.54110500 3.12441200

H -2.90738700 -3.67922400 1.22319600

C -1.44507000 -4.86393200 4.04199100

H 0.66790800 -4.80943800 4.45161300

H -3.47968300 -4.78169400 3.33985000

H -1.69776000 -5.35437000 4.97817300

H 5.23213500 0.76907400 -1.96947400

H 2.71708400 -1.97347400 0.15560100

H 3.76143900 1.79628600 1.92325900

H -3.79263400 2.02895600 1.77106900

H -3.30091800 -1.86034700 0.01963800

H -4.79881700 1.37659200 -2.34373600

C -4.71135000 3.30047700 -0.44352200

C -5.88662000 3.80734500 0.13070200

C -3.85295200 4.15047600 -1.16401800

C -6.18879000 5.16105400 -0.02421200

C -4.18809200 5.49687100 -1.29038300

C -5.35293900 6.02154100 -0.73071700

H -7.10364400 5.55136800 0.41749700

H -3.52150300 6.15579900 -1.84409500

C -4.10100100 -1.24092800 -2.48119400

C -5.19646800 -2.08546800 -2.70757000

C -3.01487500 -1.23273600 -3.37110400

C -5.19164200 -2.91381100 -3.82975200

C -3.04653000 -2.07560700 -4.48011100

C -4.12436900 -2.92523900 -4.72531400

H -6.04368100 -3.56709700 -4.00827100

H -2.20523600 -2.07151600 -5.17062400

C 5.33760000 2.71447200 -0.06118600

C 6.72389300 2.67211700 0.16242100

C 4.69908200 3.94899600 -0.26906400

C 7.44871000 3.86416200 0.17384900

C 5.45892200 5.11748900 -0.24968300

C 6.83502800 5.09735800 -0.02795800

H 8.52210100 3.82591200 0.35001200

H 4.96178100 6.07157400 -0.41507700

C 4.17822300 -1.73721300 -2.10232200

C 5.35372600 -2.50631100 -2.09066000

C 3.20122000 -1.96589300 -3.08425000

C 5.53700800 -3.48516400 -3.06718700

C 3.42096000 -2.95409700 -4.04235600

C 4.58326500 -3.72250300 -4.05394200

H 6.44624800 -4.08322200 -3.04990600

H 2.65803600 -3.13218300 -4.79778900

C 6.40731700 -2.29894200 -1.02888500

H 6.89507200 -1.32327500 -1.13292900

H 5.97213900 -2.32855400 -0.02405300

H 7.18098400 -3.06971300 -1.09054600

C 1.93355600 -1.15319200 -3.12134800

H 1.40781600 -1.18078600 -2.16146000

H 2.14743000 -0.10137100 -3.34144600

H 1.24952400 -1.52828300 -3.88823900

C 4.80481600 -4.76983400 -5.11653900

H 5.19680800 -4.31903300 -6.03652200

H 5.52445400 -5.52599700 -4.78768100

H 3.87029100 -5.27910500 -5.37325800

C 3.22126600 4.02373100 -0.56304100

H 3.01462000 3.65202800 -1.57312400

H 2.62416900 3.41562400 0.12359300

H 2.86229000 5.05568000 -0.50702200

C 7.44264900 1.36227200 0.38269600

H 6.87467900 0.69250800 1.03568000

H 7.59406100 0.83152000 -0.56482500

H 8.42750900 1.52790400 0.82939300

C 7.62758500 6.37983400 0.01922300

H 7.27653700 7.09319600 -0.73368900

H 7.53070000 6.86441000 0.99844900

H 8.69250400 6.19855400 -0.15578400

C -6.82322700 2.90904900 0.90311400

H -7.04245100 1.98975700 0.34958700

H -6.38790400 2.60500000 1.86205300

H -7.76892900 3.41783200 1.11162400

C -2.58591600 3.62818400 -1.79915700

H -2.05348200 2.93420600 -1.14131700

H -2.80332000 3.08543600 -2.72739500

H -1.91031600 4.45126800 -2.05207800

C -5.69320600 7.48110700 -0.90209900

H -5.87206400 7.72134600 -1.95663700

H -6.59309400 7.75091900 -0.34104000

H -4.87511900 8.12295600 -0.55636800

C -1.82821900 -0.33578200 -3.12584200

H -2.11794000 0.72136400 -3.10381900

H -1.36279200 -0.55423600 -2.15828100

H -1.07095400 -0.46497000 -3.90527000

C -6.36697000 -2.09703400 -1.75458500

H -6.06136400 -2.41966900 -0.75257500

H -6.80339800 -1.09762500 -1.64535200

H -7.15058800 -2.77592600 -2.10331900

C -4.12043300 -3.85159900 -5.91580100

H -3.62487200 -3.39232200 -6.77733200

H -3.58561700 -4.78130100 -5.68643700

H -5.13831600 -4.12204200 -6.21417900

C 0.76426200 1.79116000 -1.62017300

H 1.82653300 1.52175400 -1.62753700

H 0.29395300 1.29799900 -2.47733600

H 0.67375800 2.87475300 -1.74735700

H -0.77589700 2.68527800 1.19597900

**^3^Ts-1bʹ**

Fe -0.34176000 -0.29772200 0.82088700

N 1.08910800 -0.69324000 2.49461000

N -1.56602000 -0.49384600 2.41113500

C 3.14269600 -0.37070000 1.25546700

C 3.25711400 -1.30704500 0.22948800

C 3.95012900 -0.99391200 -0.93980400

C 4.51383100 0.27710300 -1.07130900

C 4.41134200 1.22551200 -0.05289700

C 3.72714700 0.88660100 1.11600900

C 2.41921100 -0.71240100 2.51240100

C 3.13865200 -1.03019600 3.67929100

H 4.22267400 -1.03563400 3.64210400

C 2.46094500 -1.33908700 4.83934300

H 3.00036900 -1.59978200 5.74594200

C 1.05685800 -1.30912500 4.84493600

C 0.25588000 -1.61008900 5.99912700

H 0.75903400 -1.89311700 6.91939300

C -1.09658500 -1.54524100 5.94420800

H -1.69634300 -1.77877200 6.81919800

C -1.77374500 -1.15879100 4.73714100

C -3.16846200 -1.02942500 4.63803000

H -3.79545700 -1.25042600 5.49690800

C -3.72139700 -0.61146000 3.44634800

H -4.79266300 -0.48558000 3.33450400

C -2.89276800 -0.34714600 2.34660100

C -1.01316800 -0.87674000 3.58863500

C 0.42009900 -0.96573300 3.63703400

C -3.42933600 0.14745200 1.05036100

C -3.75124500 1.49627400 0.91634000

C -4.13035700 2.01752600 -0.32065900

C -4.21841800 1.15432400 -1.41412200

C -3.91696600 -0.20254300 -1.29583300

C -3.52013900 -0.69903000 -0.05362700

C -0.18195100 1.62930900 0.60768200

C 0.34190400 1.04200100 -0.41181600

H 0.37250900 -0.62178300 -0.48503700

Si -0.40941800 -2.62811200 0.29848600

H -1.37774400 -3.01797400 -0.78445000

H 0.86810500 -3.30210500 -0.11184700

C -0.97206800 -3.62217400 1.81920400

C -0.05071200 -4.00301900 2.80749200

C -2.32924000 -3.84789600 2.09531600

C -0.45922100 -4.57081500 4.01178500

H 1.01310800 -3.84448600 2.63558600

C -2.74954100 -4.41000300 3.29813900

H -3.07989000 -3.58558900 1.35220000

C -1.81396600 -4.77052400 4.26472900

H 0.28066200 -4.85114600 4.75704800

H -3.80931000 -4.57009800 3.47998600

H -2.13756500 -5.20909800 5.20481700

H 5.05456900 0.52790300 -1.98097300

H 2.80360800 -2.28703700 0.33753000

H 3.63929100 1.61854000 1.91497800

H -3.65650200 2.15993300 1.77205300

H -3.25977800 -1.74788800 0.03927800

H -4.50769300 1.55114300 -2.38404400

C -4.36863900 3.48103800 -0.47705000

C -5.49968200 4.08043000 0.10685300

C -3.44922900 4.26146400 -1.19567900

C -5.68926200 5.45188000 -0.03560100

C -3.67355100 5.63587500 -1.30817200

C -4.78435200 6.24914400 -0.73824400

H -6.56810700 5.91327600 0.41140800

H -2.95640000 6.24116900 -1.85932900

C -3.96717200 -1.09851500 -2.48826600

C -5.09129800 -1.90058400 -2.72348600

C -2.87463000 -1.12974800 -3.37162200

C -5.10972600 -2.72762200 -3.84788000

C -2.93055500 -1.96902600 -4.48101600

C -4.03912800 -2.77683600 -4.73621400

H -5.98445800 -3.34818100 -4.03238900

H -2.08398100 -1.99725600 -5.16474600

C 5.04589700 2.56912400 -0.20078900

C 6.40795400 2.72803100 0.09271100

C 4.28492400 3.66653800 -0.63726500

C 6.99117100 3.98774800 -0.05152400

C 4.90260000 4.90832600 -0.76785700

C 6.25535500 5.08987800 -0.47904500

H 8.04854300 4.10967200 0.17522800

H 4.31312000 5.75793100 -1.10823100

C 4.13120200 -2.01844100 -2.01161400

C 5.30210900 -2.79267500 -2.02820300

C 3.14265500 -2.21481400 -2.98775700

C 5.46474300 -3.75688600 -3.02221300

C 3.34348300 -3.18615400 -3.96746900

C 4.49682700 -3.96755500 -4.00204600

H 6.37017900 -4.36093200 -3.02881600

H 2.57350600 -3.34020800 -4.72121600

C 6.37197000 -2.59473700 -0.98130700

H 6.83224200 -1.60308900 -1.06369000

H 5.95748900 -2.66768600 0.03005000

H 7.16296900 -3.34358400 -1.08261400

C 1.86839400 -1.40940600 -2.97575300

H 1.21506900 -1.72405700 -2.15411500

H 2.06659000 -0.34091600 -2.83877500

H 1.31482200 -1.54054600 -3.91000500

C 4.69780700 -4.99825700 -5.08508000

H 5.08179200 -4.53514100 -6.00229900

H 5.41584200 -5.76476300 -4.77749700

H 3.75668000 -5.49687500 -5.33865500

C 2.82626500 3.49876900 -0.98250300

H 2.70355600 2.83669000 -1.84830000

H 2.26444700 3.04113800 -0.16231100

H 2.36395300 4.46047800 -1.22419400

C 7.23844000 1.55433300 0.55326600

H 6.79432800 1.07124300 1.43054900

H 7.30893200 0.78660700 -0.22579300

H 8.25426200 1.86791600 0.81104800

C 6.89369200 6.44913700 -0.62228700

H 6.69942200 6.87720400 -1.61193300

H 6.49717800 7.15225100 0.11980900

H 7.97798200 6.39665200 -0.48502800

C -6.50962300 3.25528600 0.86876200

H -6.79538200 2.35818900 0.30926000

H -6.10998000 2.91450800 1.83085600

H -7.41443000 3.83588500 1.07114100

C -2.23470300 3.65332700 -1.85858000

H -1.81537100 2.82191800 -1.28498700

H -2.48591800 3.26186700 -2.85251700

H -1.45090300 4.40551200 -1.99267100

C -5.01887600 7.73268700 -0.87693300

H -5.92894400 7.93592300 -1.45337800

H -5.14248900 8.20768400 0.10288500

H -4.18366000 8.22334300 -1.38564800

C -1.65193100 -0.28728200 -3.11120200

H -1.89560600 0.77928900 -3.05159100

H -1.19010700 -0.55543300 -2.15517700

H -0.90608300 -0.42232400 -3.90000500

C -6.26771200 -1.87374700 -1.77794600

H -5.99158100 -2.25391500 -0.78749700

H -6.64122700 -0.85377800 -1.63323400

H -7.08971400 -2.48825900 -2.15647500

C -4.06059200 -3.68561900 -5.93999200

H -3.83985800 -3.13239200 -6.85977400

H -3.30742000 -4.47687600 -5.84744700

H -5.03615900 -4.16598800 -6.06197300

C -0.46166200 2.95444000 1.21602400

H 0.08391600 3.77134700 0.72703300

H -1.53241800 3.17796200 1.13982700

H -0.21166800 2.95446400 2.28262000

H 0.82999000 1.19569300 -1.36259400

MECP

Fe -0.45049100 -0.20101700 -0.30808000

N 0.54564700 1.78283700 -0.42436100

N -2.00806000 1.08216300 0.06346900

C 2.70962300 1.01696700 -1.18142100

C 3.63910700 0.46914000 -0.28695000

C 4.45246800 -0.57339700 -0.72940700

H 5.15391600 -1.02189200 -0.02973200

C 4.37799700 -1.05534800 -2.03382900

C 3.46787300 -0.46607900 -2.91219000

H 3.40708700 -0.82553600 -3.93747100

C 2.62292400 0.56429000 -2.50706500

C 1.80318900 2.10310700 -0.71835400

C 2.27528400 3.42607200 -0.60596000

H 3.31110400 3.63308300 -0.85209600

C 1.42592400 4.42256200 -0.18682900

H 1.77395000 5.44727600 -0.08832200

C 0.08662700 4.10756100 0.10668100

C -0.87609400 5.08546000 0.52686400

H -0.55166900 6.11565100 0.64340900

C -2.16211400 4.73418200 0.77010900

H -2.88968400 5.47625000 1.08539800

C -2.59776400 3.37439000 0.61623200

C -3.92647700 2.96819700 0.83435100

H -4.67172400 3.69597700 1.14198300

C -4.26150200 1.64862200 0.64303400

H -5.27716900 1.29708100 0.79004500

C -3.28028500 0.72024200 0.25275300

C -1.67129700 2.38959800 0.22445700

C -0.30504100 2.76214300 -0.03582500

C -3.62762200 -0.70917400 0.03227600

C -3.85417800 -1.16061500 -1.27816500

C -4.12807200 -2.50969700 -1.47696200

H -4.29763200 -2.87111900 -2.48930500

C -4.18710100 -3.40936200 -0.41149300

C -4.00131000 -2.92368400 0.88018600

H -4.06318000 -3.60987600 1.72225600

C -3.73280200 -1.57716400 1.12664900

C -3.83391000 -0.19603900 -2.43731400

H -3.96612500 -0.72310400 -3.38700300

H -4.63864100 0.54424600 -2.35009400

H -2.88363400 0.34435300 -2.47692800

C -3.61537000 -1.07525700 2.54509800

H -2.85075300 -0.30159200 2.65245800

H -4.56826800 -0.64815300 2.88378500

H -3.35038200 -1.88881500 3.22531800

C 3.78517400 1.00108800 1.11602500

H 4.32831100 0.29258100 1.74613400

H 4.33477200 1.95119300 1.11894900

H 2.81490200 1.17817800 1.58707100

C 1.63993300 1.17496900 -3.47400500

H 1.74545400 0.72957200 -4.46745100

H 0.61293200 1.00429900 -3.13427400

H 1.78907600 2.25701500 -3.56977500

C 5.24273500 -2.20716200 -2.47948400

H 5.45234400 -2.15659800 -3.55262500

H 6.19781900 -2.21875100 -1.94515300

H 4.74561000 -3.16588800 -2.28562700

C -4.43105200 -4.87631400 -0.65867100

H -5.08303600 -5.03253200 -1.52408800

H -3.48665000 -5.39657500 -0.85865000

H -4.89450000 -5.35592600 0.20895200

C -0.62863200 -1.00222200 -2.06779900

C 0.39192600 -1.74398700 -1.63889200

H -1.20093700 -1.26775800 -2.95724700

C 0.98456300 -3.04332200 -2.10954500

H 0.46132000 -3.39994000 -3.00155600

H 2.04729900 -2.91985400 -2.34995900

H 0.90940100 -3.81364300 -1.33228200

H 0.96072200 -1.36799400 -0.72864300

Si 0.03746300 -0.67804000 2.04441300

H -0.23051200 0.42577500 3.04629300

H -0.84392300 -1.78291800 2.57183400

C 1.77669900 -1.26892600 2.56848600

C 2.37776500 -0.80891300 3.75012700

C 2.51293400 -2.17593700 1.79311500

C 3.64863900 -1.22675400 4.13788200

H 1.83832400 -0.10449500 4.37922200

C 3.78102400 -2.60880800 2.17489600

H 2.09180900 -2.55547100 0.86362200

C 4.35792700 -2.12991500 3.34912100

H 4.08705600 -0.84750900 5.05758400

H 4.32308800 -3.31492000 1.55073400

H 5.34903000 -2.46074200 3.64867600

**M-^1^Int-1**

Fe 0.07182700 -1.18546900 -0.74655200

N -1.34988600 -1.72556500 -2.03451300

N 1.22563300 -2.07216900 -2.08295000

C -3.25226600 -0.48033300 -1.11757100

C -4.44473700 -0.67880700 -0.41557100

C -5.00084200 0.35366000 0.34169600

C -4.33262200 1.58003700 0.42116900

C -3.13627600 1.79318000 -0.26370700

C -2.63494100 0.76358900 -1.05831400

C -2.67905500 -1.51672100 -2.00775200

C -3.54753700 -2.18924700 -2.89175300

H -4.61004000 -1.98372500 -2.82135000

C -3.06359700 -3.04637400 -3.85191400

H -3.73391200 -3.55201400 -4.54094100

C -1.67496700 -3.22704700 -3.95029000

C -1.03997400 -4.04028100 -4.95177300

H -1.67152600 -4.55597500 -5.66979200

C 0.31064500 -4.15789500 -5.01530800

H 0.77695000 -4.76635100 -5.78498100

C 1.15242900 -3.48749100 -4.06316500

C 2.55592900 -3.55083500 -4.05439200

H 3.08085100 -4.12716100 -4.81087800

C 3.24986000 -2.87672400 -3.06923600

H 4.33339500 -2.90690400 -3.02702900

C 2.56074800 -2.15317900 -2.08287300

C 0.54169400 -2.71527000 -3.05962500

C -0.87408300 -2.55426200 -3.01162400

C 3.27200200 -1.45307200 -0.97893600

C 3.82105200 -0.19137400 -1.19067700

C 4.36246400 0.53672600 -0.13033900

C 4.41731500 -0.05816800 1.13005600

C 3.90569300 -1.33695700 1.35306200

C 3.31306300 -2.02258600 0.29289300

C 0.65801500 0.63406500 -1.02635300

C 0.10940000 0.51866900 0.13326800

C -0.16744900 -2.83191200 0.35213000

C -0.82137100 -1.88993300 0.89189400

H -1.72387300 0.92265400 -1.61545300

H -4.75854200 2.37452900 1.02872700

H -4.94388400 -1.64446400 -0.44235700

H 4.84453700 0.49361800 1.96369800

H 2.85530800 -2.99166000 0.46815500

H 3.75830200 0.26460800 -2.17431800

C -2.30769900 3.02801300 -0.13681700

C -1.98067800 3.78140100 -1.27930500

C -1.72365500 3.35147400 1.10044900

C -1.03969600 4.80380800 -1.17592600

C -0.77919400 4.37719800 1.15822900

C -0.40369300 5.09821100 0.02859300

H -0.78298300 5.37478900 -2.06678500

H -0.30685800 4.60153400 2.11276600

C -6.30730800 0.16779800 1.04183000

C -7.50144600 0.40391900 0.34081400

C -6.34704000 -0.21604600 2.39063400

C -8.71864800 0.25029800 1.00185500

C -7.58501200 -0.35756500 3.01829300

C -8.78136300 -0.13132900 2.34146300

H -9.64329200 0.43920800 0.45948400

H -7.61415700 -0.65062900 4.06612200

C 4.78913800 1.95218200 -0.33103600

C 5.92248900 2.25222000 -1.10859500

C 4.03448000 2.99068100 0.24182300

C 6.27518500 3.58374500 -1.31140000

C 4.42011200 4.31311100 0.00711100

C 5.53062900 4.63141500 -0.76765000

H 7.15673000 3.81221600 -1.90810500

H 3.83157400 5.11520800 0.44920500

C 3.96778300 -1.94131500 2.71563200

C 5.06219300 -2.74396500 3.07520200

C 2.93843300 -1.69124000 3.63369200

C 5.10875900 -3.28930400 4.35591200

C 3.02107900 -2.25626900 4.90757500

C 4.09514200 -3.05759500 5.28641200

H 5.95750000 -3.91049500 4.63723500

H 2.22289700 -2.06337500 5.62184500

C -2.61394200 3.50164300 -2.62320000

H -2.07362800 2.71893400 -3.16916700

H -3.64976000 3.16543600 -2.52069700

H -2.60390900 4.40152500 -3.24607500

C 0.68185700 6.14282800 0.09165900

H 1.59769900 5.77441900 -0.38761900

H 0.38820100 7.06133100 -0.42802000

H 0.92760900 6.40155300 1.12646100

C -2.05902200 2.59586700 2.36515800

H -3.00922000 2.93544300 2.79509100

H -2.15421000 1.52155300 2.18351300

H -1.28336700 2.74698200 3.12222700

C -7.47155300 0.83128400 -1.10693900

H -6.91088200 1.76407900 -1.23447900

H -6.97891100 0.08098200 -1.73567300

H -8.48355200 0.98571800 -1.49248500

C -5.07476000 -0.49095800 3.15363000

H -4.55967900 -1.37139400 2.75349600

H -4.36909300 0.34406200 3.08335700

H -5.28234500 -0.67376400 4.21195000

C -10.11087900 -0.31503700 3.02990400

H -10.51223400 -1.31979300 2.84961200

H -10.01940600 -0.18658600 4.11296400

H -10.85161900 0.40373300 2.66458100

C 6.76458300 1.15157500 -1.70959100

H 6.98442500 0.37136000 -0.97337300

H 6.25427100 0.66308000 -2.54770000

H 7.71269500 1.54856000 -2.08455200

C 2.82356500 2.72097600 1.10518600

H 2.29020400 1.81661800 0.80170100

H 3.11338800 2.59149500 2.15590000

H 2.11694600 3.55606100 1.05731400

C 5.91710500 6.06552200 -1.03083900

H 6.98020800 6.23536100 -0.82775200

H 5.73913800 6.33349500 -2.07914100

H 5.33912300 6.75483400 -0.40789400

C 1.76136400 -0.83182700 3.24589800

H 2.07395600 0.18970400 2.99899700

H 1.25946800 -1.22936600 2.35692600

H 1.03130500 -0.77864200 4.05942100

C 4.16208500 -3.67842800 6.65980000

H 3.98413400 -4.75944800 6.61120800

H 5.14716500 -3.53087200 7.11606100

H 3.41174500 -3.24873100 7.33055300

C 6.17423600 -3.00715600 2.08900600

H 5.79487800 -3.49205800 1.18250600

H 6.65187900 -2.07369900 1.77000300

H 6.94275400 -3.65257400 2.52448900

H -0.18918200 1.06301200 1.01820700

C 1.22165900 1.61113800 -1.99469900

H 1.59317000 1.13346800 -2.90808700

H 0.44017700 2.32848700 -2.28036700

H 2.03943300 2.19674400 -1.55651600

H 0.11061200 -3.87522800 0.37108900

C -1.65895600 -1.34699600 1.98159400

H -2.01837600 -2.13406500 2.65632300

H -1.08591800 -0.62686900 2.57864600

H -2.52356700 -0.81059400 1.57623800

**M-^1^Int-1ʹ**

Fe 0.01434800 -1.93157700 0.14533200

N -1.13662900 -2.79996500 1.35426100

N 1.43127200 -2.40881300 1.37651200

C -3.19881600 -1.70557500 0.84556900

C -3.25767200 -0.53868600 1.60298000

C -3.73037400 0.64937900 1.04766100

C -4.18764300 0.62911800 -0.27194500

C -4.17589700 -0.53888100 -1.03561900

C -3.66620000 -1.70762200 -0.46524800

C -2.48493600 -2.88104500 1.40687100

C -3.11331900 -3.99219900 1.95579900

H -4.19722400 -4.01554700 1.98669300

C -2.36294900 -5.06116900 2.45302800

H -2.85838700 -5.93469000 2.86695700

C -0.97090700 -4.98790000 2.43551400

C -0.06836700 -5.99162500 2.94586000

H -0.48976600 -6.91046600 3.34417700

C 1.27415300 -5.79618900 2.95031200

H 1.93662300 -6.55870000 3.35088100

C 1.86473900 -4.57824800 2.44920900

C 3.22164800 -4.26253200 2.49741200

H 3.93149200 -4.95887900 2.93473700

C 3.65317400 -3.03617200 2.00134600

H 4.69969200 -2.76231100 2.07423500

C 2.75722600 -2.13448100 1.42203500

C 1.00425700 -3.59993600 1.90521300

C -0.40057500 -3.81220000 1.89855600

C 3.22082000 -0.86099100 0.82873400

C 4.46946500 -0.78977200 0.20124400

C 4.91375700 0.39393200 -0.38376700

C 4.08941400 1.51985100 -0.34086800

C 2.83790100 1.47123400 0.27203700

C 2.41241700 0.27858300 0.85805700

C -0.41103300 -0.40891400 -0.73097600

C -0.40965300 -1.40772600 -1.58818100

H -0.60561700 0.65748000 -0.65034100

C -0.70794400 -1.70281400 -3.01811400

H -0.37126000 -0.89739000 -3.68348200

H -0.25392100 -2.64087500 -3.35319500

H -1.79450800 -1.79717100 -3.15068800

H -3.58548200 -2.61024300 -1.06462500

H -4.55181400 1.54957500 -0.72021600

H -2.88672900 -0.54489000 2.62409900

H 5.09667700 -1.67448000 0.12974100

H 1.43577400 0.22969400 1.32853300

H 4.42549500 2.44479700 -0.80332500

C -4.66661900 -0.53467400 -2.44520000

C -4.00438000 0.21842100 -3.42987300

C -5.80361700 -1.29135000 -2.78899700

C -4.47438300 0.17683300 -4.74454800

C -6.24425500 -1.29593000 -4.10985800

C -5.58858200 -0.57302700 -5.10630600

H -3.94904400 0.74950900 -5.50650100

H -7.12685100 -1.87808200 -4.36989100

C -3.70106700 1.92306000 1.82405100

C -4.90082800 2.59272500 2.12567800

C -2.47052300 2.47176600 2.23245000

C -4.85110600 3.81027600 2.80364100

C -2.46808800 3.69325700 2.90732500

C -3.64313800 4.38297800 3.19504600

H -5.78318300 4.32305700 3.03386100

H -1.51169100 4.12119500 3.20353800

C 6.23787800 0.45389300 -1.06953800

C 6.32096900 0.19415300 -2.44782500

C 7.39017300 0.77577300 -0.33971100

C 7.56385900 0.26090100 -3.07295500

C 8.61761600 0.83296300 -1.00168200

C 8.72456700 0.57949800 -2.36653200

H 7.63077700 0.06007100 -4.14079600

H 9.51233800 1.08383800 -0.43510600

C 1.94767700 2.66894400 0.28584700

C 1.84652400 3.45146000 1.44587700

C 1.19240200 2.99269500 -0.85356100

C 0.96496000 4.53113700 1.46150900

C 0.30310700 4.06688400 -0.78968700

C 0.16409200 4.83921200 0.36314900

H 0.88399100 5.13385700 2.36455800

H -0.30223200 4.30191600 -1.66318900

C -2.80170200 1.07377000 -3.10656400

H -3.10175600 2.03766400 -2.67778900

H -2.14150700 0.59109600 -2.38271000

H -2.22439000 1.28166200 -4.01296900

C -6.08974000 -0.59818400 -6.52872600

H -6.19470400 -1.62548400 -6.89523700

H -7.07425100 -0.12230700 -6.60887500

H -5.40837400 -0.06858600 -7.20128700

C -6.56572500 -2.08085600 -1.74986200

H -6.03895700 -3.00068200 -1.47129900

H -6.70561700 -1.50386000 -0.83018100

H -7.55107500 -2.36674800 -2.13000600

C 5.08245400 -0.15439300 -3.23737300

H 4.59579300 -1.05143700 -2.83879200

H 4.33890900 0.64931800 -3.19010200

H 5.32304600 -0.33443000 -4.28909600

C 10.05504100 0.64910500 -3.07432700

H 10.29007200 -0.30038100 -3.56857000

H 10.05083900 1.42597000 -3.84741900

H 10.86699500 0.87531800 -2.37671400

C 7.30729800 1.06013900 1.14049200

H 6.64115900 1.90566100 1.34661100

H 6.90311200 0.20198300 1.68905100

H 8.29297500 1.29389100 1.55310500

C -6.24418000 2.01787400 1.73886500

H -6.46166000 2.17348300 0.67600300

H -6.28724400 0.93901200 1.91795500

H -7.04632000 2.49333200 2.31141400

C -1.13879300 1.83710100 1.91116600

H -1.17565200 0.75243700 1.80297600

H -0.75326900 2.24055400 0.96889200

H -0.40406700 2.08291500 2.68324200

C -3.60180900 5.69665200 3.93508900

H -3.41130300 5.54124100 5.00383700

H -2.80418300 6.34253700 3.55171900

H -4.54895300 6.23691100 3.84218500

C 1.34033200 2.20730800 -2.13523200

H 1.39622400 1.13071500 -1.94903200

H 0.49735700 2.39421200 -2.80770100

H 2.25881300 2.49473400 -2.66240500

C 2.65096100 3.11105900 2.67633700

H 2.29456800 2.18050700 3.13412400

H 3.70886400 2.95940300 2.43716800

H 2.57736000 3.90443000 3.42601100

C -0.86092700 5.94175300 0.44211100

H -1.81604300 5.54499600 0.80919700

H -0.54473400 6.73168500 1.13135700

H -1.04152800 6.39585600 -0.53735500

**M-^1^Int-1ʹʹ**

Fe 0.25680800 -0.67745000 -0.77778700

N -1.11634000 -1.77212300 -1.75672100

N 1.47452000 -1.82211100 -1.77032200

C -3.16267000 -0.67421700 -0.97445700

C -4.22003600 -1.06012000 -0.14799800

C -4.93057700 -0.12111800 0.59534100

C -4.57262300 1.22064300 0.48893300

C -3.50394800 1.63057800 -0.31238300

C -2.80718100 0.67376800 -1.04903700

C -2.45651900 -1.73086300 -1.73170500

C -3.23369700 -2.71273300 -2.38810700

H -4.31277300 -2.61269700 -2.36361400

C -2.64325000 -3.76463000 -3.04514100

H -3.24502100 -4.52256300 -3.53871700

C -1.24083300 -3.84601500 -3.06863000

C -0.50424900 -4.91918600 -3.67767400

H -1.06278500 -5.71388600 -4.16411400

C 0.85247700 -4.96236700 -3.63090300

H 1.39292900 -5.79280500 -4.07547600

C 1.59829200 -3.91941100 -2.98641700

C 3.00037100 -3.88927500 -2.86311200

H 3.59869000 -4.69458600 -3.27822300

C 3.59815400 -2.83155700 -2.20865500

H 4.67654800 -2.77569500 -2.10417500

C 2.80831500 -1.80182700 -1.67492100

C 0.88690100 -2.85295300 -2.41932500

C -0.53640500 -2.81368900 -2.43200300

C 3.33444200 -0.63057900 -0.95062100

C 2.78606500 0.62778700 -1.22561400

C 3.21203700 1.76433600 -0.53730500

C 4.17112400 1.61772300 0.46453900

C 4.72003100 0.36776900 0.76532900

C 4.30694200 -0.75073100 0.04124200

C 0.68407000 -3.66685000 0.78490300

C 2.04749800 -3.98754000 0.86693400

C -0.17098700 -4.65244100 0.26825300

C 2.53731700 -5.22281500 0.45098100

H 2.74617700 -3.24797700 1.25763100

C 0.30709300 -5.89058700 -0.15537200

H -1.23684400 -4.44223200 0.18531900

C 1.66701400 -6.17879300 -0.06889300

H 3.60001500 -5.43988400 0.52904100

H -0.38161500 -6.63018900 -0.55640200

H 2.04605500 -7.14222100 -0.39981300

Si 0.06025000 -1.88749500 1.07017800

H -1.35370300 -2.09367300 1.54128500

H 0.82425800 -1.39434400 2.26977100

H -0.57988800 0.13701400 0.27053800

H 4.72107100 -1.72654000 0.28151200

H 2.07785400 0.74176800 -2.04529900

H 4.50076300 2.49187100 1.02027000

H -4.46810000 -2.11272300 -0.03985600

H -1.97461600 0.98089600 -1.67330100

H -5.12046300 1.96237600 1.06489400

C 5.72188200 0.21974600 1.86059100

C 7.06798900 0.54676700 1.62853800

C 5.30980300 -0.25321600 3.11490900

C 7.98832200 0.38663800 2.66093800

C 6.26358900 -0.39591200 4.12479600

C 7.60461900 -0.08534700 3.91719100

H 9.03339700 0.63316600 2.48198000

H 5.94595800 -0.76108700 5.09940200

C 2.66144800 3.09372300 -0.92880100

C 3.19220200 3.74944900 -2.04967600

C 1.58584000 3.65070600 -0.21953300

C 2.61771000 4.95152600 -2.46429800

C 1.03830300 4.84977200 -0.66914600

C 1.53137600 5.50863700 -1.79434600

H 3.02460600 5.45769100 -3.33764500

H 0.18611300 5.26512700 -0.13862400

C -6.01256400 -0.55563200 1.52571700

C -7.34615500 -0.59462300 1.09359800

C -5.67818400 -0.93275900 2.83636500

C -8.33371600 -1.01232900 1.98487300

C -6.69482600 -1.34297500 3.69820400

C -8.02788200 -1.38655800 3.29235400

H -9.36856200 -1.04820300 1.64899400

H -6.43865300 -1.63727500 4.71424000

C -3.10313100 3.06796400 -0.30341200

C -3.28799000 3.87795700 -1.43278400

C -2.57631400 3.61766300 0.88084200

C -2.96103400 5.23404200 -1.35835000

C -2.27279500 4.97652200 0.91673300

C -2.46369800 5.80435500 -0.19066300

H -3.11608600 5.86194900 -2.23378300

H -1.86852100 5.40248800 1.83383800

C 7.51455100 1.06002300 0.28039000

H 7.20980800 0.38257300 -0.52504200

H 7.06881300 2.03643400 0.05777600

H 8.60207000 1.17051300 0.24212200

C 8.62752300 -0.25276100 5.01340000

H 9.40073200 -0.97357100 4.72378100

H 9.13143800 0.69557500 5.23189300

H 8.16667400 -0.61001900 5.93904500

C 3.86508900 -0.60446500 3.37703800

H 3.18658100 0.18874700 3.04628700

H 3.56366900 -1.51003500 2.83799800

H 3.69152900 -0.78088200 4.44221200

C 4.36095400 3.16471400 -2.80679200

H 5.19054600 2.91902800 -2.13441300

H 4.08500100 2.23511100 -3.31831200

H 4.72881000 3.86673900 -3.56077300

C 0.88776000 6.78930300 -2.26343000

H 1.33156400 7.14483400 -3.19833700

H -0.18617400 6.64374000 -2.42811100

H 0.99681300 7.58482600 -1.51675300

C 1.00415800 2.95493400 0.98388900

H 0.47681000 2.03545200 0.69673900

H 1.77966400 2.66741600 1.70184700

H 0.28993900 3.60585300 1.49321800

C -9.11498100 -1.80323500 4.25157700

H -9.50717900 -0.93725900 4.79880000

H -9.95613300 -2.26686100 3.72616700

H -8.74204400 -2.51795800 4.99213400

C -4.24326000 -0.89902200 3.30399500

H -3.60670200 -1.56432600 2.70969800

H -3.81411500 0.10416500 3.20285100

H -4.16394600 -1.19923500 4.35272200

C -7.70741700 -0.19492700 -0.31661700

H -7.47297800 0.85920200 -0.50435700

H -7.14373900 -0.77888300 -1.05285200

H -8.77441800 -0.34383800 -0.50625300

C -2.32957700 2.75929100 2.09961500

H -3.26579700 2.50559600 2.60977600

H -1.83835000 1.81772500 1.83004500

H -1.69566300 3.28450400 2.82020400

C -2.12445000 7.27198400 -0.11394600

H -2.35831100 7.78640800 -1.05099800

H -2.68092000 7.76537900 0.69093000

H -1.05727100 7.42213700 0.08938300

C -3.85266700 3.31140500 -2.71344000

H -3.12449100 2.67297600 -3.22669200

H -4.73683100 2.69481700 -2.52057600

H -4.13582200 4.11162000 -3.40358200

**M-^3^Int1**

Fe 0.07111900 -1.22608900 -0.42637600

N -1.53842000 -1.68585500 -2.11482500

N 1.13442800 -2.06083400 -2.03768000

C -3.40662500 -0.49436400 -1.17095900

C -4.62080000 -0.64759300 -0.49688000

C -5.11479500 0.37633300 0.31295200

C -4.37310800 1.55354300 0.45426900

C -3.15258500 1.71967500 -0.20080600

C -2.69445600 0.69167400 -1.02182000

C -2.85948600 -1.53819800 -2.07006800

C -3.71620800 -2.29668200 -2.89835100

H -4.78830600 -2.14339800 -2.83985600

C -3.17977800 -3.17703500 -3.80640200

H -3.82121300 -3.74857800 -4.47189800

C -1.78189200 -3.31961900 -3.89142300

C -1.13349600 -4.16703900 -4.85156900

H -1.75037300 -4.73911600 -5.53903200

C 0.21865900 -4.23839400 -4.91261500

H 0.70791100 -4.86496400 -5.65307800

C 1.03937700 -3.50546400 -3.98848900

C 2.44502100 -3.55914200 -4.00393400

H 2.95586500 -4.15673600 -4.75392100

C 3.15295400 -2.85204800 -3.05993100

H 4.23729600 -2.86518300 -3.03950400

C 2.46552900 -2.12235300 -2.07276100

C 0.43109200 -2.70541700 -3.00128000

C -1.00669100 -2.56180800 -2.99081300

C 3.21552400 -1.39692700 -1.01046900

C 3.73147200 -0.13180700 -1.27226300

C 4.35605400 0.60596800 -0.26533600

C 4.51118300 0.02146800 0.99155900

C 4.02551200 -1.25919100 1.26349700

C 3.36186600 -1.95911300 0.25648200

C 0.59601800 0.66586400 -0.77461700

C 0.33783700 0.48392600 0.45933300

C -0.26044800 -2.93943000 0.45028700

C -0.82035300 -1.98917600 1.10451000

H -1.75346800 0.80570500 -1.54222400

H -4.75793300 2.34741400 1.08976700

H -5.18467900 -1.57380900 -0.57814800

H 5.00570700 0.57880700 1.78334700

H 2.93045000 -2.93253200 0.47032200

H 3.58937000 0.31582200 -2.25191600

C -2.28311000 2.92089700 -0.03100700

C -2.00814700 3.75327200 -1.13051200

C -1.64679200 3.15409500 1.19838900

C -1.06875600 4.77322600 -0.99343300

C -0.70368300 4.17807500 1.29111600

C -0.38515500 4.98514600 0.20277000

H -0.85153300 5.40932900 -1.84983300

H -0.19286900 4.33685000 2.23897100

C -6.42871000 0.22835200 1.00790900

C -7.61184000 0.55505300 0.32592800

C -6.48214800 -0.21700700 2.33761200

C -8.83323100 0.42575300 0.98534300

C -7.72318100 -0.33230500 2.96372000

C -8.91001400 -0.01928800 2.30414900

H -9.74925900 0.68411200 0.45716800

H -7.76306000 -0.67534900 3.99594500

C 4.78872400 2.00951200 -0.52900800

C 5.84632500 2.26867000 -1.42037500

C 4.11747600 3.07607400 0.09274400

C 6.20486800 3.58783900 -1.68520200

C 4.50584800 4.38465900 -0.20567500

C 5.54072600 4.66245300 -1.09265600

H 7.02521400 3.78478500 -2.37340700

H 3.98127700 5.20869400 0.27489800

C 4.20013300 -1.85932400 2.61918100

C 5.36024000 -2.59338500 2.91388600

C 3.20802000 -1.68136800 3.59398900

C 5.50892500 -3.14387400 4.18489700

C 3.39346700 -2.24846600 4.85573900

C 4.53342100 -2.98414500 5.16944600

H 6.40939500 -3.71135300 4.41403900

H 2.62351600 -2.11125800 5.61260900

C -2.69726400 3.55116700 -2.46017000

H -2.26043500 2.71383800 -3.01709100

H -3.76121400 3.32853200 -2.33207200

H -2.60528400 4.44640700 -3.08241800

C 0.68594400 6.04249300 0.29741800

H 1.58327500 5.73415200 -0.25400900

H 0.35007500 6.99276900 -0.13189400

H 0.97826700 6.22417100 1.33649000

C -1.94089200 2.30333000 2.40967400

H -2.90094400 2.57221000 2.86663700

H -1.99116200 1.24314900 2.14764200

H -1.16592200 2.43120100 3.17214500

C -7.56603800 1.05358700 -1.09838300

H -6.96167600 1.96394900 -1.18129200

H -7.11264300 0.31395300 -1.76784200

H -8.57090900 1.27593900 -1.46902600

C -5.22085000 -0.58077400 3.08161800

H -4.73956100 -1.45869800 2.63583100

H -4.48420100 0.22957700 3.05201800

H -5.43394200 -0.80901000 4.12996300

C -10.24356400 -0.17954500 2.99060400

H -10.65845100 -1.17983500 2.81594100

H -10.15216600 -0.04654100 4.07324400

H -10.97339500 0.54744600 2.62004300

C 6.60312900 1.13961600 -2.08019400

H 6.87824800 0.36717300 -1.35452400

H 6.00408700 0.64806100 -2.85524100

H 7.51814100 1.51004000 -2.55187100

C 2.99374900 2.84669000 1.07593700

H 2.36863100 1.99604800 0.79288000

H 3.38582700 2.64412500 2.08064500

H 2.35026700 3.72937800 1.14300300

C 5.93660600 6.08162100 -1.41633500

H 7.00288600 6.24956700 -1.22739700

H 5.75369300 6.30992600 -2.47291600

H 5.37069100 6.80015100 -0.81582500

C 1.96045200 -0.89476200 3.28150700

H 2.20261500 0.12331900 2.95506200

H 1.39367800 -1.35679300 2.46528100

H 1.30908400 -0.82887300 4.15869100

C 4.70787300 -3.61327900 6.52946800

H 4.56084700 -4.69901800 6.48080600

H 5.71456400 -3.43819300 6.92441300

H 3.98767300 -3.21391300 7.25022500

C 6.43212100 -2.78352000 1.86804500

H 6.02879200 -3.26445500 0.96982800

H 6.85266300 -1.82309100 1.54848500

H 7.24912300 -3.40284100 2.24981600

H 0.28152900 0.90940500 1.45051200

C 0.97172000 1.60752500 -1.85785400

H 1.23802500 1.08591300 -2.78389200

H 0.13149500 2.27848500 -2.07754000

H 1.81572200 2.24511400 -1.56453800

H -0.13655700 -4.01443900 0.40920600

C -1.63495800 -1.54645100 2.26012500

H -1.97108300 -2.38236900 2.88710700

H -1.06067600 -0.85648200 2.89058400

H -2.51700600 -0.99911100 1.90688100

**M-^3^Int-1ʹ**

Fe -0.05082500 -1.48514500 -0.24463500

N 1.39914600 -2.31024700 -1.42455700

N -1.26633600 -2.54177900 -1.49588000

C 3.30108000 -0.96241000 -0.73338300

C 2.64120900 0.27078600 -0.74325500

C 3.17334300 1.37958600 -0.08310700

C 4.38723500 1.24288000 0.59402300

C 5.06611500 0.02322800 0.62034700

C 4.51094100 -1.07189000 -0.04081900

C 2.74523300 -2.13123000 -1.45788200

C 3.58190900 -2.98788100 -2.15599200

H 4.64701000 -2.78698800 -2.18244200

C 3.05204100 -4.09028100 -2.84679700

H 3.70659400 -4.76886500 -3.38598000

C 1.67930700 -4.29001100 -2.85571400

C 1.02818800 -5.38137100 -3.54071000

H 1.65277700 -6.09611800 -4.07013200

C -0.31835200 -5.52487100 -3.53041500

H -0.78815500 -6.35782100 -4.04680700

C -1.16376000 -4.58050400 -2.84430800

C -2.54819100 -4.65950000 -2.80008400

H -3.05982500 -5.47765800 -3.29967400

C -3.28280200 -3.66742300 -2.13068400

H -4.36685300 -3.68594800 -2.11814900

C -2.61297400 -2.62421800 -1.50998100

C -0.53382000 -3.49478000 -2.17151400

C 0.86431800 -3.36002800 -2.15426400

C -3.31976300 -1.49410300 -0.85550600

C -4.16480500 -1.68464700 0.23566500

C -4.75471900 -0.58795700 0.86905700

C -4.48795700 0.69619400 0.39073800

C -3.68049400 0.90756100 -0.73196200

C -3.09954100 -0.20420700 -1.34087700

C -0.66562900 -0.70594000 1.40244800

C 0.53338900 -1.12983300 1.56705800

H -1.53460300 -0.24865100 1.85361500

C 1.74955600 -1.27463400 2.40091200

H 1.54186500 -1.09080400 3.46141500

H 2.18602600 -2.27219700 2.29113800

H 2.50642000 -0.55640100 2.06840800

H -2.47888300 -0.07397100 -2.22195500

H -4.93757000 1.54822800 0.89382600

H -4.35037700 -2.68775800 0.61084500

H 1.71297300 0.38195200 -1.29925200

H 4.81771900 2.10466100 1.09836700

H 5.02488900 -2.02841300 -0.00080000

C -3.47066900 2.27614600 -1.29149500

C -4.57405800 3.03951200 -1.72126000

C -2.17212600 2.80355400 -1.41531300

C -4.35950100 4.31531000 -2.24109100

C -2.00316000 4.08482300 -1.94123300

C -3.08202200 4.85947200 -2.35637800

H -5.21720600 4.89762200 -2.57368400

H -0.99379600 4.48119700 -2.02809700

C -5.69092800 -0.79019400 2.01571700

C -7.07360300 -0.82875200 1.77372700

C -5.20032900 -0.94212900 3.32158300

C -7.94498800 -1.02213200 2.84490200

C -6.10358000 -1.13331300 4.36667400

C -7.47909200 -1.17961400 4.14883500

H -9.01638200 -1.05218700 2.65562600

H -5.72075000 -1.24811800 5.37907700

C 6.38109900 -0.09799100 1.31837200

C 7.56300700 0.17718000 0.61181300

C 6.43839000 -0.47472700 2.66838000

C 8.78702600 0.06553400 1.26915400

C 7.68201000 -0.57424500 3.29182600

C 8.86771500 -0.31171900 2.60893500

H 9.70278800 0.28098200 0.72176500

H 7.72496500 -0.86300400 4.34033100

C 2.48596900 2.70458700 -0.14064500

C 2.55393300 3.46779200 -1.31603100

C 1.76552500 3.18281300 0.96844100

C 1.88323300 4.69177100 -1.37210000

C 1.11499700 4.41063900 0.87414400

C 1.15286600 5.17730700 -0.29060400

H 1.93279700 5.27744700 -2.28807100

H 0.54361900 4.77104100 1.72725100

C -5.98975400 2.51103800 -1.66245000

H -6.03646100 1.44512400 -1.90487600

H -6.43038100 2.63581100 -0.66600900

H -6.62795900 3.05080500 -2.36855700

C -2.87526000 6.23197300 -2.94635800

H -2.97225600 6.20829800 -4.03860100

H -3.61593400 6.94527600 -2.56908600

H -1.87947100 6.62042600 -2.71064700

C -0.94519200 2.03412700 -0.99366100

H -1.07006000 1.55927600 -0.01666700

H -0.70486600 1.23382900 -1.70449000

H -0.08073500 2.69689700 -0.94488400

C -3.71950800 -0.87890300 3.60243600

H -3.16242900 -1.61494100 3.01302900

H -3.31472500 0.10806900 3.34768100

H -3.51159400 -1.06314600 4.66030900

C -7.61756200 -0.65996800 0.37560100

H -7.40853300 0.34209700 -0.01598900

H -7.15862400 -1.37087500 -0.32031700

H -8.70069300 -0.81152900 0.35492400

C -8.43638700 -1.42074000 5.28935800

H -9.40836500 -0.95397800 5.10076200

H -8.60929600 -2.49386900 5.43615500

H -8.04429500 -1.02006600 6.22966500

C 3.33330700 2.98239100 -2.51454000

H 4.35051000 2.68605100 -2.23599100

H 2.86267700 2.10470000 -2.97146800

H 3.40045200 3.76260100 -3.27809200

C 1.63098600 2.35821700 2.22352000

H 1.05530800 1.44801300 2.02029000

H 2.60352100 2.04656300 2.61856600

H 1.11112100 2.91905500 3.00565100

C 0.39776200 6.48001300 -0.37174000

H 0.70721000 7.17100400 0.42027900

H 0.56005100 6.97655400 -1.33369600

H -0.67960200 6.30997900 -0.25845800

C 5.17667500 -0.77331800 3.43986500

H 4.48173600 0.07371800 3.41570100

H 4.64414300 -1.63048500 3.01285000

H 5.39886400 -0.99952100 4.48668600

C 7.51214200 0.59709000 -0.83736900

H 6.97988000 -0.13980800 -1.44879600

H 6.97950300 1.54771500 -0.95662700

H 8.51869400 0.71918200 -1.24773200

C 10.20467600 -0.45676900 3.29196300

H 10.62694000 -1.45459200 3.12120700

H 10.92755200 0.27362800 2.91434500

H 10.11596300 -0.31791200 4.37398400

**M-^3^Int-1ʹʹ**

Fe 0.12678100 -1.48297800 -0.40253700

N -1.11411900 -2.62815100 -1.41881500

N 1.56918500 -2.37961100 -1.62816300

C -3.19968900 -1.75006000 -0.50894900

C -2.95818800 -1.81081400 0.86580100

C -3.71716300 -1.05901800 1.76606400

C -4.70237000 -0.20898600 1.25918400

C -4.92837000 -0.09312700 -0.11268800

C -4.17872700 -0.87845500 -0.98868100

C -2.47981600 -2.65786100 -1.43672000

C -3.19028300 -3.52909800 -2.23432000

H -4.27415200 -3.51386300 -2.19244600

C -2.51363600 -4.46779200 -3.03998100

H -3.06603100 -5.18227700 -3.64246900

C -1.12319500 -4.47080400 -3.04633200

C -0.33724600 -5.39240100 -3.82938000

H -0.86448300 -6.14477300 -4.41006300

C 1.01604900 -5.33216000 -3.85204400

H 1.59284500 -6.03513500 -4.44727900

C 1.72091600 -4.32259400 -3.11306100

C 3.10129100 -4.16884300 -3.14387800

H 3.70731700 -4.85284500 -3.73241200

C 3.69746300 -3.11142000 -2.45966500

H 4.76467500 -2.93366700 -2.52703300

C 2.89729100 -2.22358500 -1.73656800

C 0.96367600 -3.40611600 -2.32512000

C -0.43803000 -3.50961100 -2.25509600

C 3.51029800 -1.00726300 -1.14619100

C 2.93717700 0.24288000 -1.39122000

C 3.54789600 1.41925600 -0.95490700

C 4.73934200 1.31565400 -0.23132200

C 5.30626700 0.07681900 0.06669300

C 4.69244100 -1.08204600 -0.41015500

C -0.34016700 1.73328800 0.55016500

C -0.49958600 1.83015500 -0.83870700

C -0.61803200 2.87315500 1.31954700

C -0.91458000 3.01552500 -1.43934600

H -0.29378500 0.96736200 -1.47027900

C -1.05272600 4.05480400 0.72841200

H -0.49076000 2.83969800 2.39927900

C -1.19643700 4.12836600 -0.65483700

H -1.01122200 3.07055800 -2.51904200

H -1.26898100 4.92223200 1.34574500

H -1.51928700 5.05478800 -1.12108600

Si 0.24476700 0.15895200 1.40634500

H 1.36507000 0.55028800 2.31030900

H -0.77570600 -0.42445000 2.31918200

H 1.26705900 -0.82851900 0.55642900

H 2.01202200 0.29661000 -1.95737200

H 5.13368300 -2.05064300 -0.18936200

H 5.24012800 2.21798100 0.10838900

H -5.31050100 0.37492700 1.94483600

H -2.19585100 -2.48807100 1.24220300

H -4.35128900 -0.80362100 -2.05902900

C -5.94939200 0.86208600 -0.63270100

C -7.16771900 0.38434800 -1.14550300

C -5.68565200 2.23977300 -0.60640900

C -8.10249500 1.29692400 -1.62870100

C -6.64702600 3.12131000 -1.10436400

C -7.85909300 2.67067300 -1.61981400

H -9.04789100 0.92672900 -2.02175400

H -6.43988200 4.18967900 -1.08862500

C -3.50310800 -1.18960900 3.23680500

C -3.67949700 -2.43938000 3.86373400

C -3.13898200 -0.07127500 4.00782500

C -3.46452900 -2.54885300 5.23585000

C -2.92258500 -0.22909600 5.37760600

C -3.07384500 -1.45818300 6.01089100

H -3.61233500 -3.51471200 5.71568800

H -2.62885400 0.63913500 5.96494400

C 2.95896100 2.75197400 -1.27490800

C 2.74226700 3.12226000 -2.61708300

C 2.62217700 3.64763900 -0.24357500

C 2.17545500 4.36423400 -2.89670300

C 2.06229800 4.88152700 -0.57139700

C 1.82255400 5.25713000 -1.88820800

H 2.00738800 4.64205000 -3.93589200

H 1.78140300 5.55835100 0.23262900

C 6.54189500 -0.00250100 0.89948400

C 7.79306500 -0.19378900 0.29293000

C 6.44229700 0.12361800 2.29361600

C 8.93128100 -0.25459700 1.09527300

C 7.60413000 0.05393000 3.06256100

C 8.85662800 -0.13588000 2.48268500

H 9.90263100 -0.39548400 0.62478900

H 7.52698000 0.15218000 4.14369000

C -7.47332300 -1.09443300 -1.16540900

H -6.85274700 -1.62416200 -1.89737200

H -7.27618000 -1.55504800 -0.19158400

H -8.52135400 -1.27295700 -1.42329000

C -8.88574100 3.63310800 -2.16347900

H -9.02816600 3.49101800 -3.24116400

H -9.86036500 3.48482300 -1.68490100

H -8.58610300 4.67267300 -2.00002800

C -4.38520600 2.77315000 -0.05624800

H -4.33889500 2.67722800 1.03475100

H -3.52028000 2.23250900 -0.45376200

H -4.26080700 3.83201700 -0.30024400

C -4.12538300 -3.66327100 3.09592900

H -4.86568000 -3.41304800 2.33049200

H -3.28781800 -4.15167900 2.58497100

H -4.56719400 -4.39870300 3.77507100

C -2.81045200 -1.61620800 7.48730200

H -3.54766600 -2.27776400 7.95437100

H -1.82059000 -2.05445600 7.66352400

H -2.84292600 -0.65255300 8.00483100

C -2.98475400 1.30541500 3.40727400

H -2.63115300 1.27220900 2.37434000

H -3.94022700 1.84498900 3.40447900

H -2.27870800 1.90268400 3.99327400

C 3.12373600 2.22576000 -3.77376800

H 4.06446100 1.69929100 -3.58808800

H 2.36166800 1.46276200 -3.96997400

H 3.23731400 2.81363200 -4.68957900

C 1.20025300 6.59031400 -2.21705400

H 1.95097000 7.30067300 -2.58394300

H 0.43768700 6.49088100 -2.99746000

H 0.72694700 7.03490900 -1.33593100

C 2.83113600 3.32038400 1.21596300

H 2.66773000 2.26069400 1.42618500

H 3.84959700 3.56597900 1.54170000

H 2.13915700 3.89728700 1.83634800

C 5.10230300 0.33576800 2.95670000

H 4.69330300 1.32617300 2.72519100

H 4.36363700 -0.39625900 2.61302000

H 5.18699400 0.25285500 4.04411700

C 10.09868000 -0.23622700 3.33298700

H 10.35215800 -1.28467900 3.53215900

H 10.96091900 0.22038000 2.83625800

H 9.96217800 0.25868000 4.29951100

C 7.90933600 -0.31942800 -1.20697500

H 7.41157800 -1.22488400 -1.57278500

H 7.43522900 0.52795000 -1.71426800

H 8.95741800 -0.36283300 -1.51712800

**M-^5^Int-1**

Fe -0.35844200 0.55361100 0.89697300

N 0.88405300 2.00353100 1.81048000

N -1.83570500 1.62162900 2.00969600

C 2.98147000 1.37938700 0.72930500

C 3.14554600 0.02380400 1.02091200

C 3.97533400 -0.78770000 0.24604900

C 4.63688400 -0.22280300 -0.84818700

C 4.48958200 1.13010100 -1.16099900

C 3.65711400 1.92161000 -0.36599000

C 2.19390800 2.26744100 1.63183900

C 2.84581300 3.30776800 2.27433800

H 3.90455900 3.46313500 2.10153200

C 2.12669500 4.11443500 3.17834900

H 2.62246000 4.92112000 3.71158100

C 0.78037900 3.88006300 3.38226400

C -0.02746100 4.67720400 4.27405500

H 0.46261100 5.46617400 4.83909800

C -1.35710800 4.47058800 4.39408100

H -1.95607900 5.09008700 5.05691200

C -2.02780900 3.44964600 3.62927000

C -3.39836400 3.26821300 3.65848100

H -4.01174300 3.89086000 4.30446500

C -3.98995000 2.29756600 2.83706500

H -5.06252800 2.14030500 2.83603000

C -3.17840100 1.50917500 2.02856000

C -1.23818300 2.61035100 2.77515600

C 0.14659600 2.81756200 2.65968800

C -3.77595700 0.50924200 1.10666800

C -4.89729200 0.80673300 0.32821300

C -5.37429700 -0.10885700 -0.61241200

C -4.69889700 -1.31869000 -0.80107900

C -3.57068900 -1.62927500 -0.04298500

C -3.15936800 -0.72507100 0.93476700

C -1.09945100 1.25468400 -0.85016500

H -1.94459400 1.88960400 -1.04369600

C -0.13559800 0.50523800 -1.12530200

C 0.80620700 -0.25055200 -1.96818000

H 0.57756700 -1.31840000 -1.89548300

H 0.72813400 0.05359900 -3.01754500

H 1.83642500 -0.10756100 -1.63038700

C 0.28349600 -0.58183500 3.78746600

H 0.52287600 -1.50683400 4.32286500

H -0.62905000 -0.14763600 4.20889900

H 1.08633200 0.14533800 3.95091400

C 0.11074900 -0.81703600 2.34477600

C 0.07543100 -1.39808900 1.23520700

H 0.09993700 -2.26402900 0.59450800

H 5.30473500 -0.83883700 -1.44589100

H 3.54878900 2.97944600 -0.59050700

H 2.65270700 -0.40153300 1.88755400

H -5.38572400 1.77312700 0.42367200

H -5.05235200 -2.01029600 -1.56141200

H -2.31475600 -0.98430400 1.55853300

C 4.23944800 -2.20129100 0.65582600

C 5.05945500 -2.43143200 1.77863700

C 3.71203500 -3.28972200 -0.05401900

C 5.31739800 -3.73982600 2.17812900

C 4.00217200 -4.58759400 0.37506600

C 4.79277200 -4.83521100 1.49171700

H 5.95441800 -3.91141500 3.04396000

H 3.59927700 -5.42784100 -0.18758700

C 5.27017100 1.74176000 -2.27881800

C 6.58920300 2.16534400 -2.04227200

C 4.69635900 1.90496100 -3.54731100

C 7.30997400 2.74830500 -3.08259200

C 5.45016200 2.49213400 -4.56408000

C 6.75825100 2.91925900 -4.35215100

H 8.32960400 3.08130500 -2.89711900

H 5.00036900 2.62054300 -5.54667300

C -2.69223000 -2.81173500 -0.28746000

C -2.46435200 -3.75146200 0.73370700

C -1.96121900 -2.89276300 -1.48714800

C -1.48602500 -4.72849700 0.55628700

C -0.97865100 -3.87670300 -1.61745400

C -0.71375300 -4.79404400 -0.60224800

H -1.30986300 -5.44860500 1.35332500

H -0.40063900 -3.92323800 -2.53875800

C -6.56279200 0.22974300 -1.45029900

C -7.85600000 0.02609600 -0.94640200

C -6.38060500 0.75302300 -2.74035500

C -8.95262900 0.35292900 -1.74385000

C -7.50155100 1.07024200 -3.50613900

C -8.79593400 0.88043100 -3.02405400

H -9.95562900 0.19104100 -1.35355200

H -7.36074200 1.47904700 -4.50505800

C 7.22188800 1.99474700 -0.68195500

H 6.59670900 2.42648200 0.10715600

H 7.35436300 0.93464100 -0.43606300

H 8.20362600 2.47539200 -0.64320400

C 7.56924100 3.53474200 -5.46524700

H 8.12044700 4.41431800 -5.11596500

H 8.30545000 2.82141900 -5.85519500

H 6.93254900 3.84380700 -6.29979900

C 3.28565500 1.44608900 -3.81761900

H 2.56938400 1.92841700 -3.14347000

H 2.98948400 1.66813400 -4.84707100

H 3.18761200 0.36547200 -3.66219300

C 5.67926500 -1.28205200 2.53825300

H 6.18518100 -0.58428700 1.86211300

H 4.92557800 -0.70167000 3.08162200

H 6.41076800 -1.64658100 3.26533300

C 2.80649900 -3.10462900 -1.24610800

H 1.77119800 -2.94456700 -0.92284500

H 3.09264600 -2.24504100 -1.85751000

H 2.81379900 -3.99550100 -1.88194900

C 5.07905800 -6.24152000 1.95513800

H 6.15238300 -6.39936400 2.10738200

H 4.58140800 -6.44889800 2.90988600

H 4.72863900 -6.98106500 1.22881300

C -9.99336100 1.26402600 -3.85761700

H -10.88223800 0.69557600 -3.56644200

H -10.22826100 2.32854400 -3.73637800

H -9.81140100 1.08813700 -4.92276300

C -4.99336700 0.96880300 -3.29574200

H -4.38481600 1.58260000 -2.62236100

H -4.46099300 0.01918600 -3.42159700

H -5.03315300 1.46571300 -4.26931000

C -8.06047200 -0.54530700 0.43566300

H -7.53290400 -1.49803000 0.55451300

H -7.67133800 0.12893900 1.20709000

H -9.12227000 -0.71406000 0.63721200

C 0.38080200 -5.82230900 -0.73702000

H 1.17540600 -5.64074200 -0.00335000

H -0.00135800 -6.83446100 -0.56420900

H 0.83334600 -5.79676800 -1.73292700

C -3.23304100 -3.70694800 2.03282000

H -4.26195500 -3.36771700 1.88403700

H -3.25856900 -4.69599900 2.50008700

H -2.76495600 -3.01772000 2.74585500

C -2.19101800 -1.92774200 -2.62591000

H -3.14865900 -2.11835400 -3.12324200

H -2.20347400 -0.89077700 -2.27685700

H -1.40479500 -2.02591200 -3.38060100

**M-^5^Int-1ʹ**

Fe 0.15434400 -1.48558900 0.21508500

N -1.34411800 -2.30231200 1.42635900

N 1.33478200 -2.52048800 1.55417700

C -3.26366700 -0.98513000 0.69767400

C -2.64973900 0.27048600 0.73768400

C -3.20634300 1.36851600 0.08234600

C -4.40479300 1.20183500 -0.61689700

C -5.04267200 -0.03848200 -0.66690100

C -4.45861600 -1.12479900 -0.01390700

C -2.69075900 -2.13896800 1.43780000

C -3.53263600 -2.97788900 2.15138100

H -4.59880300 -2.78240000 2.15926600

C -2.99911300 -4.04442400 2.90054300

H -3.65370300 -4.70682000 3.45970900

C -1.62609400 -4.22067400 2.94597900

C -0.97413600 -5.26537100 3.70198700

H -1.60172600 -5.95667600 4.25854600

C 0.37349600 -5.39377200 3.72824200

H 0.84061800 -6.19144200 4.30004400

C 1.22208300 -4.48107200 3.00487100

C 2.60637400 -4.55222900 2.98110100

H 3.11441100 -5.33873500 3.53306800

C 3.35069500 -3.59671400 2.26669000

H 4.43454600 -3.60871100 2.26990800

C 2.67576200 -2.59328600 1.58493200

C 0.58895900 -3.43860200 2.26338200

C -0.80765400 -3.31651900 2.21016900

C 3.36677900 -1.47738200 0.88785100

C 4.20109300 -1.68029200 -0.20857400

C 4.75410100 -0.58787300 -0.88291900

C 4.45795500 0.70385200 -0.44313900

C 3.66206300 0.92924500 0.68563700

C 3.12260700 -0.17841800 1.33742700

C 0.70461700 -0.66955000 -1.43671500

C -0.51327100 -1.04456900 -1.56193400

H 1.56384700 -0.19061700 -1.88332400

C -1.74627400 -1.19810300 -2.36785400

H -1.55500300 -1.03234300 -3.43417200

H -2.18013600 -2.19391400 -2.23450500

H -2.49776200 -0.47636200 -2.03346200

H 2.51769300 -0.03745400 2.22792200

H 4.87881700 1.55053200 -0.97894900

H 4.40846600 -2.68949000 -0.55471600

H -1.73418300 0.40196800 1.30734700

H -4.85637700 2.05595400 -1.11593900

H -4.94201900 -2.09639600 -0.06983200

C 3.42477800 2.30477400 1.21660200

C 4.51621800 3.10494500 1.60896000

C 2.11525700 2.79895400 1.36396800

C 4.27841600 4.38160600 2.11565600

C 1.92306900 4.08291200 1.87539300

C 2.98956900 4.89206100 2.25516100

H 5.12696800 4.99181900 2.42055000

H 0.90547400 4.45281900 1.98003600

C 5.68736800 -0.80290000 -2.03010000

C 7.07143200 -0.81513800 -1.79298500

C 5.19469900 -0.99445000 -3.32993500

C 7.94167700 -1.02178500 -2.86272500

C 6.09671500 -1.19819800 -4.37358800

C 7.47359600 -1.21841300 -4.16043900

H 9.01403800 -1.03077300 -2.67690200

H 5.71180200 -1.34355100 -5.38124900

C -6.35060500 -0.19023900 -1.37239500

C -7.54169900 0.04893700 -0.66761100

C -6.39461900 -0.55927300 -2.72481200

C -8.76038500 -0.08931900 -1.32952700

C -7.63346200 -0.68632100 -3.35308800

C -8.82743400 -0.45873900 -2.67235200

H -9.68315400 0.09841500 -0.78371500

H -7.66571400 -0.96877000 -4.40371200

C -2.55716300 2.71090200 0.17029700

C -2.65400200 3.44800900 1.36024900

C -1.83419500 3.22740200 -0.92006900

C -2.01144600 4.68523500 1.44874000

C -1.21271900 4.46735500 -0.79381300

C -1.28253400 5.20997000 0.38487100

H -2.08466000 5.25102600 2.37551800

H -0.64052900 4.85807400 -1.63292200

C 5.94441500 2.61419000 1.52845300

H 6.02583200 1.55518500 1.79104000

H 6.36042800 2.73088800 0.52057200

H 6.58225200 3.18542500 2.20962600

C 2.76085400 6.26499500 2.83587800

H 2.89394500 6.25703800 3.92450000

H 3.46784600 6.99489900 2.42694100

H 1.74693300 6.62116500 2.62905700

C 0.89633000 1.99399300 0.98235000

H 1.00891200 1.51656400 0.00478600

H 0.69316600 1.19809400 1.71113600

H 0.01349000 2.63409500 0.94859200

C 3.71257300 -0.95835800 -3.60773800

H 3.16233000 -1.66033000 -2.97243800

H 3.30302400 0.04035900 -3.41254300

H 3.50158000 -1.20603800 -4.65199100

C 7.61852400 -0.60472900 -0.40176500

H 7.39612600 0.40350100 -0.03393600

H 7.17353100 -1.30561000 0.31308500

H 8.70385900 -0.73961200 -0.38250400

C 8.42932700 -1.47355100 -5.29917500

H 9.40282800 -1.00799800 -5.11587700

H 8.59853400 -2.54857400 -5.43593500

H 8.03763900 -1.08076100 -6.24302700

C -3.43504200 2.92144200 2.54023500

H -4.43594100 2.59190700 2.24128000

H -2.94136500 2.05598300 2.99615600

H -3.54281500 3.69037400 3.31071400

C -1.66239700 2.43094800 -2.18888000

H -1.06049900 1.53504000 -1.99744900

H -2.62072400 2.09596800 -2.59897000

H -1.15274800 3.02222600 -2.95508800

C -0.56683600 6.53255100 0.49598300

H -0.92103600 7.24224400 -0.26016000

H -0.71754100 6.98773900 1.48000900

H 0.51145900 6.40293000 0.34510600

C -5.12334300 -0.81993500 -3.49423100

H -4.45101500 0.04492500 -3.46244700

H -4.57034700 -1.66589800 -3.07095400

H -5.33651000 -1.04519600 -4.54315700

C -7.50616900 0.45822900 0.78507200

H -6.96162800 -0.27267200 1.39293500

H -6.99314000 1.41821900 0.91446400

H -8.51686000 0.55669400 1.19161600

C -10.15883200 -0.63090700 -3.35999900

H -10.56701300 -1.63315900 -3.18127300

H -10.89414400 0.09221900 -2.99244500

H -10.06741700 -0.50090600 -4.44287200

**M-^5^Int-1ʹʹ**

Fe 0.11910500 -1.48890100 -0.40512400

N -1.15598600 -2.62708200 -1.44972900

N 1.54499200 -2.35356200 -1.66476200

C -3.22589400 -1.77343900 -0.50207600

C -2.96550700 -1.83521900 0.86844700

C -3.70833900 -1.07806600 1.77861400

C -4.69447600 -0.22245500 1.28388600

C -4.93876000 -0.10630800 -0.08508700

C -4.20749100 -0.89827000 -0.96994300

C -2.51347000 -2.67096600 -1.44613700

C -3.23063500 -3.53218800 -2.25124200

H -4.31348800 -3.53223400 -2.19568500

C -2.54582400 -4.44134600 -3.08573900

H -3.09645200 -5.14922800 -3.69835800

C -1.15970700 -4.42889200 -3.11428100

C -0.37579800 -5.32331300 -3.93226600

H -0.90350200 -6.06422500 -4.52714800

C 0.97492700 -5.24950400 -3.96737800

H 1.55105800 -5.93142700 -4.58741600

C 1.68455400 -4.25556200 -3.20707200

C 3.06162100 -4.10281600 -3.24563300

H 3.66197300 -4.77062100 -3.85803900

C 3.66715600 -3.06167200 -2.53508800

H 4.73328700 -2.88060200 -2.60885600

C 2.87403900 -2.19716400 -1.78241600

C 0.92932800 -3.36269500 -2.38588500

C -0.46966600 -3.47809500 -2.30727500

C 3.49400900 -0.99556700 -1.16732700

C 2.93881200 0.26418700 -1.40473000

C 3.55818800 1.42881300 -0.95005500

C 4.74096700 1.30362500 -0.21538100

C 5.29018300 0.05500100 0.07430800

C 4.66759900 -1.09191100 -0.42077800

C -0.32471800 1.74816600 0.53010500

C -0.47563200 1.84286700 -0.85982900

C -0.60747000 2.88870900 1.29649500

C -0.88500500 3.02797600 -1.46486900

H -0.26844800 0.97883200 -1.48960500

C -1.03672200 4.07003600 0.70092900

H -0.48695000 2.85642600 2.37701900

C -1.17028900 4.14217700 -0.68348600

H -0.97432200 3.08185900 -2.54525000

H -1.25600400 4.93864700 1.31550300

H -1.48773200 5.06877600 -1.15303600

Si 0.25207500 0.17188900 1.38523100

H 1.36388900 0.55050900 2.30394100

H -0.77960900 -0.42001900 2.27933200

H 1.26452700 -0.80061500 0.52541300

H 2.02013200 0.33458900 -1.97930400

H 5.09600200 -2.06813300 -0.20864600

H 5.24875300 2.19616800 0.13946100

H -5.28868100 0.36689100 1.97707200

H -2.20173100 -2.51619300 1.23548500

H -4.39345400 -0.82344800 -2.03800000

C -5.95776200 0.85716500 -0.59369200

C -7.19000000 0.39010000 -1.08219200

C -5.67667800 2.23167600 -0.58286200

C -8.12169000 1.31015300 -1.55710700

C -6.63563200 3.12079800 -1.07179800

C -7.86140800 2.68079400 -1.56367600

H -9.07785100 0.94844200 -1.93156700

H -6.41552400 4.18667300 -1.06849200

C -3.47454900 -1.20613300 3.24646600

C -3.64475700 -2.45453300 3.87819100

C -3.09899100 -0.08673100 4.01050000

C -3.41241200 -2.56149500 5.24752900

C -2.86517000 -0.24240700 5.37774500

C -3.01021600 -1.46985000 6.01538900

H -3.55573900 -3.52611700 5.73117100

H -2.56263200 0.62654400 5.95947700

C 2.98543500 2.77069900 -1.26172400

C 2.78241600 3.15589500 -2.60203500

C 2.64705500 3.65923600 -0.22481000

C 2.22575600 4.40408900 -2.87410600

C 2.09717000 4.89973400 -0.54508900

C 1.86974200 5.28935000 -1.85990400

H 2.06809300 4.69315600 -3.91186800

H 1.81489200 5.57084100 0.26327800

C 6.51754200 -0.04725600 0.91674500

C 7.77165600 -0.24578600 0.31875800

C 6.40754000 0.06503100 2.31131900

C 8.90227900 -0.32732900 1.13010200

C 7.56186700 -0.02533900 3.08932000

C 8.81717700 -0.22248100 2.51792700

H 9.87600100 -0.47370700 0.66622000

H 7.47656100 0.06228000 4.17076800

C -7.51287000 -1.08509800 -1.08524500

H -6.90545700 -1.62880700 -1.81798500

H -7.31167000 -1.53899500 -0.10905000

H -8.56529200 -1.25406200 -1.33128700

C -8.88406600 3.65116100 -2.10066200

H -9.02965300 3.51398200 -3.17859700

H -9.85832400 3.50648100 -1.62040400

H -8.57792600 4.68835900 -1.93425900

C -4.36087700 2.75341900 -0.05846300

H -4.29444700 2.65845000 1.03154600

H -3.50902700 2.20381900 -0.47182800

H -4.23082500 3.81060700 -0.30641100

C -4.10254800 -3.67969300 3.11938800

H -4.84975300 -3.43025800 2.36048200

H -3.27172100 -4.17323500 2.60230400

H -4.53987000 -4.41101100 3.80581100

C -2.72895600 -1.62537500 7.48871800

H -3.46755300 -2.27635500 7.96829900

H -1.74205500 -2.07457000 7.65349800

H -2.74317600 -0.65948200 8.00281800

C -2.95017100 1.28962600 3.40729000

H -2.62502800 1.25587000 2.36503900

H -3.90113500 1.83666300 3.43195500

H -2.22360600 1.88002900 3.97485500

C 3.16855500 2.26868100 -3.76436800

H 4.10594300 1.73697100 -3.57675400

H 2.40548200 1.51014900 -3.97313700

H 3.29096400 2.86473000 -4.67373900

C 1.25770000 6.62918200 -2.18098800

H 2.01352600 7.33566600 -2.54480300

H 0.49383400 6.53989200 -2.96138100

H 0.78842200 7.07277000 -1.29721200

C 2.84424300 3.31822800 1.23318200

H 2.67074600 2.25832800 1.43384000

H 3.86282400 3.55211100 1.56695200

H 2.15392500 3.89632900 1.85431000

C 5.06411800 0.28390700 2.96527300

H 4.66577600 1.27975100 2.73848000

H 4.32192800 -0.43877300 2.60940800

H 5.13912900 0.19127700 4.05263500

C 10.05075400 -0.34548500 3.37755800

H 10.28717900 -1.39852300 3.57351300

H 10.92351200 0.10079700 2.88995000

H 9.91387700 0.14665000 4.34547500

C 7.89923800 -0.35772400 -1.18128000

H 7.39798000 -1.25606400 -1.55963700

H 7.43525400 0.49780300 -1.68413800

H 8.94950600 -0.40584300 -1.48328800

**M-^1^Int-2**

Fe -0.20372100 -0.38000900 -0.88859300

N -1.45882600 -0.73586100 -2.37983100

N 1.11048000 -0.98937600 -2.21717300

C -3.41260000 0.02478700 -1.19758300

C -3.68148200 -0.82705600 -0.12968900

C -4.15426900 -0.32135000 1.08052000

C -4.36573700 1.05184400 1.20166700

C -4.12087200 1.91823300 0.13509900

C -3.65998700 1.38913100 -1.07063000

C -2.78163600 -0.51367500 -2.43090200

C -3.52704100 -0.78229500 -3.58704900

H -4.59398200 -0.58749800 -3.56944400

C -2.91484500 -1.29648800 -4.71480700

H -3.48942300 -1.51906400 -5.60929600

C -1.53220500 -1.52763000 -4.68781300

C -0.77173000 -2.06618700 -5.78287500

H -1.29487200 -2.32466000 -6.69911600

C 0.56741500 -2.25437700 -5.67841300

H 1.13133700 -2.66747300 -6.50969400

C 1.28050100 -1.90086900 -4.48065600

C 2.66875900 -2.01389700 -4.32250200

H 3.28063100 -2.42228700 -5.12144000

C 3.23876900 -1.57278200 -3.14701300

H 4.31196800 -1.61337400 -2.99671200

C 2.44451100 -1.04994200 -2.11267900

C 0.55410300 -1.38557900 -3.39568100

C -0.85538500 -1.21686900 -3.49607800

C 3.10092500 -0.47448300 -0.90737400

C 3.64962100 0.80234300 -1.00757800

C 4.27444000 1.39914000 0.08837800

C 4.34275700 0.69051500 1.28762400

C 3.80993500 -0.59589600 1.40066200

C 3.19488500 -1.17577400 0.29179800

C -0.28975000 1.52587100 -0.89400200

C 0.28600800 1.10060700 0.18176600

H 0.50628600 -0.99660700 0.29749800

Si -0.35206300 -2.57028800 -0.00954600

H 0.60588100 -3.09508000 1.02568300

H -1.68612200 -2.85125600 0.60809700

C -0.09763300 -3.80726000 -1.42135300

C 1.16972700 -4.36265700 -1.65143200

C -1.10419100 -4.09108200 -2.35596900

C 1.42917700 -5.14909100 -2.77032800

H 1.97201400 -4.17678100 -0.93985900

C -0.85724600 -4.88453400 -3.47347700

H -2.09947500 -3.67145100 -2.21706700

C 0.41453100 -5.40963800 -3.68829100

H 2.42233500 -5.56287500 -2.92467100

H -1.65484800 -5.08276600 -4.18464000

H 0.61252400 -6.02084400 -4.56460800

H -3.48876800 -1.89029600 -0.22360500

H -4.71320600 1.45627100 2.14918500

H -3.43940700 2.05822100 -1.89715600

H 2.76581900 -2.16954800 0.38111800

H 4.82218900 1.14519300 2.15133000

H 3.57677100 1.34851600 -1.94452200

C -4.37223700 -1.24207500 2.23303000

C -5.57284500 -1.95677600 2.34646000

C -3.35224200 -1.40844900 3.18351100

C -5.73809400 -2.83671700 3.41557300

C -3.55303900 -2.30250100 4.23397300

C -4.73854200 -3.02419600 4.36801600

H -6.66978800 -3.39239400 3.50377600

H -2.75965400 -2.44306700 4.96574000

C -4.28186500 3.39239200 0.29166100

C -5.34387300 4.05847000 -0.34175000

C -3.35244900 4.11366600 1.06030400

C -5.45923000 5.43994400 -0.19959400

C -3.49925100 5.49717600 1.17013100

C -4.54551400 6.17780300 0.55159500

H -6.28546500 5.95400400 -0.68740600

H -2.77419100 6.05814600 1.75701000

C 3.89442400 -1.34123600 2.69074000

C 2.85966600 -1.22759400 3.62957600

C 5.00898400 -2.15194400 2.95865300

C 2.95621300 -1.93320600 4.82950700

C 5.06927900 -2.84128200 4.16755000

C 4.05006000 -2.74621900 5.11555300

H 2.15364900 -1.84547400 5.55914800

H 5.93344300 -3.46959200 4.37635600

C 4.87073100 2.76221900 -0.03717300

C 6.24408500 2.89720400 -0.29959100

C 4.06299400 3.90115200 0.09804300

C 6.78692300 4.17355200 -0.43151700

C 4.64370900 5.16234400 -0.04212500

C 6.00103900 5.31972600 -0.31069500

H 7.85123900 4.27800300 -0.63467200

H 4.01650100 6.04539900 0.06362500

C 6.12547100 -2.27611500 1.95022800

H 5.75158000 -2.64236700 0.98749100

H 6.59786200 -1.30601000 1.75662200

H 6.89770000 -2.96662600 2.30153200

C 4.12906900 -3.52375700 6.40576400

H 3.95349300 -4.59201700 6.23146900

H 5.11693000 -3.42665900 6.86922600

H 3.38225200 -3.17826000 7.12700500

C 1.66033100 -0.35727700 3.34907000

H 1.95947100 0.67741600 3.14488700

H 1.10782100 -0.70829600 2.46988000

H 0.97358000 -0.34811600 4.20033100

C 2.59362900 3.77079200 0.41293300

H 2.07388600 3.13246600 -0.30836500

H 2.44332400 3.30711900 1.39546900

H 2.10328700 4.74866100 0.42324600

C 7.12482100 1.67815200 -0.43201300

H 7.18499400 1.12682800 0.51355900

H 6.73157500 0.97969400 -1.17890900

H 8.14136400 1.95718400 -0.72451600

C 6.60787300 6.68919000 -0.48952700

H 6.78776200 6.90238000 -1.55023100

H 5.94844200 7.47173400 -0.10192100

H 7.57031500 6.76985000 0.02684000

C -2.06138900 -0.63384000 3.07901700

H -1.62845100 -0.68908700 2.07449600

H -2.21781500 0.43091800 3.29125100

H -1.32262500 -1.01453000 3.78961500

C -4.93692900 -3.96886500 5.52742400

H -5.70063500 -4.72031000 5.30426300

H -4.00798200 -4.49261800 5.77552700

H -5.25811300 -3.42778500 6.42598500

C -6.66655100 -1.78769600 1.31975000

H -6.91533700 -0.73114300 1.17182000

H -6.35862000 -2.18014600 0.34342100

H -7.57575400 -2.31514100 1.62298800

C -2.21325700 3.42183900 1.77280100

H -2.55600800 2.97243300 2.71321600

H -1.77733800 2.61478300 1.17616500

H -1.42044200 4.13492800 2.01900300

C -6.35870000 3.29514800 -1.15908500

H -5.92247600 2.91323800 -2.08928300

H -6.74049400 2.42781700 -0.60999500

H -7.20610400 3.93399300 -1.42495300

C -4.70313800 7.66905000 0.71494900

H -3.73566700 8.15762500 0.86843700

H -5.17541300 8.11966100 -0.16382300

H -5.33148200 7.90452500 1.58276100

H 0.77795100 1.39428300 1.09741200

C -0.68362800 2.73082200 -1.66932900

H 0.20256400 3.33266600 -1.90851000

H -1.17805100 2.47519600 -2.61264000

H -1.36258400 3.36954500 -1.08985700

**M-^3^Int-2**

Fe 0.24876500 -0.45308300 -0.68041600

N -1.08965800 -0.87183900 -2.38953700

N 1.55956100 -0.77657400 -2.23553900

C -3.15100500 -0.30275200 -1.26334100

C -3.34628600 -1.12149700 -0.15385300

C -4.03385800 -0.64483700 0.96276100

C -4.50526500 0.66934200 0.95815700

C -4.32015300 1.50149100 -0.14655400

C -3.64757000 0.99949000 -1.26077600

C -2.41763500 -0.78801200 -2.46394400

C -3.11423300 -1.09412500 -3.64624800

H -4.19597500 -1.01516400 -3.65177400

C -2.41990500 -1.49022800 -4.77030400

H -2.94384900 -1.74224600 -5.68826200

C -1.01805700 -1.54534800 -4.72584500

C -0.19662300 -1.90816800 -5.84785600

H -0.68478600 -2.18941900 -6.77673000

C 1.15549800 -1.89278800 -5.75643300

H 1.76998800 -2.16482200 -6.60991800

C 1.81273600 -1.50005000 -4.54038200

C 3.20637100 -1.39209000 -4.40968900

H 3.85101100 -1.64357000 -5.24693400

C 3.73707900 -0.94896500 -3.21570500

H 4.80708400 -0.83234200 -3.08272500

C 2.88591700 -0.64462100 -2.14443300

C 1.03017700 -1.17616700 -3.41733300

C -0.40163600 -1.21079400 -3.50482400

C 3.39357600 -0.10934500 -0.85461100

C 3.82520600 1.21348200 -0.78635200

C 4.17426700 1.78446500 0.43738700

C 4.13186400 0.99305200 1.58646900

C 3.72739300 -0.34123100 1.53337300

C 3.35030200 -0.88195100 0.30400300

C 0.21210500 1.50446700 -0.68265700

C -0.27113500 1.06735100 0.41662100

H -0.28073700 -1.22741600 0.57551100

Si 0.19471500 -2.78869900 -0.06871600

H 1.12120600 -3.17529700 1.04149300

H -1.12621800 -3.41620900 0.24994600

C 0.80331000 -3.79165400 -1.56195000

C -0.08877700 -4.19381300 -2.56688200

C 2.16643700 -4.04719700 -1.77335000

C 0.35558300 -4.81225400 -3.73293400

H -1.15501000 -4.01432500 -2.43786100

C 2.62101900 -4.65992500 -2.93767800

H 2.89231400 -3.76769100 -1.01230900

C 1.71538500 -5.04104700 -3.92518500

H -0.36005200 -5.10881800 -4.49509700

H 3.68378400 -4.84152400 -3.07509500

H 2.06727700 -5.51720600 -4.83623300

H -5.03833500 1.04727300 1.82737800

H -2.96184500 -2.13691200 -0.15256500

H -3.49213300 1.64069100 -2.12479000

H 3.83830800 1.82339400 -1.68631000

H 3.01358000 -1.91250500 0.26126500

H 4.40399900 1.42959600 2.54416800

C 4.52285500 3.23268000 0.51040100

C 5.76208400 3.68726300 0.02493200

C 3.59752700 4.14076400 1.04702600

C 6.05327200 5.04707100 0.07996900

C 3.92601800 5.49849000 1.07579600

C 5.14405600 5.97107800 0.59806400

H 7.01498700 5.39827700 -0.29024900

H 3.20498400 6.20416300 1.48419600

C 3.67804400 -1.18125200 2.76495900

C 4.69541200 -2.11256700 3.01549600

C 2.60451000 -1.04354300 3.66066700

C 4.62647400 -2.89707800 4.16710800

C 2.57113900 -1.84654100 4.79853300

C 3.57113900 -2.78108100 5.06809400

H 5.41800500 -3.61760600 4.36374600

H 1.73958800 -1.74271300 5.49334600

C -4.84918700 2.89752400 -0.15417300

C -6.15408100 3.14087400 -0.60538800

C -4.03859800 3.96134900 0.27566900

C -6.63230600 4.45193700 -0.62375700

C -4.55123700 5.25641800 0.24015700

C -5.84582800 5.52250200 -0.20665400

H -7.64439900 4.63981900 -0.97696400

H -3.92219900 6.08185000 0.56907700

C -4.29825400 -1.54320500 2.12571200

C -5.48176600 -2.29686800 2.15711900

C -3.36849200 -1.64366000 3.17145000

C -5.71649800 -3.14601700 3.23778700

C -3.63772500 -2.50585100 4.23382900

C -4.80617800 -3.26370200 4.28639400

H -6.63252400 -3.73357400 3.25870100

H -2.91093600 -2.59023800 5.03960300

C -6.49005600 -2.19415200 1.03817600

H -6.91478500 -1.18524700 0.97560700

H -6.03014700 -2.40234900 0.06599500

H -7.31300200 -2.89967900 1.18542700

C -2.10066100 -0.82791000 3.15624900

H -1.55584400 -0.94459100 2.21385100

H -2.31747300 0.24208600 3.26672700

H -1.43591700 -1.12457800 3.97241900

C -5.08928900 -4.16773500 5.46033800

H -5.56374300 -3.61226900 6.27877600

H -5.76437200 -4.98309400 5.18202100

H -4.16851700 -4.60907900 5.85522800

C -2.63968500 3.70545900 0.77866900

H -2.65754800 3.13530800 1.71565700

H -2.05331700 3.11137700 0.07057200

H -2.11075400 4.64475100 0.96635300

C -7.03128300 2.00409500 -1.07167100

H -6.57764100 1.47296400 -1.91616400

H -7.18033200 1.26276900 -0.27843500

H -8.01335800 2.36915600 -1.38623200

C -6.37140800 6.93643200 -0.22984900

H -6.45858700 7.34375900 0.78424800

H -5.70117900 7.59825600 -0.78978800

H -7.35995500 6.98800500 -0.69604200

C 6.77413800 2.71631300 -0.53538900

H 6.93896300 1.87663900 0.14846900

H 6.43904900 2.28849000 -1.48725000

H 7.73448200 3.21025700 -0.70966700

C 2.27207800 3.68295600 1.60956300

H 1.85141100 2.83362000 1.06390800

H 2.38347300 3.36625300 2.65449000

H 1.54019900 4.49654200 1.59183500

C 5.48233900 7.44078800 0.62927100

H 6.44561700 7.61425800 1.12187300

H 5.55740700 7.85136400 -0.38459000

H 4.72132800 8.01474600 1.16639900

C 1.49589900 -0.05850200 3.38603600

H 1.87476800 0.96623400 3.30327000

H 0.99493100 -0.28281400 2.43740000

H 0.74753700 -0.07890200 4.18328000

C 5.85344100 -2.26811000 2.05911400

H 5.52504100 -2.67931300 1.09746200

H 6.32858500 -1.30400400 1.84683300

H 6.61208200 -2.94142700 2.46884800

C 3.49549100 -3.65359000 6.29627200

H 3.22761000 -3.07012500 7.18390900

H 2.73380400 -4.43346300 6.17654900

H 4.45072300 -4.14985900 6.49286900

C 0.56851300 2.66066400 -1.54088100

H 0.02100800 3.56903800 -1.25923900

H 1.63966600 2.87640100 -1.44510200

H 0.37358700 2.44803000 -2.59760900

H -0.70907600 1.30043900 1.37512800

**M-^5^Int-2**

Fe 0.20822200 0.08129900 -1.02656400

N -1.18250000 -0.61129100 -2.48984200

N 1.48940300 -0.72702800 -2.43460700

C -3.14519500 -0.03779300 -1.23488100

C -3.33485200 -0.89281200 -0.15141700

C -3.90940000 -0.43476100 1.03371000

C -4.29295700 0.90433000 1.11987500

C -4.11862700 1.77699100 0.04350100

C -3.53913500 1.29507500 -1.13239400

C -2.51723300 -0.55931800 -2.48249300

C -3.29571200 -0.97635500 -3.56092700

H -4.37575900 -0.91143200 -3.49497400

C -2.66075800 -1.46632800 -4.70931900

H -3.24762100 -1.79161300 -5.56480900

C -1.27611900 -1.53440400 -4.75629800

C -0.52549300 -2.01424900 -5.88663300

H -1.07183000 -2.34455800 -6.76669300

C 0.82895300 -2.05439200 -5.86589200

H 1.37917300 -2.41760600 -6.73034400

C 1.58461400 -1.62544100 -4.71174600

C 2.96913800 -1.65718600 -4.63281700

H 3.55494200 -2.01685800 -5.47402900

C 3.60900100 -1.21364300 -3.44951700

H 4.68917200 -1.22946900 -3.35619400

C 2.84208300 -0.76350400 -2.39690700

C 0.85415200 -1.15056600 -3.58673200

C -0.54605400 -1.09989600 -3.61125400

C 3.42615800 -0.28957100 -1.11240800

C 3.89235000 1.01608900 -0.98022700

C 4.34221800 1.49494800 0.25269700

C 4.33845800 0.63244800 1.35080200

C 3.87201800 -0.67811600 1.24078700

C 3.41347700 -1.12610600 0.00262200

C 0.41791000 1.93613700 -0.47332500

C -0.05421600 1.27071500 0.50763600

H -0.18150200 -1.37298300 0.35833400

Si -0.11955200 -2.86591600 0.50686400

H 1.12006400 -3.16392700 1.26536300

H -1.32441600 -3.23744100 1.29092900

C -0.11565800 -3.70732400 -1.15665100

C 1.08812500 -4.00778800 -1.80705900

C -1.31694000 -3.98687700 -1.82271200

C 1.09403500 -4.55222200 -3.08695100

H 2.03861700 -3.80367900 -1.32047900

C -1.31620300 -4.52937100 -3.10299400

H -2.26979300 -3.77438000 -1.34369200

C -0.10894000 -4.80822600 -3.73795600

H 2.03817500 -4.75419400 -3.58302600

H -2.25724000 -4.72029600 -3.60963900

H -0.10547600 -5.21595500 -4.74419400

H -3.04584400 -1.93671700 -0.23256100

H -4.75399400 1.27139900 2.03380500

H -3.39495700 1.96735100 -1.97405700

H 3.03540300 -2.14018800 -0.09096700

H 4.69084600 0.99360400 2.31387200

H 3.87060000 1.67902400 -1.84127700

C -4.13768400 -1.38414700 2.16248900

C -5.28244100 -2.19651700 2.16360800

C -3.20527400 -1.47863300 3.20598400

C -5.47177800 -3.09854100 3.20974500

C -3.42642300 -2.39606400 4.23245600

C -4.55180300 -3.21785600 4.25010900

H -6.36127200 -3.72587500 3.21163300

H -2.69839200 -2.47136900 5.03800600

C -4.58487800 3.19308700 0.13458100

C -5.85292800 3.53450300 -0.36095500

C -3.76436300 4.17504300 0.71082200

C -6.27997300 4.85932000 -0.27526700

C -4.22637400 5.48887900 0.77754700

C -5.48188500 5.85072700 0.29213500

H -7.26238600 5.12322400 -0.66227300

H -3.58655200 6.25087500 1.21890100

C 3.83089300 -1.58502900 2.42439600

C 2.80784200 -1.43741100 3.37569600

C 4.79604300 -2.59084400 2.57589700

C 2.76605700 -2.30569000 4.46447900

C 4.72396200 -3.43724100 3.68285300

C 3.71513500 -3.31356400 4.63513200

H 1.97205100 -2.19283400 5.20064300

H 5.47796900 -4.21279800 3.80328700

C 4.77151800 2.91684600 0.39315600

C 5.92703000 3.37429900 -0.26816800

C 4.01296700 3.80569500 1.17372200

C 6.29457100 4.71191500 -0.14651000

C 4.41147500 5.14165600 1.25908100

C 5.54424800 5.61564400 0.60599800

H 7.19398000 5.06003500 -0.65130400

H 3.81525700 5.82805400 1.85752600

C 5.90180000 -2.75797700 1.56159200

H 5.50996500 -3.09871100 0.59633300

H 6.41793100 -1.80986000 1.37607900

H 6.63932700 -3.49018200 1.90287600

C 3.63379600 -4.25567300 5.81037700

H 3.36483900 -3.72440500 6.72949500

H 2.87166700 -5.02664200 5.64357600

H 4.58752200 -4.76459500 5.98031400

C 1.76224100 -0.36204800 3.21589900

H 2.20059700 0.63986500 3.28367400

H 1.27709300 -0.42143900 2.23543100

H 0.99303800 -0.44581800 3.98867500

C 2.78614300 3.36076400 1.93576000

H 2.25262400 2.54993400 1.43307100

H 3.05687400 2.99943400 2.93616200

H 2.08994400 4.19505000 2.06872400

C 6.78115400 2.43952400 -1.09250000

H 6.92826800 1.47955900 -0.58761600

H 6.32043400 2.22177000 -2.06282400

H 7.76305400 2.88233000 -1.28382000

C 5.94513700 7.06732200 0.68799400

H 7.02611600 7.17511600 0.82685800

H 5.67989700 7.59991900 -0.23329300

H 5.44526500 7.57352600 1.51951200

C -1.97711100 -0.60383700 3.22072600

H -1.42092700 -0.68591100 2.28108700

H -2.23908000 0.45417100 3.34325400

H -1.30927100 -0.88382700 4.04018500

C -4.75371100 -4.22983200 5.35027400

H -4.27798900 -5.18487700 5.09616800

H -4.31606400 -3.88631600 6.29303900

H -5.81671900 -4.42695500 5.52124700

C -6.30002500 -2.09397900 1.05323400

H -6.69111500 -1.07405600 0.96426000

H -5.85916000 -2.34663300 0.08252200

H -7.14258800 -2.76881100 1.22974700

C -2.39976800 3.81785300 1.24404600

H -2.47119900 3.10653900 2.07574400

H -1.78546800 3.33398200 0.47811600

H -1.87177500 4.70616000 1.60357300

C -6.74933100 2.48562900 -0.97406400

H -6.29328600 2.03827400 -1.86426800

H -6.93807100 1.66623600 -0.27113300

H -7.71244100 2.91490400 -1.26479400

C -5.97586300 7.27136300 0.40705500

H -5.15378200 7.98935800 0.32227800

H -6.71057000 7.50322500 -0.37038300

H -6.45930500 7.43969400 1.37722500

H -0.41796700 1.30142000 1.52368000

C 0.91912800 3.19605800 -1.07297300

H 2.00751500 3.25533700 -0.95617800

H 0.69490100 3.25288700 -2.14187300

H 0.48000100 4.06786800 -0.57395800

**M-^1^Ts-1**

Fe -0.23474100 -0.51474100 -0.85453000

N -1.45690700 -1.28394800 -2.18497200

N 1.11261300 -1.02329700 -2.17273100

C -3.45287800 -0.73896600 -0.96110700

C -3.55456500 -1.45136200 0.23134000

C -4.05644600 -0.83962600 1.38024800

C -4.46150900 0.49312800 1.31501600

C -4.37402700 1.22013900 0.12678200

C -3.88307900 0.58504200 -1.01403000

C -2.79628800 -1.35220500 -2.14621900

C -3.52653300 -1.96126700 -3.17500000

H -4.60740100 -1.99522500 -3.09087500

C -2.87907100 -2.50903800 -4.26784600

H -3.44013700 -2.98816100 -5.06498000

C -1.48061600 -2.43550600 -4.33486000

C -0.68005500 -2.96401100 -5.40616300

H -1.18141800 -3.46253100 -6.23084900

C 0.67158400 -2.85070200 -5.39040700

H 1.26708400 -3.25823300 -6.20218700

C 1.35532700 -2.18719100 -4.31268400

C 2.74253800 -1.99833000 -4.25071300

H 3.38566100 -2.38450700 -5.03589900

C 3.26702800 -1.29976000 -3.18350400

H 4.33231700 -1.11146500 -3.10801300

C 2.43648300 -0.81331600 -2.16074300

C 0.59285900 -1.67577900 -3.25124700

C -0.82315100 -1.80662500 -3.26417300

C 3.03150900 -0.00214700 -1.06397500

C 3.31262200 1.34224700 -1.29878000

C 3.87545200 2.13758500 -0.29875100

C 4.15122800 1.56151800 0.94166000

C 3.88494500 0.21286700 1.18969300

C 3.32880500 -0.56499800 0.17482900

C -0.60639200 1.35972700 -1.00061600

C 0.09572900 1.12640500 0.06189900

H 0.66519400 -0.44758300 0.34432900

Si 0.06850900 -2.56818700 0.25767900

H 1.12861500 -2.67253800 1.32170000

H -1.14449600 -3.12087100 0.95363500

C 0.59004400 -3.91549400 -0.97406300

C 1.94349800 -4.20410100 -1.20491800

C -0.34698100 -4.53805800 -1.81306700

C 2.34594400 -5.05628100 -2.22976000

H 2.70308300 -3.75561400 -0.56750300

C 0.04376900 -5.39786600 -2.83645600

H -1.40839100 -4.33933000 -1.66992600

C 1.39522700 -5.65373200 -3.05395600

H 3.40324100 -5.25558900 -2.38518300

H -0.70707300 -5.86024800 -3.47209400

H 1.70463700 -6.31935800 -3.85524100

H -3.20378500 -2.47684000 0.28416300

H -4.83215300 0.98012500 2.21356400

H -3.78587800 1.14542600 -1.93928200

H 3.10584600 -1.60992900 0.36688800

H 4.58832500 2.17181200 1.72854900

H 3.08140100 1.78308100 -2.26501900

C -4.09594000 -1.58957900 2.66856100

C -5.19651300 -2.39806900 2.98203700

C -3.00945300 -1.49127300 3.55339800

C -5.19712500 -3.10354200 4.18557700

C -3.04596800 -2.21434900 4.74386400

C -4.13109300 -3.02450700 5.07851500

H -6.05049900 -3.73371100 4.42893400

H -2.20149500 -2.14883400 5.42751700

C -4.72253400 2.66949200 0.08591300

C -5.85206200 3.10782000 -0.62616000

C -3.89700900 3.59966700 0.74097900

C -6.13384000 4.47126500 -0.67905400

C -4.20949100 4.95752600 0.65390300

C -5.32204800 5.41328100 -0.04806800

H -7.01129500 4.80841400 -1.22792400

H -3.56153900 5.67800100 1.14974100

C 4.19825200 -0.39892500 2.51437000

C 3.21611700 -0.44780900 3.51398600

C 5.47548900 -0.93185100 2.74921100

C 3.52729000 -1.03989700 4.73796600

C 5.74767500 -1.51674400 3.98445000

C 4.78386500 -1.58592200 4.98968400

H 2.76513000 -1.07756500 5.51369200

H 6.73701200 -1.93268500 4.16605700

C 4.19425000 3.57209300 -0.56552200

C 5.46755100 3.92025400 -1.04659500

C 3.22770100 4.56379000 -0.34627600

C 5.75058800 5.25934900 -1.30589600

C 3.54900900 5.89456000 -0.61794200

C 4.80333500 6.26225500 -1.09761100

H 6.73577000 5.52818400 -1.68307000

H 2.79657700 6.66279000 -0.45063000

C 6.54124100 -0.86966100 1.68191500

H 6.21068200 -1.36000400 0.75951500

H 6.78089000 0.16743200 1.41962400

H 7.46175200 -1.35637600 2.01688000

C 5.08755300 -2.25969700 6.30465200

H 4.93530700 -3.34339200 6.23123800

H 6.12658600 -2.09412100 6.60761300

H 4.43686100 -1.88925200 7.10289600

C 1.84263100 0.12810200 3.27565500

H 1.90076200 1.15980200 2.91054700

H 1.29389700 -0.45209500 2.52432600

H 1.25316800 0.12764200 4.19707200

C 1.86059800 4.20282000 0.17877700

H 1.35962000 3.47486800 -0.46739400

H 1.92913700 3.74189000 1.17157600

H 1.22183800 5.08731300 0.25952900

C 6.51556800 2.85918200 -1.28047200

H 6.76243700 2.33286100 -0.35134000

H 6.16301600 2.09993100 -1.98752900

H 7.43562200 3.29641700 -1.67900000

C 5.14329300 7.70608100 -1.37213100

H 5.61859400 7.82330100 -2.35216300

H 4.25014400 8.33799200 -1.35211700

H 5.84318100 8.09436100 -0.62250000

C -1.82296200 -0.61800300 3.22637900

H -1.41703300 -0.83644100 2.23273100

H -2.09602200 0.44436700 3.22442800

H -1.02344700 -0.76044900 3.95855800

C -4.14812800 -3.78160500 6.38329900

H -4.92256400 -4.55457300 6.38685600

H -3.18450300 -4.26654000 6.57233400

H -4.34661200 -3.10831100 7.22605500

C -6.36269200 -2.50830500 2.02998100

H -6.80515800 -1.52645900 1.82674600

H -6.05032700 -2.92122400 1.06404900

H -7.14380500 -3.15641200 2.43810800

C -2.68681300 3.16722700 1.53697600

H -2.97161400 2.87197200 2.55474100

H -2.17622900 2.31140400 1.08586600

H -1.96705900 3.98749800 1.62262300

C -6.76381900 2.12245800 -1.31833300

H -6.28836100 1.68201100 -2.20215200

H -7.02932100 1.29279700 -0.65462800

H -7.68709900 2.60991800 -1.64495300

C -5.65875200 6.88266200 -0.10139900

H -4.76589400 7.50134300 0.03235900

H -6.11896700 7.15195000 -1.05754500

H -6.36809000 7.15163100 0.69075900

H 0.59458500 1.58549800 0.90262800

C -1.22123900 2.45218000 -1.79887800

H -0.46589100 3.20191800 -2.06651900

H -1.67107500 2.08229900 -2.72579900

H -2.00185200 2.96990800 -1.22765500

**M-^3^Ts-1a**

Fe -0.34176000 -0.29772200 0.82088700

N 1.08910800 -0.69324000 2.49461000

N -1.56602000 -0.49384600 2.41113500

C 3.14269600 -0.37070000 1.25546700

C 3.25711400 -1.30704500 0.22948800

C 3.95012900 -0.99391200 -0.93980400

C 4.51383100 0.27710300 -1.07130900

C 4.41134200 1.22551200 -0.05289700

C 3.72714700 0.88660100 1.11600900

C 2.41921100 -0.71240100 2.51240100

C 3.13865200 -1.03019600 3.67929100

H 4.22267400 -1.03563400 3.64210400

C 2.46094500 -1.33908700 4.83934300

H 3.00036900 -1.59978200 5.74594200

C 1.05685800 -1.30912500 4.84493600

C 0.25588000 -1.61008900 5.99912700

H 0.75903400 -1.89311700 6.91939300

C -1.09658500 -1.54524100 5.94420800

H -1.69634300 -1.77877200 6.81919800

C -1.77374500 -1.15879100 4.73714100

C -3.16846200 -1.02942500 4.63803000

H -3.79545700 -1.25042600 5.49690800

C -3.72139700 -0.61146000 3.44634800

H -4.79266300 -0.48558000 3.33450400

C -2.89276800 -0.34714600 2.34660100

C -1.01316800 -0.87674000 3.58863500

C 0.42009900 -0.96573300 3.63703400

C -3.42933600 0.14745200 1.05036100

C -3.75124500 1.49627400 0.91634000

C -4.13035700 2.01752600 -0.32065900

C -4.21841800 1.15432400 -1.41412200

C -3.91696600 -0.20254300 -1.29583300

C -3.52013900 -0.69903000 -0.05362700

C -0.18195100 1.62930900 0.60768200

C 0.34190400 1.04200100 -0.41181600

H 0.37250900 -0.62178300 -0.48503700

Si -0.40941800 -2.62811200 0.29848600

H -1.37774400 -3.01797400 -0.78445000

H 0.86810500 -3.30210500 -0.11184700

C -0.97206800 -3.62217400 1.81920400

C -0.05071200 -4.00301900 2.80749200

C -2.32924000 -3.84789600 2.09531600

C -0.45922100 -4.57081500 4.01178500

H 1.01310800 -3.84448600 2.63558600

C -2.74954100 -4.41000300 3.29813900

H -3.07989000 -3.58558900 1.35220000

C -1.81396600 -4.77052400 4.26472900

H 0.28066200 -4.85114600 4.75704800

H -3.80931000 -4.57009800 3.47998600

H -2.13756500 -5.20909800 5.20481700

H 5.05456900 0.52790300 -1.98097300

H 2.80360800 -2.28703700 0.33753000

H 3.63929100 1.61854000 1.91497800

H -3.65650200 2.15993300 1.77205300

H -3.25977800 -1.74788800 0.03927800

H -4.50769300 1.55114300 -2.38404400

C -4.36863900 3.48103800 -0.47705000

C -5.49968200 4.08043000 0.10685300

C -3.44922900 4.26146400 -1.19567900

C -5.68926200 5.45188000 -0.03560100

C -3.67355100 5.63587500 -1.30817200

C -4.78435200 6.24914400 -0.73824400

H -6.56810700 5.91327600 0.41140800

H -2.95640000 6.24116900 -1.85932900

C -3.96717200 -1.09851500 -2.48826600

C -5.09129800 -1.90058400 -2.72348600

C -2.87463000 -1.12974800 -3.37162200

C -5.10972600 -2.72762200 -3.84788000

C -2.93055500 -1.96902600 -4.48101600

C -4.03912800 -2.77683600 -4.73621400

H -5.98445800 -3.34818100 -4.03238900

H -2.08398100 -1.99725600 -5.16474600

C 5.04589700 2.56912400 -0.20078900

C 6.40795400 2.72803100 0.09271100

C 4.28492400 3.66653800 -0.63726500

C 6.99117100 3.98774800 -0.05152400

C 4.90260000 4.90832600 -0.76785700

C 6.25535500 5.08987800 -0.47904500

H 8.04854300 4.10967200 0.17522800

H 4.31312000 5.75793100 -1.10823100

C 4.13120200 -2.01844100 -2.01161400

C 5.30210900 -2.79267500 -2.02820300

C 3.14265500 -2.21481400 -2.98775700

C 5.46474300 -3.75688600 -3.02221300

C 3.34348300 -3.18615400 -3.96746900

C 4.49682700 -3.96755500 -4.00204600

H 6.37017900 -4.36093200 -3.02881600

H 2.57350600 -3.34020800 -4.72121600

C 6.37197000 -2.59473700 -0.98130700

H 6.83224200 -1.60308900 -1.06369000

H 5.95748900 -2.66768600 0.03005000

H 7.16296900 -3.34358400 -1.08261400

C 1.86839400 -1.40940600 -2.97575300

H 1.21506900 -1.72405700 -2.15411500

H 2.06659000 -0.34091600 -2.83877500

H 1.31482200 -1.54054600 -3.91000500

C 4.69780700 -4.99825700 -5.08508000

H 5.08179200 -4.53514100 -6.00229900

H 5.41584200 -5.76476300 -4.77749700

H 3.75668000 -5.49687500 -5.33865500

C 2.82626500 3.49876900 -0.98250300

H 2.70355600 2.83669000 -1.84830000

H 2.26444700 3.04113800 -0.16231100

H 2.36395300 4.46047800 -1.22419400

C 7.23844000 1.55433300 0.55326600

H 6.79432800 1.07124300 1.43054900

H 7.30893200 0.78660700 -0.22579300

H 8.25426200 1.86791600 0.81104800

C 6.89369200 6.44913700 -0.62228700

H 6.69942200 6.87720400 -1.61193300

H 6.49717800 7.15225100 0.11980900

H 7.97798200 6.39665200 -0.48502800

C -6.50962300 3.25528600 0.86876200

H -6.79538200 2.35818900 0.30926000

H -6.10998000 2.91450800 1.83085600

H -7.41443000 3.83588500 1.07114100

C -2.23470300 3.65332700 -1.85858000

H -1.81537100 2.82191800 -1.28498700

H -2.48591800 3.26186700 -2.85251700

H -1.45090300 4.40551200 -1.99267100

C -5.01887600 7.73268700 -0.87693300

H -5.92894400 7.93592300 -1.45337800

H -5.14248900 8.20768400 0.10288500

H -4.18366000 8.22334300 -1.38564800

C -1.65193100 -0.28728200 -3.11120200

H -1.89560600 0.77928900 -3.05159100

H -1.19010700 -0.55543300 -2.15517700

H -0.90608300 -0.42232400 -3.90000500

C -6.26771200 -1.87374700 -1.77794600

H -5.99158100 -2.25391500 -0.78749700

H -6.64122700 -0.85377800 -1.63323400

H -7.08971400 -2.48825900 -2.15647500

C -4.06059200 -3.68561900 -5.93999200

H -3.83985800 -3.13239200 -6.85977400

H -3.30742000 -4.47687600 -5.84744700

H -5.03615900 -4.16598800 -6.06197300

C -0.46166200 2.95444000 1.21602400

H 0.08391600 3.77134700 0.72703300

H -1.53241800 3.17796200 1.13982700

H -0.21166800 2.95446400 2.28262000

H 0.82999000 1.19569300 -1.36259400

**M-^3^Ts-1**

Fe -0.41364600 -0.16759500 0.76772600

N 1.08675100 -0.56626900 2.39855400

N -1.58879000 -0.53730900 2.35426600

C 3.13037400 -0.11435400 1.15661400

C 3.21923600 -1.01387200 0.09494400

C 3.97966100 -0.70842100 -1.03469300

C 4.62954300 0.52798800 -1.09694500

C 4.55669600 1.44010600 -0.04178800

C 3.80626400 1.10228100 1.08755600

C 2.41402000 -0.49321400 2.40719300

C 3.16313300 -0.77494300 3.56689800

H 4.24431300 -0.70033200 3.52340900

C 2.51880900 -1.15681900 4.72232300

H 3.08165100 -1.39520600 5.62082400

C 1.11645500 -1.23601300 4.73426100

C 0.35338300 -1.62399000 5.88760700

H 0.88672500 -1.89326700 6.79493900

C -1.00046900 -1.65422600 5.84822200

H -1.57263900 -1.95075500 6.72258400

C -1.71579200 -1.28132400 4.65965700

C -3.11767100 -1.23616500 4.58475700

H -3.71510100 -1.52006700 5.44631300

C -3.71487400 -0.81560100 3.41663000

H -4.79345300 -0.75055000 3.32471400

C -2.92187600 -0.46634500 2.31369100

C -0.99168800 -0.91880200 3.50984600

C 0.44675800 -0.91113400 3.53894800

C -3.51519800 0.02367200 1.03995300

C -3.89838400 1.35896000 0.92190300

C -4.36289300 1.85754900 -0.29518700

C -4.44805400 0.99309800 -1.38862700

C -4.07031400 -0.34524300 -1.28837500

C -3.61088600 -0.82437100 -0.06133400

C -0.46110300 1.76471900 0.72320600

C 0.13178300 1.36157000 -0.34577600

H 0.32912000 -0.27591500 -0.55843100

Si -0.36299700 -2.47808100 0.11282000

H -1.36225200 -2.87788800 -0.93730800

H 0.92526200 -3.05447100 -0.40007000

C -0.78582000 -3.57485900 1.60955700

C 0.19890500 -3.92209500 2.54777000

C -2.11084400 -3.90978900 1.92799900

C -0.11909100 -4.55885800 3.74489200

H 1.24127800 -3.68084000 2.34303300

C -2.44160800 -4.54110500 3.12441200

H -2.90738700 -3.67922400 1.22319600

C -1.44507000 -4.86393200 4.04199100

H 0.66790800 -4.80943800 4.45161300

H -3.47968300 -4.78169400 3.33985000

H -1.69776000 -5.35437000 4.97817300

H 5.23213500 0.76907400 -1.96947400

H 2.71708400 -1.97347400 0.15560100

H 3.76143900 1.79628600 1.92325900

H -3.79263400 2.02895600 1.77106900

H -3.30091800 -1.86034700 0.01963800

H -4.79881700 1.37659200 -2.34373600

C -4.71135000 3.30047700 -0.44352200

C -5.88662000 3.80734500 0.13070200

C -3.85295200 4.15047600 -1.16401800

C -6.18879000 5.16105400 -0.02421200

C -4.18809200 5.49687100 -1.29038300

C -5.35293900 6.02154100 -0.73071700

H -7.10364400 5.55136800 0.41749700

H -3.52150300 6.15579900 -1.84409500

C -4.10100100 -1.24092800 -2.48119400

C -5.19646800 -2.08546800 -2.70757000

C -3.01487500 -1.23273600 -3.37110400

C -5.19164200 -2.91381100 -3.82975200

C -3.04653000 -2.07560700 -4.48011100

C -4.12436900 -2.92523900 -4.72531400

H -6.04368100 -3.56709700 -4.00827100

H -2.20523600 -2.07151600 -5.17062400

C 5.33760000 2.71447200 -0.06118600

C 6.72389300 2.67211700 0.16242100

C 4.69908200 3.94899600 -0.26906400

C 7.44871000 3.86416200 0.17384900

C 5.45892200 5.11748900 -0.24968300

C 6.83502800 5.09735800 -0.02795800

H 8.52210100 3.82591200 0.35001200

H 4.96178100 6.07157400 -0.41507700

C 4.17822300 -1.73721300 -2.10232200

C 5.35372600 -2.50631100 -2.09066000

C 3.20122000 -1.96589300 -3.08425000

C 5.53700800 -3.48516400 -3.06718700

C 3.42096000 -2.95409700 -4.04235600

C 4.58326500 -3.72250300 -4.05394200

H 6.44624800 -4.08322200 -3.04990600

H 2.65803600 -3.13218300 -4.79778900

C 6.40731700 -2.29894200 -1.02888500

H 6.89507200 -1.32327500 -1.13292900

H 5.97213900 -2.32855400 -0.02405300

H 7.18098400 -3.06971300 -1.09054600

C 1.93355600 -1.15319200 -3.12134800

H 1.40781600 -1.18078600 -2.16146000

H 2.14743000 -0.10137100 -3.34144600

H 1.24952400 -1.52828300 -3.88823900

C 4.80481600 -4.76983400 -5.11653900

H 5.19680800 -4.31903300 -6.03652200

H 5.52445400 -5.52599700 -4.78768100

H 3.87029100 -5.27910500 -5.37325800

C 3.22126600 4.02373100 -0.56304100

H 3.01462000 3.65202800 -1.57312400

H 2.62416900 3.41562400 0.12359300

H 2.86229000 5.05568000 -0.50702200

C 7.44264900 1.36227200 0.38269600

H 6.87467900 0.69250800 1.03568000

H 7.59406100 0.83152000 -0.56482500

H 8.42750900 1.52790400 0.82939300

C 7.62758500 6.37983400 0.01922300

H 7.27653700 7.09319600 -0.73368900

H 7.53070000 6.86441000 0.99844900

H 8.69250400 6.19855400 -0.15578400

C -6.82322700 2.90904900 0.90311400

H -7.04245100 1.98975700 0.34958700

H -6.38790400 2.60500000 1.86205300

H -7.76892900 3.41783200 1.11162400

C -2.58591600 3.62818400 -1.79915700

H -2.05348200 2.93420600 -1.14131700

H -2.80332000 3.08543600 -2.72739500

H -1.91031600 4.45126800 -2.05207800

C -5.69320600 7.48110700 -0.90209900

H -5.87206400 7.72134600 -1.95663700

H -6.59309400 7.75091900 -0.34104000

H -4.87511900 8.12295600 -0.55636800

C -1.82821900 -0.33578200 -3.12584200

H -2.11794000 0.72136400 -3.10381900

H -1.36279200 -0.55423600 -2.15828100

H -1.07095400 -0.46497000 -3.90527000

C -6.36697000 -2.09703400 -1.75458500

H -6.06136400 -2.41966900 -0.75257500

H -6.80339800 -1.09762500 -1.64535200

H -7.15058800 -2.77592600 -2.10331900

C -4.12043300 -3.85159900 -5.91580100

H -3.62487200 -3.39232200 -6.77733200

H -3.58561700 -4.78130100 -5.68643700

H -5.13831600 -4.12204200 -6.21417900

C 0.76426200 1.79116000 -1.62017300

H 1.82653300 1.52175400 -1.62753700

H 0.29395300 1.29799900 -2.47733600

H 0.67375800 2.87475300 -1.74735700

H -0.77589700 2.68527800 1.19597900

**M-^5^Ts-1**

Fe -0.00733600 -0.69282800 -0.74595600

N -1.28600400 -1.31236000 -2.24254200

N 1.35355500 -1.12140300 -2.28140400

C -3.27194000 -0.70222800 -1.04657100

C -3.68124000 -1.36314300 0.10940600

C -4.20955700 -0.64847900 1.18710300

C -4.35606700 0.73438000 1.07656400

C -3.95888300 1.41660600 -0.07596200

C -3.40272200 0.68447700 -1.12588600

C -2.63024000 -1.44508300 -2.16663900

C -3.35890000 -2.19098400 -3.07065000

H -4.43607200 -2.25503000 -2.96563100

C -2.68845700 -2.84062000 -4.13796000

H -3.24782500 -3.42957900 -4.85948800

C -1.31338100 -2.71743500 -4.25464400

C -0.51575700 -3.35505400 -5.27881900

H -1.03177600 -3.93380300 -6.04093100

C 0.83650400 -3.26611300 -5.28905300

H 1.41378000 -3.77228900 -6.05877100

C 1.54649200 -2.52091700 -4.28051300

C 2.92595800 -2.43157400 -4.18636300

H 3.55157500 -2.93913000 -4.91628800

C 3.51267800 -1.68951500 -3.14800100

H 4.58849100 -1.58738700 -3.06432700

C 2.68837800 -1.04863700 -2.22832500

C 0.76864000 -1.84709600 -3.29426000

C -0.62561500 -1.94149800 -3.28011900

C 3.23235400 -0.19509900 -1.13584800

C 3.10378900 1.19024900 -1.22937200

C 3.56516900 2.02693300 -0.21250000

C 4.13040200 1.44342700 0.92349300

C 4.24008500 0.05823400 1.05129500

C 3.80538600 -0.75603700 0.00307800

C -0.14753100 1.24405800 -0.31690200

C -0.07306100 0.68917900 0.82923100

H 0.02035900 -0.75065700 0.90134000

Si 0.12915100 -2.72965000 0.68661300

H 1.14286500 -2.79752300 1.77975200

H -1.18871500 -3.11136800 1.26858800

C 0.62163700 -3.98984100 -0.61346200

C 1.96910800 -4.31548600 -0.81842000

C -0.33288200 -4.55418000 -1.47042000

C 2.35308600 -5.17001100 -1.84667100

H 2.73117000 -3.89815200 -0.16396800

C 0.04568700 -5.41628900 -2.49420400

H -1.38528500 -4.30306300 -1.35401600

C 1.39014100 -5.72104800 -2.68782200

H 3.40429200 -5.39906700 -1.99621500

H -0.70883400 -5.82943200 -3.15694200

H 1.68713200 -6.38164300 -3.49739600

H -3.55953900 -2.44032900 0.18741600

H -4.78413200 1.28928000 1.90777900

H -3.05918800 1.19857400 -2.01965000

H 3.89001500 -1.83588100 0.09092600

H 4.48593000 2.07897700 1.73072300

H 2.62805000 1.62036100 -2.10644500

C -4.58214000 -1.35364500 2.44808000

C -5.82955900 -1.98342500 2.56388700

C -3.67114600 -1.38635400 3.51625000

C -6.14674800 -2.64404000 3.75080800

C -4.02255600 -2.05885900 4.68501800

C -5.25485300 -2.69757500 4.81988900

H -7.11613900 -3.13014700 3.84227200

H -3.31418000 -2.08785900 5.51092300

C -4.12159100 2.89628800 -0.19127600

C -5.00634100 3.42685500 -1.14696700

C -3.39304600 3.76501200 0.64327400

C -5.13155900 4.81145000 -1.26484300

C -3.53880900 5.14224700 0.48353800

C -4.40194700 5.68680100 -0.46520700

H -5.82202300 5.21539100 -2.00265300

H -2.95984200 5.80889700 1.12055500

C 4.78370500 -0.54063100 2.30492000

C 3.95233000 -0.65318400 3.43102400

C 6.11399300 -0.98201800 2.36099600

C 4.46963900 -1.20763700 4.60062000

C 6.59584800 -1.52798800 3.55030200

C 5.78871700 -1.65149800 4.67986600

H 3.82539800 -1.29601500 5.47336100

H 7.63014900 -1.86379900 3.59671000

C 3.44434800 3.50877100 -0.34482400

C 4.14235500 4.17598300 -1.37119300

C 2.63533200 4.24450300 0.53788800

C 3.99864900 5.55425700 -1.50631500

C 2.50991900 5.62412400 0.35887200

C 3.17783500 6.29759900 -0.65818700

H 4.54560900 6.06545800 -2.29660300

H 1.87095400 6.18547400 1.03803600

C 7.01405800 -0.86803300 1.15446600

H 6.68726900 -1.53730300 0.35014500

H 7.00498800 0.14726600 0.74356700

H 8.04642600 -1.12610900 1.40790100

C 6.31968700 -2.27430200 5.94686900

H 5.83244500 -1.85441700 6.83260400

H 6.14135800 -3.35654200 5.95586000

H 7.39840300 -2.11826500 6.04751300

C 2.51656600 -0.18812300 3.37915700

H 2.45027300 0.89870900 3.24896600

H 1.98884700 -0.64312900 2.53390100

H 1.98504600 -0.45381500 4.29780300

C 1.89797800 3.58858800 1.68189200

H 1.61075800 2.56273000 1.44251100

H 2.52102800 3.55217000 2.58446800

H 0.99211700 4.15030300 1.93249400

C 5.06309000 3.43319600 -2.31182000

H 5.68530600 2.70989300 -1.77553600

H 4.50629500 2.87327700 -3.07134700

H 5.72246800 4.13189200 -2.83506800

C 3.01713400 7.78437600 -0.85209300

H 3.98611800 8.27117600 -1.00653400

H 2.39940600 8.00166500 -1.73170300

H 2.53853800 8.25119100 0.01407100

C -2.32797300 -0.70673700 3.39972400

H -1.78760500 -1.06439100 2.51657600

H -2.43740200 0.37847900 3.28604100

H -1.71132500 -0.89929900 4.28271300

C -5.59648700 -3.45177100 6.08076600

H -5.15506800 -4.45570500 6.06632900

H -5.21230300 -2.93963000 6.96903100

H -6.67827700 -3.56876100 6.19760000

C -6.81792300 -1.94097600 1.42391900

H -7.04437800 -0.90851000 1.13495000

H -6.41974000 -2.43775700 0.53210900

H -7.75549200 -2.43385800 1.69679900

C -2.45911200 3.24717400 1.71171500

H -2.99621600 3.06044300 2.65009800

H -1.98676800 2.30819000 1.41646400

H -1.67248500 3.97827900 1.92474000

C -5.83983500 2.53151400 -2.03402500

H -5.24659500 2.09609300 -2.84591500

H -6.26864100 1.69645300 -1.47134000

H -6.65926300 3.09549900 -2.48941900

C -4.54451400 7.18193600 -0.60380300

H -3.56941200 7.66070000 -0.74756800

H -5.17816000 7.44641100 -1.45563600

H -4.99298900 7.62037000 0.29533200

H -0.04453000 0.87144900 1.89419700

C -0.26407200 2.49226600 -1.10171100

H 0.49066200 3.22000800 -0.78223000

H -0.14173300 2.30896300 -2.17227300

H -1.25270000 2.93867600 -0.94019300

**M-^1^Int-3**

Fe 0.22485600 -0.42916300 -0.92091900

N -0.97794900 -0.95179300 -2.33697400

N 1.57695600 -0.77427300 -2.23691300

C -3.06371000 -0.43063000 -1.18692000

C -3.28020700 -1.22149300 -0.05927300

C -4.01705700 -0.72754400 1.01892800

C -4.52738700 0.57069400 0.94938100

C -4.33524500 1.36917200 -0.17854000

C -3.59662500 0.85671900 -1.24549000

C -2.32060800 -0.95375600 -2.36515600

C -3.02528700 -1.39826800 -3.49351100

H -4.10907200 -1.38588700 -3.45499300

C -2.36029200 -1.84027500 -4.62192500

H -2.90903600 -2.19178300 -5.49069900

C -0.95936300 -1.81515400 -4.62866400

C -0.13174800 -2.22657800 -5.73188100

H -0.61803600 -2.59974100 -6.62866800

C 1.22216300 -2.15882300 -5.66321900

H 1.83156600 -2.47959100 -6.50302400

C 1.88505500 -1.65942300 -4.48871200

C 3.27220700 -1.51717700 -4.34254400

H 3.94071600 -1.81122600 -5.14605200

C 3.76865300 -0.99244900 -3.16364200

H 4.83485300 -0.85752100 -3.01754600

C 2.90317700 -0.62717300 -2.12379500

C 1.08854500 -1.26830200 -3.40284600

C -0.32560000 -1.35440400 -3.46419000

C 3.39208400 -0.02398400 -0.85653500

C 3.72886900 1.32787100 -0.82813700

C 4.09462400 1.94523400 0.36821500

C 4.15842100 1.17302300 1.52903700

C 3.83868000 -0.18468300 1.51735400

C 3.44858900 -0.77606400 0.31582300

C 0.13324100 1.45184500 -0.70619200

C -0.39398200 1.09655500 0.46554500

H -0.63501500 0.00105000 0.66044700

Si 0.30872300 -2.69920900 -0.10753100

H 1.18905000 -2.97713300 1.08524200

H -0.98365000 -3.37568500 0.27705000

C 0.99388800 -3.83575300 -1.46703700

C 0.15023900 -4.31908800 -2.48016000

C 2.36838400 -4.07601700 -1.61851800

C 0.64837200 -4.99592200 -3.59056400

H -0.92397100 -4.15636100 -2.40215400

C 2.87814800 -4.74603200 -2.72757500

H 3.06113200 -3.73952200 -0.84964900

C 2.01875700 -5.20502300 -3.72291300

H -0.03359600 -5.35317100 -4.35800000

H 3.94895000 -4.91306200 -2.81407300

H 2.41278300 -5.72781300 -4.59032000

H 3.17200500 -1.82473700 0.30659000

H 4.44271900 1.64274800 2.46741500

H 3.66306000 1.91738200 -1.73896000

H -3.43586900 1.46897600 -2.12914600

H -5.10774900 0.95976900 1.78262800

H -2.88129500 -2.23027700 -0.02049400

C 3.87395800 -0.98454700 2.77619700

C 5.02596200 -1.70572000 3.12017200

C 2.74571000 -1.00730700 3.61042900

C 5.03218100 -2.44589000 4.30191000

C 2.79054500 -1.75887600 4.78329500

C 3.92251900 -2.48813300 5.14397300

H 5.92674600 -3.00425300 4.57127200

H 1.91601000 -1.77824500 5.43098900

C 4.34536300 3.41426700 0.40981000

C 5.48815600 3.95728800 -0.20157200

C 3.42272000 4.25523600 1.05418000

C 5.68597400 5.33592600 -0.16537800

C 3.65384300 5.63149700 1.05960100

C 4.77560500 6.19157500 0.45402700

H 6.57674300 5.75410600 -0.63064700

H 2.93528000 6.28306500 1.55347400

C -4.30870900 -1.59110200 2.20160100

C -5.59820000 -2.12316300 2.36574500

C -3.30381600 -1.88105000 3.13825200

C -5.86141800 -2.93766500 3.46673900

C -3.60833600 -2.69559600 4.22767800

C -4.88091900 -3.23287700 4.41108200

H -6.85867000 -3.35705700 3.58619500

H -2.82691700 -2.91997100 4.95121000

C -4.95385600 2.72594900 -0.26282700

C -6.25473500 2.85854500 -0.77361800

C -4.24363500 3.85872400 0.16113600

C -6.82561800 4.12817700 -0.85208300

C -4.84776200 5.11185500 0.06681800

C -6.13896600 5.26698300 -0.43421300

H -7.83283400 4.23086200 -1.25167800

H -4.29387400 5.99034200 0.39254500

C 6.24186200 -1.68175100 2.22607400

H 6.01768200 -2.10271300 1.23916700

H 6.59557000 -0.65777500 2.06028000

H 7.06198200 -2.25957800 2.66208500

C 3.93333300 -3.32422700 6.39939800

H 3.51006900 -4.31821400 6.20995200

H 4.95095000 -3.46610800 6.77691400

H 3.33809400 -2.86066700 7.19283100

C 1.49802500 -0.24931200 3.23379300

H 1.71155400 0.79881400 2.99829900

H 1.03689100 -0.68479200 2.33983400

H 0.76261500 -0.27585200 4.04291400

C 2.20167500 3.70383300 1.75316500

H 1.78210000 2.83911600 1.23144700

H 2.44636600 3.37627500 2.77142800

H 1.42290400 4.46885900 1.83360500

C 4.99052600 7.68448900 0.44563300

H 6.05537200 7.93447000 0.49334800

H 4.58925100 8.13276000 -0.47158500

H 4.49069800 8.16499500 1.29249600

C 6.50551000 3.06747900 -0.87594100

H 6.76466900 2.20953700 -0.24667400

H 6.12582200 2.66435600 -1.82194000

H 7.42259300 3.62203900 -1.09548800

H -0.71515300 1.69259600 1.31953800

C 0.43591200 2.82049300 -1.21392900

H 0.14035200 3.62294900 -0.52445600

H 1.50912200 2.92478700 -1.41048400

H -0.07402500 2.98382400 -2.17093800

C -6.69485600 -1.83601400 1.36722900

H -7.06089600 -0.80641600 1.45966800

H -6.34308500 -1.95768700 0.33744500

H -7.54630200 -2.50550300 1.52107500

C -1.90867000 -1.33166100 2.97998000

H -1.38547400 -1.81239200 2.14538700

H -1.91925600 -0.25417900 2.78092400

H -1.31604900 -1.50775900 3.88187800

C -5.19110200 -4.09085600 5.61217100

H -5.45111200 -3.47190000 6.47970400

H -6.03678700 -4.75819200 5.41856700

H -4.33088500 -4.70620200 5.89482500

C -2.84587400 3.72118800 0.70910800

H -2.83822900 3.11650800 1.62386800

H -2.18850800 3.21402400 -0.00403200

H -2.41405800 4.69886800 0.94320400

C -6.78666500 6.62785300 -0.49699200

H -7.50504600 6.69081200 -1.32059000

H -7.33015600 6.84605400 0.43049700

H -6.04208900 7.41838100 -0.63455300

C -7.02599700 1.64673900 -1.23823600

H -6.49654100 1.12126600 -2.04112600

H -7.16065100 0.92310200 -0.42628900

H -8.01522100 1.92923900 -1.60992600

**M-^3^Int-3**

Fe 0.30383700 -0.34525900 -0.76202800

N -1.10305100 -0.83543400 -2.38605400

N 1.55373300 -0.69550300 -2.27957700

C -3.16457000 -0.35044400 -1.21515100

C -3.29569200 -1.16847800 -0.09327200

C -4.01465200 -0.72863800 1.01951100

C -4.57272900 0.55189800 0.99991500

C -4.45125000 1.38234500 -0.11448000

C -3.74968800 0.91454700 -1.22646200

C -2.43318300 -0.82190100 -2.42354400

C -3.14500900 -1.21135800 -3.57277600

H -4.22915200 -1.18730700 -3.55076900

C -2.46112500 -1.62305600 -4.69656000

H -2.99547100 -1.93833300 -5.58857300

C -1.05695900 -1.62620800 -4.68386700

C -0.24947600 -2.02713300 -5.80237500

H -0.74802500 -2.36431600 -6.70666400

C 1.10329200 -1.99261200 -5.73241300

H 1.70866200 -2.30344100 -6.57892900

C 1.77411800 -1.53879400 -4.54570400

C 3.17052400 -1.44239500 -4.43311200

H 3.80343100 -1.74178500 -5.26339300

C 3.71823800 -0.95979100 -3.26436100

H 4.79114100 -0.86038700 -3.14263500

C 2.88312500 -0.58883700 -2.20018800

C 1.00643800 -1.15312000 -3.43330300

C -0.42705800 -1.20974900 -3.49572800

C 3.42761300 -0.03067900 -0.93373900

C 3.78418500 1.31511200 -0.87667200

C 4.19884400 1.88967600 0.32488100

C 4.28570400 1.08154600 1.46007900

C 3.94342000 -0.26987200 1.41914900

C 3.51222100 -0.82018400 0.21177600

C 0.19614200 1.55693500 -0.57491700

C -0.40124300 1.17242400 0.54577100

H -0.61259400 0.05638800 0.72191000

Si 0.33314600 -2.68764200 -0.03680800

H 1.28402500 -3.07432800 1.06900500

H -0.95816100 -3.32422000 0.41276900

C 0.87436700 -3.78640800 -1.49438000

C -0.05167100 -4.20469600 -2.46352200

C 2.22598200 -4.06517500 -1.74956300

C 0.34635600 -4.85757500 -3.62748200

H -1.11195300 -4.01017100 -2.30694700

C 2.63642000 -4.71168100 -2.91294500

H 2.98025300 -3.77772600 -1.01946300

C 1.69635600 -5.10776800 -3.86093600

H -0.39797500 -5.16500800 -4.35769800

H 3.69271900 -4.90881400 -3.07873400

H 2.01174100 -5.61210000 -4.77040300

H -5.13238000 0.90097200 1.86450700

H -2.84608600 -2.15658400 -0.08679600

H -3.65144300 1.54966600 -2.10331000

H 3.69256000 1.93368900 -1.76572000

H 3.22036800 -1.86452200 0.17929900

H 4.60413900 1.51945900 2.40276500

C 4.47240800 3.35310700 0.40608200

C 5.59529400 3.90412600 -0.23798100

C 3.59293000 4.18253800 1.12026200

C 5.81610500 5.27626100 -0.15955700

C 3.84668700 5.55536300 1.16633500

C 4.95039800 6.12108700 0.53662600

H 6.68969200 5.70012400 -0.65179600

H 3.15992100 6.19761600 1.71431700

C 3.98291600 -1.10311900 2.65607900

C 5.11209300 -1.87663600 2.95283300

C 2.87219800 -1.10580100 3.51648900

C 5.11651800 -2.64955300 4.11500300

C 2.91454500 -1.89020600 4.66607900

C 4.02707500 -2.67102400 4.98163000

H 5.99417400 -3.24987600 4.34675600

H 2.05382500 -1.89754800 5.33250700

C -5.09039000 2.73172000 -0.13423900

C -6.43629500 2.85518400 -0.50830800

C -4.34845600 3.87055500 0.22005200

C -7.02326100 4.12128800 -0.52217700

C -4.96877800 5.11747800 0.19205800

C -6.30631300 5.26387400 -0.17658500

H -8.06827700 4.21598100 -0.81070400

H -4.39387500 5.99982300 0.46811400

C -4.25030900 -1.63290600 2.18497900

C -5.49411500 -2.27446800 2.31009800

C -3.24844500 -1.84833200 3.14303600

C -5.71485000 -3.12204400 3.39430800

C -3.50966900 -2.70009600 4.21606600

C -4.73599100 -3.34443600 4.36107800

H -6.67614300 -3.62433400 3.48520800

H -2.73023400 -2.86668800 4.95711300

C -6.58471200 -2.06318300 1.28677100

H -7.03003100 -1.06520700 1.37891600

H -6.19869900 -2.14695900 0.26560800

H -7.38539500 -2.79792800 1.41313800

C -1.90035500 -1.18538600 3.02087400

H -1.31715400 -1.62857400 2.20549400

H -1.99644800 -0.11452400 2.81064100

H -1.32363700 -1.30466700 3.94222600

C -5.00800200 -4.23927100 5.54448100

H -5.44763000 -3.67110300 6.37353900

H -5.71026100 -5.03830100 5.28627900

H -4.08802000 -4.70211200 5.91509700

C -2.90594000 3.74324400 0.64135600

H -2.82085100 3.18152400 1.57948800

H -2.31463800 3.19781200 -0.10090600

H -2.44988400 4.72629800 0.79228600

C -7.24197900 1.63897500 -0.89666500

H -6.78629300 1.11619300 -1.74526900

H -7.29591200 0.91493700 -0.07607800

H -8.26361000 1.91493800 -1.17363700

C -6.94728000 6.62913700 -0.20714600

H -6.79940000 7.15696400 0.74149400

H -6.51258600 7.25183300 -0.99790100

H -8.02382900 6.56044900 -0.39007600

C 6.56389100 3.02852900 -0.99736200

H 6.83718200 2.13956300 -0.41907500

H 6.13365000 2.67349000 -1.94090500

H 7.47973400 3.57732400 -1.23582300

C 2.39017300 3.62866200 1.84856700

H 1.93900000 2.78164200 1.32425300

H 2.66582000 3.27625700 2.85055500

H 1.62483100 4.40130000 1.97314300

C 5.21974000 7.60349700 0.60727500

H 6.14785200 7.80942200 1.15322000

H 5.32940800 8.03577900 -0.39372300

H 4.40811700 8.13285600 1.11544600

C 1.64604200 -0.29233100 3.18798900

H 1.88792600 0.76306900 3.02176800

H 1.17534000 -0.65597200 2.26734500

H 0.90842600 -0.35060700 3.99340300

C 6.30681800 -1.87815300 2.03025600

H 6.04744000 -2.28073300 1.04424600

H 6.68822100 -0.86363900 1.86822900

H 7.11874500 -2.48632500 2.43951800

C 4.03332000 -3.52372800 6.22603300

H 3.80152700 -2.92900300 7.11686900

H 3.28086600 -4.31820500 6.16038400

H 5.00712300 -3.99793800 6.38138700

C 0.51039400 2.92912100 -1.06363600

H 0.16289000 3.72294200 -0.38861200

H 1.59157000 3.04979700 -1.19793600

H 0.05295100 3.08938000 -2.04722900

H -0.75849200 1.72604600 1.41288900

**M-^5^Int-3**

Fe -0.06220100 -0.53488200 -0.54682900

N -1.29900400 -1.39132600 -2.11973200

N 1.40324600 -1.25525200 -2.08021900

C -3.34775900 -0.57527200 -1.13179900

C -3.93378400 -1.10278900 0.01710600

C -4.63975700 -0.27203900 0.88753200

C -4.78012200 1.07885800 0.56727900

C -4.19764000 1.62564500 -0.57792000

C -3.46462000 0.78398700 -1.41559900

C -2.62647900 -1.46487600 -2.07856200

C -3.34263600 -2.34559100 -2.91076600

H -4.42375700 -2.37705400 -2.83481600

C -2.66138100 -3.13691500 -3.80612000

H -3.19555000 -3.82028900 -4.46050800

C -1.25810500 -3.06098300 -3.87508900

C -0.47436600 -3.86238500 -4.76974600

H -0.98832100 -4.54149500 -5.44367600

C 0.87789800 -3.78677700 -4.75815200

H 1.47403800 -4.40722100 -5.42072100

C 1.56010600 -2.90696200 -3.85494400

C 2.96269500 -2.82864600 -3.78206600

H 3.57156900 -3.43650700 -4.44507800

C 3.54335100 -1.99357300 -2.86285300

H 4.62119800 -1.90949000 -2.78484900

C 2.73299100 -1.22463500 -2.00040800

C 0.82390200 -2.08857400 -2.97985400

C -0.61729500 -2.16759100 -2.99550000

C 3.36344800 -0.38055900 -0.96205900

C 2.89528500 0.90547500 -0.69915000

C 3.49182100 1.70002500 0.28151100

C 4.57020500 1.17689700 0.99642900

C 5.03633700 -0.11954700 0.77161300

C 4.43313600 -0.88887800 -0.22039800

C -0.31274000 1.36053900 0.14093400

C -0.37169100 1.42524500 1.48086300

H -0.33427600 0.53574700 2.11217400

Si 0.19234300 -2.57948900 0.92202100

H 1.15108500 -2.52239300 2.08060300

H -1.05623800 -3.16426100 1.53080500

C 0.87025000 -3.99005800 -0.16355000

C 2.24935600 -4.21024300 -0.29966500

C 0.01969200 -4.76173300 -0.97204500

C 2.75630100 -5.15149100 -1.19268500

H 2.94275600 -3.63493800 0.31207600

C 0.51627300 -5.70784200 -1.86469100

H -1.05809800 -4.62209500 -0.89789300

C 1.89058800 -5.90502100 -1.98154800

H 3.83067700 -5.30020500 -1.27125800

H -0.16942100 -6.29199500 -2.47331900

H 2.28161600 -6.64307200 -2.67696500

H -3.81807300 -2.15748400 0.25274100

H -5.34928300 1.72129200 1.23402800

H -2.98406400 1.19176100 -2.30045300

H 4.77681700 -1.90733700 -0.38367900

H 5.04686700 1.78133700 1.76345500

H 2.05835900 1.29807900 -1.26526200

C -5.21330200 -0.81270200 2.15417500

C -6.57393700 -1.14244100 2.23123300

C -4.37681200 -0.97304700 3.27122600

C -7.08306600 -1.63545800 3.43256300

C -4.92474900 -1.46655600 4.45411400

C -6.27353500 -1.80576900 4.55395700

H -8.13956000 -1.88982800 3.49464700

H -4.28006000 -1.58671600 5.32267100

C -4.35585400 3.07251900 -0.90853500

C -5.04093400 3.45158600 -2.07633500

C -3.82193800 4.05910800 -0.05778300

C -5.16415300 4.80703400 -2.38391100

C -3.95901400 5.40061500 -0.40945100

C -4.62372800 5.79689400 -1.56825900

H -5.70024400 5.09515200 -3.28621400

H -3.53248200 6.15919300 0.24484600

C 6.12378700 -0.69263300 1.61704800

C 5.78237300 -1.41095500 2.77533200

C 7.46783400 -0.50816300 1.26517500

C 6.80343800 -1.93862500 3.56337500

C 8.46080700 -1.05111400 2.08109100

C 8.14806000 -1.77098500 3.23215900

H 6.54262300 -2.49167400 4.46382500

H 9.50515600 -0.90423100 1.81236100

C 2.94153300 3.05290800 0.58089300

C 2.93133200 4.05242400 -0.40760200

C 2.38284700 3.30976300 1.84487300

C 2.33799100 5.28114200 -0.12628000

C 1.78972300 4.55008500 2.08058400

C 1.74405200 5.54343800 1.10654100

H 2.33090300 6.05152000 -0.89504800

H 1.33660900 4.73715700 3.05222200

C 7.83564600 0.27202200 0.02634800

H 7.38930400 -0.17225200 -0.87065300

H 7.46930200 1.30341400 0.08189800

H 8.92017400 0.30383800 -0.11261200

C 9.23105800 -2.37221600 4.09309000

H 9.00467600 -2.25325600 5.15785700

H 9.33702500 -3.44653000 3.89963900

H 10.20125200 -1.90414600 3.89990100

C 4.33663900 -1.59839800 3.16732300

H 3.82996400 -0.63469400 3.29217900

H 3.77355100 -2.14584200 2.40306600

H 4.25227900 -2.15080900 4.10728100

C 2.38405200 2.27358800 2.94360100

H 2.12740500 1.28394400 2.55498300

H 3.36362700 2.20082000 3.43102400

H 1.65104800 2.53190600 3.71311800

C 3.54722700 3.82767900 -1.76905300

H 4.48803300 3.27239500 -1.70243200

H 2.88022900 3.25440300 -2.42388900

H 3.74725100 4.78338800 -2.26268800

C 1.04258600 6.85392400 1.36050000

H 1.59050500 7.69433400 0.92143300

H 0.04058700 6.84843800 0.91364600

H 0.92601500 7.04546000 2.43162700

C -2.91411600 -0.61200100 3.19514200

H -2.36460600 -1.29978400 2.54143000

H -2.76743200 0.39062200 2.77972000

H -2.44822800 -0.64835400 4.18417800

C -6.83278200 -2.36557200 5.83822100

H -6.65836500 -3.44645200 5.90427900

H -6.36022100 -1.90513800 6.71198300

H -7.91244700 -2.20076600 5.91073400

C -7.48024600 -0.96243000 1.03697500

H -7.62790400 0.09857400 0.80370600

H -7.05560600 -1.42722900 0.14048800

H -8.46327300 -1.40626400 1.21976300

C -3.11256300 3.70513000 1.22601500

H -3.82370700 3.62653300 2.05809100

H -2.58474000 2.75214500 1.15350500

H -2.38128100 4.47585400 1.48927700

C -5.66580400 2.43000500 -2.99913000

H -4.91947300 1.95337200 -3.64534400

H -6.16163800 1.63058300 -2.43947600

H -6.40740900 2.90345300 -3.64956600

C -4.75394100 7.25892300 -1.91555400

H -3.76928900 7.72996400 -2.01504900

H -5.28919600 7.40002100 -2.85936700

H -5.29789900 7.80389900 -1.13545700

H -0.43734300 2.37443500 2.02510500

C -0.38445400 2.64781300 -0.65051300

H -0.26711900 3.54264900 -0.02206200

H 0.38588800 2.69811800 -1.43223400

H -1.34991600 2.73005300 -1.16878300

**M-^5^Ts-2**

Fe 0.47263600 -0.18929600 1.27184700

N -0.07789200 1.83576400 1.28897600

N 2.35170200 0.71678700 0.99033900

C -2.50829500 1.52191800 1.33809800

C -2.73740000 0.71796400 0.22071300

C -3.93427000 0.01016400 0.07922700

C -4.89773100 0.12835700 1.08439800

C -4.68228900 0.91030700 2.21957200

C -3.48040300 1.60615700 2.33695700

C -1.30227700 2.38405500 1.42243800

C -1.47792700 3.75648300 1.61784900

H -2.48578400 4.14588700 1.70780400

C -0.38113800 4.60305900 1.66731500

H -0.50955600 5.67131000 1.81521100

C 0.89882800 4.06714500 1.50947800

C 2.09106600 4.86941000 1.54074300

H 1.98614300 5.94106600 1.68401700

C 3.31350400 4.30506600 1.40274300

H 4.21301700 4.91361600 1.43214600

C 3.45670200 2.88723600 1.21665700

C 4.69776600 2.26000100 1.08686500

H 5.61082700 2.84701500 1.12671400

C 4.74337000 0.88997200 0.88049900

H 5.68841900 0.37997300 0.73149800

C 3.56165800 0.14678900 0.81743800

C 2.29768700 2.07672600 1.16681500

C 1.01171400 2.66980500 1.31779300

C 3.65013300 -1.29535700 0.47840700

C 4.51068000 -2.14037900 1.18206800

C 4.70496700 -3.45724700 0.76548600

C 4.03517500 -3.91486300 -0.37142200

C 3.15460800 -3.09519500 -1.08053800

C 2.97215100 -1.78358400 -0.63682500

C -0.06543800 -1.51962400 2.62733100

C 0.79599600 -2.48456400 2.98133900

H 1.74501700 -2.62936800 2.46487700

Si -0.43556800 -1.76797000 -0.28590100

H 0.32199700 -3.04625700 -0.31606800

H -1.83428000 -2.19341900 0.00886900

C -0.46661100 -1.11095800 -2.07298500

C 0.18667300 0.08179000 -2.41800500

C -1.14495000 -1.79384300 -3.09095900

C 0.18240800 0.56696600 -3.72503700

H 0.70553400 0.64930200 -1.64446300

C -1.16008500 -1.31466700 -4.39713700

H -1.68449600 -2.70847400 -2.85730800

C -0.49160300 -0.13616000 -4.72030400

H 0.69733900 1.49403100 -3.96343900

H -1.70241200 -1.85902800 -5.16626300

H -0.50406400 0.23804600 -5.74049400

H 2.30856800 -1.12584800 -1.18763000

H 4.20988100 -4.93277200 -0.70875800

H 5.03343000 -1.77404200 2.06207400

H -1.99108800 0.67014300 -0.56562400

H -5.84739800 -0.38836400 0.97565500

H -3.30354700 2.22225600 3.21511800

H 0.58873100 -3.18191400 3.79902100

C -1.40916300 -1.36629100 3.29853700

H -1.58741500 -2.14386600 4.05583200

H -1.50338700 -0.38696100 3.78477500

H -2.21704900 -1.41909200 2.55765800

C 2.40073900 -3.57874300 -2.27307800

C 2.48758100 -2.88510500 -3.49399000

C 1.54370500 -4.69293300 -2.16434300

C 1.70084600 -3.29620200 -4.56906700

C 0.76440400 -5.05701500 -3.26027600

C 0.81740800 -4.36413200 -4.46748800

H 1.76493600 -2.74575100 -5.50537900

H 0.08413900 -5.90124700 -3.16234200

C 5.63250700 -4.35562500 1.51444900

C 7.01696400 -4.25569000 1.30844400

C 5.11859000 -5.29771500 2.42019900

C 7.87077200 -5.09930100 2.01902100

C 6.00436300 -6.12239800 3.11169900

C 7.38389800 -6.03543800 2.92869800

H 8.94410100 -5.02339200 1.85538700

H 5.60696500 -6.85198000 3.81491600

C -5.73795600 1.03560900 3.26750000

C -6.57904300 2.16100800 3.27114800

C -5.89619400 0.03274500 4.23521000

C -7.56534200 2.26626600 4.24939200

C -6.89421700 0.17580800 5.20022900

C -7.73586200 1.28517800 5.22599200

H -8.22171300 3.13470500 4.24801800

H -7.01536400 -0.60194100 5.95177400

C -4.19638300 -0.83540000 -1.12276300

C -4.15300800 -0.27796100 -2.41279900

C -4.48714000 -2.20662100 -0.96711100

C -4.41275700 -1.09165000 -3.51584400

C -4.70786800 -2.98742900 -2.09978100

C -4.67397500 -2.44996600 -3.38501500

H -4.37432800 -0.64940800 -4.50931500

H -4.90736000 -4.04990900 -1.97261700

C 3.40392100 -1.70017000 -3.69617500

H 2.89272800 -0.76235900 -3.44905500

H 4.30523600 -1.76186800 -3.08012500

H 3.70986000 -1.63390600 -4.74505100

C -0.09112800 -4.73505100 -5.61106700

H -1.09108300 -4.30808600 -5.45995800

H 0.28828500 -4.35275600 -6.56389800

H -0.20856300 -5.82015000 -5.70009400

C 1.42125100 -5.50208800 -0.89342800

H 2.20627800 -6.26577400 -0.82689900

H 1.49015900 -4.87484700 -0.00035900

H 0.45799000 -6.01978500 -0.86387900

C 7.58023400 -3.25724200 0.32565500

H 7.10076300 -3.35024100 -0.65477500

H 7.41386500 -2.22715200 0.66225100

H 8.65704000 -3.39921400 0.19589000

C 3.63176800 -5.41456500 2.65174200

H 3.20649100 -4.46451500 2.99342500

H 3.10043000 -5.68437900 1.73241800

H 3.41101100 -6.17551200 3.40576800

C 8.32123000 -6.91472700 3.71849200

H 9.28203300 -7.03646200 3.20866700

H 8.52365200 -6.48159000 4.70567700

H 7.89313100 -7.90951700 3.87912700

C -4.99714600 -1.17939200 4.24522200

H -5.04505500 -1.72589800 3.29689700

H -3.94912000 -0.89486100 4.38979900

H -5.27593800 -1.86822200 5.04782800

C -8.79243600 1.43893800 6.29175200

H -8.46656600 2.14223100 7.06766100

H -9.72713200 1.82613700 5.87243400

H -9.00910600 0.48417200 6.78045900

C -6.42712200 3.23901100 2.22482200

H -5.46410700 3.75415600 2.31931700

H -6.46258400 2.81925900 1.21363800

H -7.21964700 3.98772100 2.31391700

C -4.55304100 -2.87363100 0.38757300

H -3.81837000 -2.45933200 1.08324600

H -5.54359000 -2.76063600 0.84550800

H -4.35718800 -3.94564500 0.29214800

C -4.85829700 -3.32827700 -4.59654900

H -5.17503900 -2.74723400 -5.46840400

H -3.91746300 -3.82989500 -4.85811000

H -5.60465500 -4.10947300 -4.41839100

C -3.80238000 1.16832300 -2.66756400

H -4.13163200 1.82805200 -1.86023100

H -2.71659200 1.28540400 -2.77535800

H -4.25969400 1.51355400 -3.59998100

**9. References**

1. Neese F. The ORCA program system. *WIREs Comput Mol Sci*. 2012; **2**(1): 73-8. doi: 10.1002/wcms.81

2. Neese F. Software update: the ORCA program system, version 4.0. *WIREs Comput Mol Sci*. 2018; **8**(1): e1327. doi: 10.1002/wcms.1327

3. Neese F. Software update: The ORCA program system—Version 5.0. *WIREs Comput Mol Sci*. 2022; **12**(5): e1606. doi: 10.1002/wcms.1606

4. Römelt M, Ye S, Neese F. Calibration of modern density functional theory methods for the prediction of ^57^Fe Mössbauer isomer shifts: meta-GGA and double-hybrid functionals. *Inorg Chem*. 2009; **48**(3): 784-5. doi: 10.1021/ic801535v

5. McWilliams SF, Brennan-Wydra E, MacLeod KC *et al.* Density functional calculations for prediction of Fe-57 Mossbauer isomer shifts and quadrupole splittings in beta-diketiminate complexes. *ACS Omega*. 2017; **2**(6): 2594-606. doi: 10.1021/acsomega.7b00595

6. Frisch MJT, G. W.; Schlegel, H. B.; Scuseria, G. E.; Robb, M. A.; Cheeseman, J. R.; Scalmani, G.; Barone, V.; Mennucci, B.; Petersson, G. A.; Nakatsuji, H.; Caricato, M.; Li, X.; Hratchian, H. P.; Izmaylov, A. F.; Bloino, J.; Zheng, G.; Sonnenberg, J. L.; Hada, M.; Ehara, M.; Toyota, K.; Fukuda, R.; Hasegawa, J.; Ishida, M.; Nakajima, T.; Honda, Y.; Kitao, O.; Nakai, H.; Vreven, T.; Montgomery J. A., Jr.; Peralta, J. E.; Ogliaro, F.; Bearpark, M.; Heyd, J. J.; Brothers, E.; Kudin, K. N.; Staroverov, V. N.; Kobayashi, R.; Normand, J.; Raghavachari, K.; Rendell, A.; Burant, J. C.; Iyengar, S. S.; Tomasi, J.; Cossi, M.; Rega, N.; Millam, J. M.; Klene, M.; Knox, J. E.; Cross, J. B.; Bakken, V.; Adamo, C.; Jaramillo, J.; Gomperts, R.; Stratmann, R. E.; Yazyev, O.; Austin, A. J.; Cammi, R.; Pomelli, C.; Ochterski, J. W.; Martin, R. L.; Morokuma, K.; Zakrzewski, V. G.; Voth, G. A.; Salvador, P.; Dannenberg, J. J.; Dapprich, S.; Daniels, A. D.; Farkas, Ö.; Foresman, J. B.; Ortiz, J. V.; Cioslowski, J; Fox, D. J. . Gaussian 09. *Inc, Wallingford CT*. 2009.

7. Legault CY. CYLview, 1.0b (Université de Sherbrooke, 2009). *Université de Sherbrooke*. 2009.

8. Lu T. sobMECP program. 2018. http://sobereva.com/286 (2018).

9. Harvey JN, Aschi M, Schwarz H *et al.* The singlet and triplet states of phenyl cation. A hybrid approach for locating minimum energy crossing points between non-interacting potential energy surfaces. *Theor Chem Acc*. 1998; **99**: 95-9. doi: 10.1007/s002140050309

10. Lu T, Chen F. Multiwfn: a multifunctional wavefunction analyzer. *J Comput Chem*. 2012; **33**(5): 580-92. doi: 10.1002/jcc.22885

11. Humphrey W, Dalke A, Schulten K. VMD: Visual molecular dynamics. *J Mol Graph*. 1996; **14**(1): 33-8. doi: 10.1016/0263-7855(96)00018-5

12. Lu T, Chen Q. Independent gradient model based on Hirshfeld partition: A new method for visual study of interactions in chemical systems. *J Comput Chem*. 2022; **43**(8): 539-55. doi: 10.1002/jcc.26812

13. Lu T, Chen Q. Interaction Region Indicator: A Simple Real Space Function Clearly Revealing Both Chemical Bonds and Weak Interactions**. *Chem–Methods*. 2021; **1**(5): 231-9. doi: 10.1002/cmtd.202100007

14. Chai J-D, Head-Gordon M. Long-range corrected hybrid density functionals with damped atom-atom dispersion corrections. *Phys Chem Chem Phys*. 2008; **10**(44): 6615-20. doi: 10.1039/b810189b

15. Schäfer A, Horn H, Ahlrichs R. Fully optimized contracted Gaussian basis sets for atoms Li to Kr. *J Chem Phys*. 1992; **97**(4): 2571-7. doi: 10.1063/1.463096

16. Schäfer A, Huber C, Ahlrichs R. Fully optimized contracted Gaussian basis sets of triple zeta valence quality for atoms Li to Kr. *J Chem Phys*. 1994; **100**(8): 5829-35. doi: 10.1063/1.467146

17. Hratchian HP, Schlegel HB. Accurate reaction paths using a Hessian based predictor-corrector integrator. *J Chem Phys*. 2004; **120**(21): 9918-24. doi: 10.1063/1.1724823

18. Weigend F, Ahlrichs R. Balanced basis sets of split valence, triple zeta valence and quadruple zeta valence quality for H to Rn: Design and assessment of accuracy. *Phys Chem Chem Phys*. 2005; **7**(18): 3297-305. doi: 10.1039/b508541a

19. Weigend F. Accurate Coulomb-fitting basis sets for H to Rn. *Phys Chem Chem Phys*. 2006; **8**(9): 1057-65. doi: 10.1039/b515623h

20. Barone V, Cossi M. Quantum calculation of molecular energies and energy gradients in solution by a conductor solvent model. *J Phys Chem A*. 1998; **102**(11): 1995-2001. doi: 10.1021/jp9716997

21. Cossi M, Rega N, Scalmani G *et al.* Energies, structures, and electronic properties of molecules in solution with the C-PCM solvation model. *J Comput Chem*. 2003; **24**(6): 669-81. doi: 10.1002/jcc.10189

22. Russell SK, Darmon JM, Lobkovsky E *et al.* Synthesis of aryl-substituted bis(imino)pyridine iron dinitrogen complexes. *Inorg Chem*. 2010; **49**(6): 2782-92. doi: 10.1021/ic902162z

23. Beromi MM, Younker JM, Zhong H *et al.* Catalyst Design Principles Enabling Intermolecular Alkene-Diene [2+2] Cycloaddition and Depolymerization Reactions. *J Am Chem Soc*. 2021; **143**(42): 17793-805. doi: 10.1021/jacs.1c08912

24. Hoberg H, Jenni K, Raabe E *et al.* Neue methoden zur herstellung von trialkylphosphan-alkadien-eisen(0)-komplexen. *J Organomet Chem*. 1987; **320**(3): 325-38. doi: 10.1016/0022-328x(87)85057-x

25. Hu M-Y, He P, Qiao T-Z *et al.* Iron-Catalyzed Regiodivergent Alkyne Hydrosilylation. *J Am Chem Soc*. 2020; **142**(39): 16894-902. doi: 10.1021/jacs.0c09083

26. Zhang D, Truhlar DG. Spin Splitting Energy of Transition Metals: A New, More Affordable Wave Function Benchmark Method and Its Use to Test Density Functional Theory. *J Chem Theory Comput*. 2020; **16**(7): 4416-28. doi: 10.1021/acs.jctc.0c00518
